# Effects of aftercrop tomato and maize on the soil microenvironment and microbial diversity in a long-term cotton continuous cropping field

**DOI:** 10.3389/fmicb.2024.1410219

**Published:** 2024-07-19

**Authors:** Shouyan Han, Xiaohui Ji, Liwen Huang, Gaijie Liu, Jingyi Ye, Aiying Wang

**Affiliations:** ^1^College of Life Sciences, Shihezi University, Shihezi, Xinjiang, China; ^2^Key Laboratory of Oasis Town and Mountain-basin System Ecology, Xinjiang Production and Construction Corps, Shihezi, Xinjiang, China

**Keywords:** crop rotation, continuous cropping, high-throughput sequencing, soil aggregate structure, physical and chemical factors

## Abstract

Long-term continuous cropping affects the soil microecological community and leads to nutrient imbalances, which reduces crop yields, and crop rotation can increase soil productivity. To study the effects of the cultivation of tomato (*Solanum lycopersicum*) and corn (*Zea mays*) on the microbial community, physical and chemical factors and the structure of aggregates in cotton (*Gossypium hirsutum*) long-term continuous cropping soils were examined. Four cropping patterns were established, including one continuous cropping pattern and three crop rotation patterns, and the diversity of the soil microecological community was measured using high-throughput sequencing. The physical and chemical properties of different models of soil were measured, and the soil aggregate structure was determined by dry and wet sieving. Planting of aftercrop tomato and corn altered the bacterial community of the cotton continuous soil to a lesser extent and the fungal community to a greater extent. In addition, continuous cropping reduced the diversity and richness of the soil fungal community. Different aftercrop planting patterns showed that there were very high contents of soil organic carbon and organic matter in the cotton-maize rotation model, while the soil aggregate structure was the most stable in the corn-cotton rotation model. Planting tomato in continuous cropping cotton fields has a greater effect on the soil microbial community than planting maize. Therefore, according to the characteristics of different succeeding crop planting patterns, the damage of continuous cropping of cotton to the soil microenvironment can be alleviated directionally, which will enable the sustainable development of cotton production.

## 1 Introduction

Xinjiang, China, with its vast territory and diverse ecological types, has created conditions for the cultivation of crops, as well as many forms of agricultural cropping systems. There are differences in the climate of different regions in Xinjiang. Thus, farmers in different regions should choose the planting mode that is suitable for local agricultural production to maximize their returns. Because Xinjiang is the primary area in which cotton (*Gossypium hirsutum*) is produced in China, the existence of large areas of long-term continuous cropping farmland has led to the occurrence of continuous cropping obstacles. The current methods of controlling continuous cropping obstacles include composting to improve the soil, changing the cropping system, and applying microbial agents (Tan et al., 2021; Zhang et al., 2022). Improvement of the cropping systems can be a widely effective approach, and rational crop rotation, relay cropping and intercropping can effectively improve the soil structure and reduce the number of pathogens, thus, promoting crop growth.

Changes in land use patterns can lead to changes in the soil properties, as well as its functions, and previous crop residues (roots and straw) as important and abundant biomass resources, may affect the physical, chemical and biological properties of the soil microenvironment and the growth of crops (Hu et al., 2020; Liu et al., 2023). Soil microorganisms play a unique and indispensable role in mediating the decomposition of organic matter in the soil and nutrient cycling and are important participants in the assessment of soil quality and ecosystem functioning in agriculture (Sui et al., 2022; Xu et al., 2023). Therefore, it is important to explore the differences of soil microbial communities in different planting patterns. In addition, study of the soil microbial community diversity in different cropping patterns will promote the development of eco-agriculture. Current methods that are used to study microbial diversity have evolved from traditional plate counting to high-throughput sequencing. High-throughput sequencing is advantageous because it is highly efficient and accurate, easily operated, quick, produces a large amount of information and has high-throughput output etc. (Pareek et al., 2011; Soon et al., 2013). The sequencing technology used in this study was Single Molecule Real-Time sequencing, also known as third-generation sequencing. It is a novel sequencing technique that can generate extremely long contiguous sequence reads (Takeda et al., 2019).

As crops and agricultural activities (including tillage, fertilization, and irrigation etc.) displace native vegetation, many biotic and abiotic parameters change, which may lead to the enrichment and decrease of some microorganisms (Kibblewhite et al., 2008; Hu et al., 2023). In this study, changes in the soil microbial population after planting other succeeding crops in long-term continuous cropping cotton fields were explored, which will help to explore the remediation of the soil microenvironment after long-term continuous cropping. In addition, different planting patterns have varying effects on the soil physical and chemical factors. For example, some studies have found that different cropping patterns differentially utilize soil moisture (Wang et al., 2022). Proper crop rotation can alter the soil nutrients, as well as promote root uptake and the utilization of nutrients. Thus, crop rotation can be used to regulate imbalances in the soil nutrients. In addition, it has been shown that long-term continuous cropping tends to reduce the soil particle size, disrupt soil aggregate structure, make the soil voids smaller and compacted, and increase the soil fractal dimension (Xing and Shangli, 2014). Diversified cropping systems can enhance soil cohesion, as well as stability (Yan et al., 2022), thus, promoting the maximum benefit of crops.

In this study, the soil microbial community, soil physical and chemical factors, and soil aggregate structure of four different cropping patterns were investigated. These included cotton continuous cropping, cotton-tomato (*Solanum lycopersicum*) rotation, cotton-corn (*Zea mays*) rotation, and corn-cotton rotation. This study was conducted for several reasons. The first was to study the difference in the soil microenvironment microbial community in long-term continuous cropping cotton fields after planting with different succeeding crops and differences in variation between bacterial and fungal communities. Secondly, this study was designed to explore the effects of planting of different succeeding crops on the soil physical and chemical factors. The third reason was to examine the differences in the stability of soil aggregates when different succeeding crops were planted and fourth, to explore the advantages of three succeeding crop planting patterns.

## 2 Materials and methods

### 2.1 Overview of the experimental field

The field trials were conducted in Shihezi City, Xinjiang, China, which is located in the desert early-maturing or extra-early-maturing cotton cultivation ecoregion at the northern foothills of the Tianshan Mountains in Xinjiang. The area is located in a typical temperate continental climate with annual precipitation that ranges from 125.0 to 207.7 mm and an average temperature of −16°C in January and 26°C in July. Crops are planted only once a year from the spring to fall, and no crops are planted in the winter. The site where the study was conducted had long-standing cotton continuous cropping and corn continuous cropping.

### 2.2 Experimental design

A field experiment with four models in 2021 included cotton succession (MM), cotton–maize rotation (MY), maize–cotton rotation (YM), and cotton–tomato rotation (MF). Four plots of each planting pattern were duplicated, and the nomenclature was the name of the planting pattern plus the number 1–4. For example: MY1, MY2, and MY3 etc. The cotton was Jinken 1402; the tomato was Yaxin No. 8, and the maize was Xinyu No. 8. The plots were 10 m^2^ (2 m × 5 m) with a 0.5 m buffering zone, and four field replicates were established. The plots were watered using drip irrigation, which was conducted once every 10 days on average. A total of 4.3 kg/km^2^ phosphorus (P) fertilizer was applied during each phase of watering before the cotton began flowering. The P and nitrogen (N) fertilizers were each applied simultaneously at 4.3 kg/km^2^ after flowering, and 11 g/km^2^ of foliar potash fertilizer was sprayed every 10 days on average. The four types of plots were fertilized in the same manner, and the practice was consistent across the four modes of fertilization.

### 2.3 Collection of the soil samples

**Figure 1:**
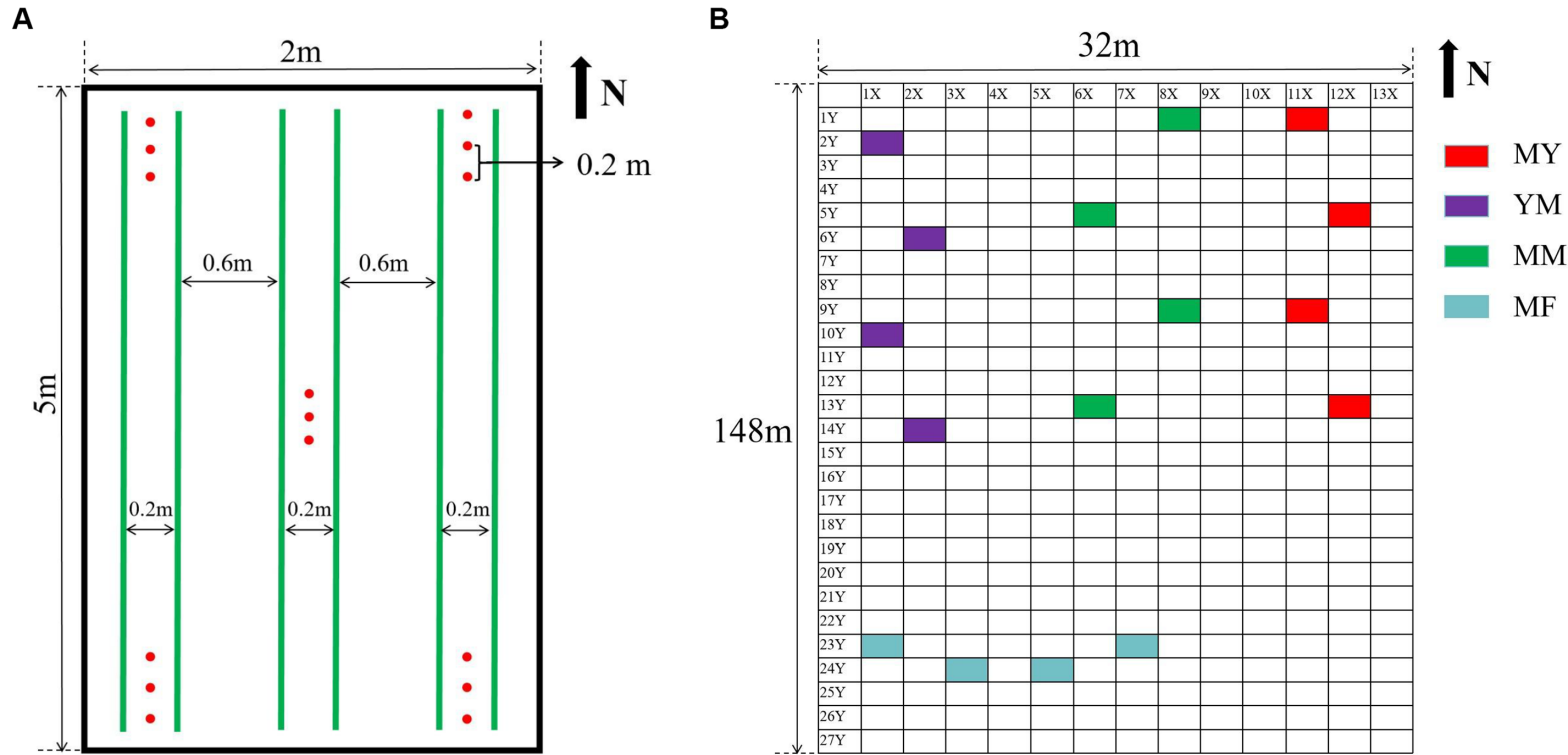
Collection of soil samples. **(A)** Five-point sampling method. In the picture, the green line represents the crop planting row, and the red dot is the sampling position. **(B)** Plot distribution of collected soil samples, each rectangular represents a plot except for X and Y axes.

The soil samples were collected in August 2021 using a five-point sampling method. This method selects the soil below 5 cm and above 10 cm at the four corners and center of each plot, Three samples were taken at each point, each 20 cm apart, and about 100 g of soil was sampled at each point (Figure 1), The five-point sampled soil was mixed as one sample. Therefore, about 1,500 g of soil was obtained from each plot, and each soil sample was divided into two portions. One portion was stored at −80°C as a high-throughput sequencing soil sample. The other was stored at room temperature to determine the physicochemical properties and agglomerate structure.

### 2.4 Determination of the soil physical and chemical factors

The soil electrical conductivity (EC), as well as its pH, were determined using the soil water intrusion assay. The content of effective P in the soil was determined as described by Experiment and Practice Instruction of Soil Science (Yuan, 2020). The soil total nitrogen (TN) was determined using the semimicro-Kjeldahl method. The contents of soil organic matter (OM), total P (TP), and total potassium (TK) were measured as described by the National Standard of the People’s Republic of China. The content of soil organic carbon (SOC) was determined by the oxidative-spectrophotometric method using potassium dichromate. The content of soil alkaline nitrogen (AN) was determined using the Sichuan Provincial Local Standard, while that of the soil fast potassium (AK) was determined using a Sinobestbio (Beijing, China) soil fast potassium kit.

### 2.5 Determination of the soil aggregate structure

The soil aggregate structure was determined by dry sieving and wet sieving. The dry sieve technique involved the weighing of 200 g of air-dried soil, which was then placed in the sieve and shaken manually for 2 min. The weight of each level of soil was then weighed. A total of 50 g of soil samples were weighed from each level based on the ratio used to determine the wet sieve aggregate structure using a TPE-100 Soil Aggregate Structure Analyzer (Zhejiang china). Finally, the percentage of each grain size in the dry sieve and wet sieve was calculated separately. The soil aggregate structure mass fraction was obtained by dividing the mass per particle size by the total mass. The formula for calculating the mean weight diameter (MWD) of the soil aggregate structure is as follows:


(1) $$MWD=\sum W_{i}X_{i}$$

where *W_i_* is mass fraction of the aggregate structure of group *i* particle size; *X_i_* is the average diameter of the group, and *i* is the particle size aggregate structure.

### 2.6 Extraction of the macrogenomes from the soil microenvironment and determination of the microbial diversity

The soil microbial DNA was extracted using a DNeasy PowerSoil ProKit kit (Qiagen, Hilden, Germany), Information on extracted soil DNA was uploaded to Supplementary Table 1. Agarose gel electrophoresis (1% concentration) was used to detect the integrity of extracted DNA. Negative control experiments with sterile double-distilled water instead of soil have been tested for the presence of contamination. The extracted macrogenes were sent to Biomarker Technologies (Beijing, China) for sequencing, the concentration and purity of extracted DNA were measured by NanoDrop 2000 (ThermoFisher, CA, United States). Those that were sequenced for bacterial diversity were the 16S rDNA sequences, and the sequences that were sequenced for fungal diversity were nucleotides of the ITS region. Synthesis of specific primers with Barcode based on full-length primer sequences, PCR amplification and purification, quantification and homogenization of the products to form SMRT Bell, sequencing after passing quality control. Three-generation microbial diversity are based on the PacBio (Menlo Park, CA, United States) sequencing platform, which utilizes Single Molecule Real-Time Sequencing to sequence the marker genes, followed by filtering, clustering or denoising of the CCS sequences. Finally, species annotation, as well as abundance analysis, were used to reveal the composition of the sample species.

### 2.7 Statistical analysis

The soil aggregate structure mass fraction was calculated using Microsoft Excel 2022 (Redman, WA, United States) and analyzed for significance using SPSS 25.0 (IBM, Inc., Armonk, NY, United States; *p* < 0.05). Finally, a bar graph was plotted using an Origin2022 paired comparison plot package (OriginLab, Northampton, MA, United States). Microbial community diversity analysis, redundancy analysis (RDA), and mapping were performed using R. In addition, a significance analysis (*p* < 0.05) was performed on alpha-diversity using SPSS 25.0. A non-metric multidimensional scaling (NMDS) analysis was performed at the operational taxonomic unit (OTU) level, with Pearson as the distance algorithm for bacterial sequencing and Binary-Jaccard as the distance algorithm for fungal sequencing. SILVA was used as the reference database to annotate the characteristic sequences, and QIIME was used to generate the abundance table of species at different taxonomic levels. Finally, Origin2022 was used to plot the community structure picture. The LDA Effect Size (LEfSe) was analyzed and plotted using R and set as LDA = 3.

## 3 Results

### 3.1 Soil physical and chemical factors

**Figure 2:**
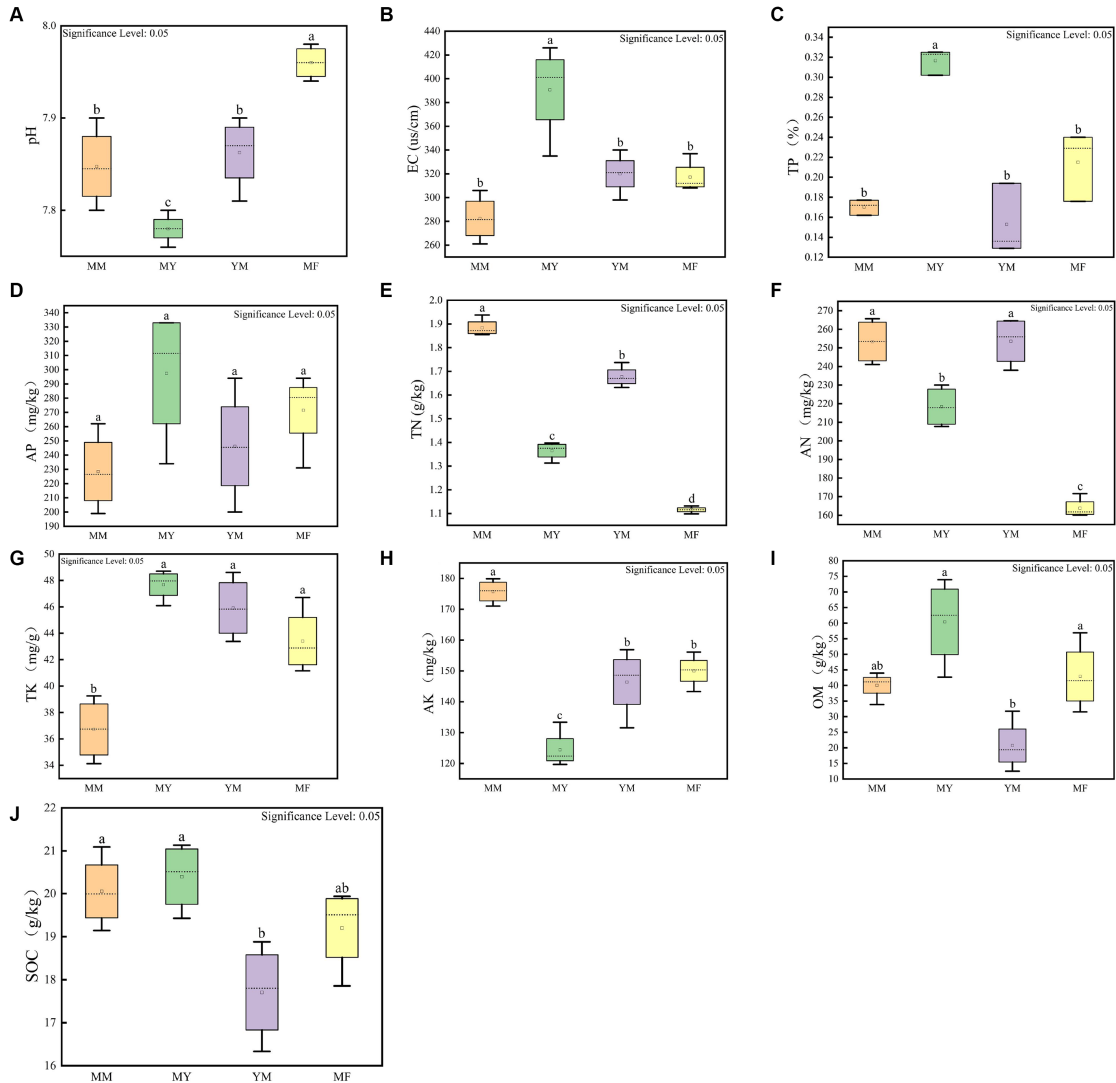
Soil physical and chemical factors measurement results. **(A)** pH box diagram. **(B)** Electric conductivity box diagram. **(C)** Total phosphorus box diagram. **(D)** Effective phosphorus box diagram. **(E)** Total nitrogen box diagram. **(F)** Alkaline nitrogen box diagram. **(G)** Total potassium box diagram. **(H)** Fast potassium box diagram. **(I)** Organic matter content box diagram. **(J)** Organic carbon box diagram. The small letters above represent significant differences (*p* < 0.05).

Figure 2 shows the results of 10 soil physical and chemical factors in the four planting patterns, and the soil physical and chemical factors varied greatly among the different planting patterns. The soil physical and chemical factors that had a higher content in the MM mode compared with the other three rotation modes were TN, AN, and AK, while the soil physical and chemical factors, such as TP, AP, and TK, had lower contents in the MM mode (Figure 2). The MY model had higher levels of several soil physical and chemical factors than the other three cropping models, including the EC, TP, AP, TK, OM, and SOC (Figure 2). The levels of OM and SOC were lower in the YM model than in the other three models, and it is hypothesized that this may be related to the long-term continuous cropping of maize (Figure 2). The MF model had the highest soil pH and the lowest content of soil N presumably because the cultivation of tomato can increase the soil pH (Figure 2).

### 3.2 Soil aggregate structure

**Figure 3:**
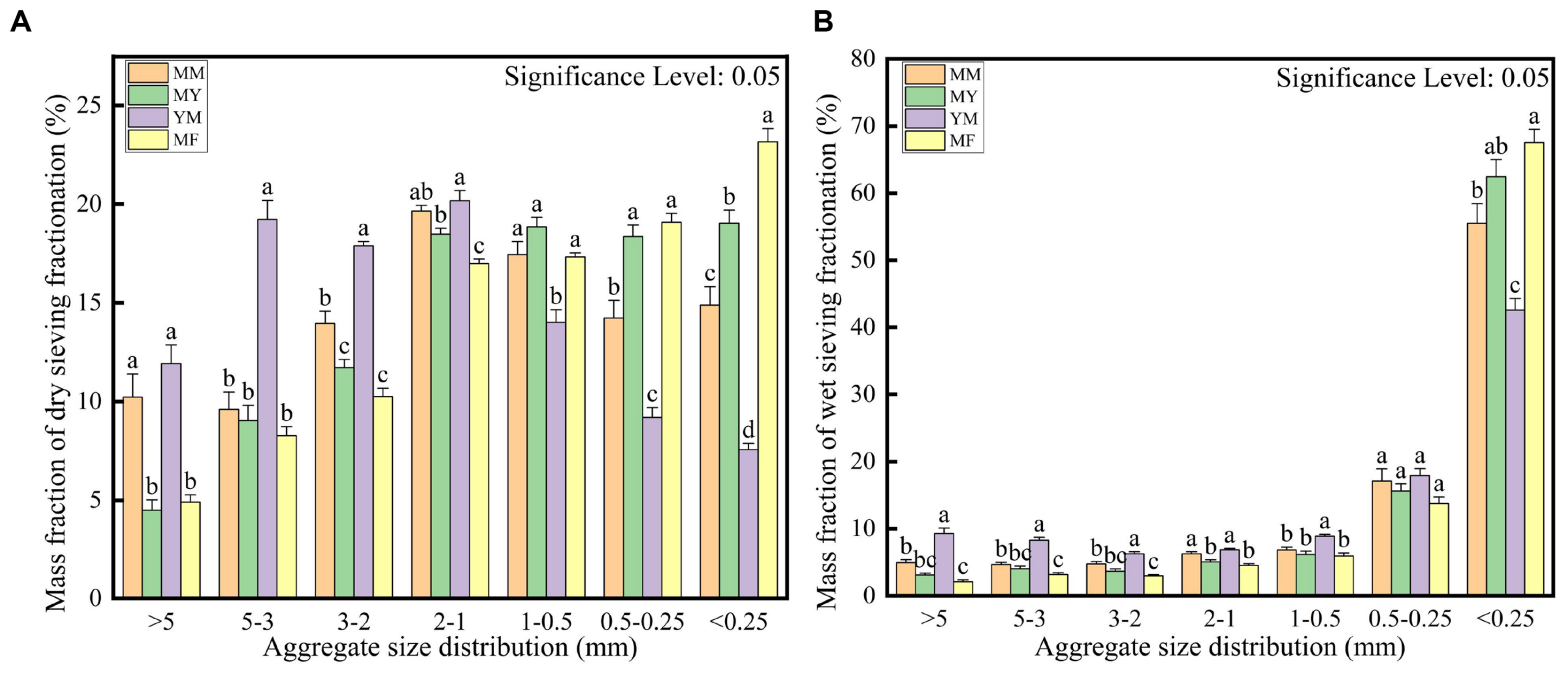
Calculated mass fraction of soil aggregate structure. **(A)** Dry sieving. **(B)** Wet sieving.

The soil aggregate structure was divided into large macroaggregates (>2 mm), small macroaggregates (2–0.25 mm), and microaggregates (<0.25 mm; Yan et al., 2022). It is apparent from the dry sieve assay in Figure 3A that the proportion of particle size of each agglomerate varied for different patterns of cultivation. The YM mode had the highest mass fraction of large macroaggregates; the MM and MY modes focused on small macroaggregates, and the MF mode had the highest mass fraction of microaggregates compared with the other modes, although it had the largest mass fraction of the small clusters. As shown in Figure 3B, the four modes of microaggregates had the highest percentage of the aggregates when the wet sieve assay was used, which may be owing to the excessive moisture that breaks the weak hydrogen bonding between the large macroaggregates (Yan et al., 2022). However, it also revealed the same differences in other particle size mass fractions as in the dry sieve assay.

### 3.3 Soil microbial diversity high-throughput sequencing data analysis

#### 3.3.1 Bacterial diversity sequencing

**Figure 4:**
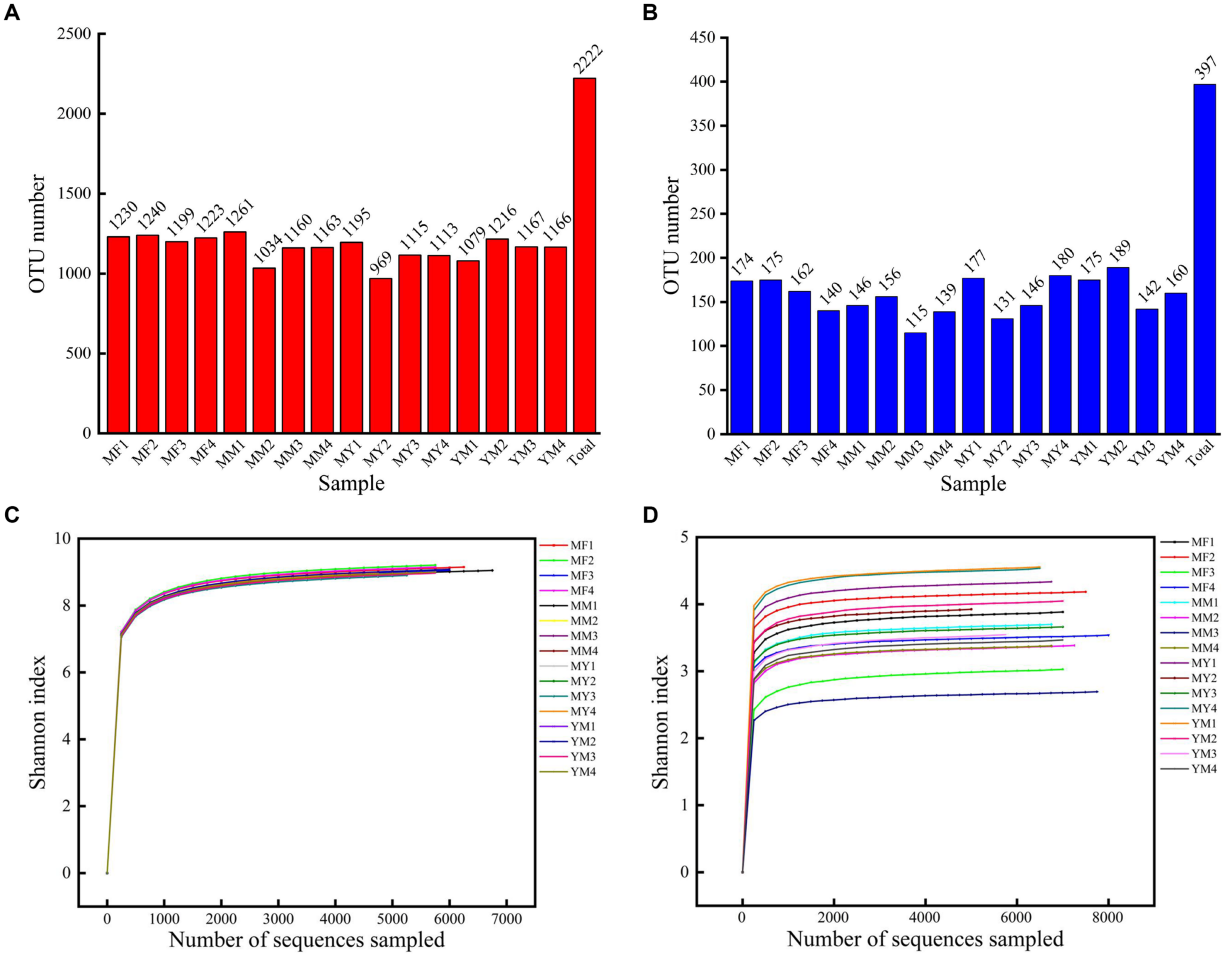
**(A)** Bar chart of bacterial OTU quantity. **(B)** Bar chart of fungus OTU quantity. **(C)** Shannon dilution curve for bacterial sequencing. **(D)** Shannon dilution curve for fungus sequencing.

A total of 119,820 CCS sequences were obtained after 16 samples were sequenced and identified by a barcode. Each sample had at least 6,828 CCS sequences, which yielded an average of 7,489 CCS sequences. The OTUs were obtained by clustering the reads at a 97.0% similarity level using USEARCH, and 2,222 OTUs were obtained from 16 samples. Among them, there were 1,259 OTUs for the four planting modes (Figure 4A).

#### 3.3.2 Sequencing fungal diversity

A total of 121,337 CCS sequences were obtained from 16 samples, and 397 OTUs were obtained by clustering the reads at the 97.0% similarity level using USEARCH. There were 149 OTUs in total for the four cropping patterns (Figure 4B).

Figures 4C,D show that the Shannon dilution curve reached saturation, which is when the sequencing depth continues to increase. The diversity of microorganisms also no longer increased at greater sequencing depths.

### 3.4 Soil microbial alpha-diversity analysis

**Figure 5:**
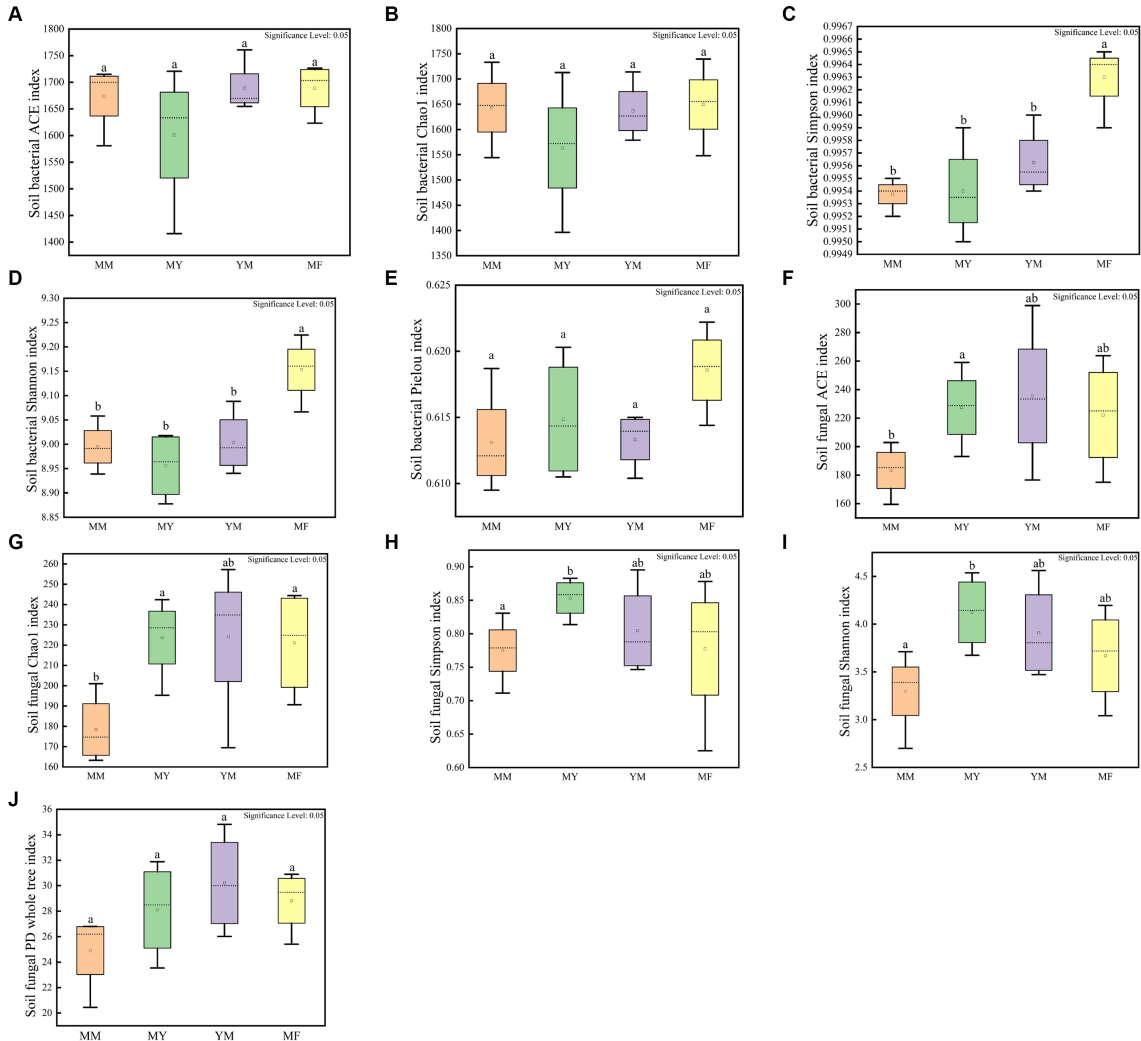
Soil microbial alpha diversity box plot. **(A)** Soil bacterial community ACE index box plot. **(B)** Soil bacterial community Chao 1 index box plot. **(C)** Soil bacterial community Simpson index box plot. **(D)** Soil bacterial community Shannon index box plot. **(E)** Soil bacterial community Pielou index box plot. **(F)** Soil fungal community ACE index box plot. **(G)** Soil fungal community Chao 1 index box plot. **(H)** Soil fungal community Simpson index box plot. **(I)** Soil fungal community Shannon index box plot. **(J)** Soil fungal community PD whole tree index box plot. Different small letters represent significant differences.

The cultivation of aftercrop had no significant effect on the abundance of soil bacterial communities as shown in Figure 5, while different aftercrop had different effects on soil bacterial community diversity. The Simpson and Shannon indices showed that the MF model had significantly greater diversity of the soil bacterial community in the MF model than in the remaining three models (Figures 5A–E), which indicated that the soil microecology community in the MF model was highly diverse.

In the soil fungal community, the MM model was lower than that of the other three models in both the mean richness and the mean-diversity indices, and the alpha-diversity of the MM model was significantly different from that of the MY model. It is apparent that long-term continuous cropping reduced the diversity, as well as the abundance, of the soil fungal community (Figures 5F–J).

### 3.5 Analysis of the microbial beta-diversity in the soil microenvironment

**Figure 6:**
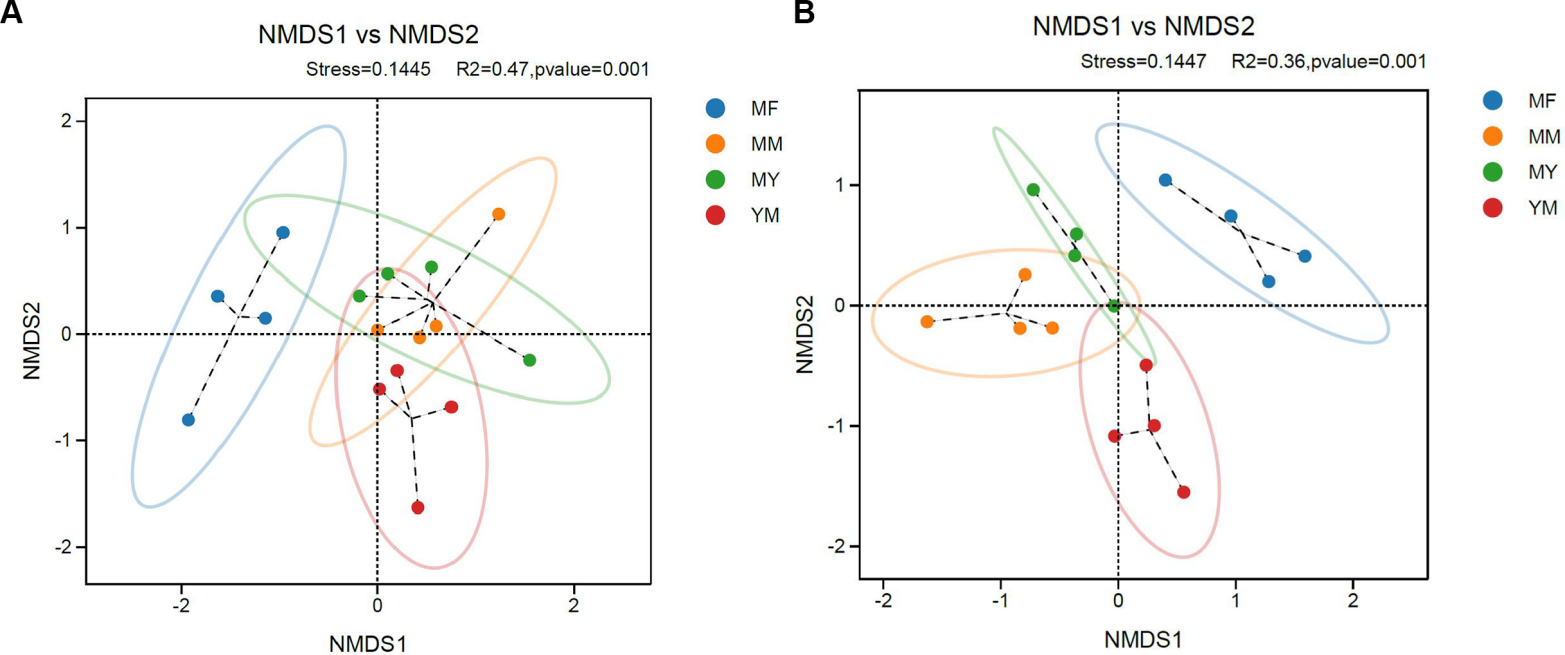
Coordinate diagram of NMDS analysis. **(A)** Bacteria. **(B)** Fungi.

The stress values in the NMDS analysis were 0.1445 and 0.1447 in bacteria and fungi, respectively, which <0.2, indicating that the NMDS analysis was informative. As shown in Figure 6, the NMDS analysis showed that both the fungi and bacteria in the MM, MY, and YM models of the soil microecology community composition were relatively close, while there was a greater difference in the MF model between the soil fungal community composition and the other three models of the composition of the difference (Figure 6B). The soil bacterial community in the MF model was closer in composition with the MY model and differed from the other two models in the soil bacterial community composition (Figure 6A). It is apparent that the cultivation of tomato significantly changed the soil fungal community of long-term cotton succession compared with corn (Figure 6B).

### 3.6 Analysis of the relative abundance of the soil microecological microbial community

A Naive Bayes classifier was used in combination with a comparison approach, and the feature sequences were annotated with SILVA as the reference database. The taxonomic information of the species that corresponded to each feature can be obtained, which, in turn, counts the community composition of each sample at each level. Two kingdoms, 28 phyla, 62 orders, 165 orders, 271 families, 490 genera, and 746 species were obtained by sequencing the bacterial diversity. Ten phyla, 22 orders, 52 orders, 883 families, 146 genera, and 209 species were obtained from sequencing the fungal diversity.

**Figure 7:**
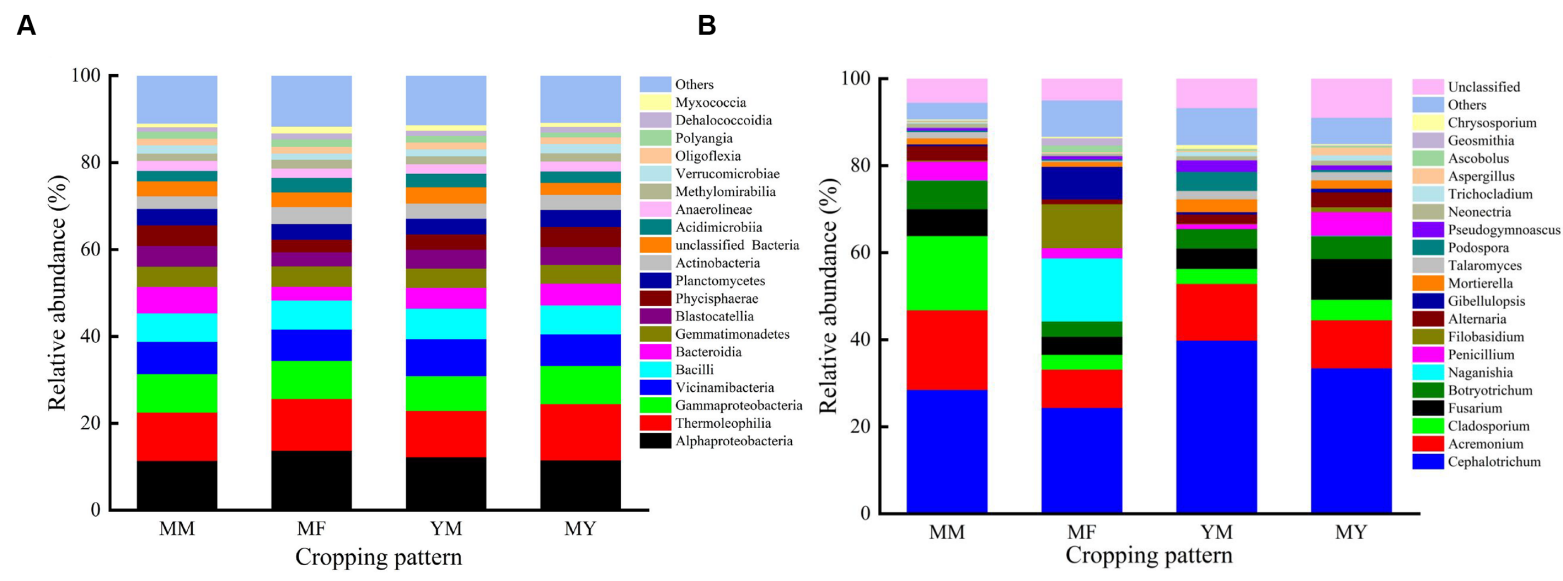
Relative abundance of the most common bacterial and fungus. **(A)** Bacterial. **(B)** Fungus.

At the class level, the dominant bacterial communities with higher relative abundance varied less among the different cropping patterns, which may be an effect owing to the long duration of cotton cultivation. The primary dominant bacterial communities were as follows: Gaiellales, Vicinamibacterales, Burkholderiales, Bacillales, Rhizobiales, Gemmatimonadales, Solirubrobacterales, Tepidisphaerales, and Pyrinomonadales etc. Figure 7A shows that Alphaproteobacteria had the highest relative abundance in the MM, MF, and YM patterns. The relative abundance was 11.32%, 13.65%, and 12.14%, respectively. In contrast, the soil bacterial community with the highest relative abundance in the MY model was Thermoleophilia with a relative abundance share of 12.97%.

At the genus level, the dominant flora in the soil fungal community were as follows: *Cephalotrichum*, *Acremonium*, *Cladosporium*, *Fusarium*, *Botryotrichum*, *Naganishia*, *Penicillium*, *Filobasidium*, *Alternaria*, and *Gibellulopsis* etc. As shown in Figure 7B, the relative abundance of *Cephaalotrichum* was the highest among the four cropping patterns. The relative abundance of *Cladosporium* was significantly reduced in the rotational cropping pattern compared with that of the continuous cropping pattern. It was also found that the relative abundance of fungal communities varied considerably among the different cropping patterns. Thus, it was deduced that Cultivation of aftercrop had a greater effect on the soil bacterial community than on the fungal community.

### 3.7 LEfSe analysis

**Figure 8:**
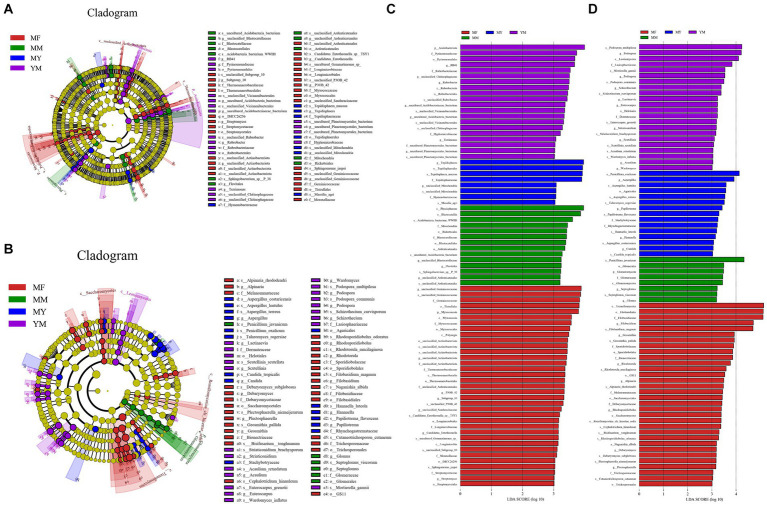
LEfSe analysis. **(A)** Branching diagram of bacterial LEfSe analysis; **(B)** Branching diagram of fungal LEfSe analysis; **(C)** Histogram of bacterial LEfSe analysis. **(D)** histogram of fungal LEfSe analysis.

The LEfSe analysis of the bacterial high-throughput sequencing data (Figures 8A,C) showed that there were as many as 35 biomarker communities in the MF rotation pattern, and the ones that played an important role were as follows: Geminicoccaceae, Myxococcota, and Tistrellales etc. There were 20 biomarkers in the YM model (Figures 8A,C), and the primary ones that played an important role were as follows: Acidobacteriota, Pyrinomonadaceae, Pyrinomonadales, and *RB41*. There were 14 biomarkers in the MM model (Figures 8A,C), and the primary ones that played an important role were as follows: Phycisphaerae, Blastocatellia, *WWH8*, and Rickettsiales among others. In contrast, there were fewer biomarkers in the MY rotation pattern soil bacterial biomarker community with only eight species (Figures 8A,C). The primary ones that played an important role were as follows: *Tepidisphaera* and Tepidisphaerales among others. Biomarker communities relative abundance histogram were uploaded to Supplementary Figure 1.

In the LEfSe analysis of the data from the fungal high-throughput sequencing (Figures 8B,D), the MF model was still found to have the highest number of biomarker communities with 32 fungal communities. The primary ones that played an important role were as follows: Tremellomycetes, Filobasidiacceae, and Filobasidiales among others. There were 22 biomarkers in the YM model (Figures 8B,D), and the primary ones that played an important role were as follows: *Podospora multipilosa*, *Podospora*, and Leotiomycetes among others. There were 18 biomarkers in the MY model (Figures 8B,D), and the primary ones that played an important role were as follows: *Penicillium oxalicum*, *Aspergillus*, and *Aspergillus lentulus* among others. With the least number of biomarkers in the MM model (Figures 8B,D), there were only eight biomarkers, and the primary biomarkers with a high impact were as follows: *Penicillium javanicum* and Glomeromycota among others. Biomarker communities relative abundance histogram were uploaded to Supplementary Figure 2.

### 3.8 RDA of the composition of the microbial community and soil physical and chemical properties in the soil microenvironment

**Figure 9:**
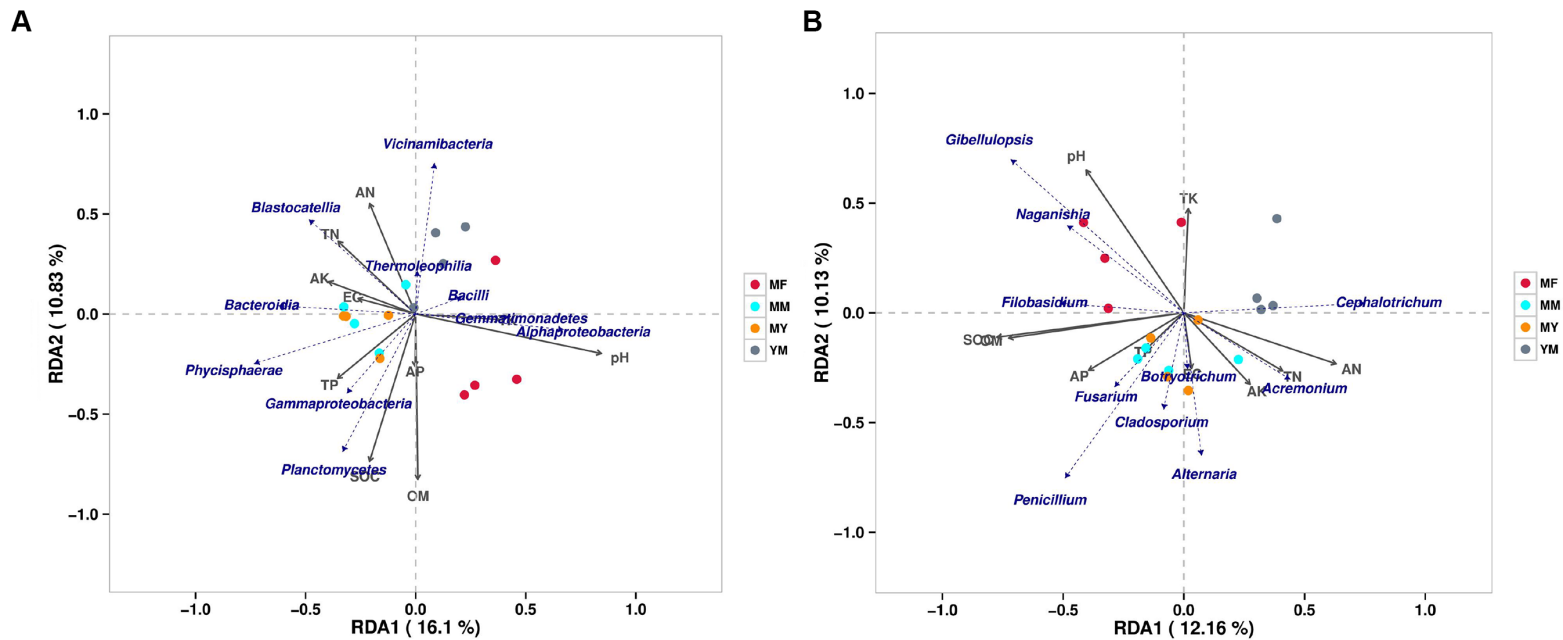
RDA analysis of soil bacterial and fungal communities. **(A)** Bacterial RDA analysis. **(B)** fungal RDA analysis.

At the class level, a bacterial RDA showed that the long-term continuous cropping cotton fields planted with different succeeding crops positively correlated with varying soil bacterial communities and soil physical and chemical factors (Figure 9A). In addition, the MM pattern bacterial communities positively correlated with physical and chemical factors, such as TN, AK, and EG, and negatively correlated with the TK and pH. It also positively correlated with the population composition of Blastocatellia, Bacteroidia, and Phycisphaerae etc. The bacterial communities in the MY model positively correlated with the AK, EG, TP, and SOC. Simultaneously, it positively correlated with the population composition of Bacteroidia, Phycisphaerae, Gammaproteobacteria and Planctomycetes. The YM model bacterial communities positively correlated with the TN and AN, while they negatively correlated with the TP, SOC, AP, and OM and positively correlated with the population composition of Vicinamibacteria, Blastocatellia, and Themoleophilia. In contrast, the MF model bacterial community positively correlated with AK and pH and also with the population composition of Bacillus, Gammaproteobacteria, and Alphaproteobacteria.

At the genus level, the fungal RDA (Figure 9B) showed that the patterns of the plants of different succeeding crops have varying effects on the soil physical and chemical factors and the microbial community. The MM model fungal communities positively correlated with TP and EC, while they negatively correlated with the pH and TK. They positively correlated with the *Penicillium*, *Alternaria*, *Botryotrichum*, and *Cladosporium* communities. In addition, as shown in Figure 9B, there was a large overlap between the MY and the MM models. The YM model fungal community negatively correlated with the SOM, OM, and AP and positively correlated with the community *Cephalotrichum*. The MF model fungal community positively correlated with the pH, whereas it negatively correlated with the EC, AK, TN, and AN and positively correlated with the communities *Gibellulopsis*, *Naganishia*, and *Filobasidium*.

### 3.9 Analysis of the correlation between the structure of soil aggregates and the soil physical and chemical properties

There is a strong correlation between the soil aggregate structure and physical and chemical factors, and as shown in previous studies, it is possible to influence the soil aggregates by regulating carbon (C) and N in the soil (Mustafa et al., 2020). Different cropping patterns resulted in varying soil physicochemical factors and therefore, differences in the soil aggregate mass fraction between patterns. To further analyze the relationship between the aggregates and soil physical and chemical factors, SPSS 25.0 was used to conduct a Spearman’s correlation coefficient analysis between the MWD index of aggregates and the physical and chemical factors. As shown in Table 1, the soil aggregates are closely related to the contents of soil N, P and organic matter. These results suggest that there is a close relationship between the soil microorganisms, aggregate structure, and soil physical and chemical factors.

**Table 1:** Correlation analysis between soil physicochemical factors and MWD index.

	pH	EC	TP	AP	TN	AN	TK	AK	SOC	OM
MWD	−0.095	−0.410	−0.638^⁎⁎^	−0.364	0.629^⁎⁎^	0.687^⁎⁎^	−0.114	0.244	−0.440	−0.624^⁎⁎^

^**^The correlation is significant at the 0.01 level.^*^Significant correlation at 0.05 level.

## 4 Discussion

The soil physicochemical properties, an important component of soil health, affect moisture, aeration, soil temperature, nutrient cycling, and root growth, which, in turn, affects crop yields and environmental quality (Zhou et al., 2014; Sainju et al., 2022; Zhao and Nian, 2023). Crop rotations can have an impact on the soil physicochemical properties (Sandhu et al., 2017), and long-term continuous cropping patterns can lead to imbalances in the soil nutrients (Munishi et al., 2021). In this study, an analysis of the four planting patterns revealed that the soil nutrients of the MM continuous cropping model were not balanced, and most of the contents of the soil nutrients were at maximal or minimal levels. This resulted in a nutrient imbalance in the soil microenvironment. However, there were relatively high contents of EC, OM and SOC in the MY model, and it was hypothesized that this model has more soil microorganisms compared with the other three models. It was apparent that using a subsequent crop of maize in a cotton continuous cropping field alleviated the imbalance in soil nutrients. Because the YM and MM models are different planting patterns of previous crops, there are some differences in the physical and chemical properties of the soil. This shows that the physical and chemical properties of the farmland soil are closely related to the cultivation of previous crops. It was also found that different cropping patterns diversify the soil nutrient structure, which will help to improve the soil.

Soil aggregates are the basic units of soil and have a crucial influence on the microbial abundance and diversity and even on certain functional taxa (Xiong et al., 2022). Different tillage practices can affect the physical and chemical properties of the soil, which can, in turn, affect the soil aggregate structure (Zheng et al., 2018). Figure 2 shows that the varying rotation patterns have different mass fraction sizes at each grain level, presumably owing to the differences in cotton corn stover. This led to differences in the soil physicochemical properties, and thus, differences in the grain structure of the agglomerates. In addition, to study the effect of crop rotation patterns on soil aggregate stability, the soil aggregate MWD index was calculated (Pirmoradian et al., 2005). The results showed that the YM mode had the highest soil aggregate stability, while the MY and MF rotation modes were less stable. Thus, the continuous cropping cotton field has an unstable effect on the stability of soil aggregates after different succeeding crops are planted.

Some studies have shown that intensive cultivation based on monoculture has a significant impact on ecosystem functioning (Woo et al., 2022), and that crop rotation can contribute to an increase in farmland biodiversity and also serve as a way to mitigate the prevalence of soil diseases (Gong and Wu, 2021; Zhou et al., 2023). Root secretions and crop residues determine the diversity and composition of the soil microbial communities (Fierer, 2017). In addition, rhizosphere microbes are recruited from the soil (Wippel et al., 2021). Thus, the rhizosphere microbial community is strongly influenced by the soil microbial community, which demonstrates that the soil microbes are critical to the health of crops. In this study, the soil microecology communities of the four cropping patterns were analyzed using high-throughput sequencing, and there was no significant difference in the richness indices of the soil bacterial communities of the four cropping patterns, while the bacterial diversity index of the MF pattern was significantly higher than that of the other three patterns. In contrast, there were differences in the presence of both the richness and diversity indices in the fungal community. Thus, it is apparent that the soil fungal community is more easily affected by the planting of succeeding crops. In addition, NMDS analyses were performed to explore the differences in microbial communities among the four models. They showed that the soil microbial communities of the MF model differed from those of the other three models. It was deduced that the planting of maize changed the microbial community of the soil in the long-term continuous cropping of cotton less than that of tomato, which may be related to differences in the root secretions of maize and tomato. Therefore, the MF planting model can be used to improve the imbalance in the microecological community caused by the long-term rotation of cotton.

The soil microbiome is a complex mixture that is primarily composed of fungi and bacteria (Silva-Filho and Vivanco, 2017) and plant health. Productivity and nutrient cycling are strongly driven by soil microbes (Beule et al., 2022), and there are complex links between the two. The primary dominant bacterial taxon in the MM, MF, and YM models was Alphaproteobacteria, which is closely associated with all complex life forms and exists in different lifestyles (Muñoz-Gómez et al., 2019). It was hypothesized that this is why these microbes are the dominant flora. While in the MY model, Thermoleophilia became the dominant bacterial group, which has an important role in geochemical cycling (Hu et al., 2019). However, the relative abundance of the dominant bacterial flora of the four cropping patterns did not differ much overall, which may be related to the long-term planting of cotton and use of the same amount of fertilizer, thus, making it the core population in soil microecology. The soils contain highly diverse fungal communities that play a key role in the decomposition of organic matter and nutrient cycling (Sutela et al., 2019). *Cephaalotrichum* was the dominant fungal community in all four cropping patterns, and most of the fungi were found in decaying plants, manure, and soil (Woudenberg et al., 2017). Therefore, it was deduced that the dominance of *Cephaalotrichum* was related to the annual return of straw to the farmland. The relative abundance of *Cladosporium* was found to be higher in the cotton continuous cropping pattern than in the other three patterns. These microbes may be used as a secondary disease invasion, and some species can act as pathogens (Benitez et al., 2017). It is apparent that the results of long-term continuous cropping can lead to an increase in pathogenic bacteria. Previous studies have shown that different patterns of crop rotation had varying effects on the fungal and bacterial communities (Xi et al., 2021), and the dominant fungal communities were shown to vary considerably among the four patterns. Thus, deductions on this finding confirmed the previous conclusions that the soil fungal communities were more affected by the current year’s crop.

To better visualize the differences in soil microbial communities among different planting modes, an LEfSe analysis was performed that revealed many biomarkers between the soil microecology community of the four cropping patterns. Both the fungal and bacterial communities in the MF rotation model soil microecology biomarker were the most abundant. It could also be deduced that planting a succeeding crop of tomato has a greater effect on the composition of soil microbial community in long-term continuous cropping of cotton than when the succeeding crop is maize.

Soil microorganisms have a major role in the environment in nutrient cycling, C storage, and soil remediation (Burges et al., 2018; Kumar et al., 2022), thus, the composition of soil nutrients affects the composition of microbial communities (Wang et al., 2017). The linkage between soil physicochemical properties and the soil microecology community was analyzed by an RDA, and it was shown that the microbial communities of different crop rotation patterns will be associated with varying physical and chemical factors. Thus, it is hypothesized that the reason for this is that different crops have varying impacts on the soil nutrients, and different cropping patterns also result in varying soil microecology communities. In conclusion, there is a close relationship between soil microbial communities, physicochemical properties, and aggregate structure.

## 5 Summary

In this study, the composition, abundance, and diversity of the soil microecology community were analyzed in depth by applying high-throughput sequencing to four planting patterns. The soil physicochemical properties, as well as the aggregate structure, of the four cropping patterns were also examined. It was concluded that the soil physicochemical properties are related to different cropping patterns, and that the same crop will have different soil physicochemical properties depending on the cropping pattern in which it is grown; The rotation of various succeeding crops has different effects on the stability of aggregates, and rotation may not necessarily improve the stability of aggregates; The planting of aftercrop corn and tomato had a greater effect on the soil fungal community than the bacterial community in the cotton continuous cropping farmland, and the relative abundance of the dominant soil fungal community varied considerably among the four cropping patterns. The MF model microbial community is relatively different from the other three models, and cotton crop succession reduced the richness and diversity of soil microorganisms. RDA analysis revealed a close relationship among the soil microbial community soil physicochemical properties and the soil aggregate structure; In addition, it was shown that there are advantages present in all three rotation patterns, with higher EC, SOC, and OM contents in the MY pattern, which can be seen in the richness of soil nutrients, as well as higher soil microbial activity. The YM model soil aggregates are more stable, and the MF cropping patterns altered the microbial community of long-term cotton plantings to a greater extent. In agricultural production, we can combine the actual situation with the advantages of three different succeeding crop rotations to improve the soil microenvironment and promote the green and sustainable development of agriculture.

## Data availability statement

The datasets presented in this study can be found in online repositories. The names of the repository/repositories and accession number(s) can be found at: https://www.ncbi.nlm.nih.gov/, PRJNA1094459 and https://www.ncbi.nlm.nih.gov/, PRJNA1094467.

## Author contributions

SH: Data curation, Formal analysis, Investigation, Methodology, Writing – original draft, Writing – review & editing. XJ: Data curation, Formal analysis, Writing – original draft. LH: Data curation, Methodology, Software, Writing – review & editing. GL: Methodology, Software, Visualization, Writing – review & editing. JY: Methodology, Visualization, Writing – review & editing. AW: Funding acquisition, Project administration, Resources, Supervision, Validation, Writing – review & editing.
