# Supplementary figures and images for: Effects of aftercrop tomato and maize on the soil microenvironment and microbial diversity in a long-term cotton continuous cropping field (part 1 of 2)
Source: Front Microbiol. 2024 Jul 19;15:1410219. doi: 10.3389/fmicb.2024.1410219 (PMC11295657; doi:10.3389/fmicb.2024.1410219)

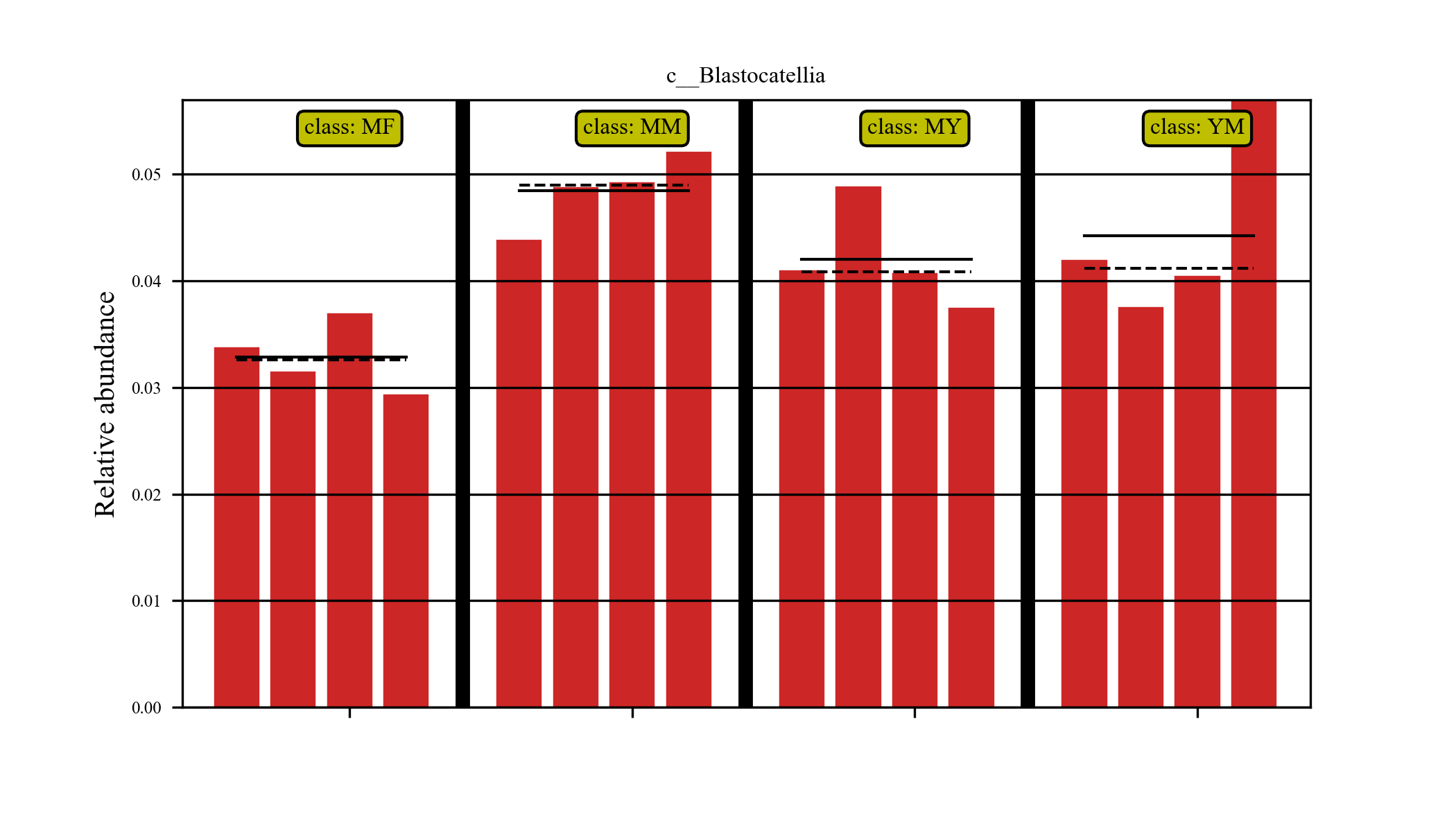

Supplement: Supplementary file 1 [file Data_Sheet_1.ZIP › Supplementary figure 1. bacterial biomarker community/1_c__Blastocatellia.png]

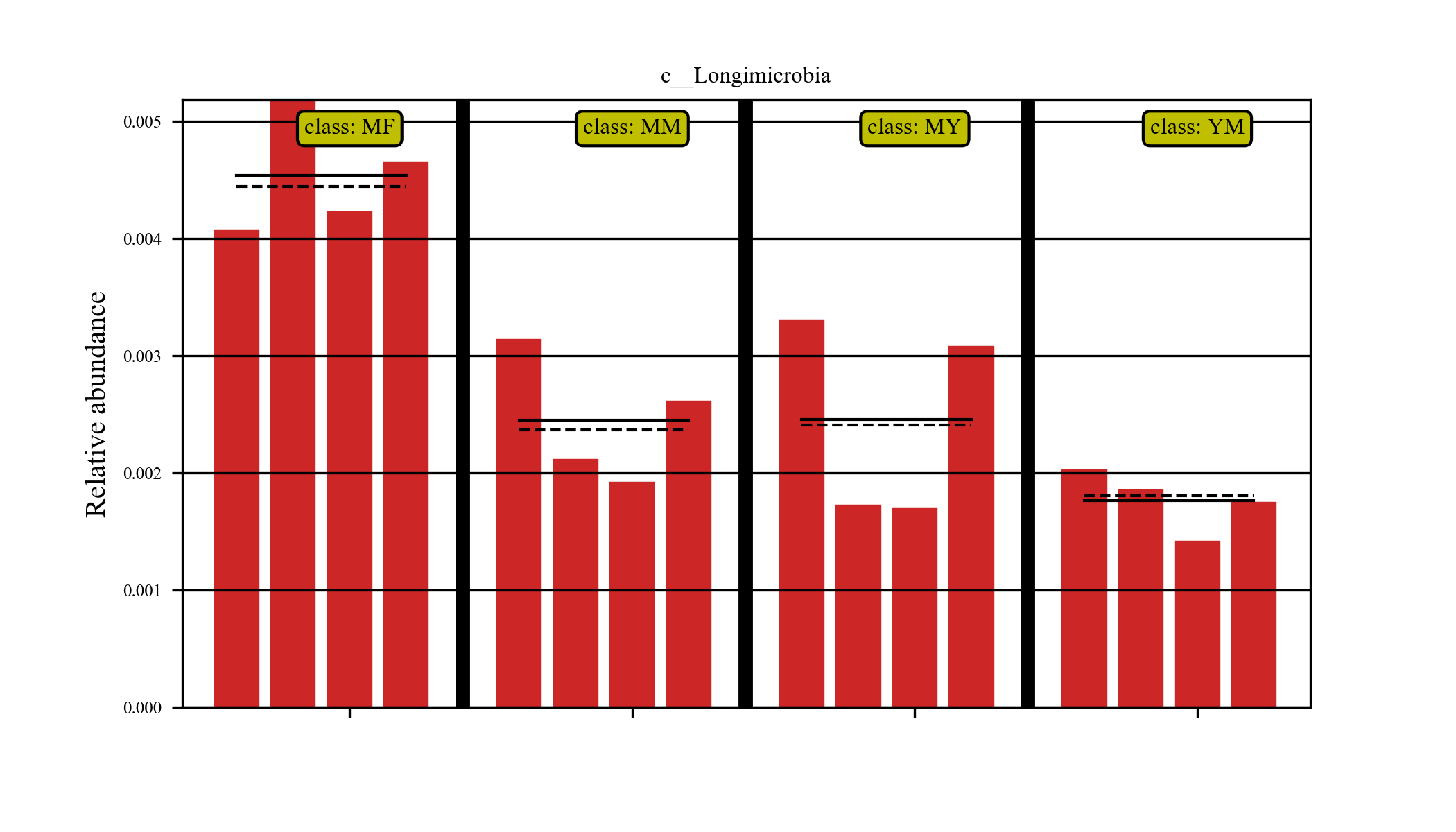

Supplement: Supplementary file 1 [file Data_Sheet_1.ZIP › Supplementary figure 1. bacterial biomarker community/1_c__Longimicrobia.png]

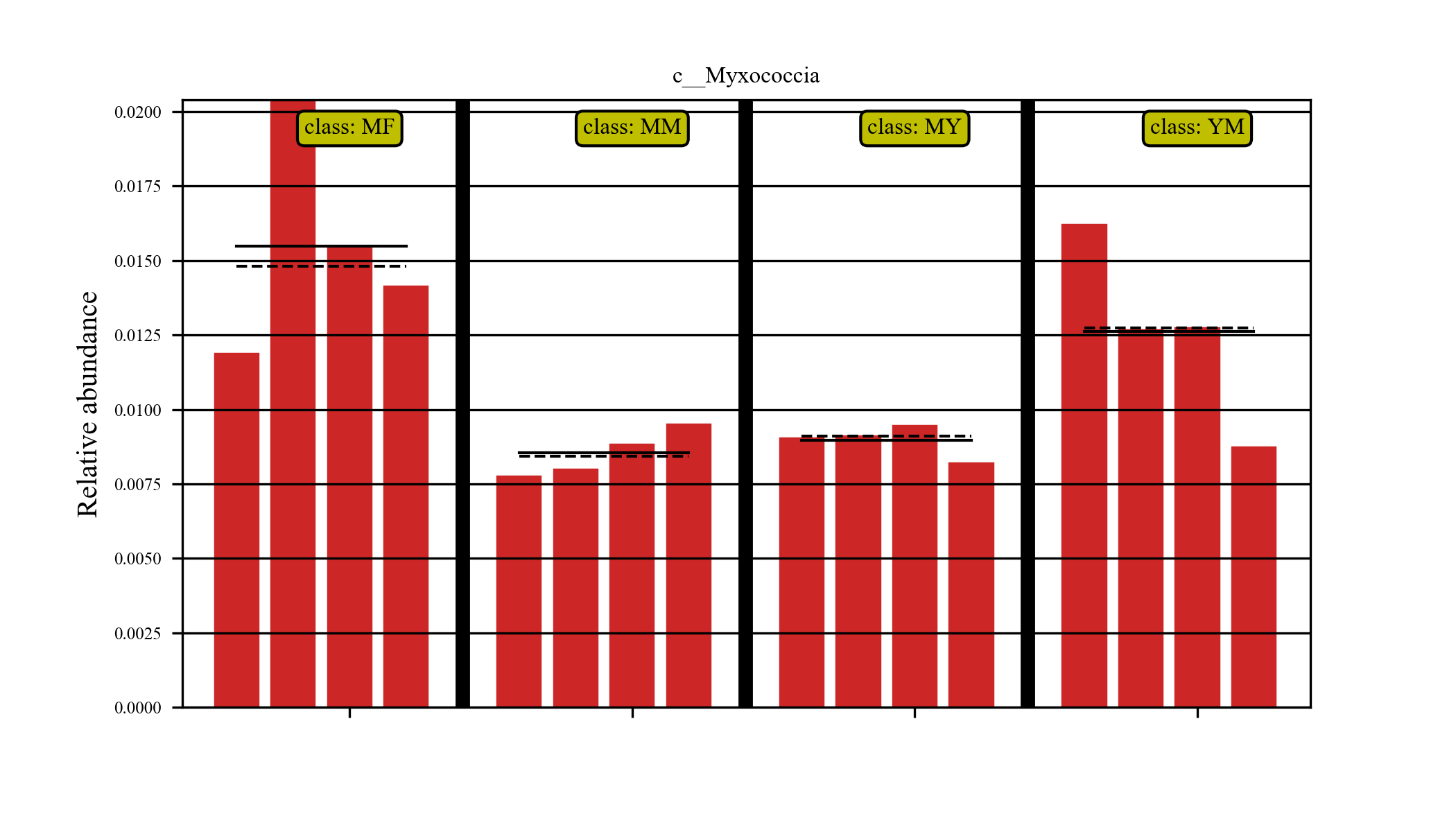

Supplement: Supplementary file 1 [file Data_Sheet_1.ZIP › Supplementary figure 1. bacterial biomarker community/1_c__Myxococcia.png]

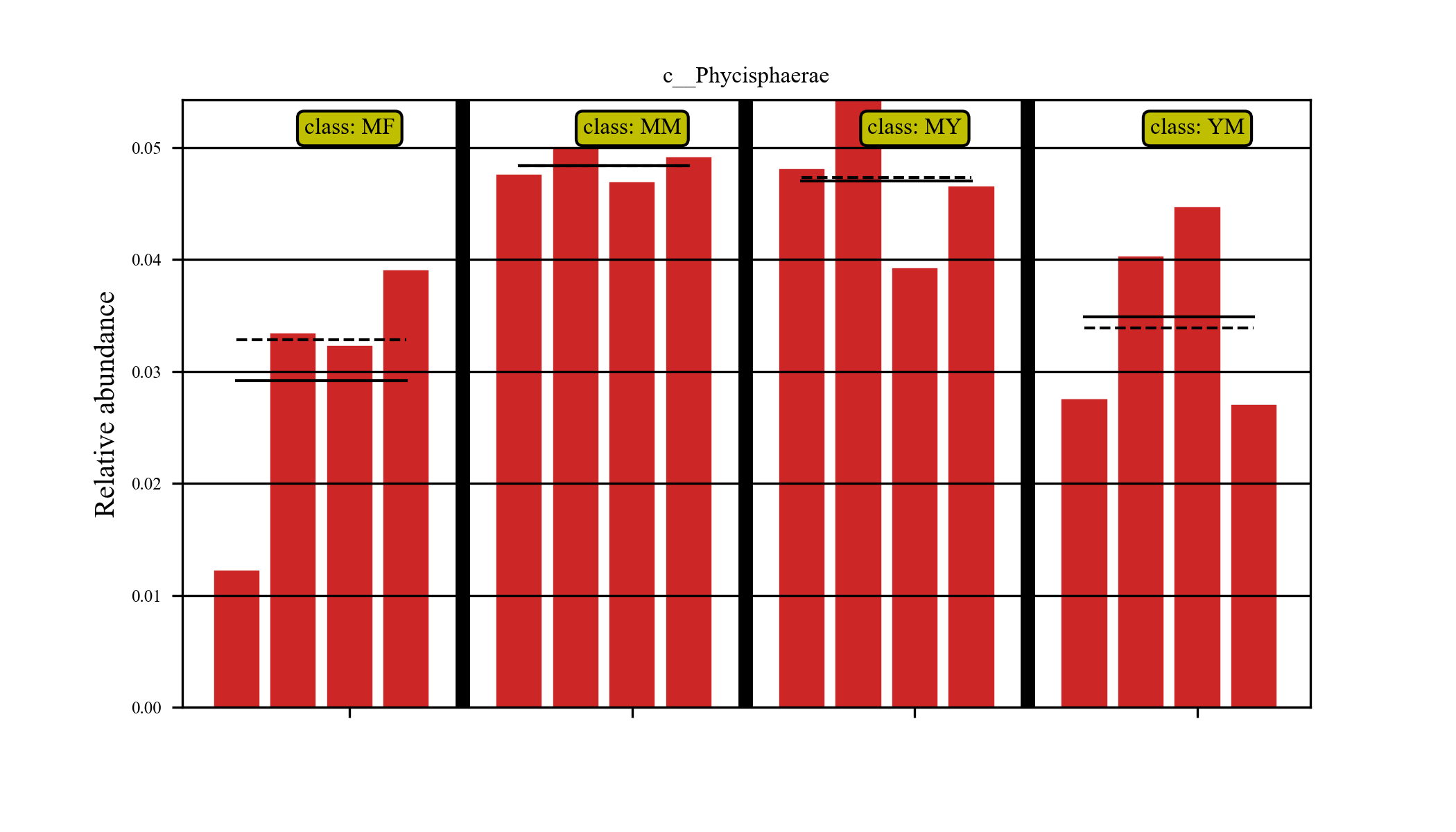

Supplement: Supplementary file 1 [file Data_Sheet_1.ZIP › Supplementary figure 1. bacterial biomarker community/1_c__Phycisphaerae.png]

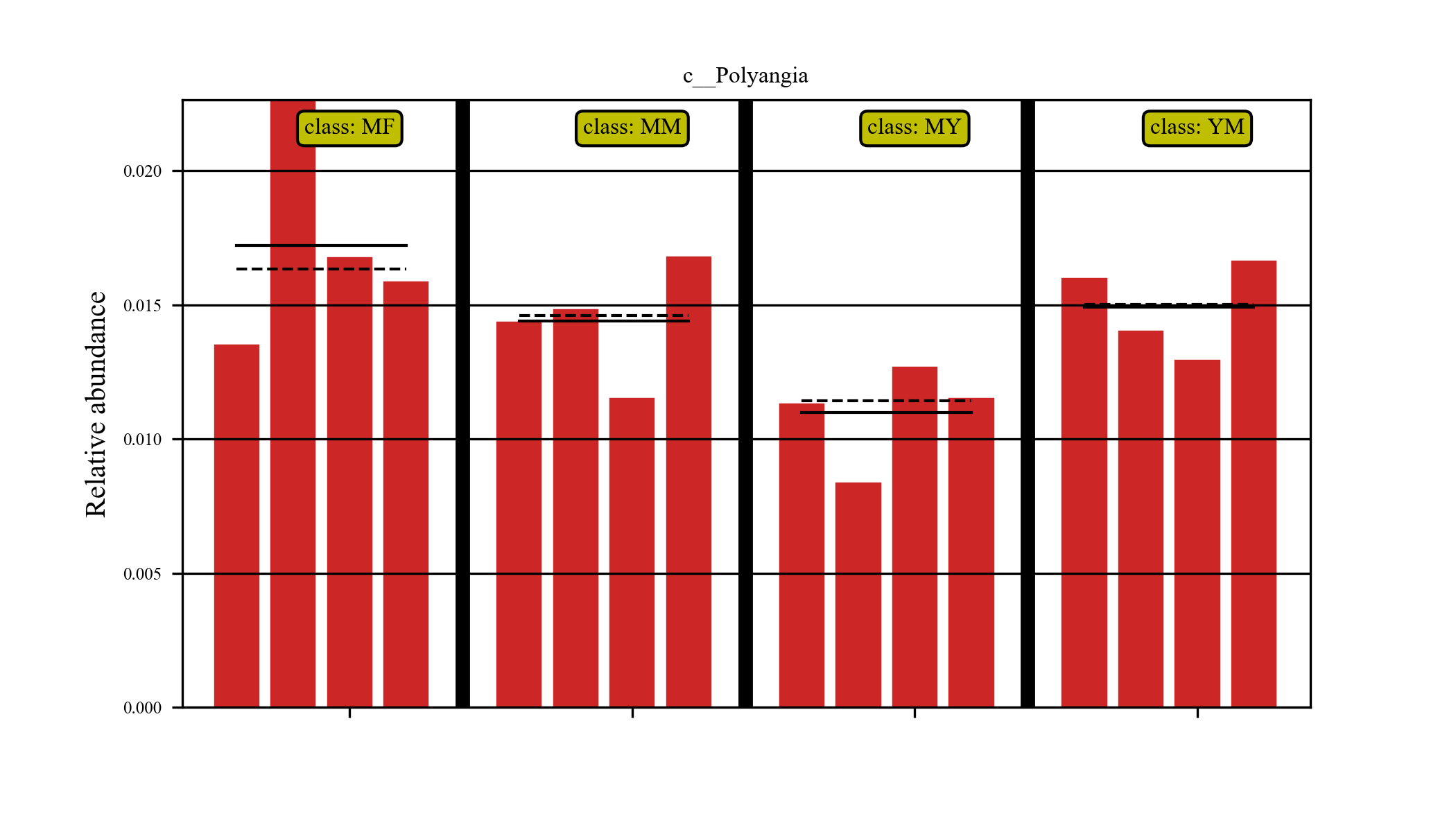

Supplement: Supplementary file 1 [file Data_Sheet_1.ZIP › Supplementary figure 1. bacterial biomarker community/1_c__Polyangia.png]

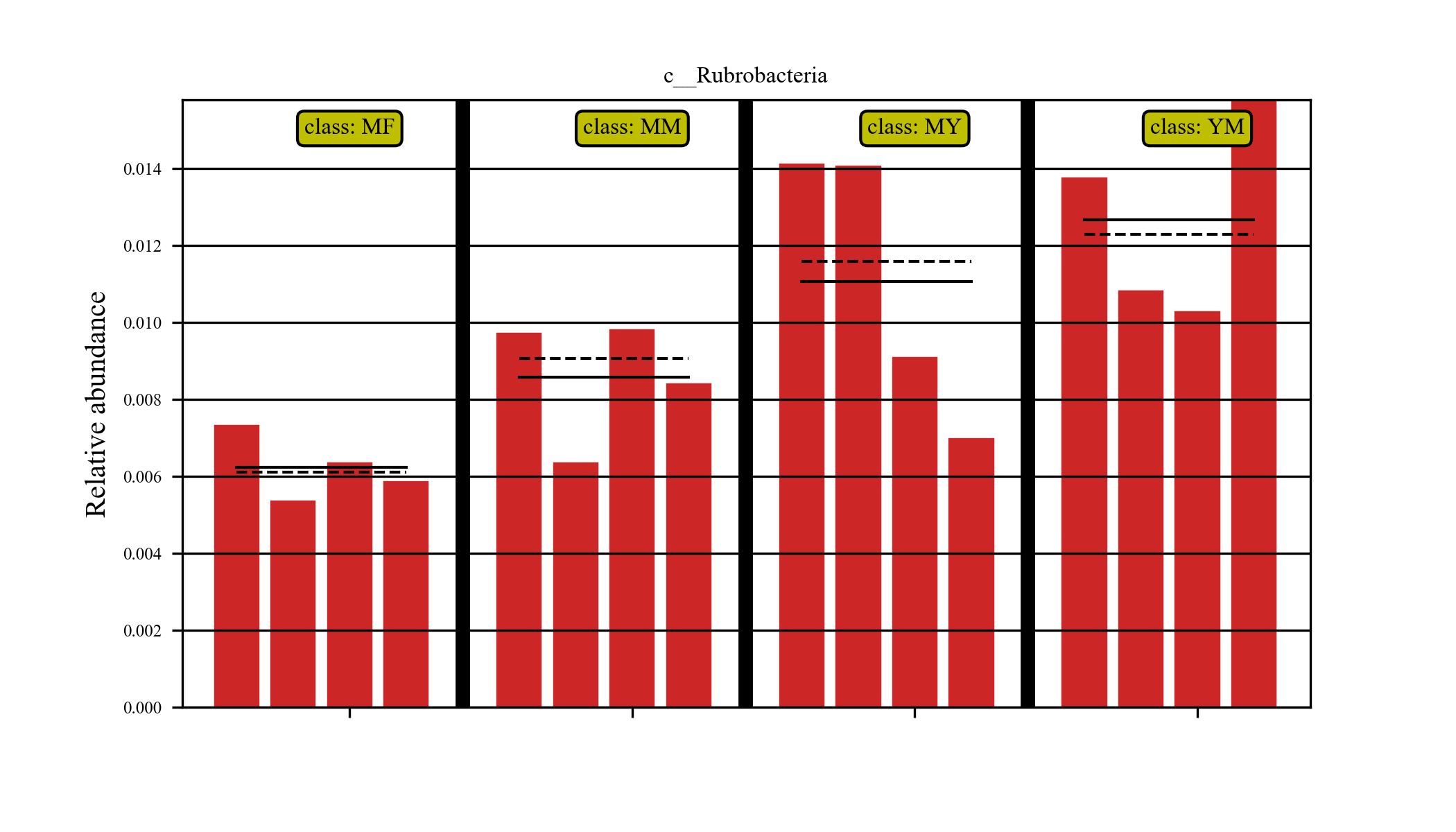

Supplement: Supplementary file 1 [file Data_Sheet_1.ZIP › Supplementary figure 1. bacterial biomarker community/1_c__Rubrobacteria.png]

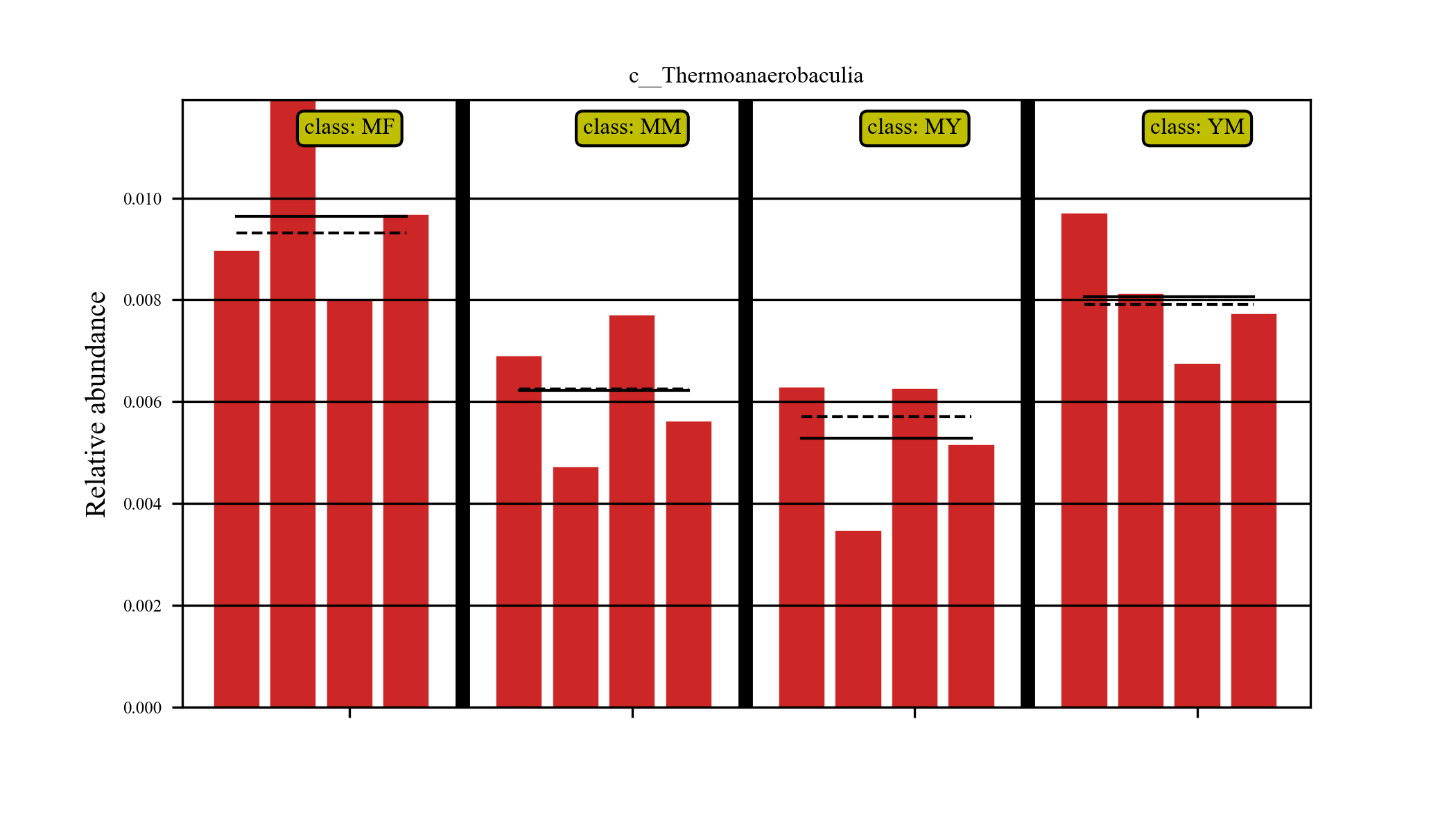

Supplement: Supplementary file 1 [file Data_Sheet_1.ZIP › Supplementary figure 1. bacterial biomarker community/1_c__Thermoanaerobaculia.png]

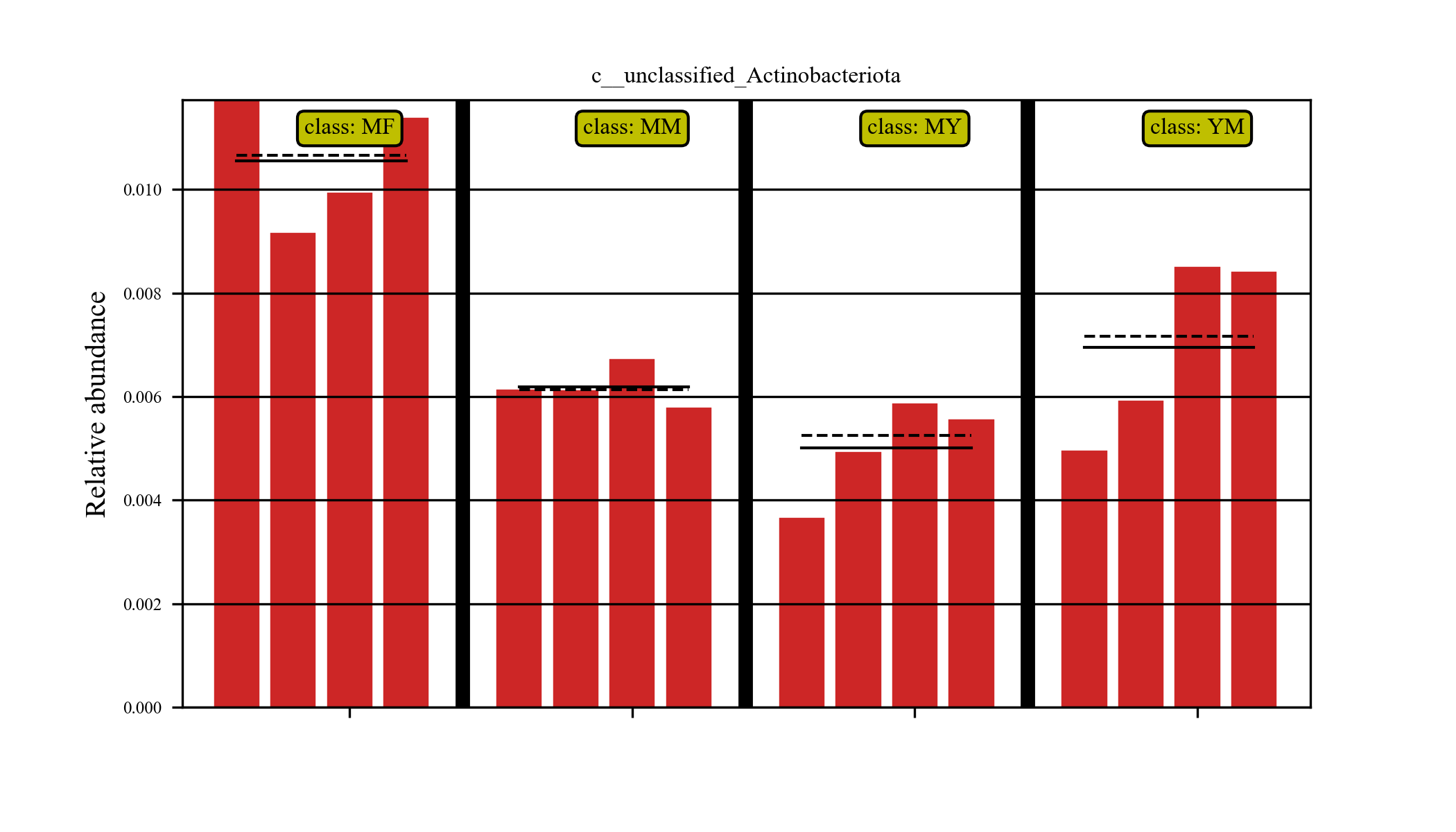

Supplement: Supplementary file 1 [file Data_Sheet_1.ZIP › Supplementary figure 1. bacterial biomarker community/1_c__unclassified_Actinobacteriota.png]

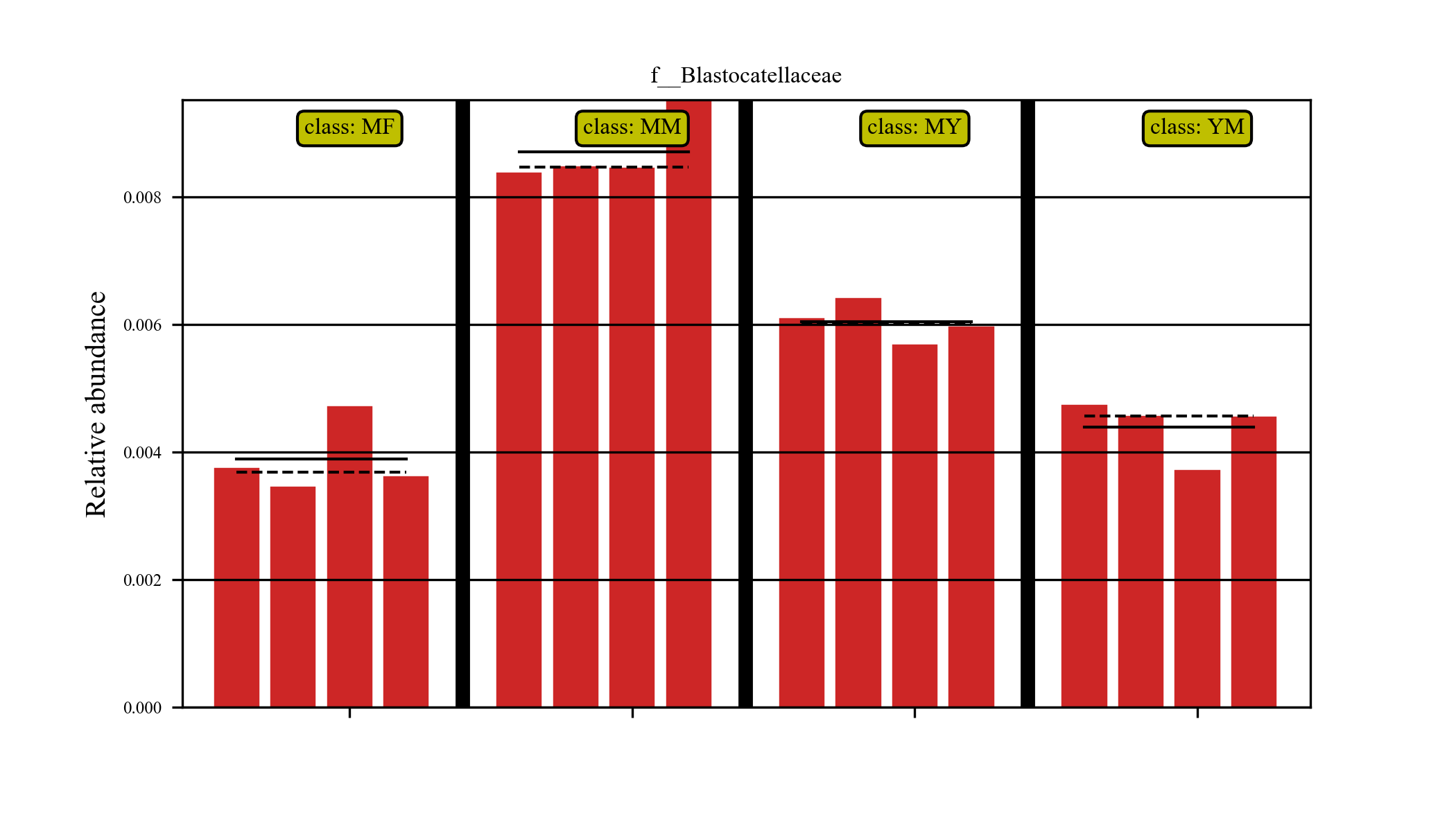

Supplement: Supplementary file 1 [file Data_Sheet_1.ZIP › Supplementary figure 1. bacterial biomarker community/1_f__Blastocatellaceae.png]

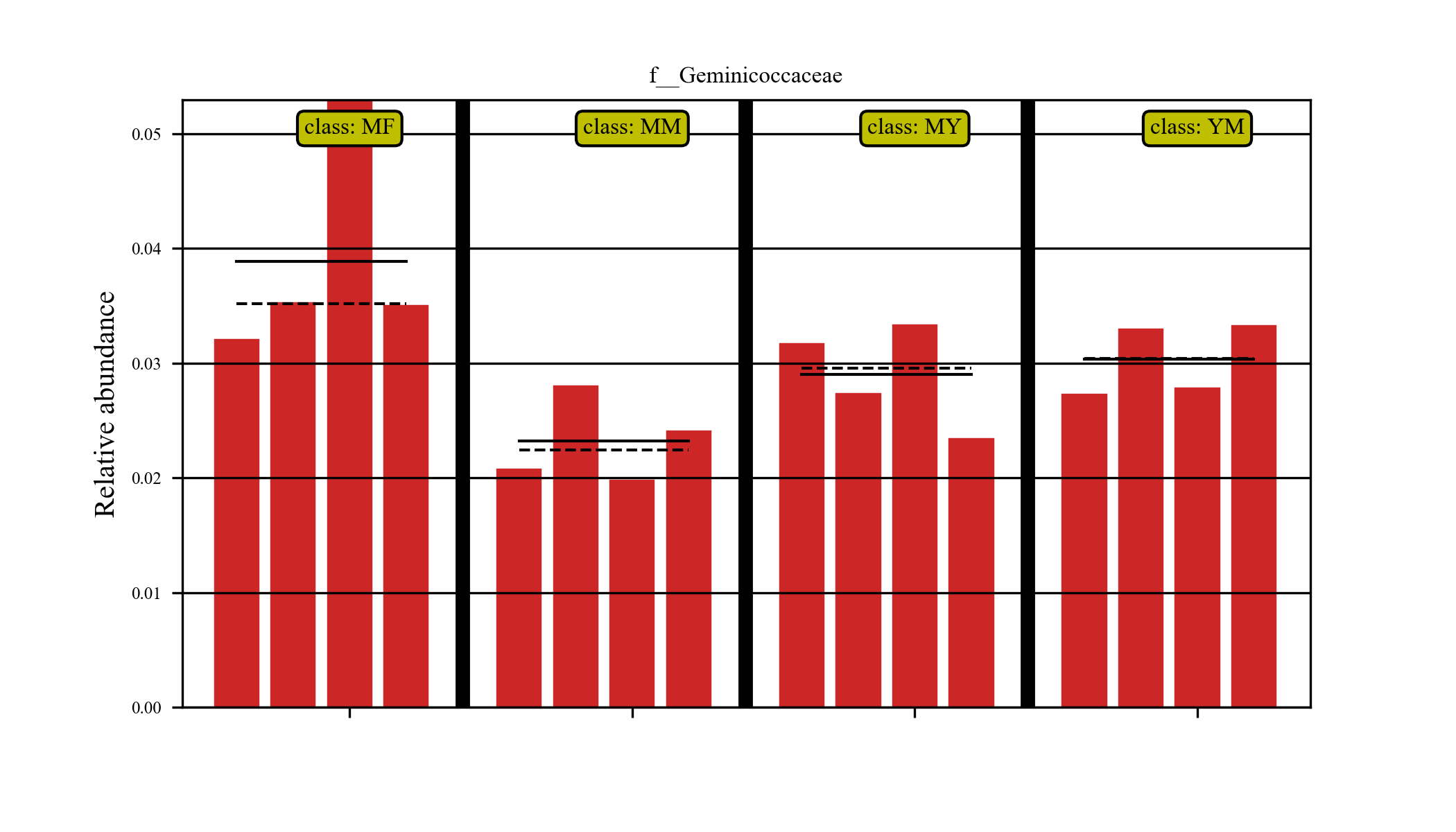

Supplement: Supplementary file 1 [file Data_Sheet_1.ZIP › Supplementary figure 1. bacterial biomarker community/1_f__Geminicoccaceae.png]

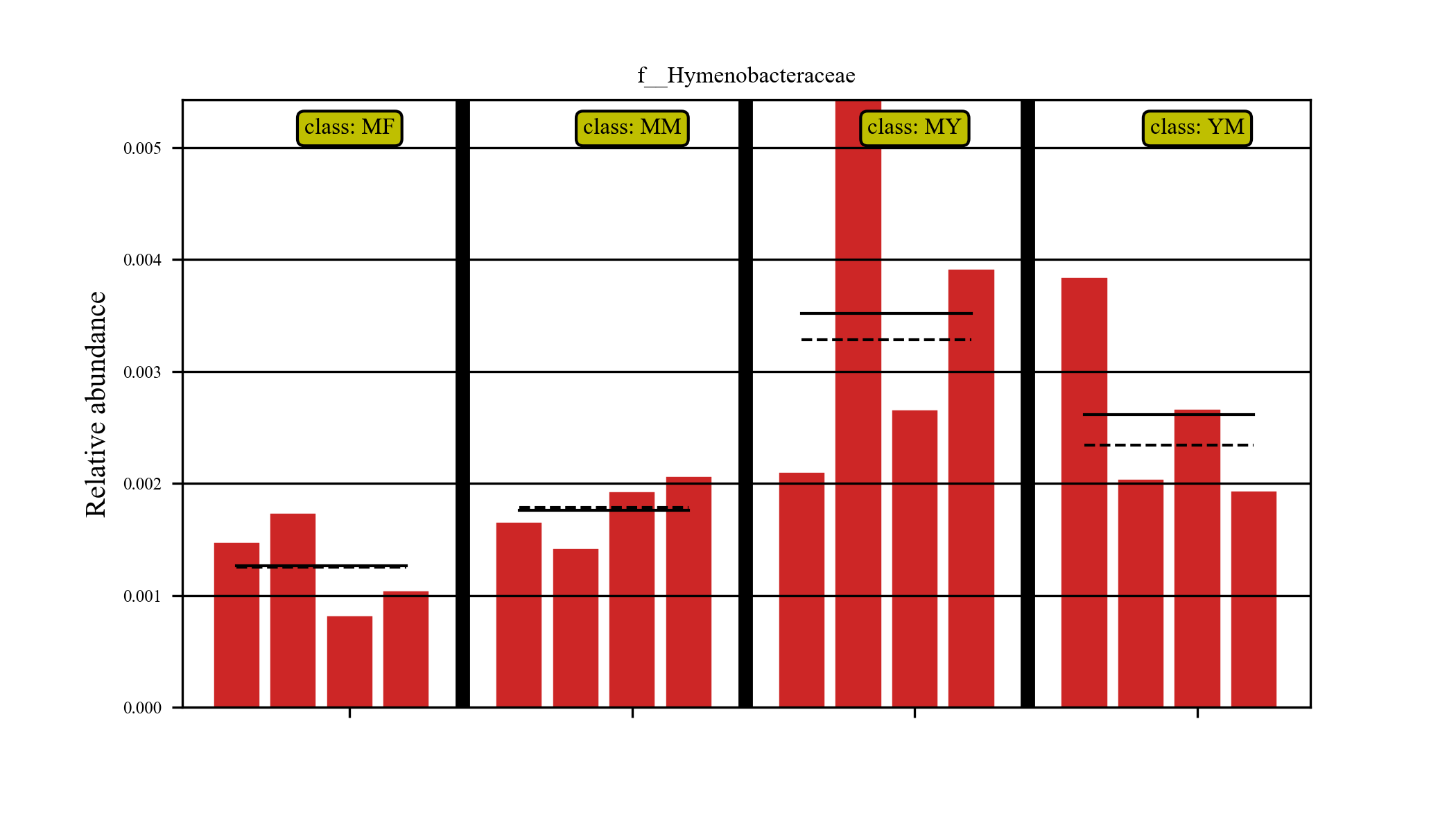

Supplement: Supplementary file 1 [file Data_Sheet_1.ZIP › Supplementary figure 1. bacterial biomarker community/1_f__Hymenobacteraceae.png]

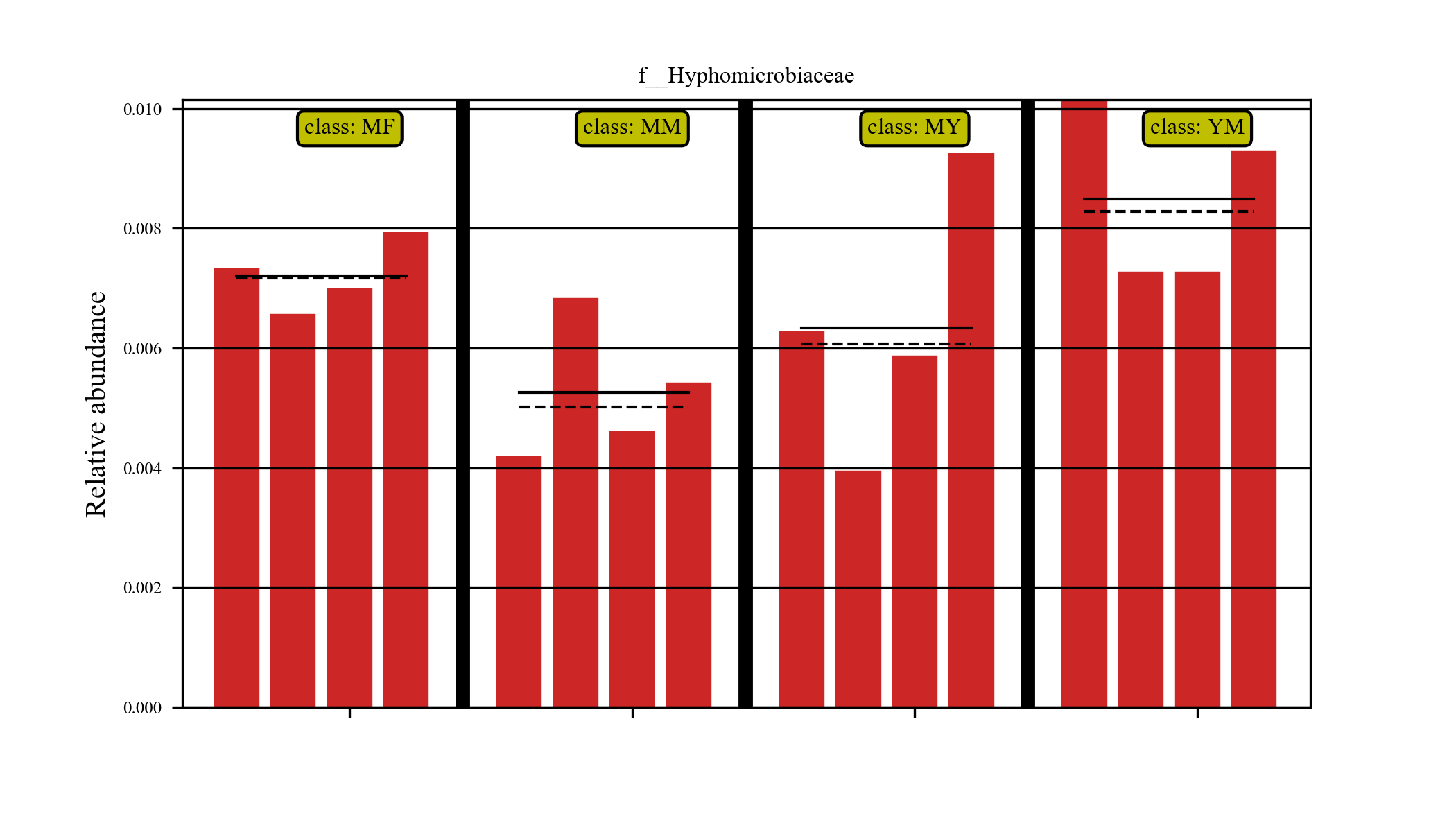

Supplement: Supplementary file 1 [file Data_Sheet_1.ZIP › Supplementary figure 1. bacterial biomarker community/1_f__Hyphomicrobiaceae.png]

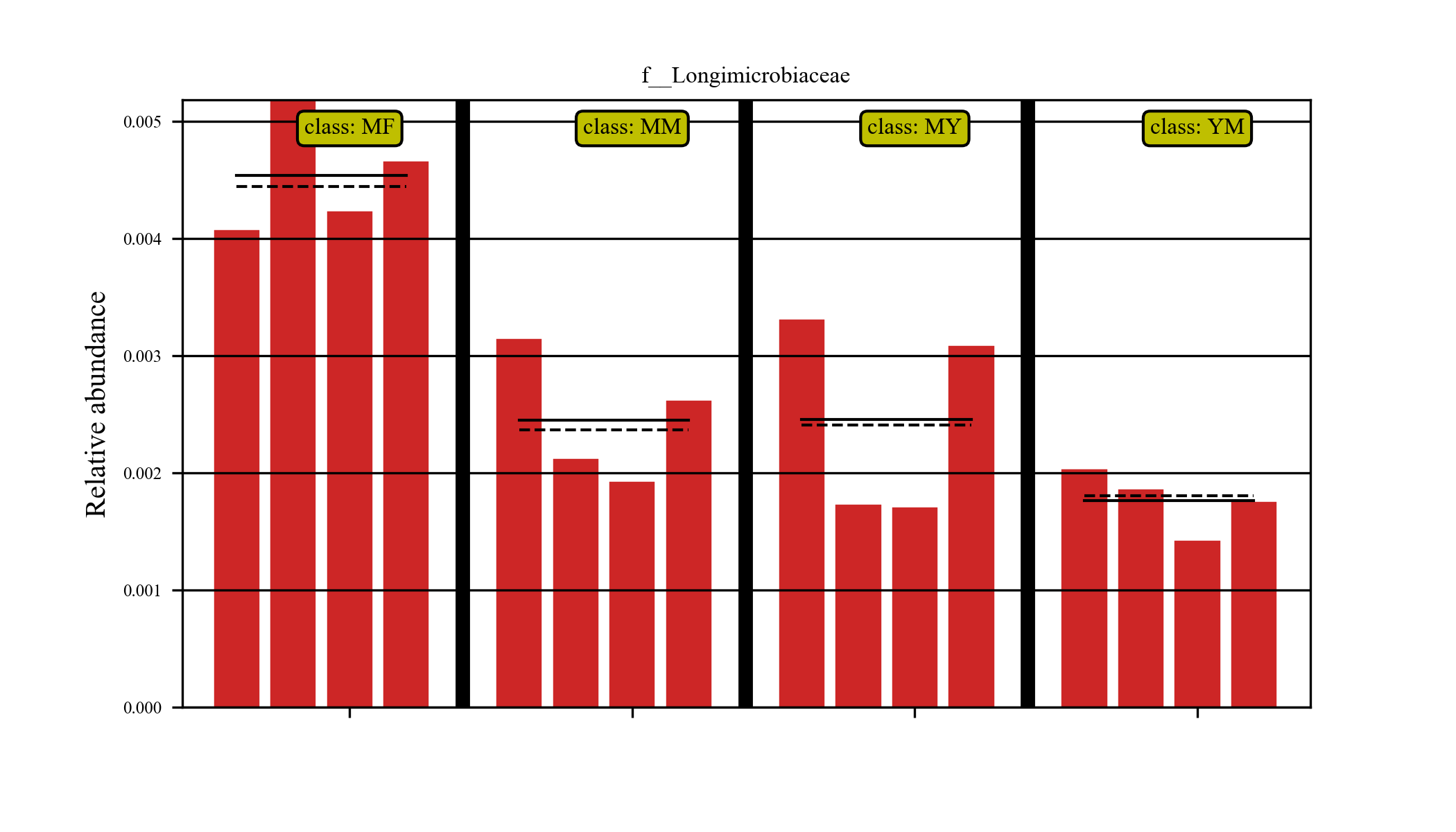

Supplement: Supplementary file 1 [file Data_Sheet_1.ZIP › Supplementary figure 1. bacterial biomarker community/1_f__Longimicrobiaceae.png]

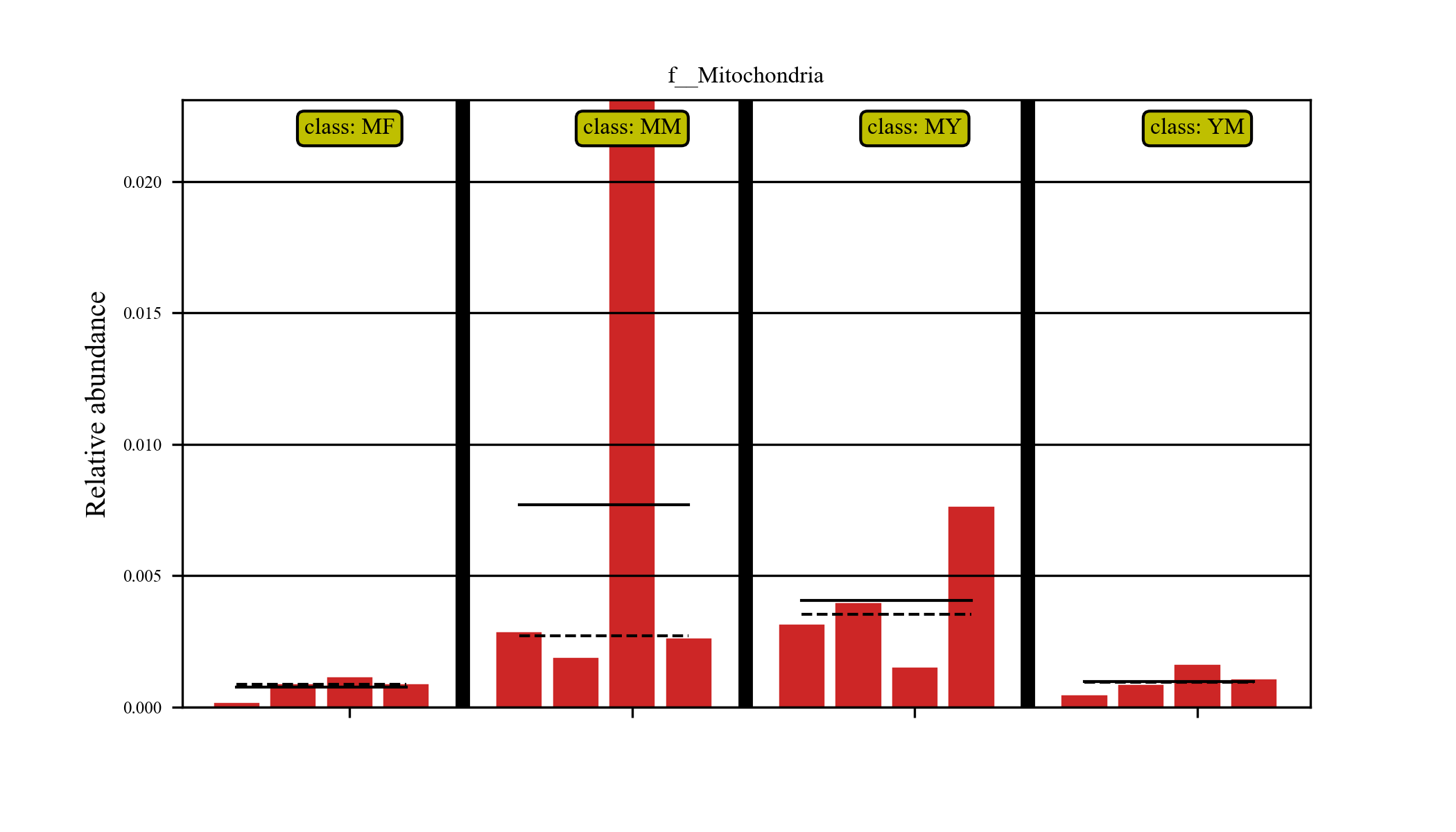

Supplement: Supplementary file 1 [file Data_Sheet_1.ZIP › Supplementary figure 1. bacterial biomarker community/1_f__Mitochondria.png]

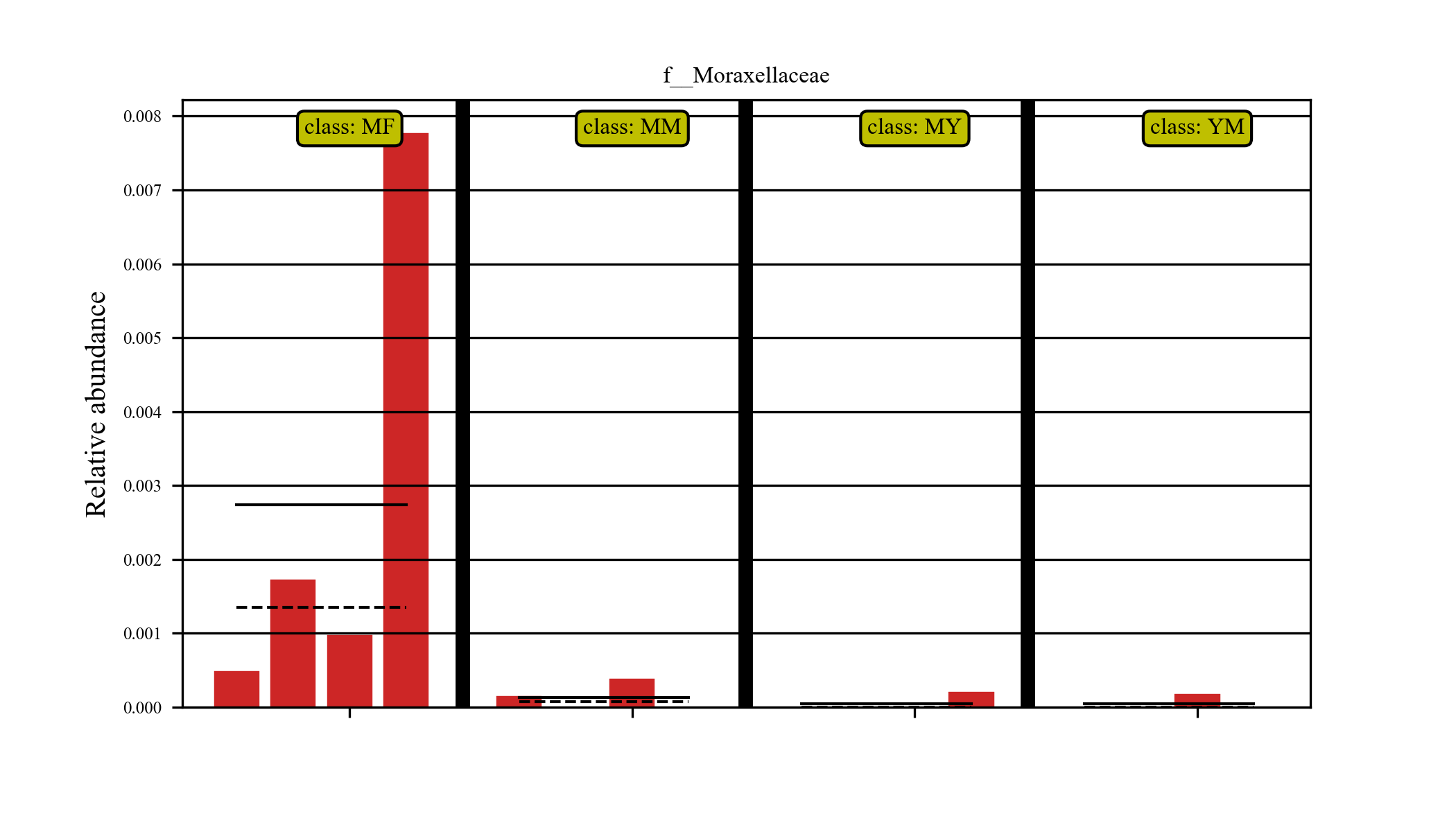

Supplement: Supplementary file 1 [file Data_Sheet_1.ZIP › Supplementary figure 1. bacterial biomarker community/1_f__Moraxellaceae.png]

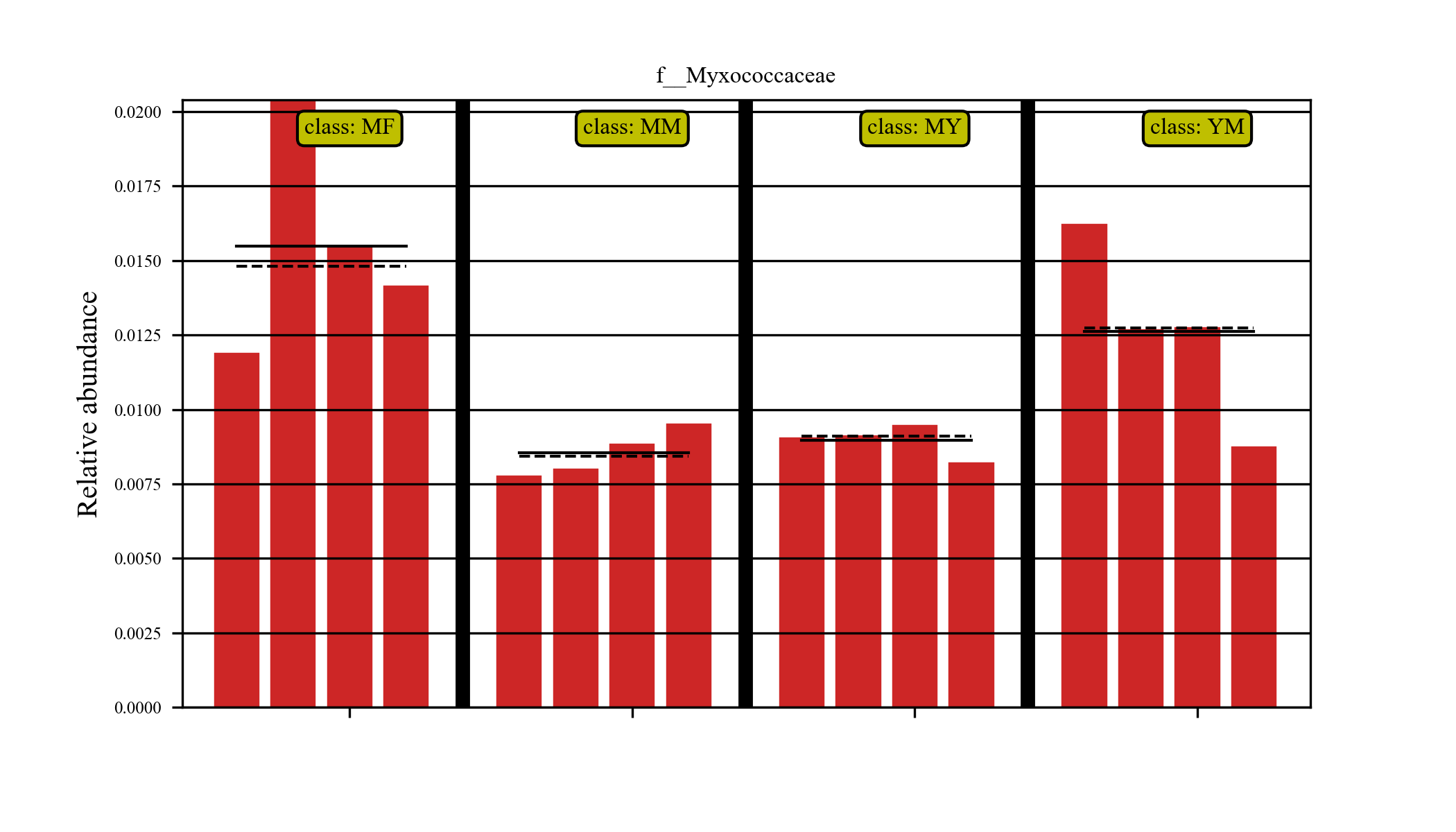

Supplement: Supplementary file 1 [file Data_Sheet_1.ZIP › Supplementary figure 1. bacterial biomarker community/1_f__Myxococcaceae.png]

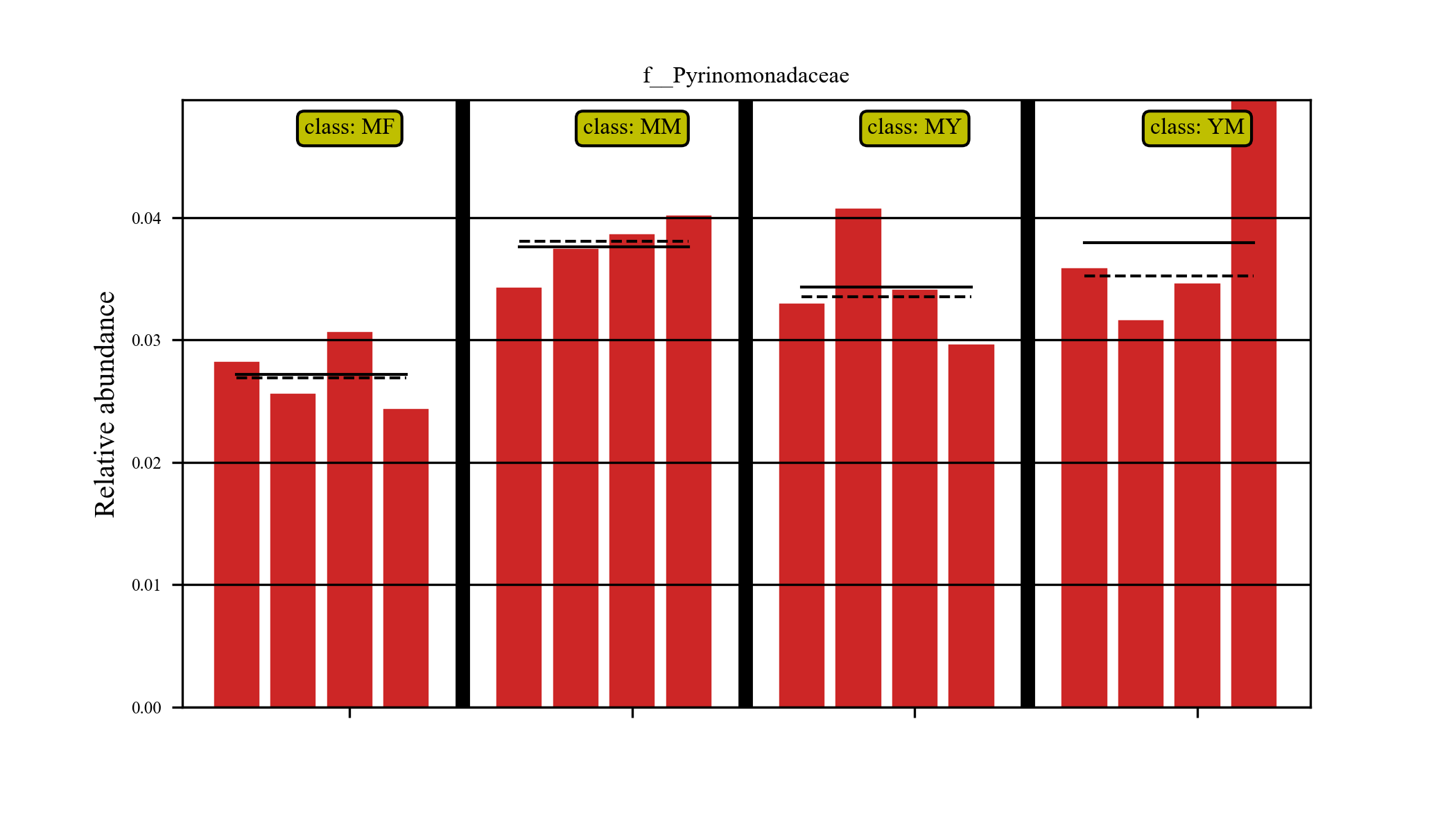

Supplement: Supplementary file 1 [file Data_Sheet_1.ZIP › Supplementary figure 1. bacterial biomarker community/1_f__Pyrinomonadaceae.png]

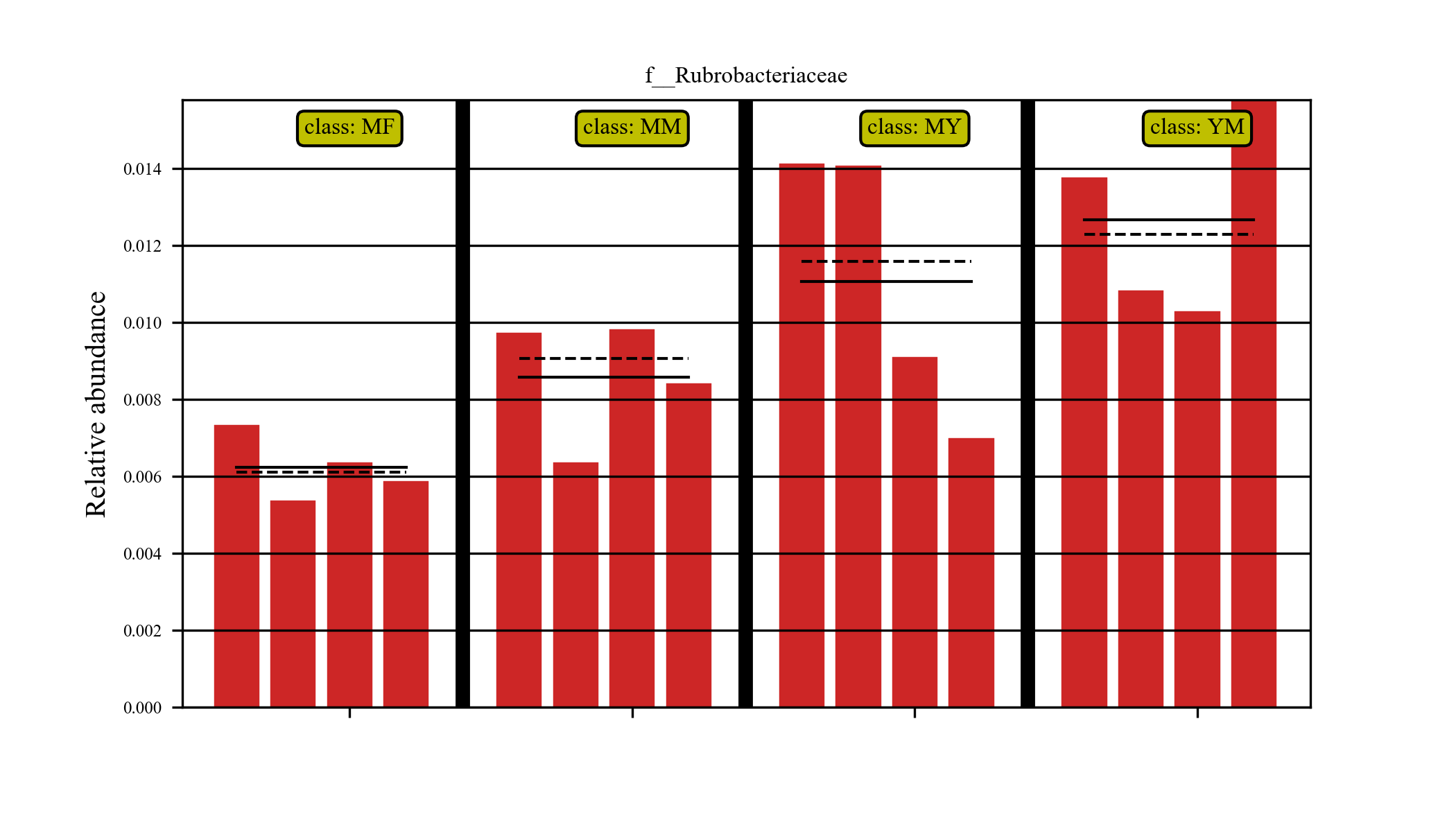

Supplement: Supplementary file 1 [file Data_Sheet_1.ZIP › Supplementary figure 1. bacterial biomarker community/1_f__Rubrobacteriaceae.png]

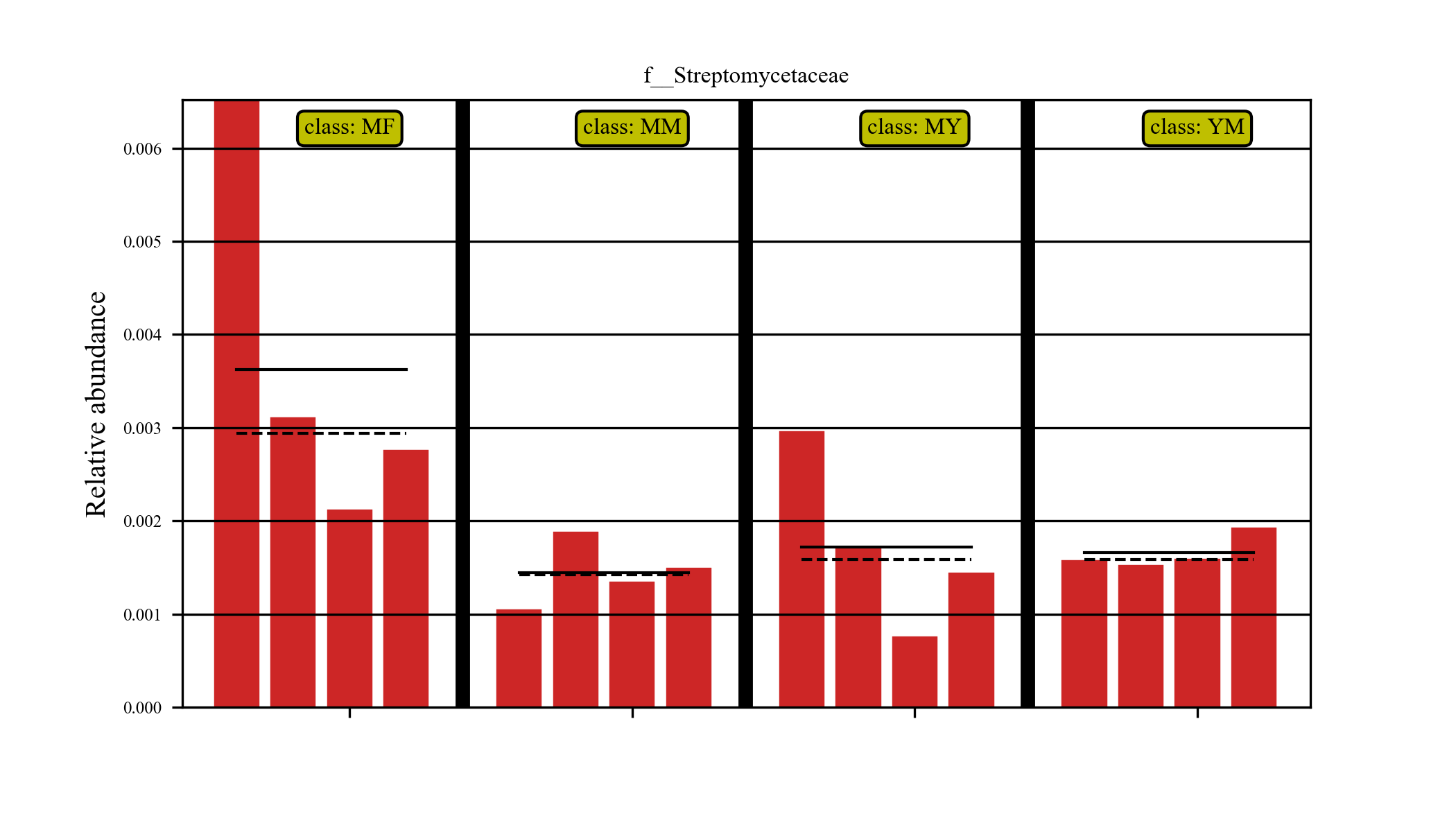

Supplement: Supplementary file 1 [file Data_Sheet_1.ZIP › Supplementary figure 1. bacterial biomarker community/1_f__Streptomycetaceae.png]

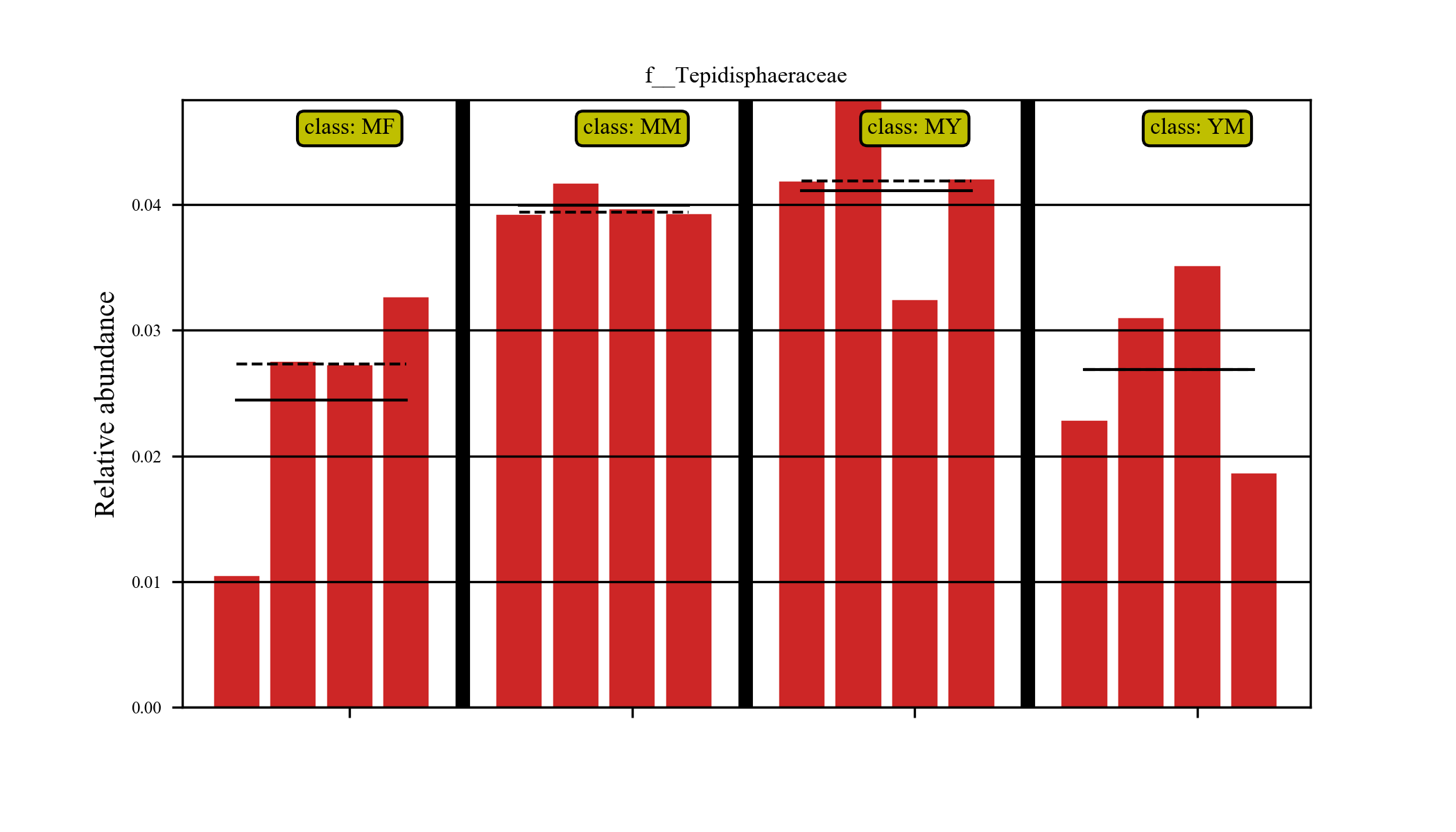

Supplement: Supplementary file 1 [file Data_Sheet_1.ZIP › Supplementary figure 1. bacterial biomarker community/1_f__Tepidisphaeraceae.png]

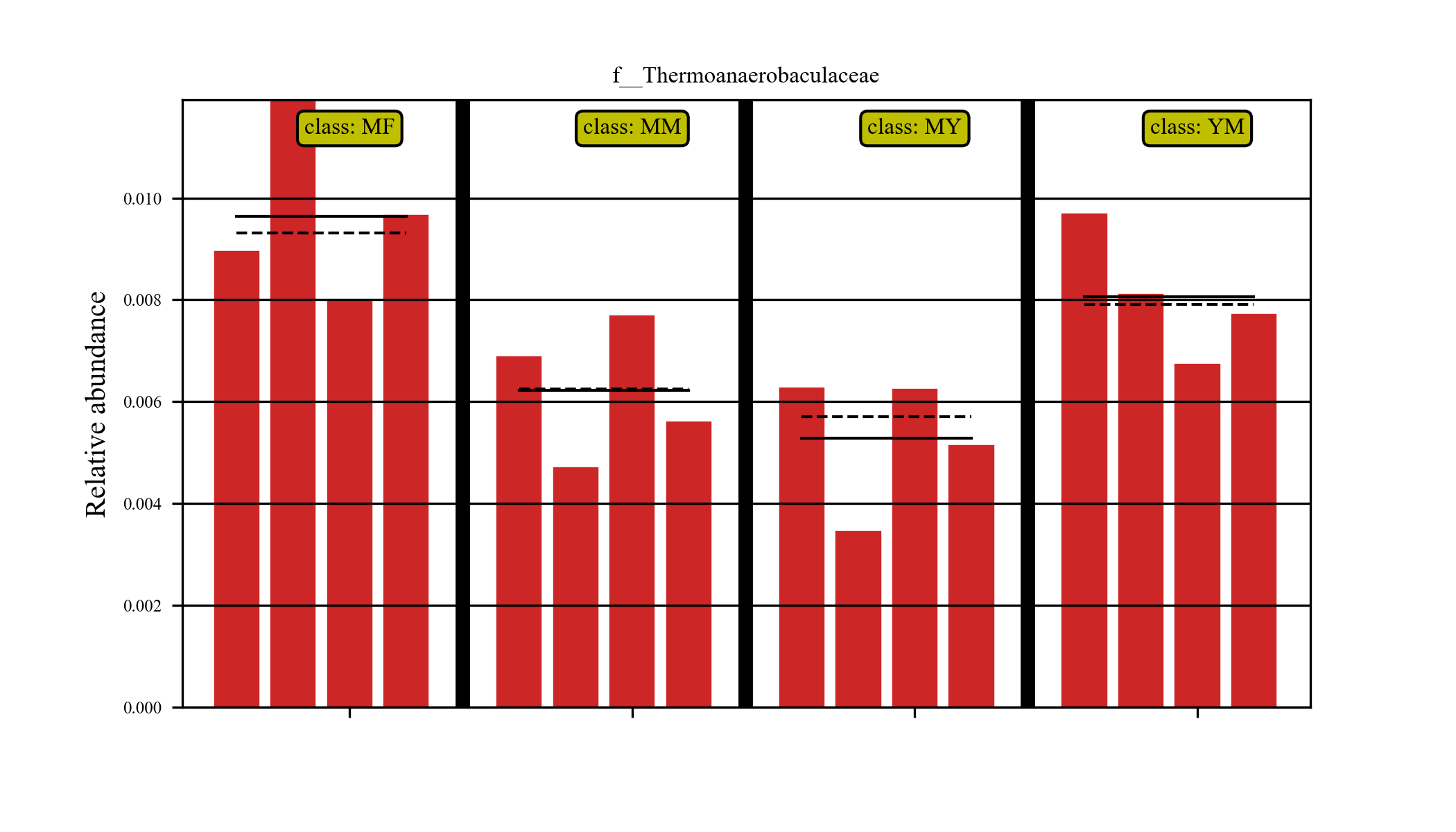

Supplement: Supplementary file 1 [file Data_Sheet_1.ZIP › Supplementary figure 1. bacterial biomarker community/1_f__Thermoanaerobaculaceae.png]

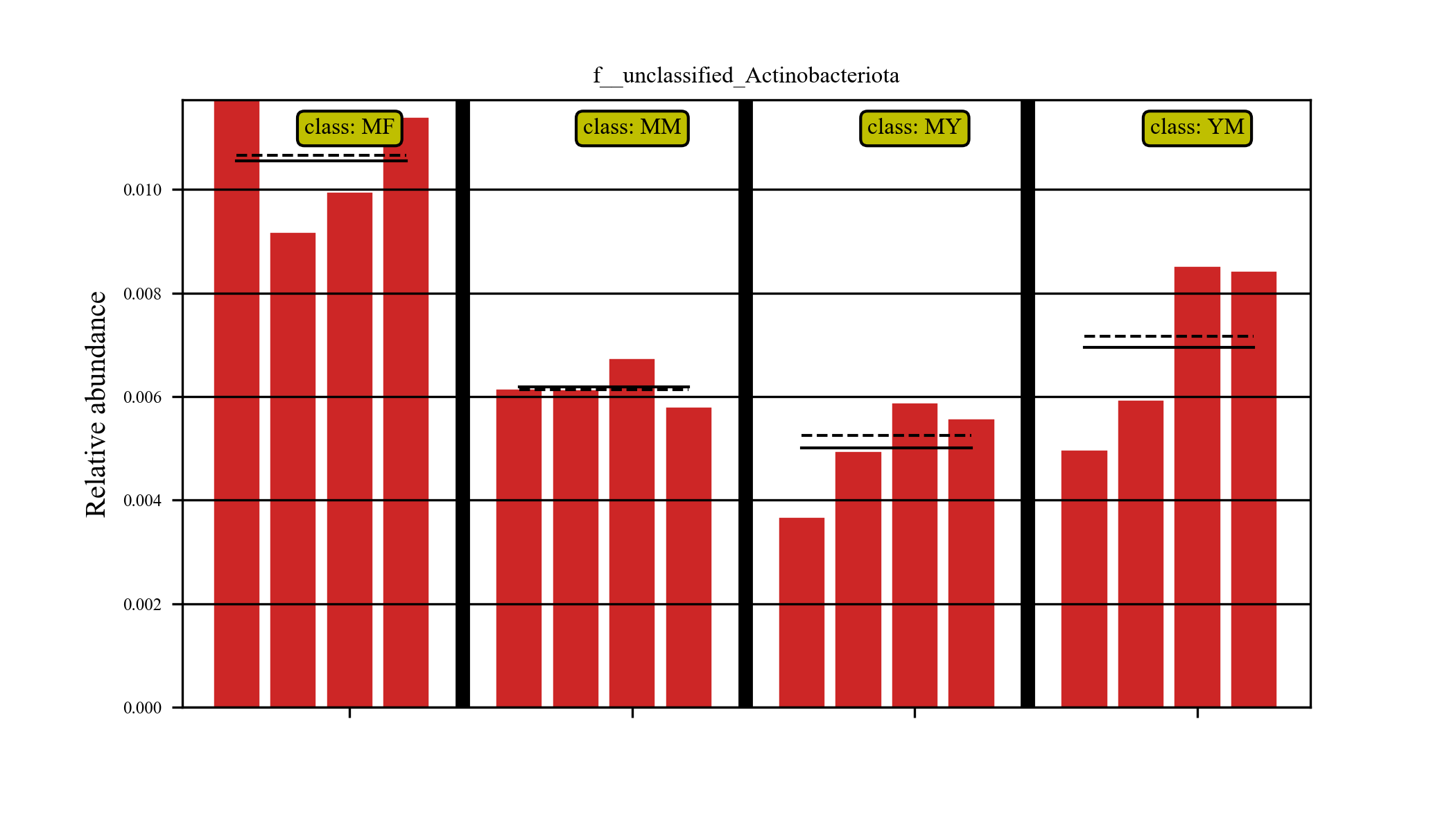

Supplement: Supplementary file 1 [file Data_Sheet_1.ZIP › Supplementary figure 1. bacterial biomarker community/1_f__unclassified_Actinobacteriota.png]

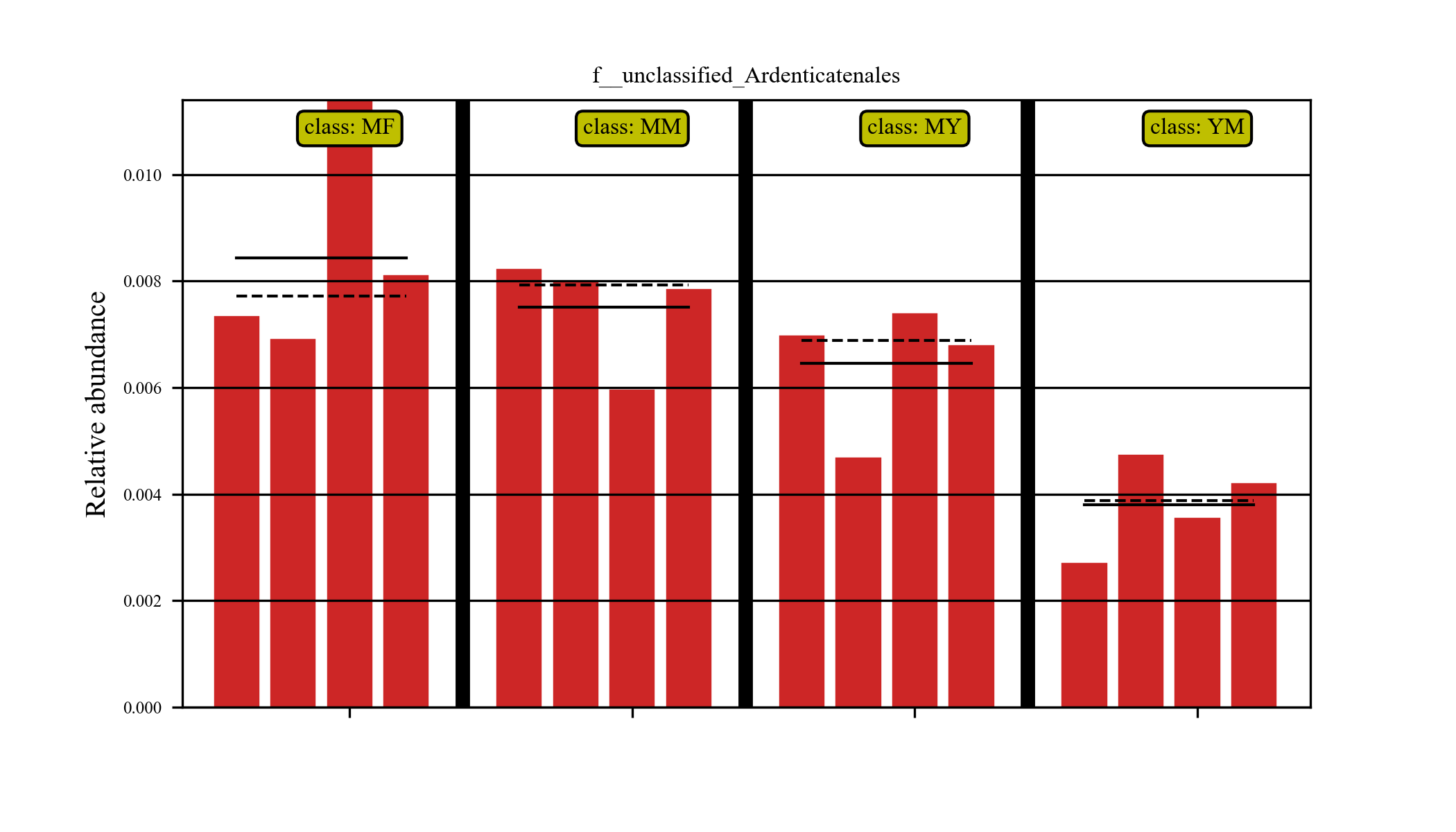

Supplement: Supplementary file 1 [file Data_Sheet_1.ZIP › Supplementary figure 1. bacterial biomarker community/1_f__unclassified_Ardenticatenales.png]

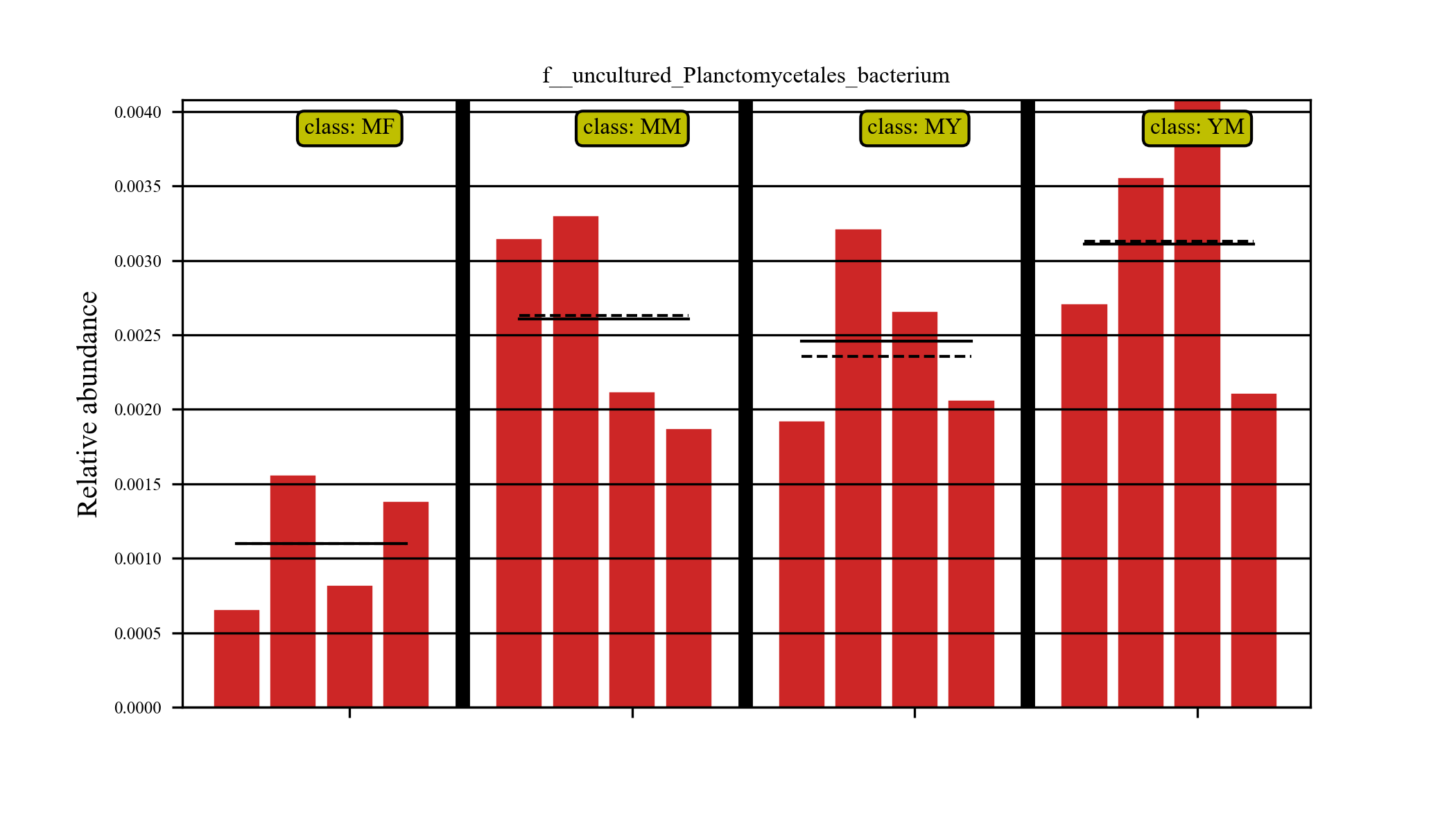

Supplement: Supplementary file 1 [file Data_Sheet_1.ZIP › Supplementary figure 1. bacterial biomarker community/1_f__uncultured_Planctomycetales_bacterium.png]

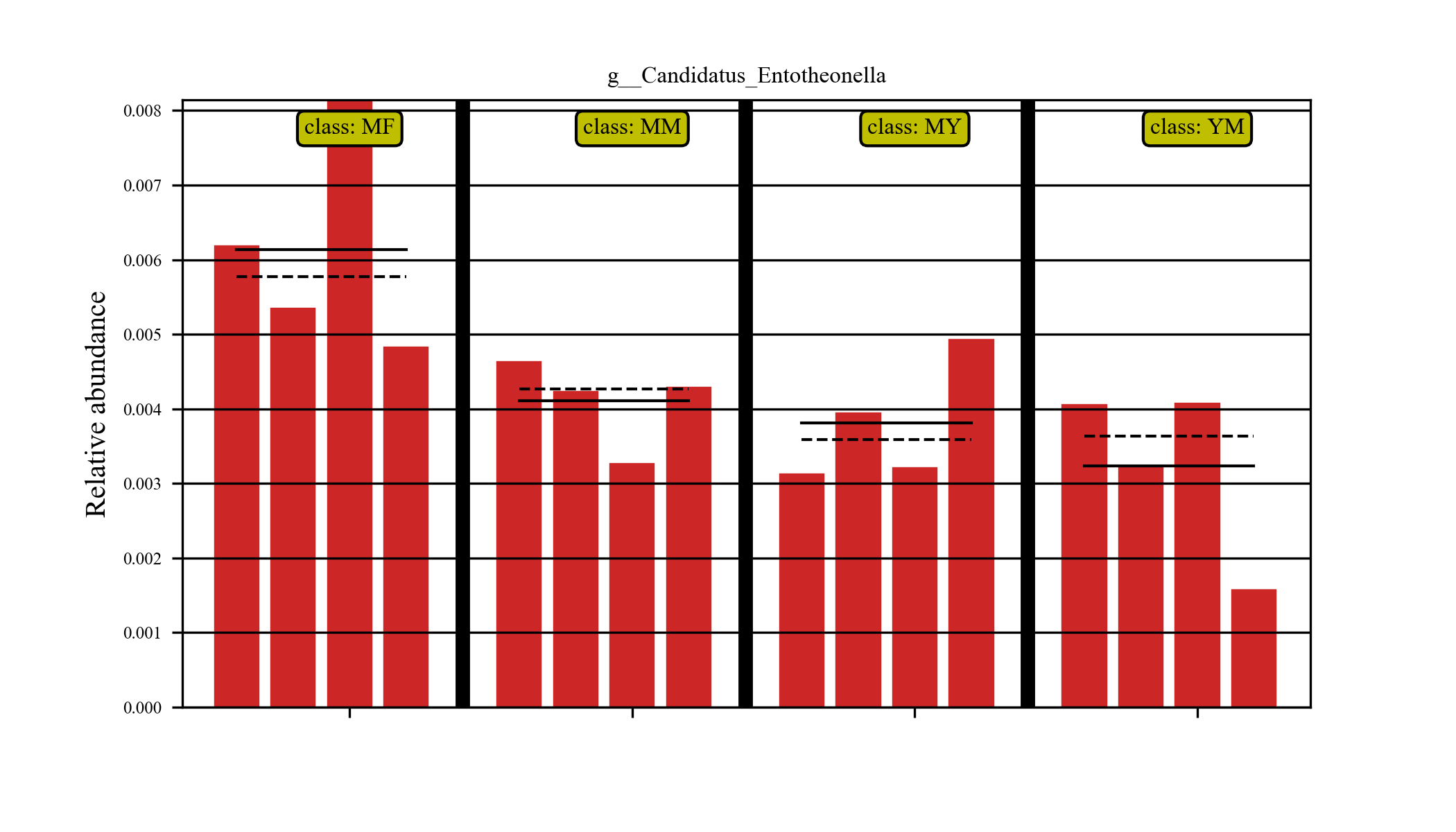

Supplement: Supplementary file 1 [file Data_Sheet_1.ZIP › Supplementary figure 1. bacterial biomarker community/1_g__Candidatus_Entotheonella.png]

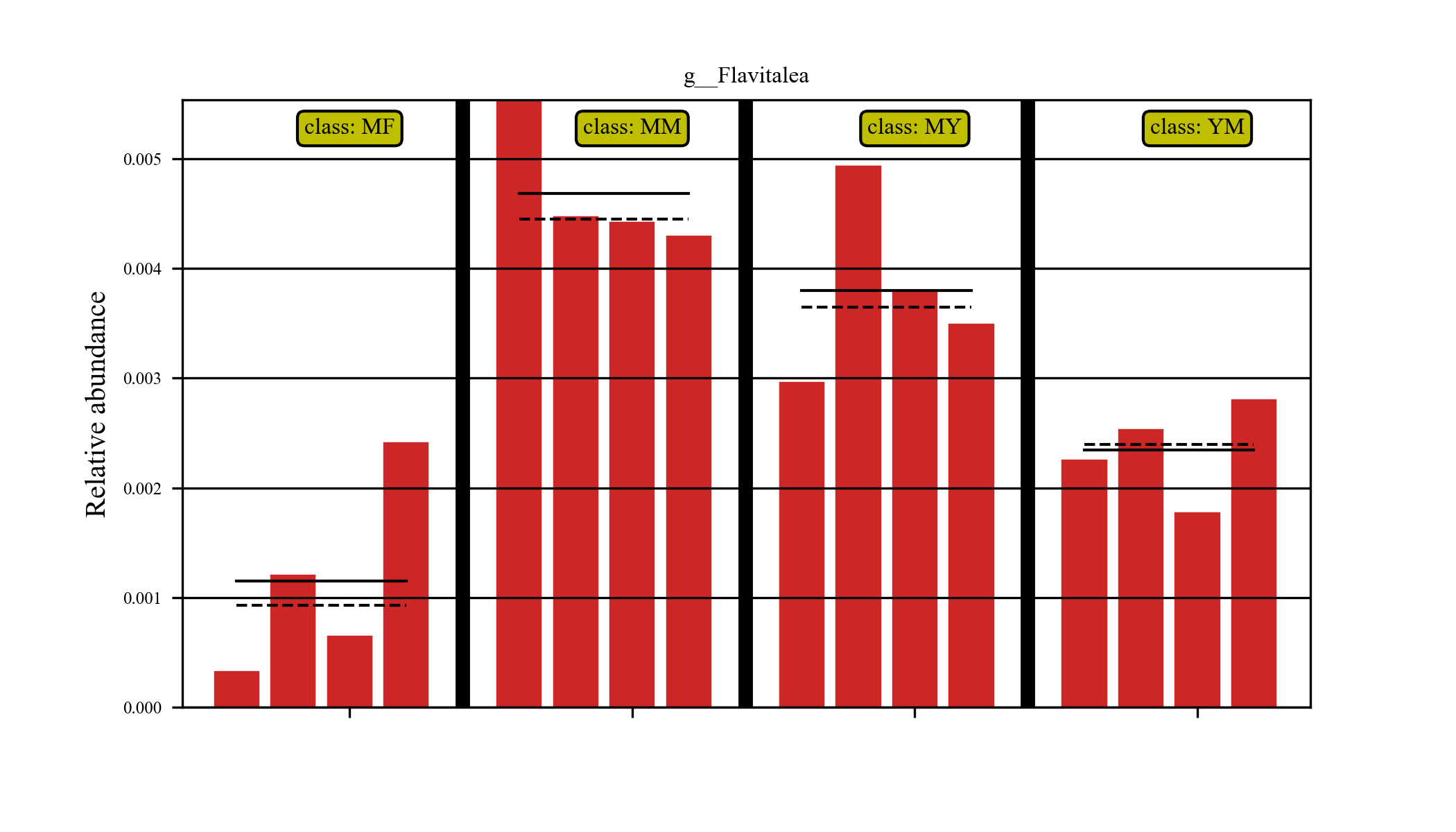

Supplement: Supplementary file 1 [file Data_Sheet_1.ZIP › Supplementary figure 1. bacterial biomarker community/1_g__Flavitalea.png]

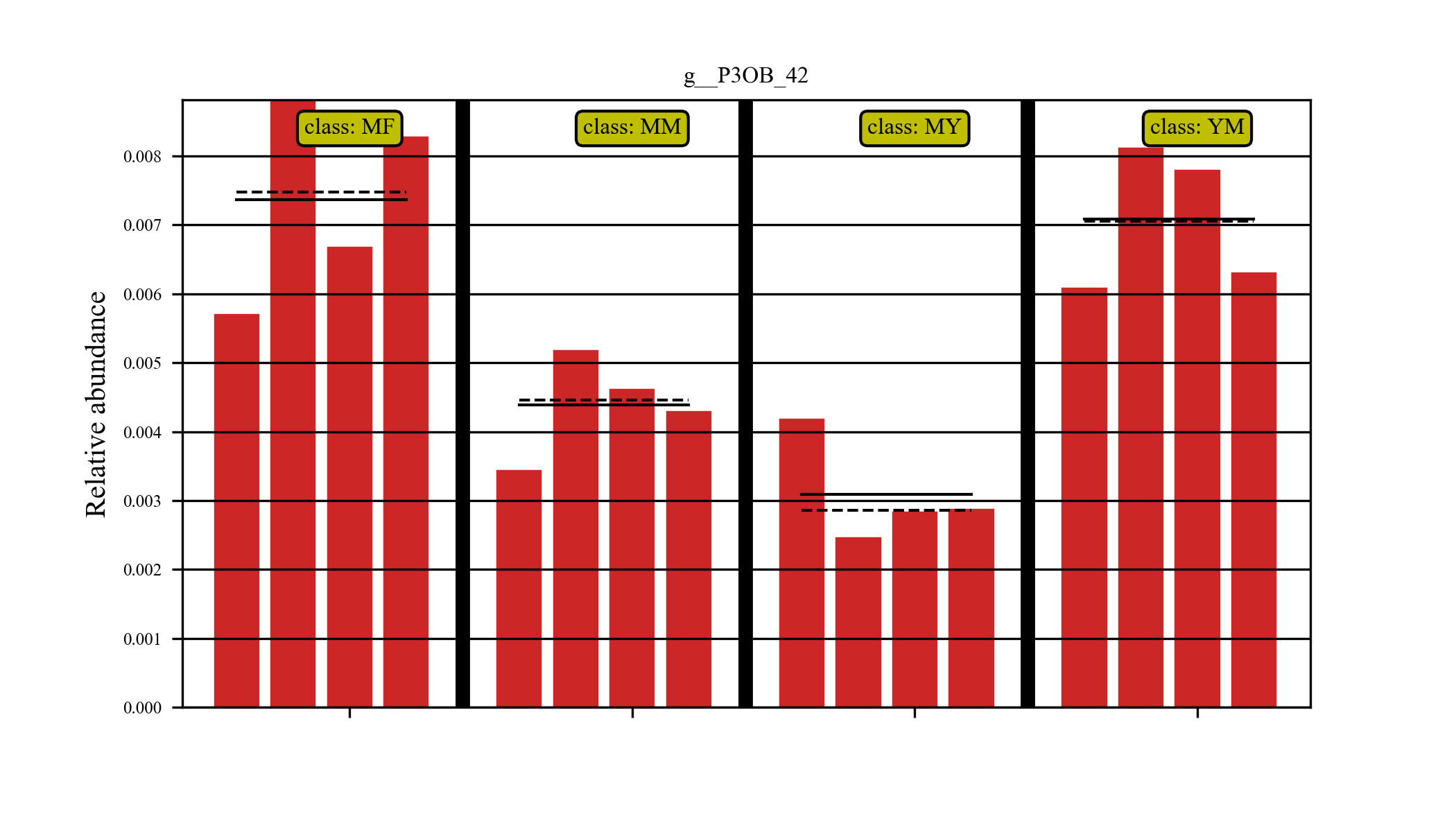

Supplement: Supplementary file 1 [file Data_Sheet_1.ZIP › Supplementary figure 1. bacterial biomarker community/1_g__P3OB_42.png]

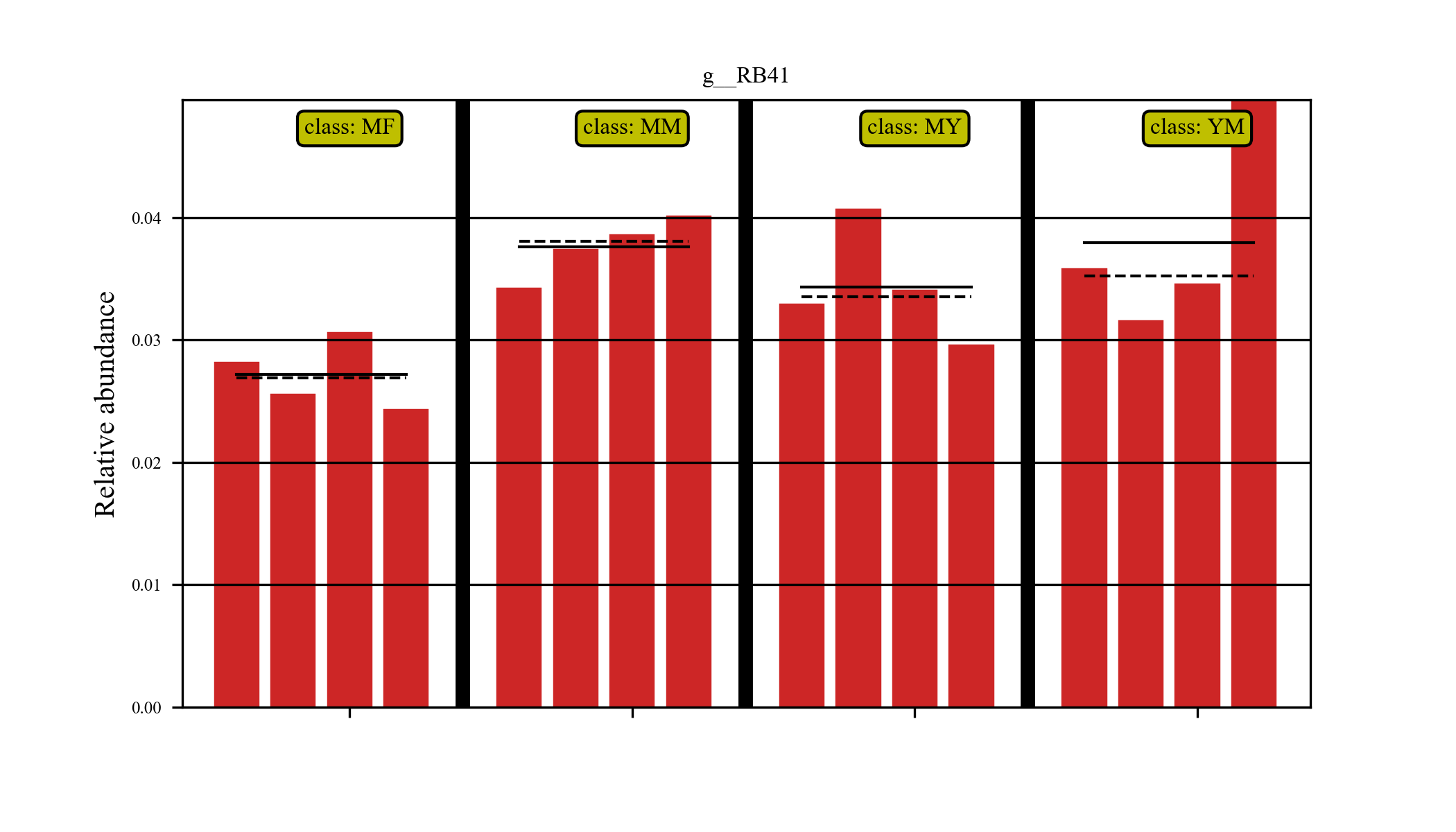

Supplement: Supplementary file 1 [file Data_Sheet_1.ZIP › Supplementary figure 1. bacterial biomarker community/1_g__RB41.png]

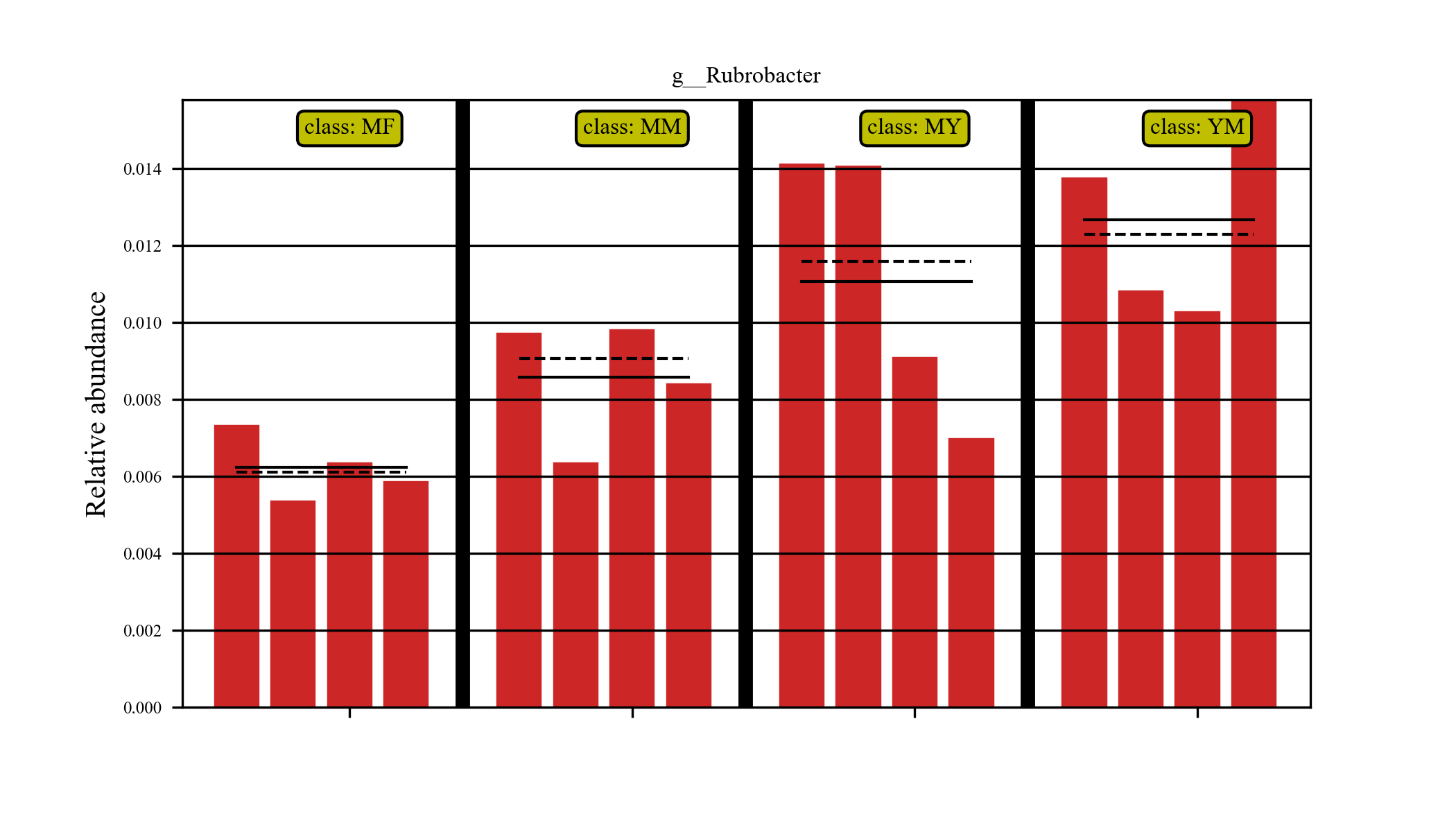

Supplement: Supplementary file 1 [file Data_Sheet_1.ZIP › Supplementary figure 1. bacterial biomarker community/1_g__Rubrobacter.png]

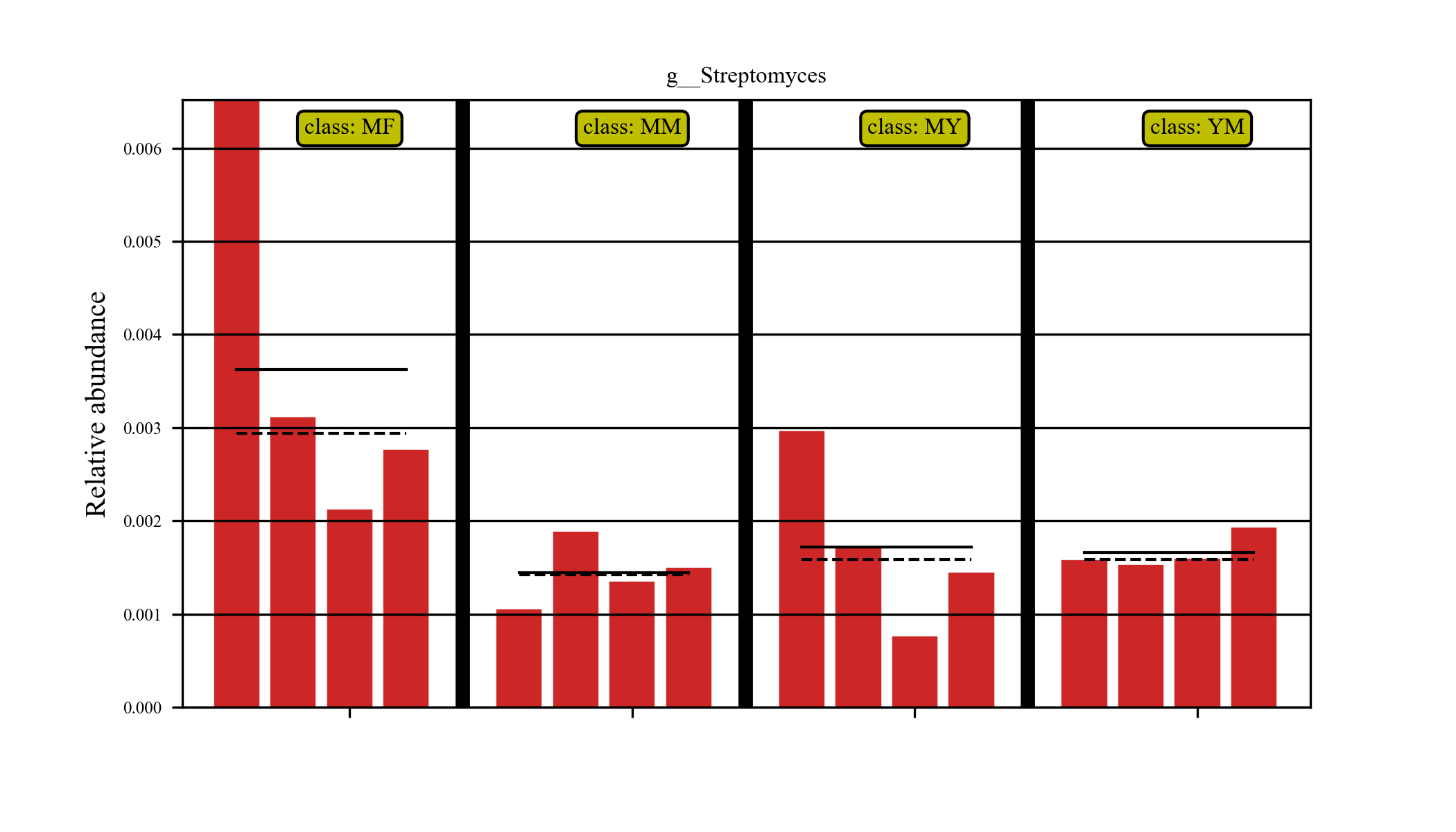

Supplement: Supplementary file 1 [file Data_Sheet_1.ZIP › Supplementary figure 1. bacterial biomarker community/1_g__Streptomyces.png]

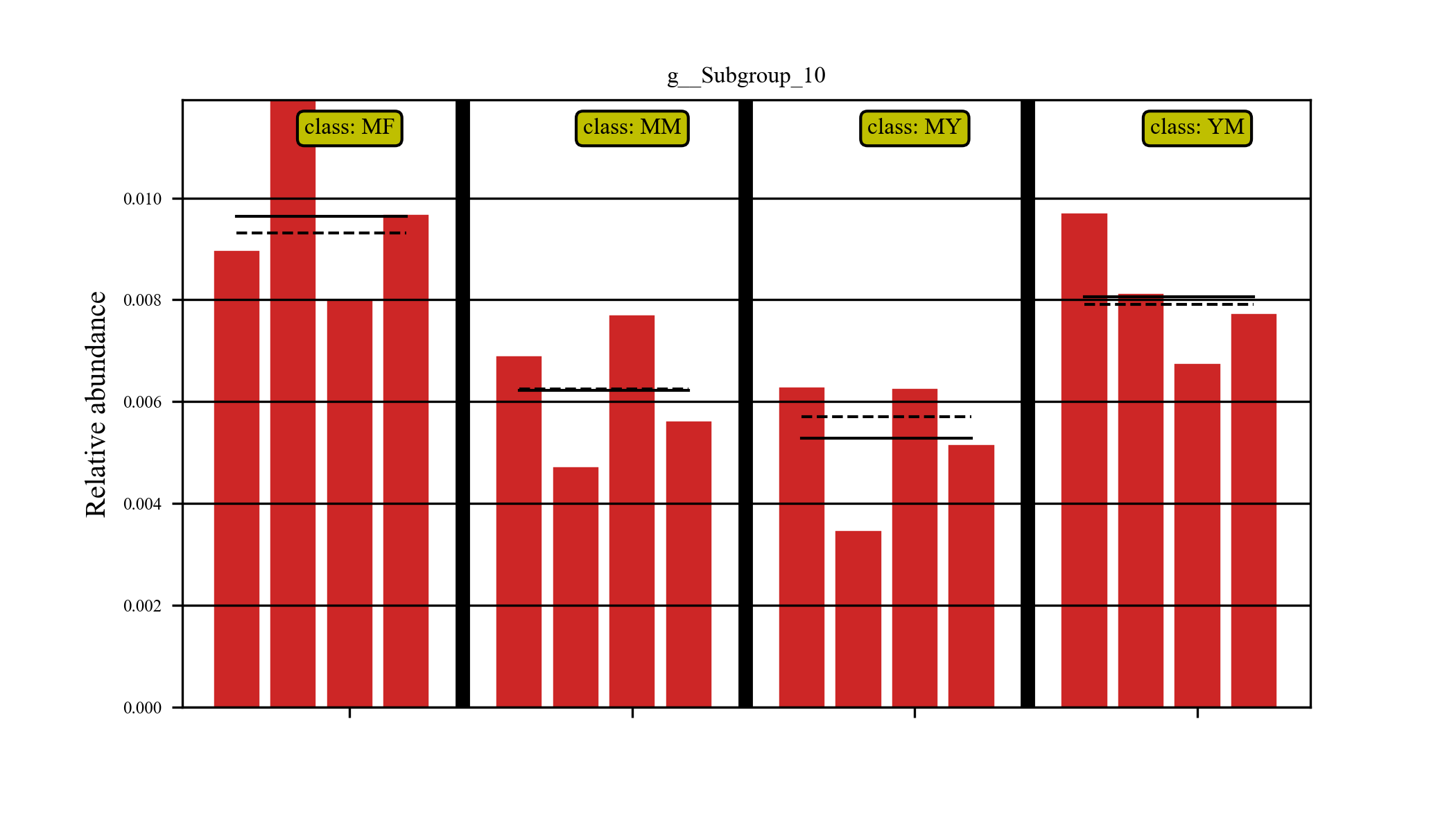

Supplement: Supplementary file 1 [file Data_Sheet_1.ZIP › Supplementary figure 1. bacterial biomarker community/1_g__Subgroup_10.png]

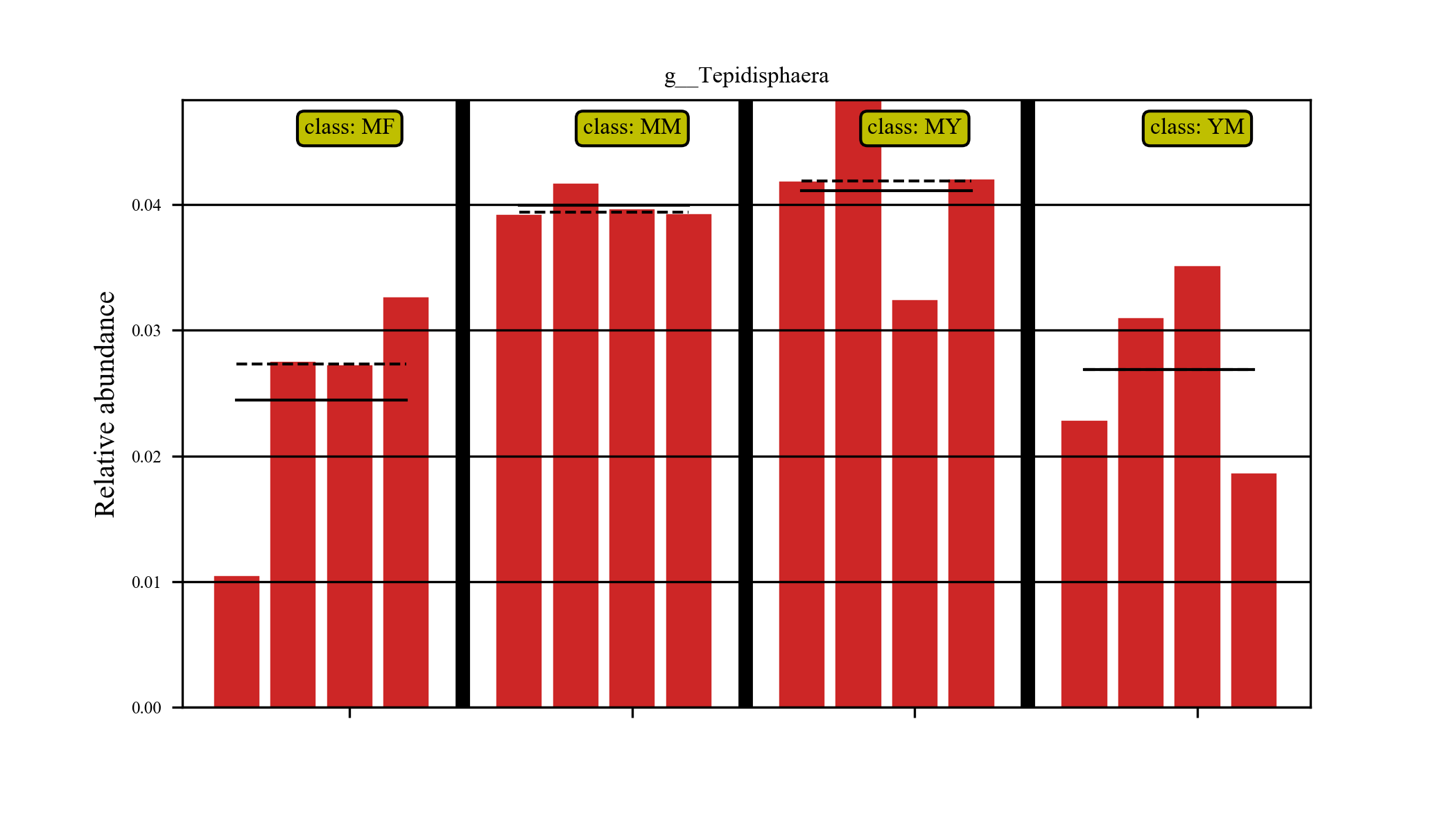

Supplement: Supplementary file 1 [file Data_Sheet_1.ZIP › Supplementary figure 1. bacterial biomarker community/1_g__Tepidisphaera.png]

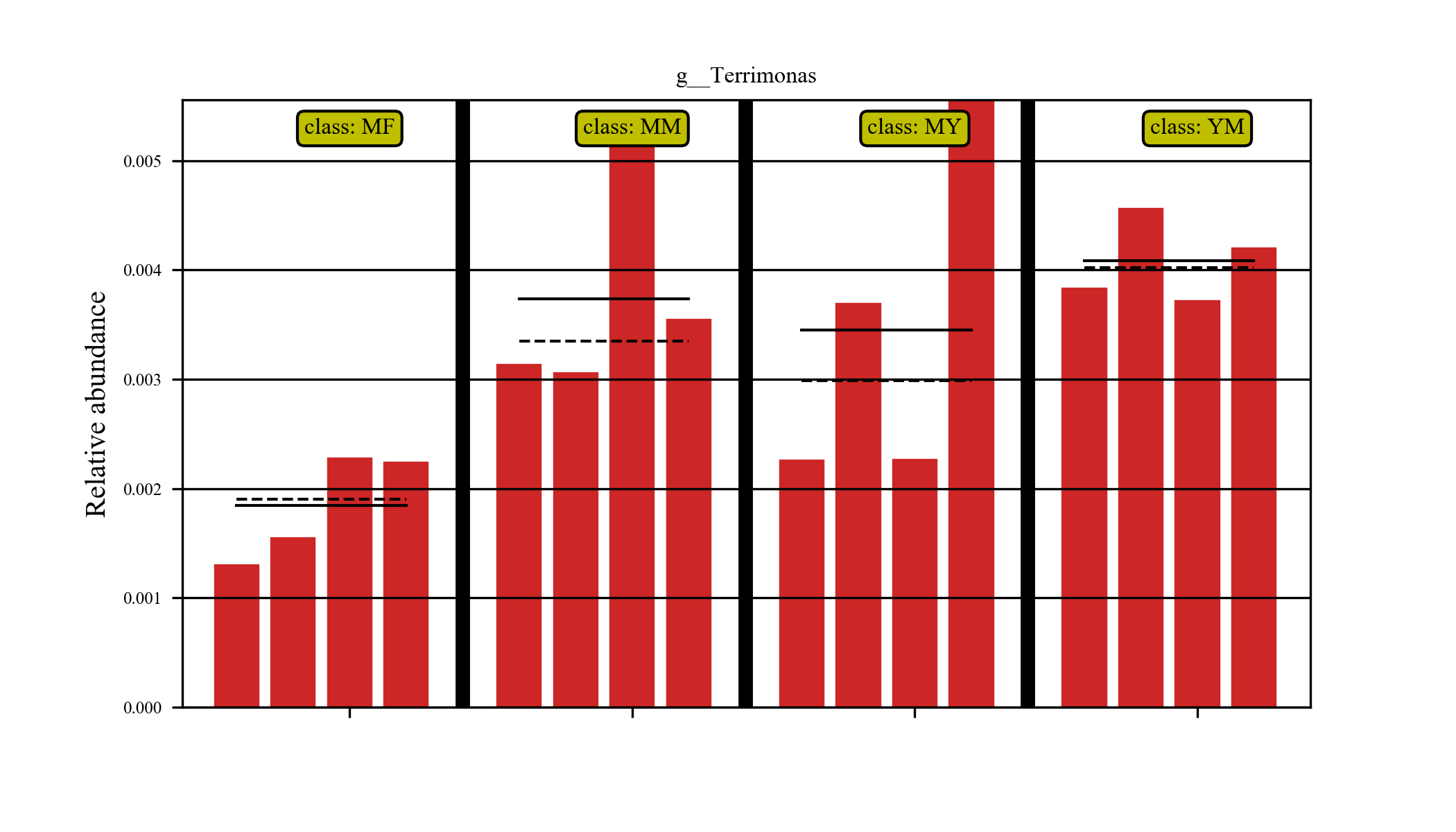

Supplement: Supplementary file 1 [file Data_Sheet_1.ZIP › Supplementary figure 1. bacterial biomarker community/1_g__Terrimonas.png]

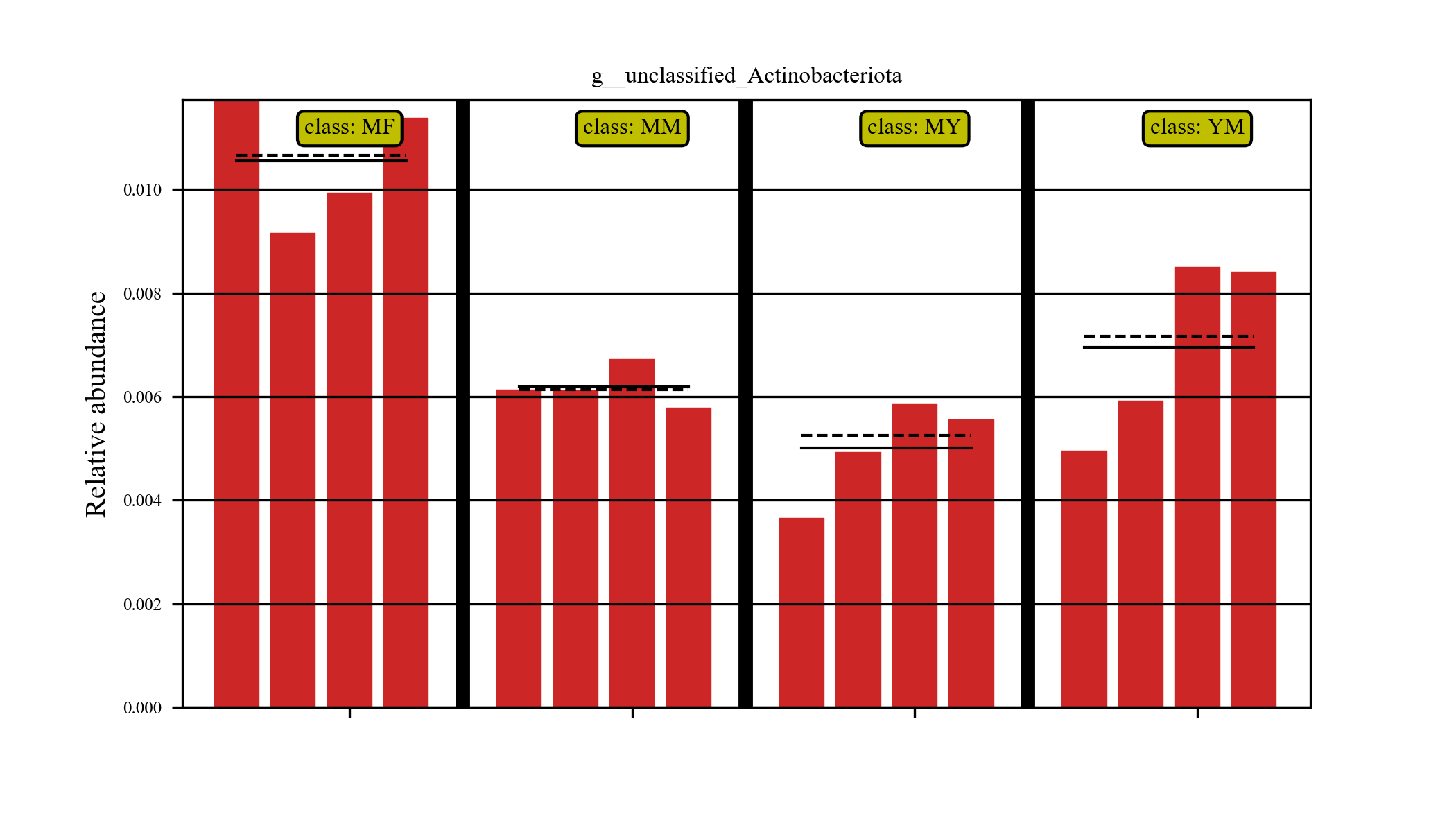

Supplement: Supplementary file 1 [file Data_Sheet_1.ZIP › Supplementary figure 1. bacterial biomarker community/1_g__unclassified_Actinobacteriota.png]

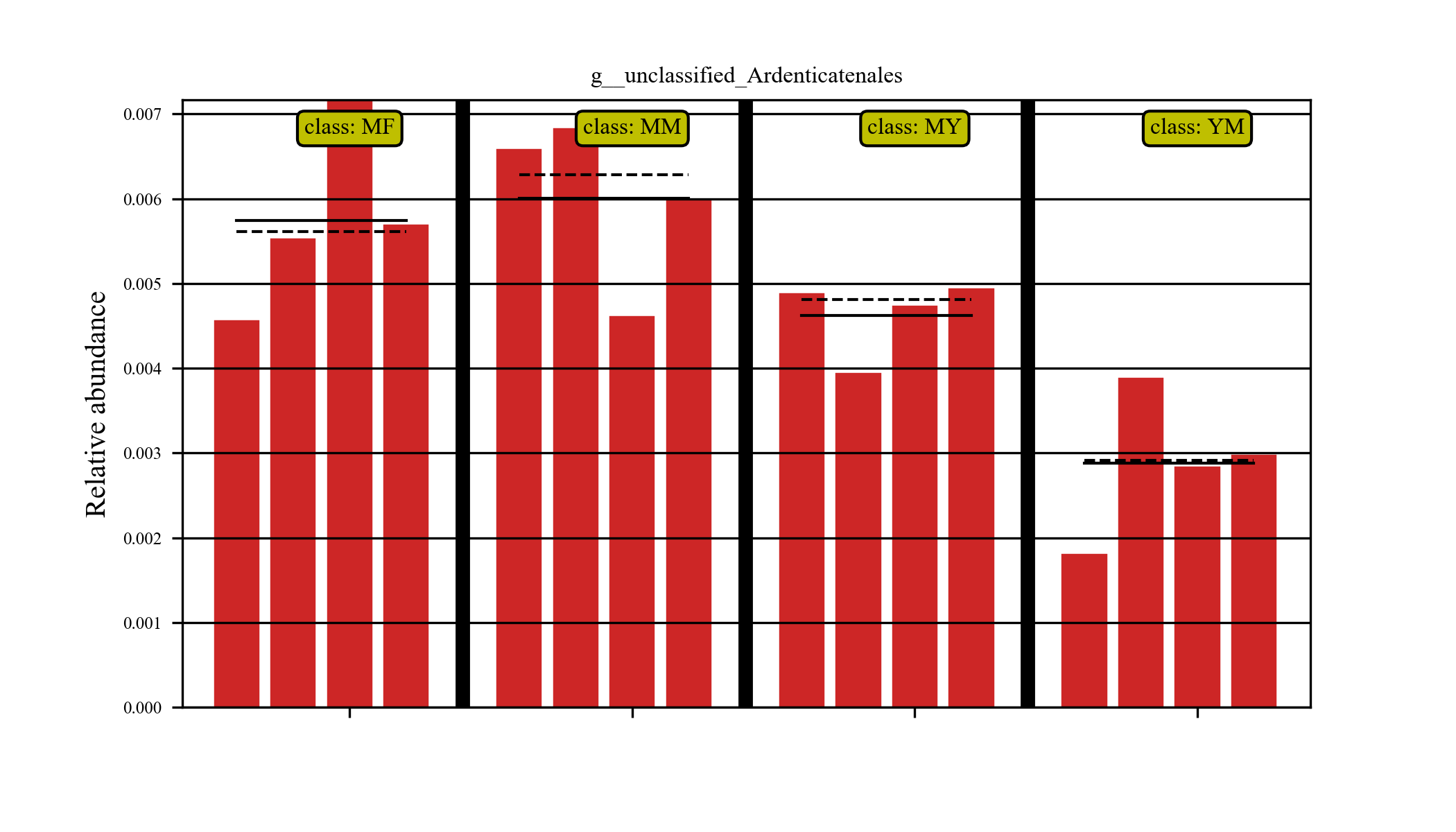

Supplement: Supplementary file 1 [file Data_Sheet_1.ZIP › Supplementary figure 1. bacterial biomarker community/1_g__unclassified_Ardenticatenales.png]

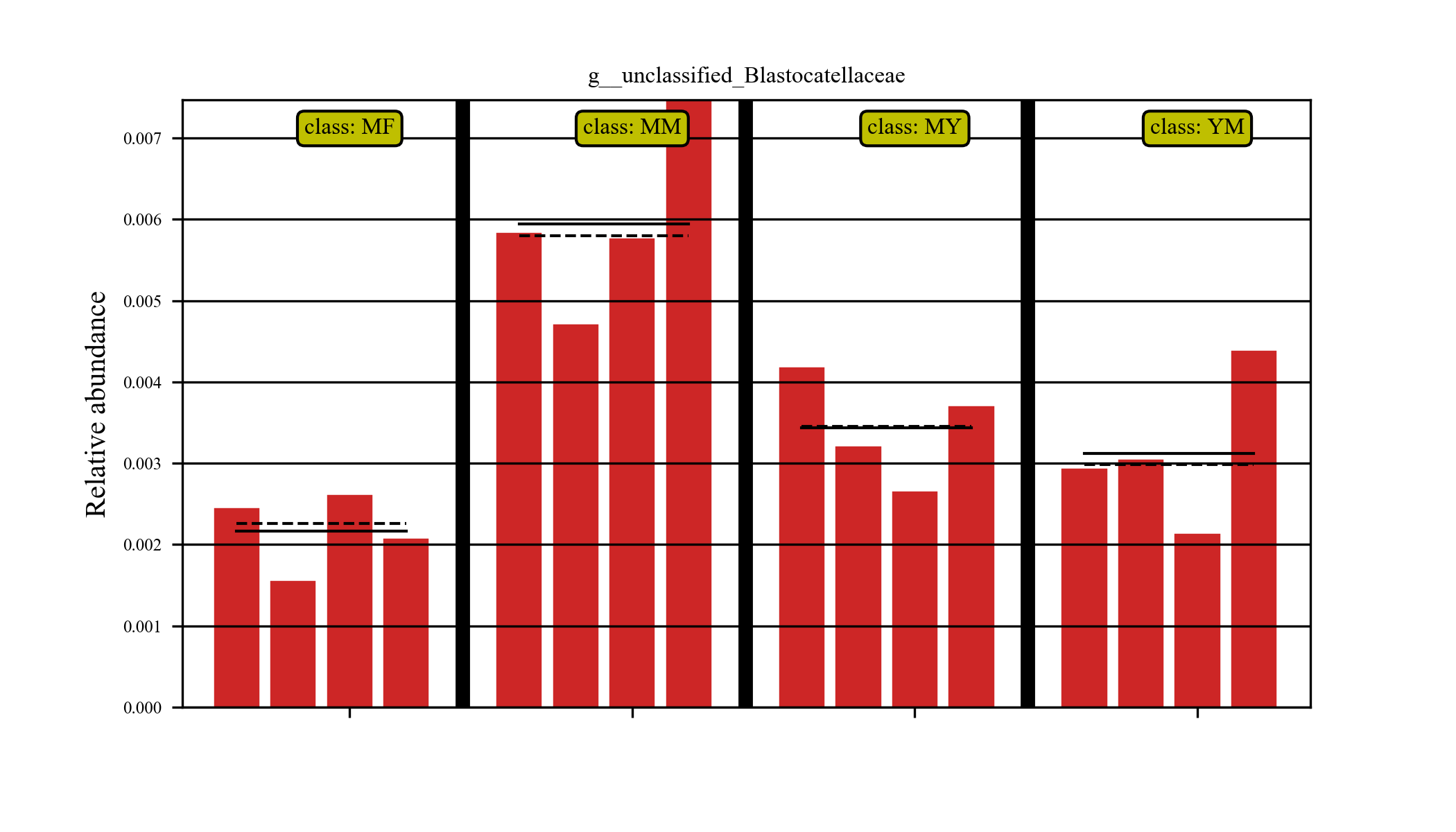

Supplement: Supplementary file 1 [file Data_Sheet_1.ZIP › Supplementary figure 1. bacterial biomarker community/1_g__unclassified_Blastocatellaceae.png]

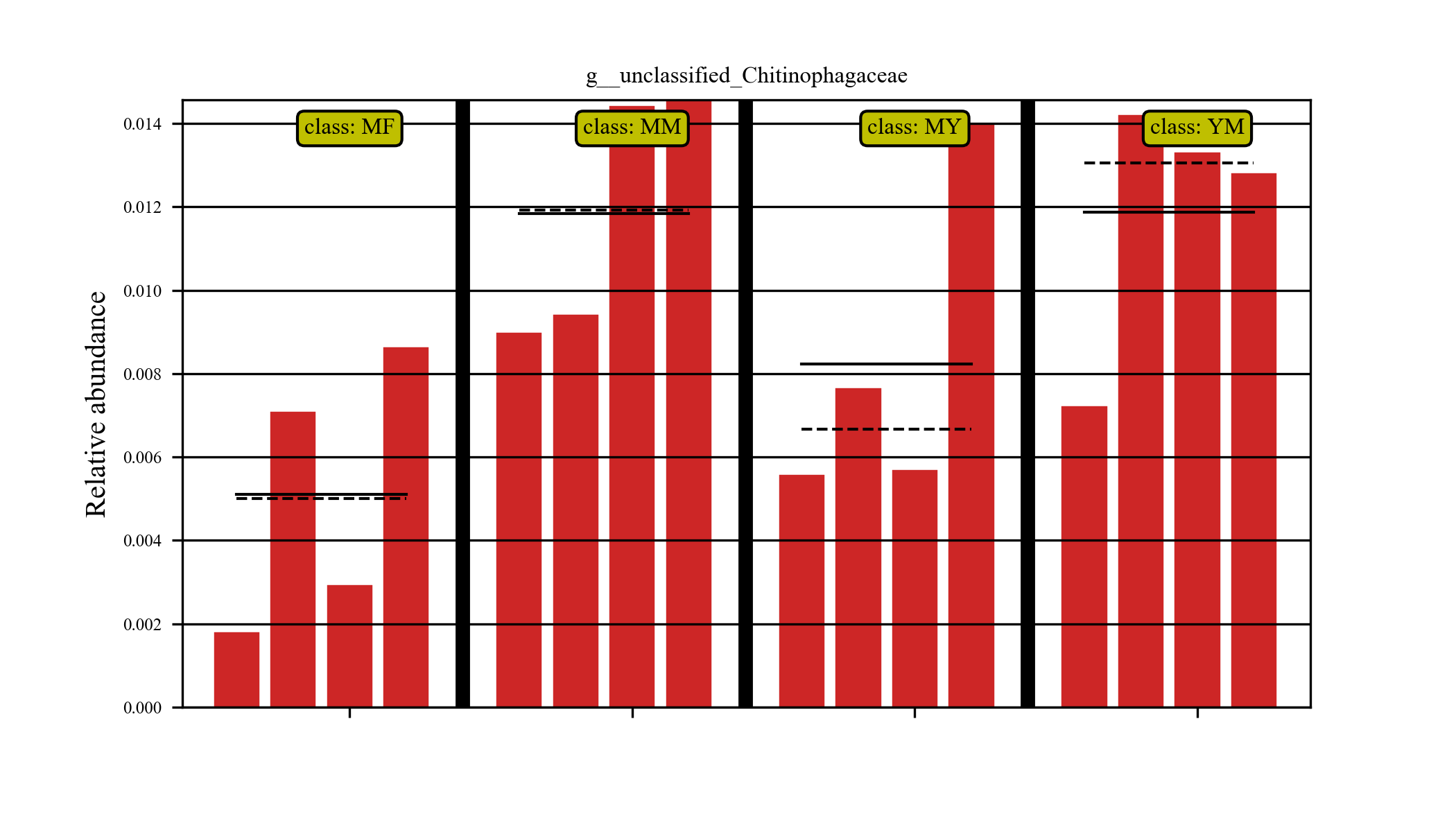

Supplement: Supplementary file 1 [file Data_Sheet_1.ZIP › Supplementary figure 1. bacterial biomarker community/1_g__unclassified_Chitinophagaceae.png]

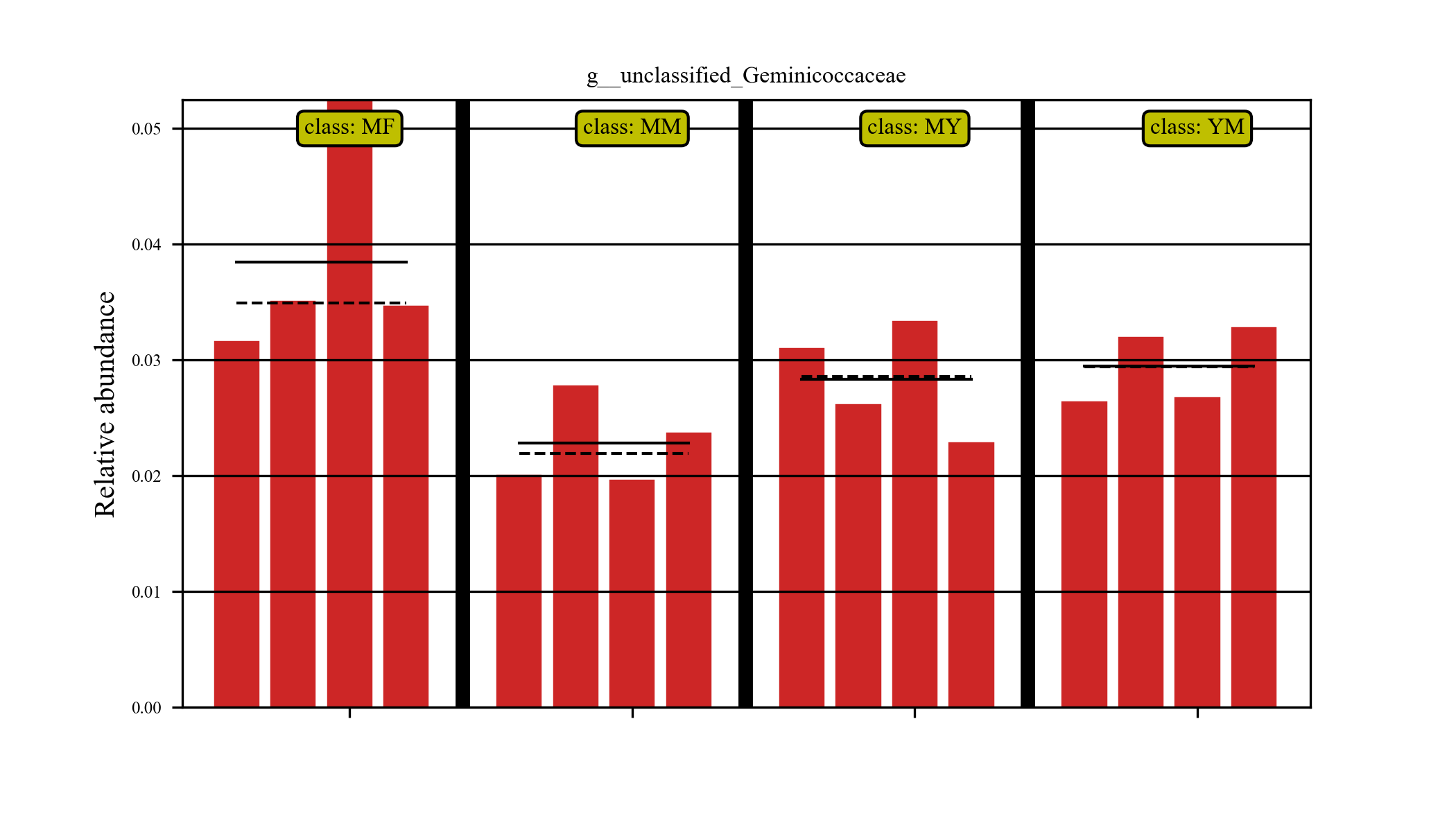

Supplement: Supplementary file 1 [file Data_Sheet_1.ZIP › Supplementary figure 1. bacterial biomarker community/1_g__unclassified_Geminicoccaceae.png]

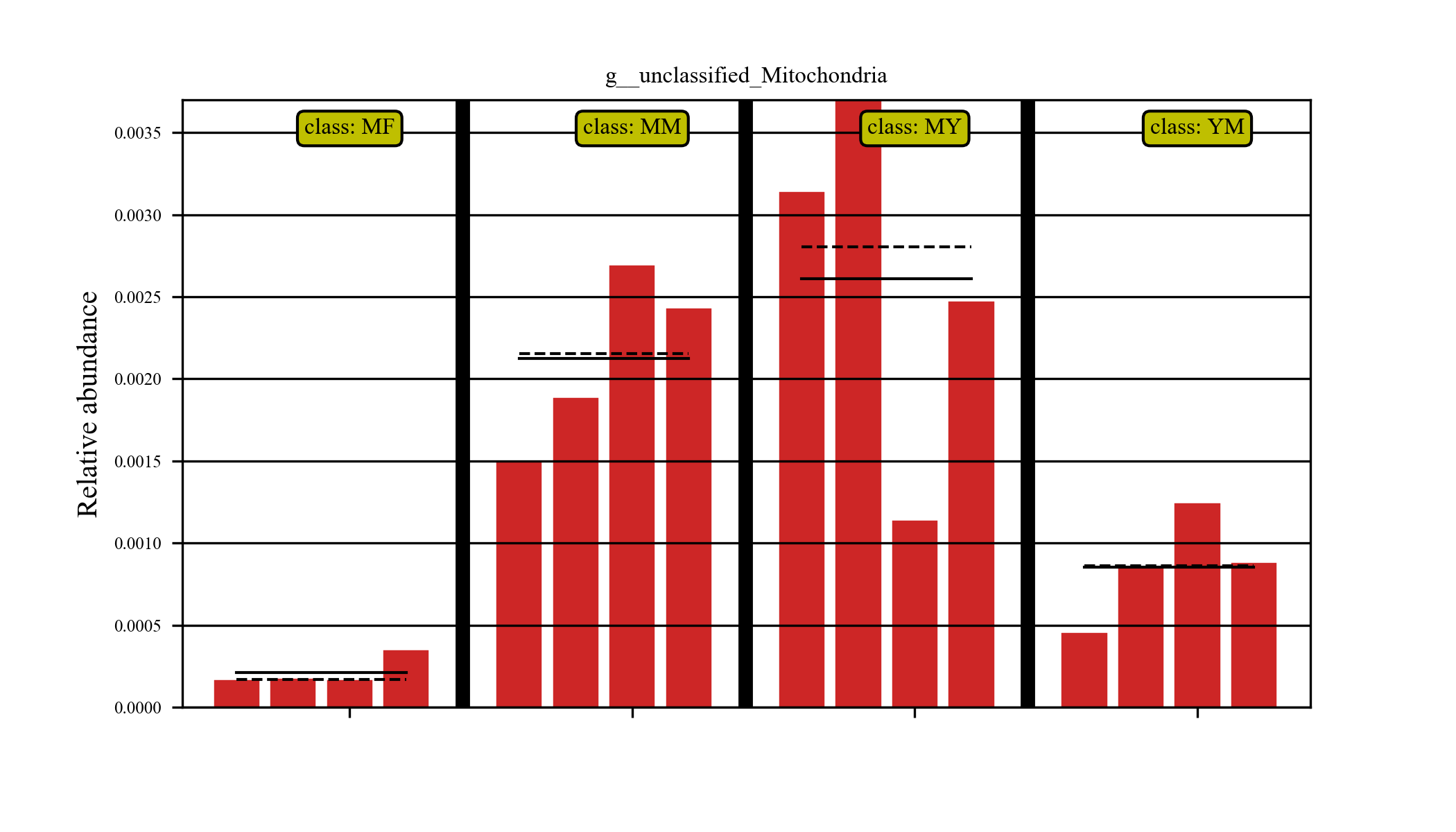

Supplement: Supplementary file 1 [file Data_Sheet_1.ZIP › Supplementary figure 1. bacterial biomarker community/1_g__unclassified_Mitochondria.png]

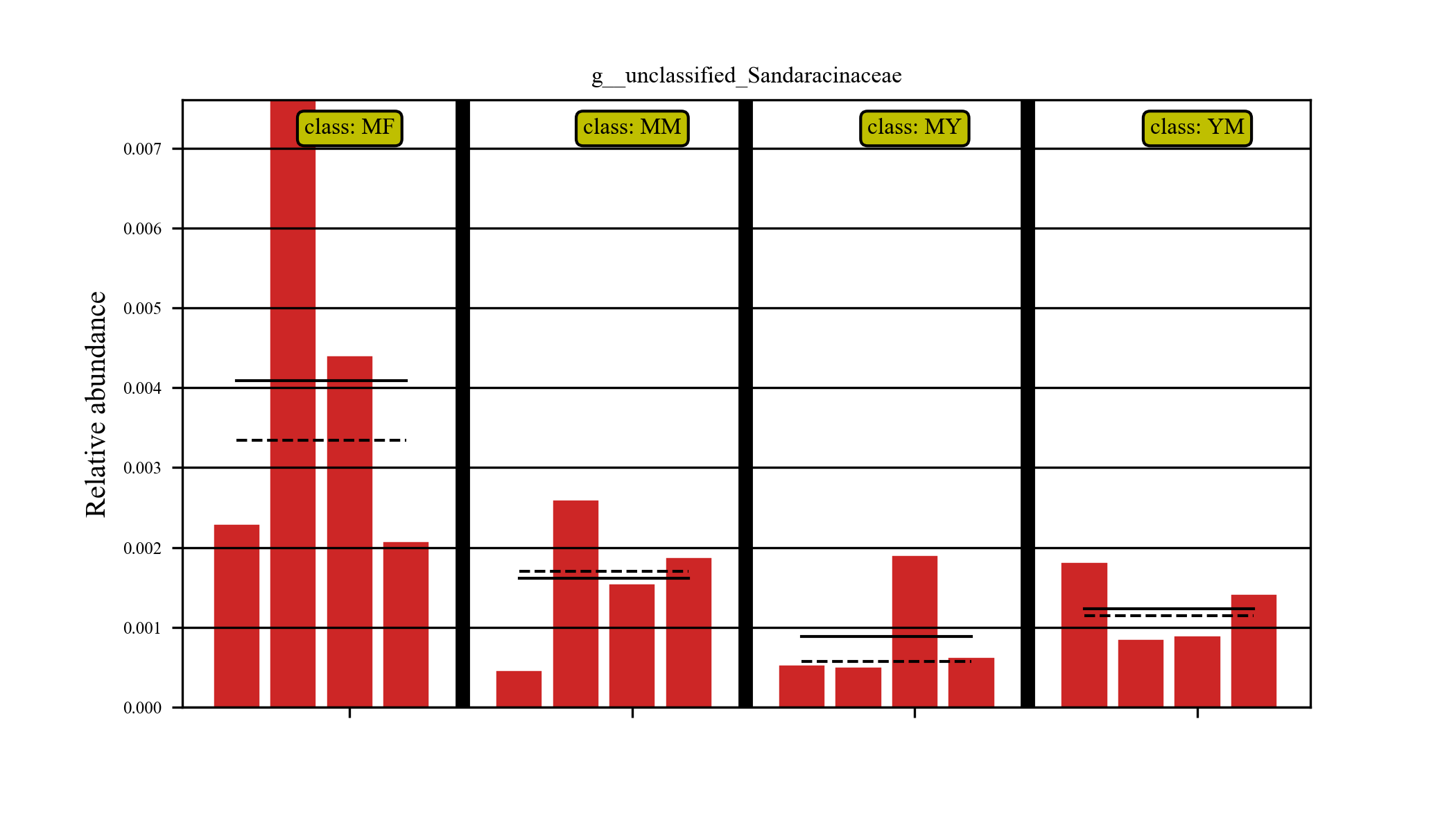

Supplement: Supplementary file 1 [file Data_Sheet_1.ZIP › Supplementary figure 1. bacterial biomarker community/1_g__unclassified_Sandaracinaceae.png]

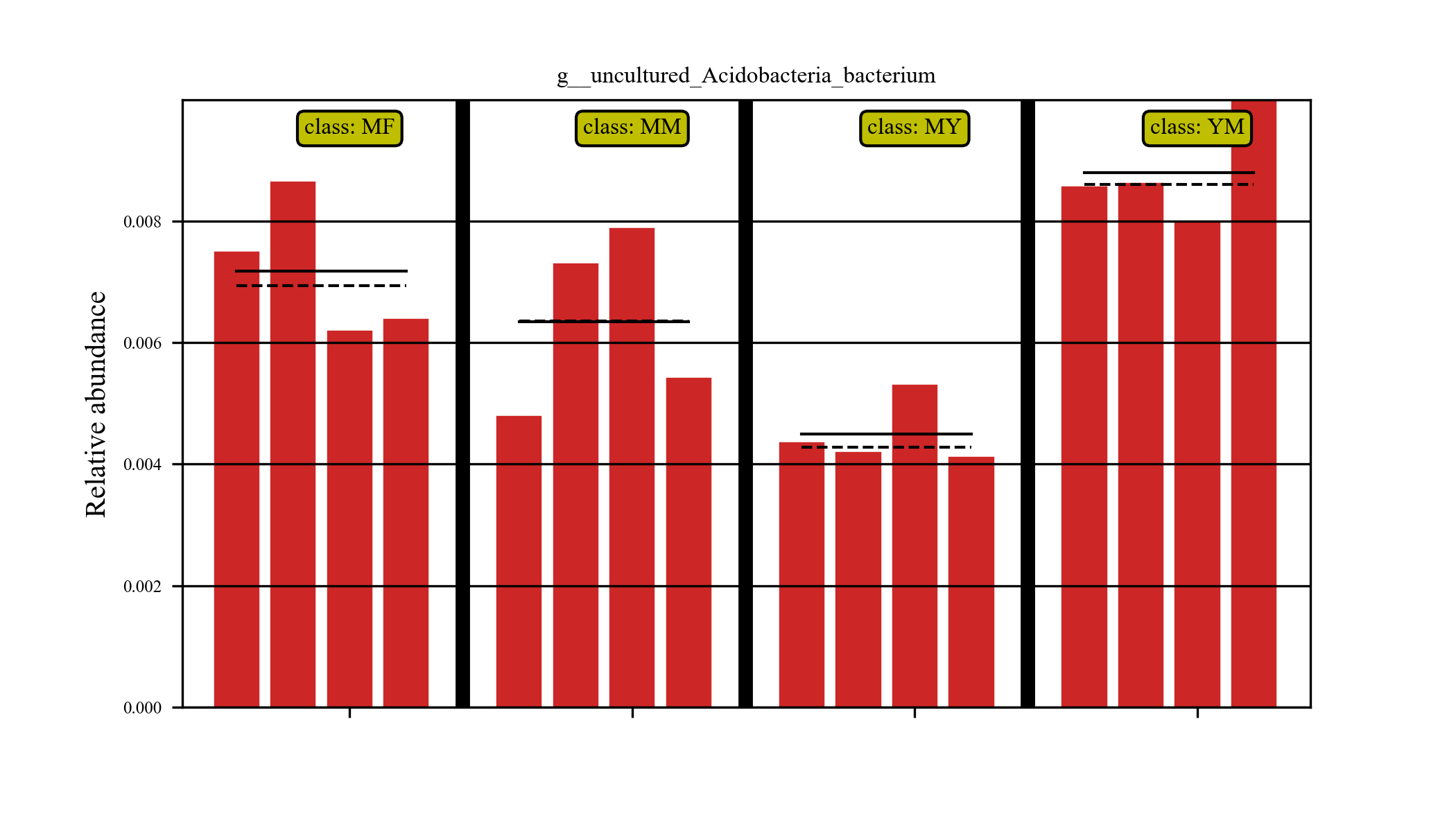

Supplement: Supplementary file 1 [file Data_Sheet_1.ZIP › Supplementary figure 1. bacterial biomarker community/1_g__uncultured_Acidobacteria_bacterium.png]

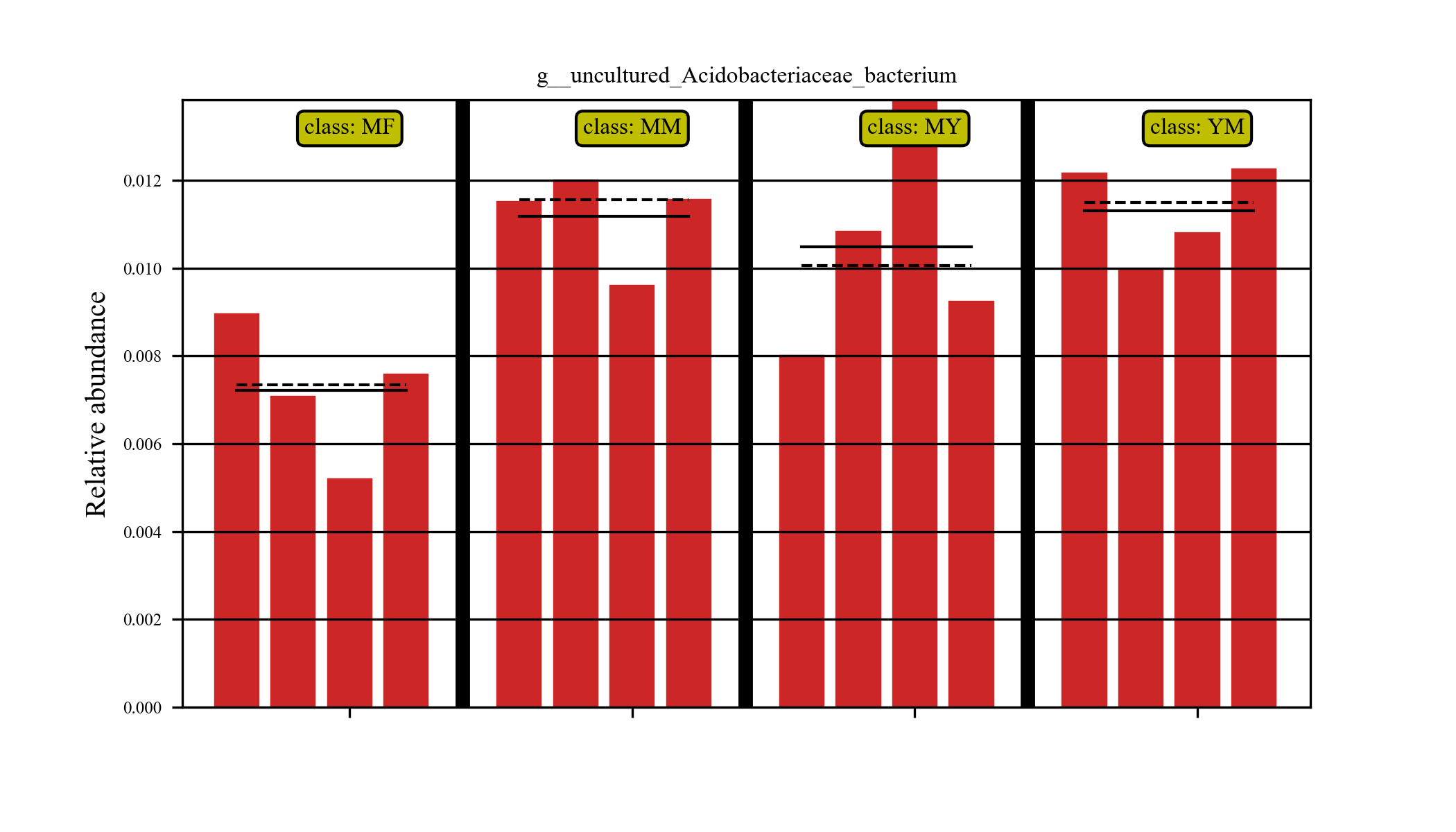

Supplement: Supplementary file 1 [file Data_Sheet_1.ZIP › Supplementary figure 1. bacterial biomarker community/1_g__uncultured_Acidobacteriaceae_bacterium.png]

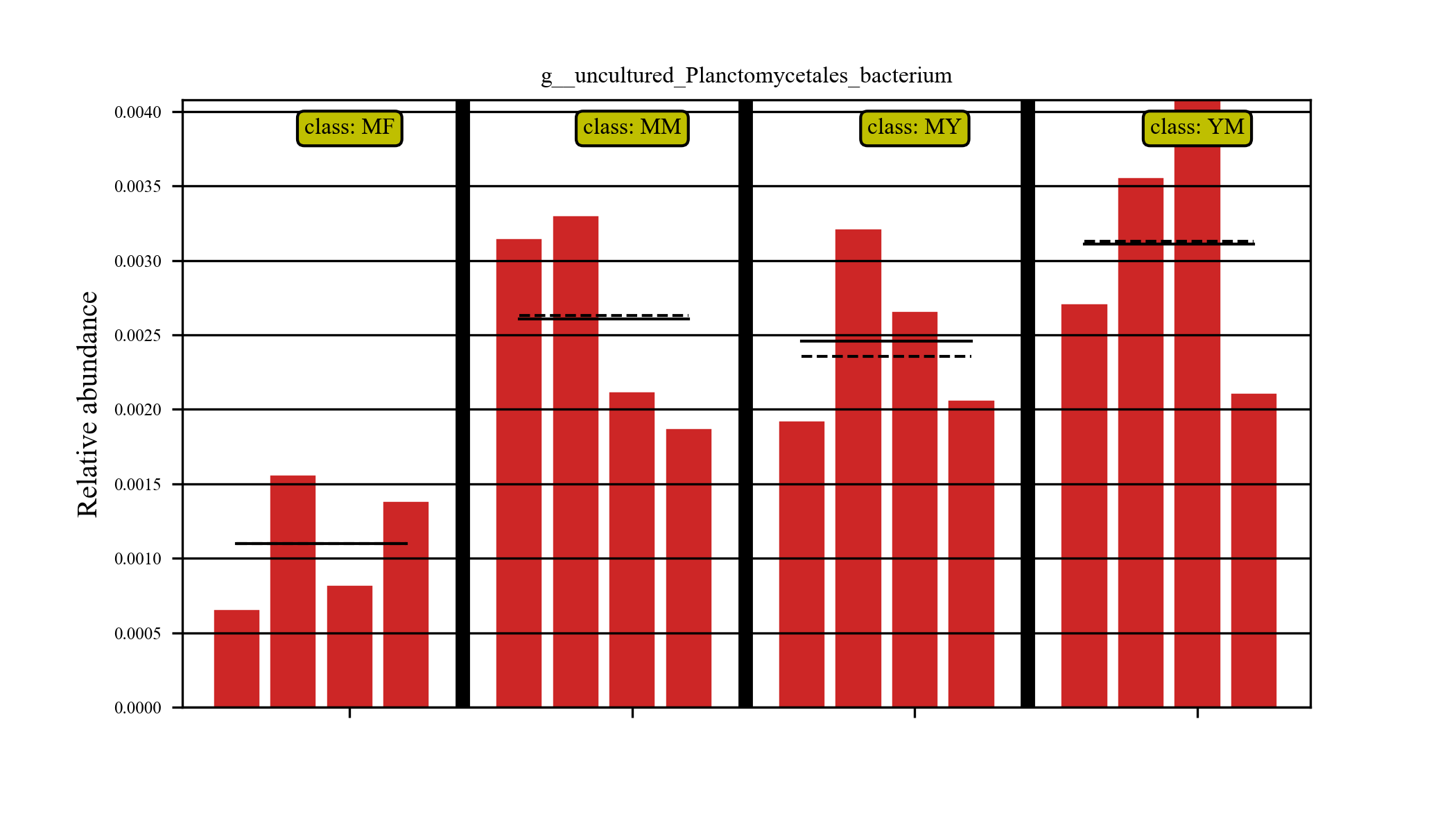

Supplement: Supplementary file 1 [file Data_Sheet_1.ZIP › Supplementary figure 1. bacterial biomarker community/1_g__uncultured_Planctomycetales_bacterium.png]

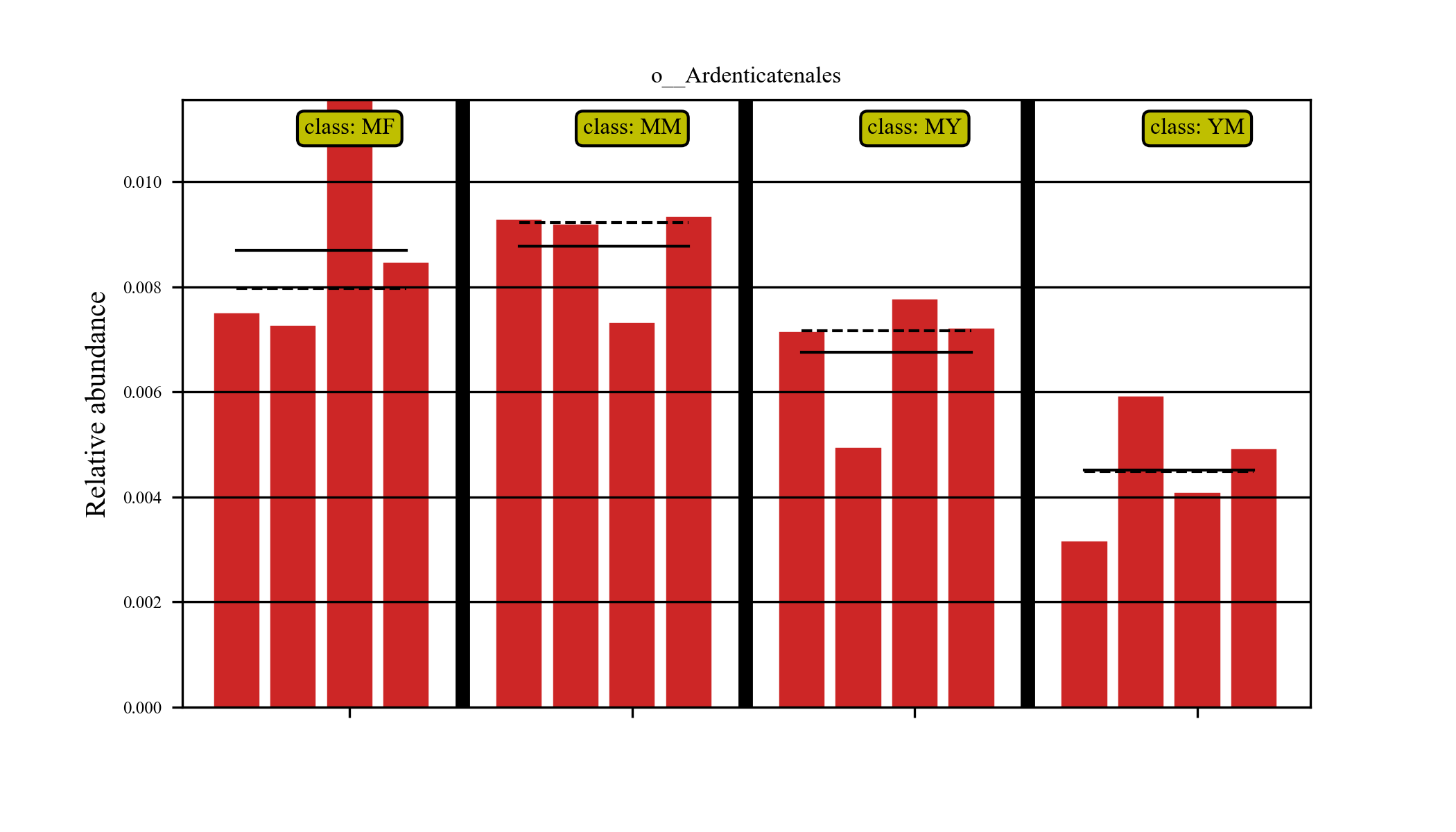

Supplement: Supplementary file 1 [file Data_Sheet_1.ZIP › Supplementary figure 1. bacterial biomarker community/1_o__Ardenticatenales.png]

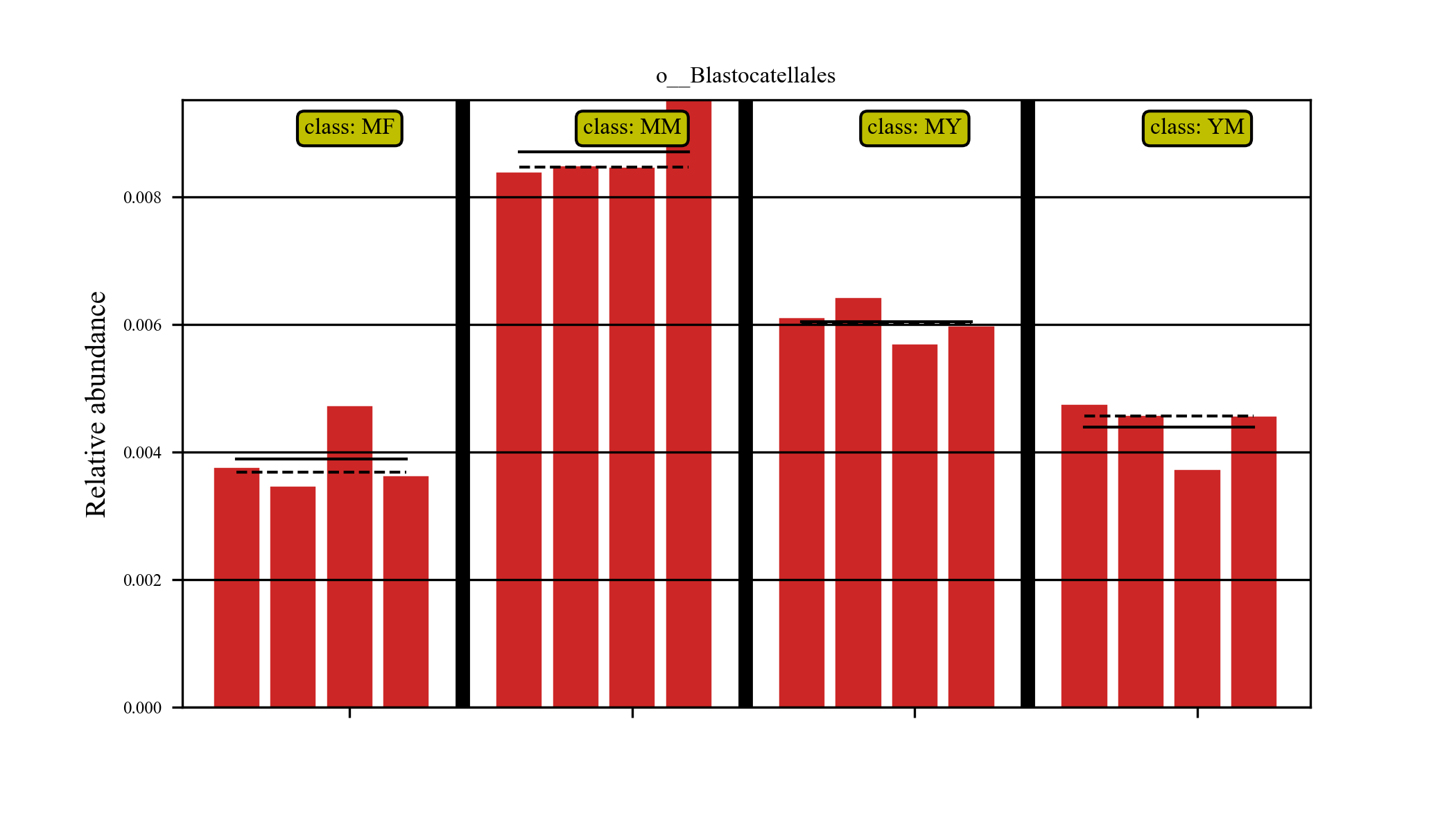

Supplement: Supplementary file 1 [file Data_Sheet_1.ZIP › Supplementary figure 1. bacterial biomarker community/1_o__Blastocatellales.png]

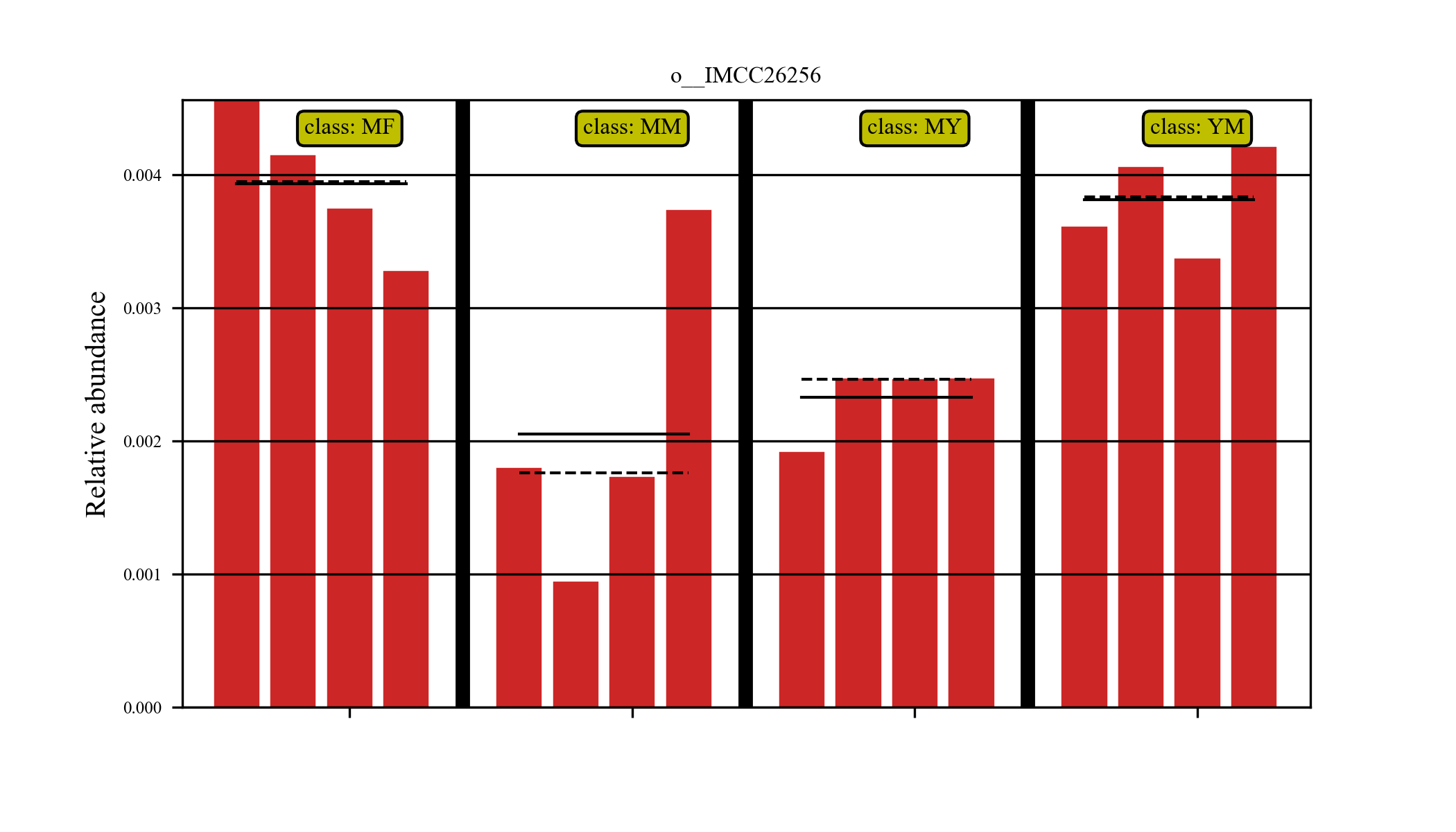

Supplement: Supplementary file 1 [file Data_Sheet_1.ZIP › Supplementary figure 1. bacterial biomarker community/1_o__IMCC26256.png]

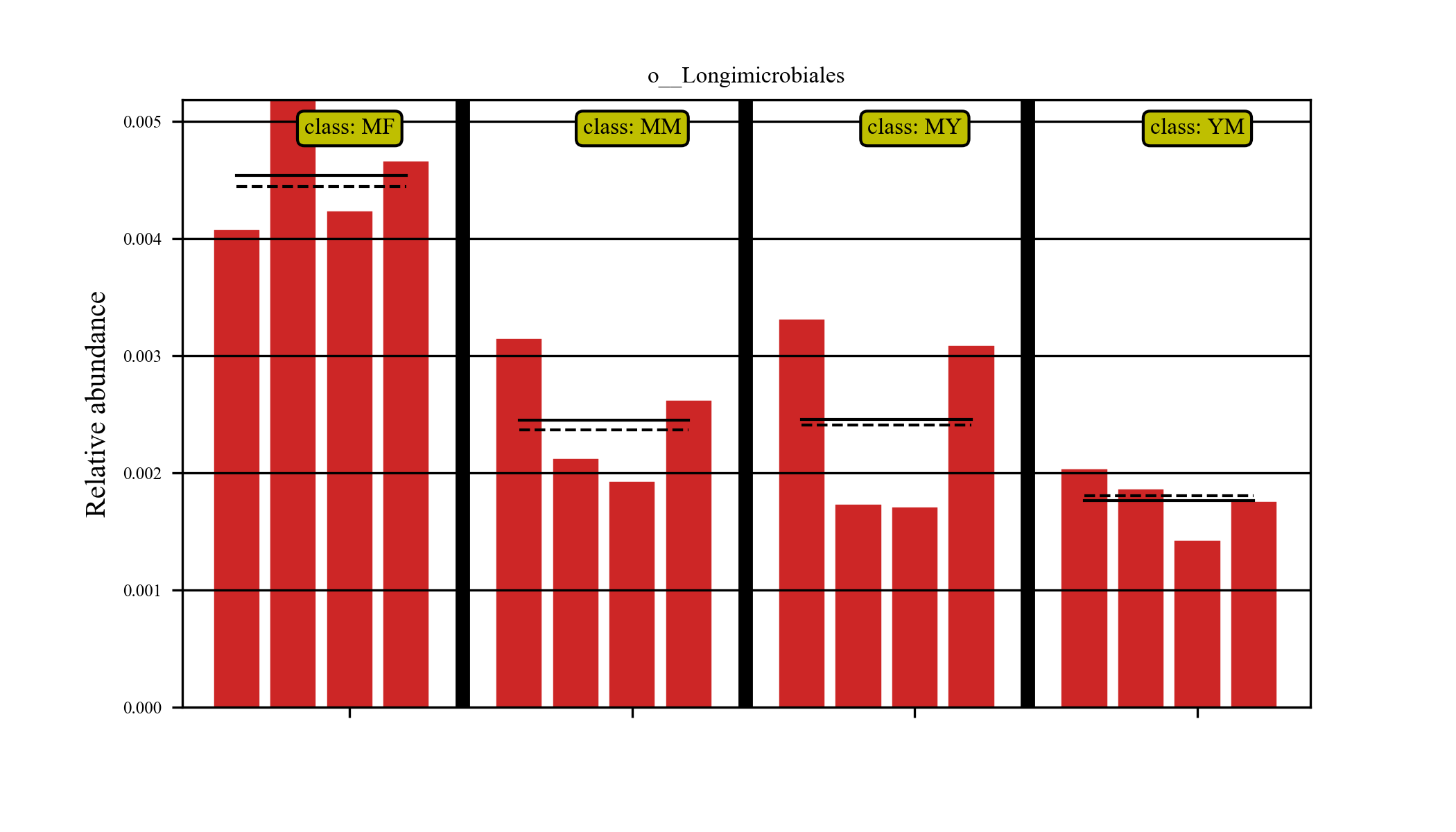

Supplement: Supplementary file 1 [file Data_Sheet_1.ZIP › Supplementary figure 1. bacterial biomarker community/1_o__Longimicrobiales.png]

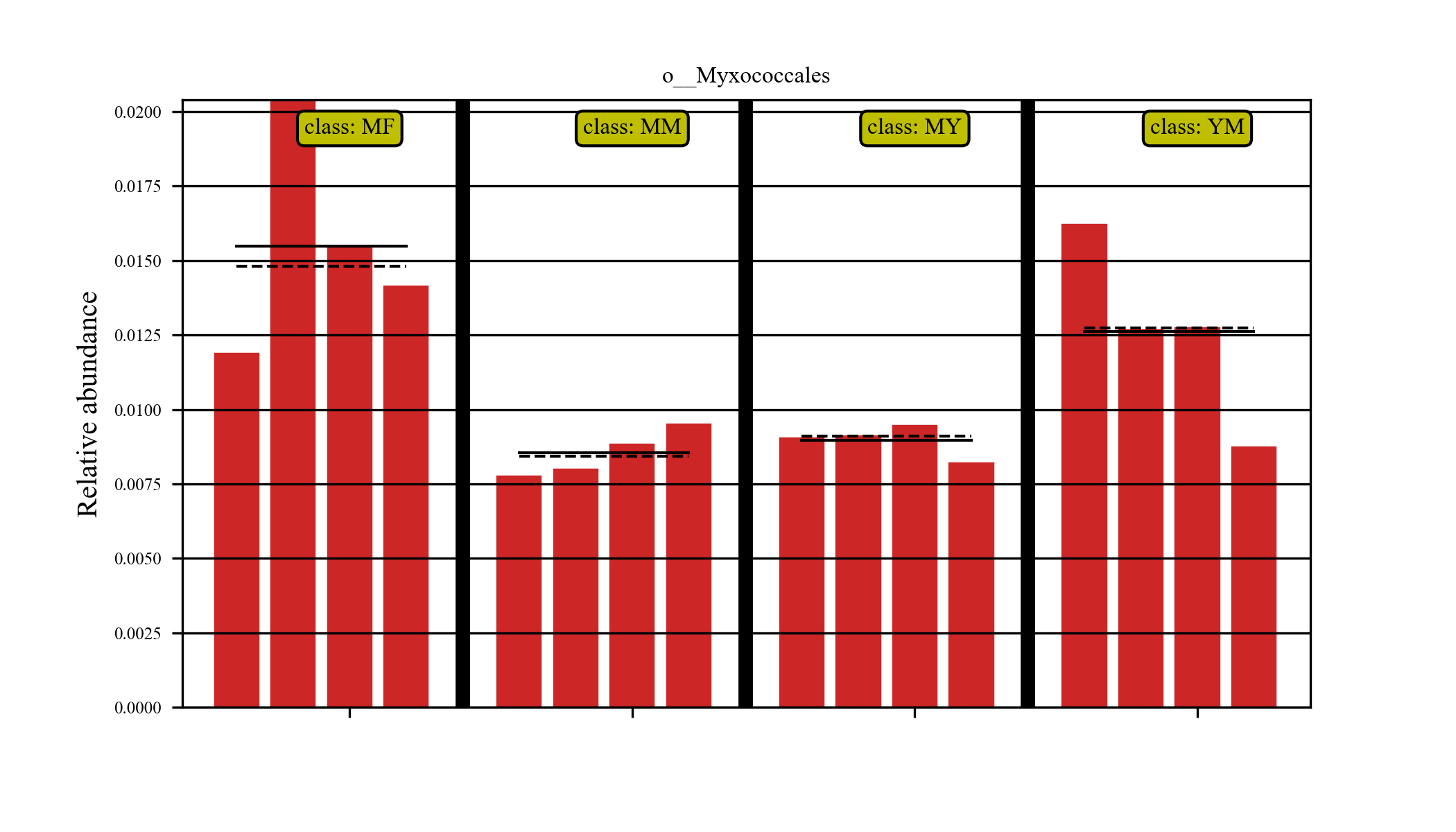

Supplement: Supplementary file 1 [file Data_Sheet_1.ZIP › Supplementary figure 1. bacterial biomarker community/1_o__Myxococcales.png]

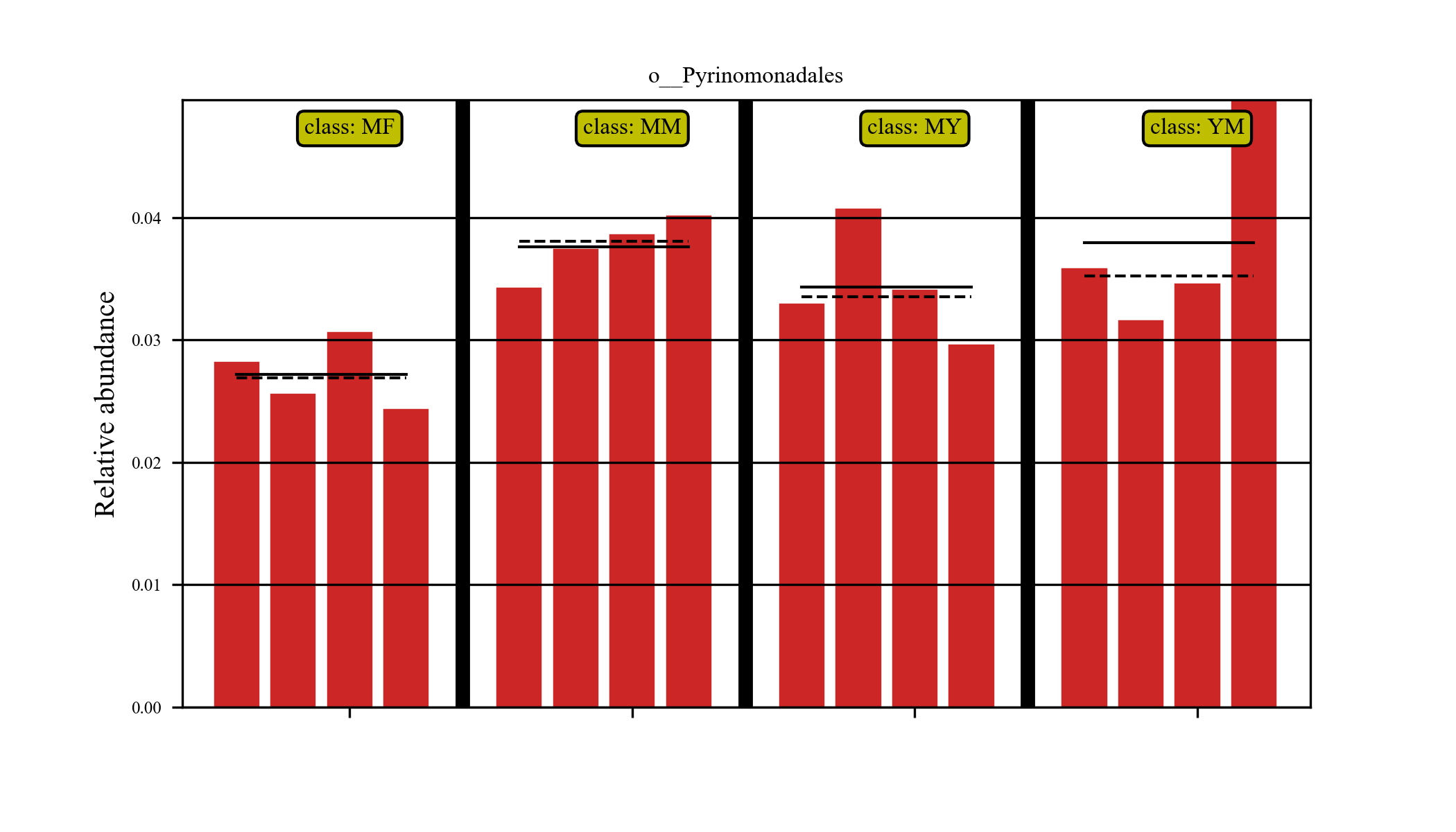

Supplement: Supplementary file 1 [file Data_Sheet_1.ZIP › Supplementary figure 1. bacterial biomarker community/1_o__Pyrinomonadales.png]

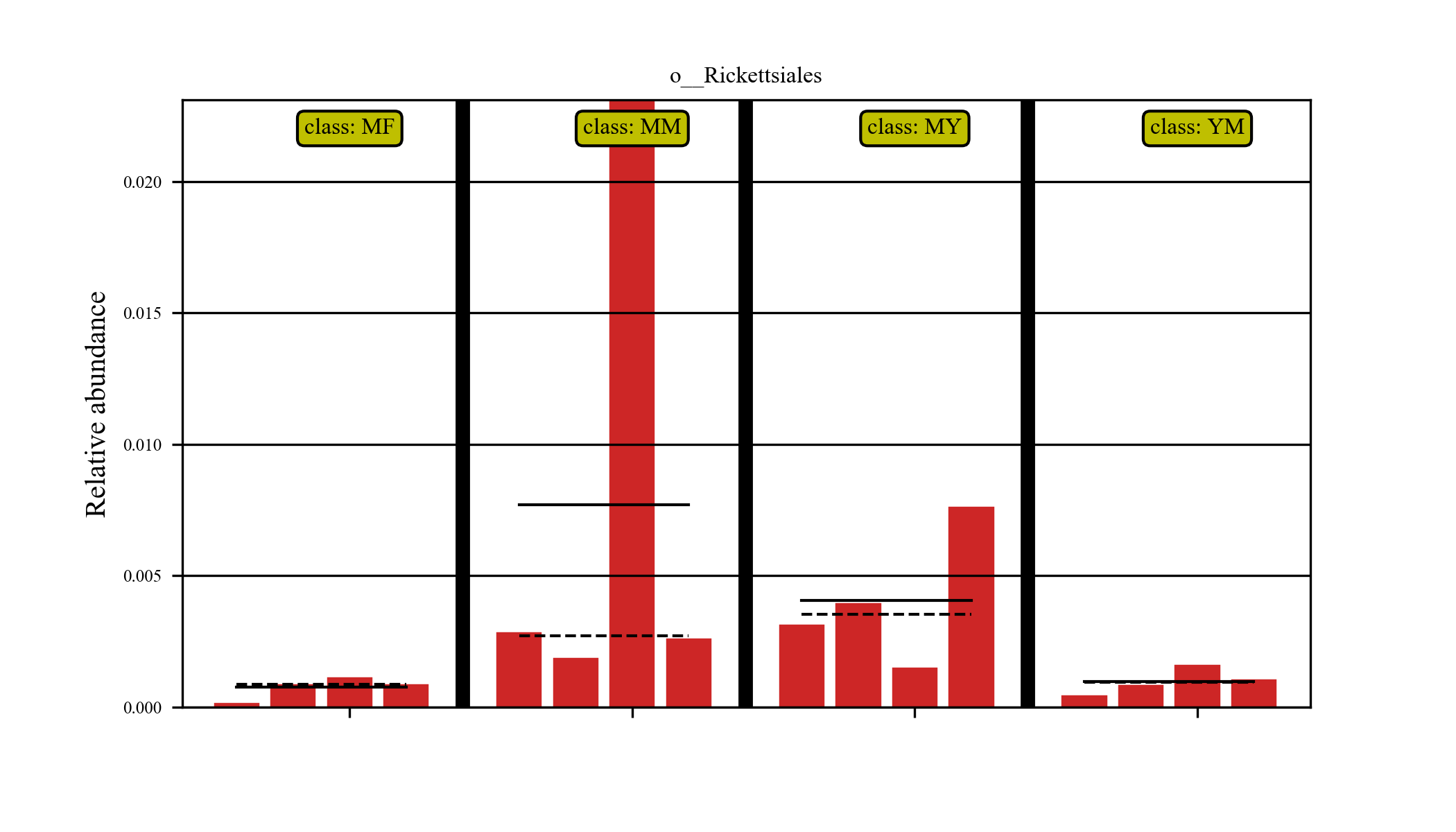

Supplement: Supplementary file 1 [file Data_Sheet_1.ZIP › Supplementary figure 1. bacterial biomarker community/1_o__Rickettsiales.png]

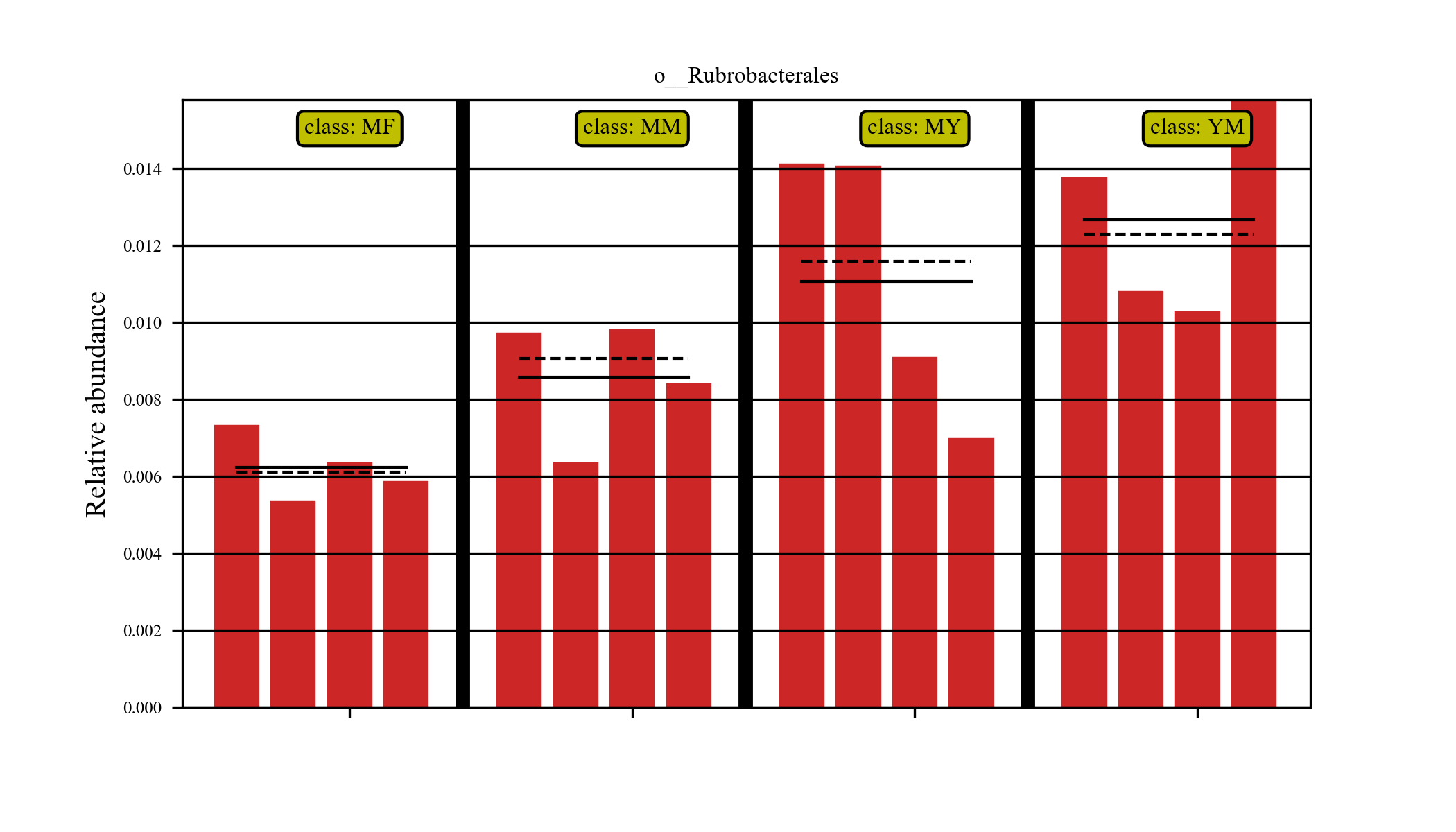

Supplement: Supplementary file 1 [file Data_Sheet_1.ZIP › Supplementary figure 1. bacterial biomarker community/1_o__Rubrobacterales.png]

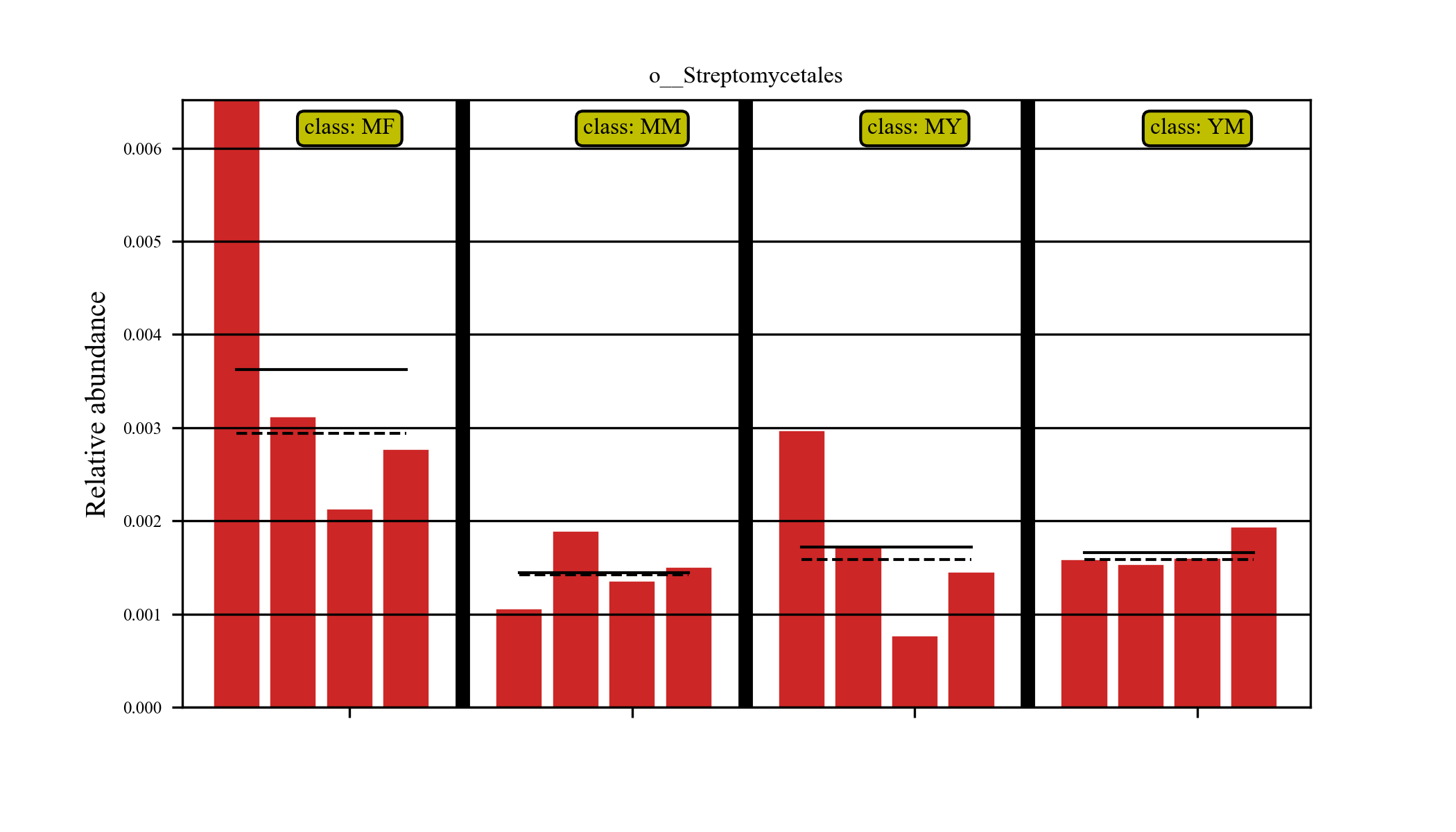

Supplement: Supplementary file 1 [file Data_Sheet_1.ZIP › Supplementary figure 1. bacterial biomarker community/1_o__Streptomycetales.png]

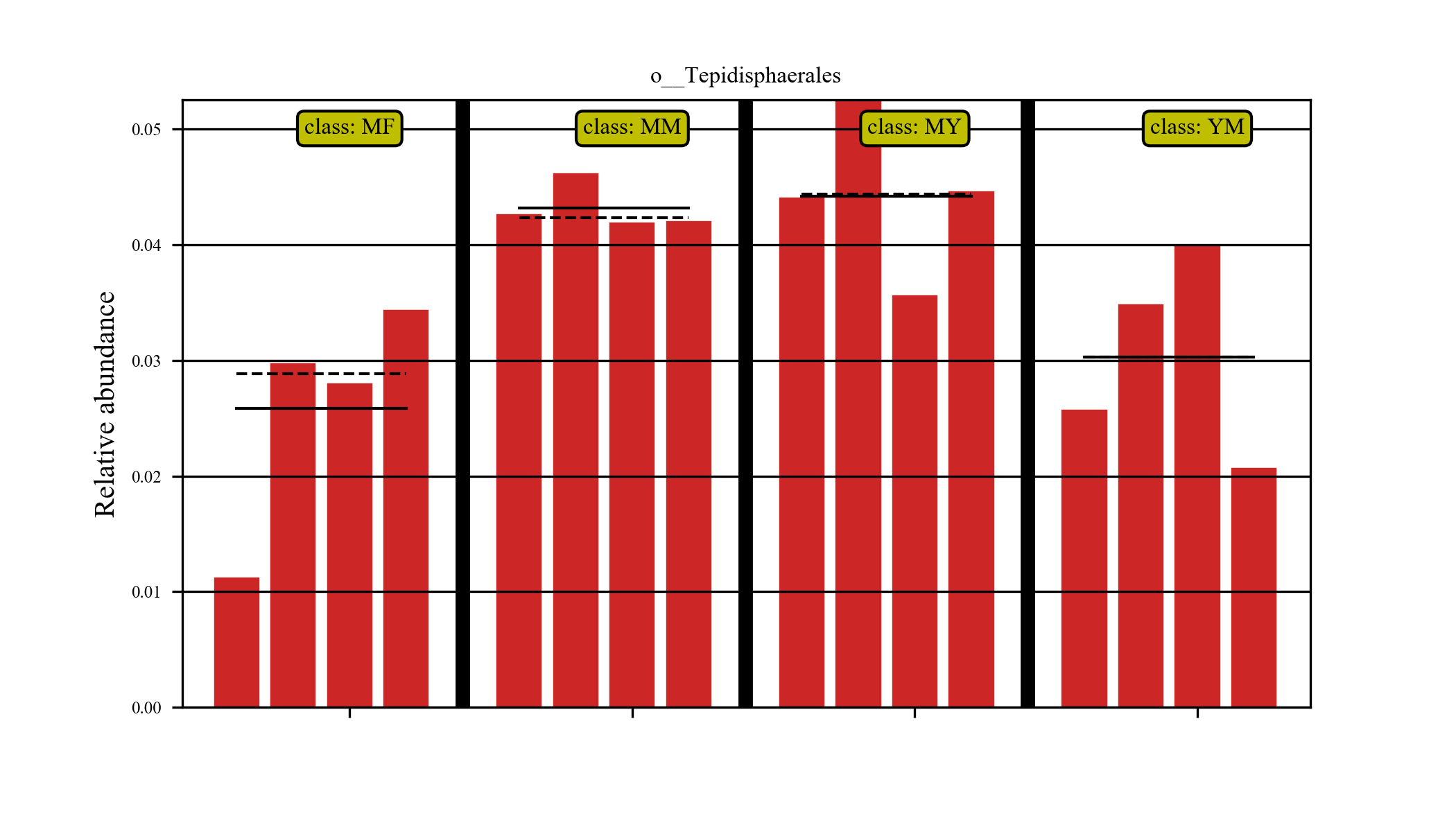

Supplement: Supplementary file 1 [file Data_Sheet_1.ZIP › Supplementary figure 1. bacterial biomarker community/1_o__Tepidisphaerales.png]

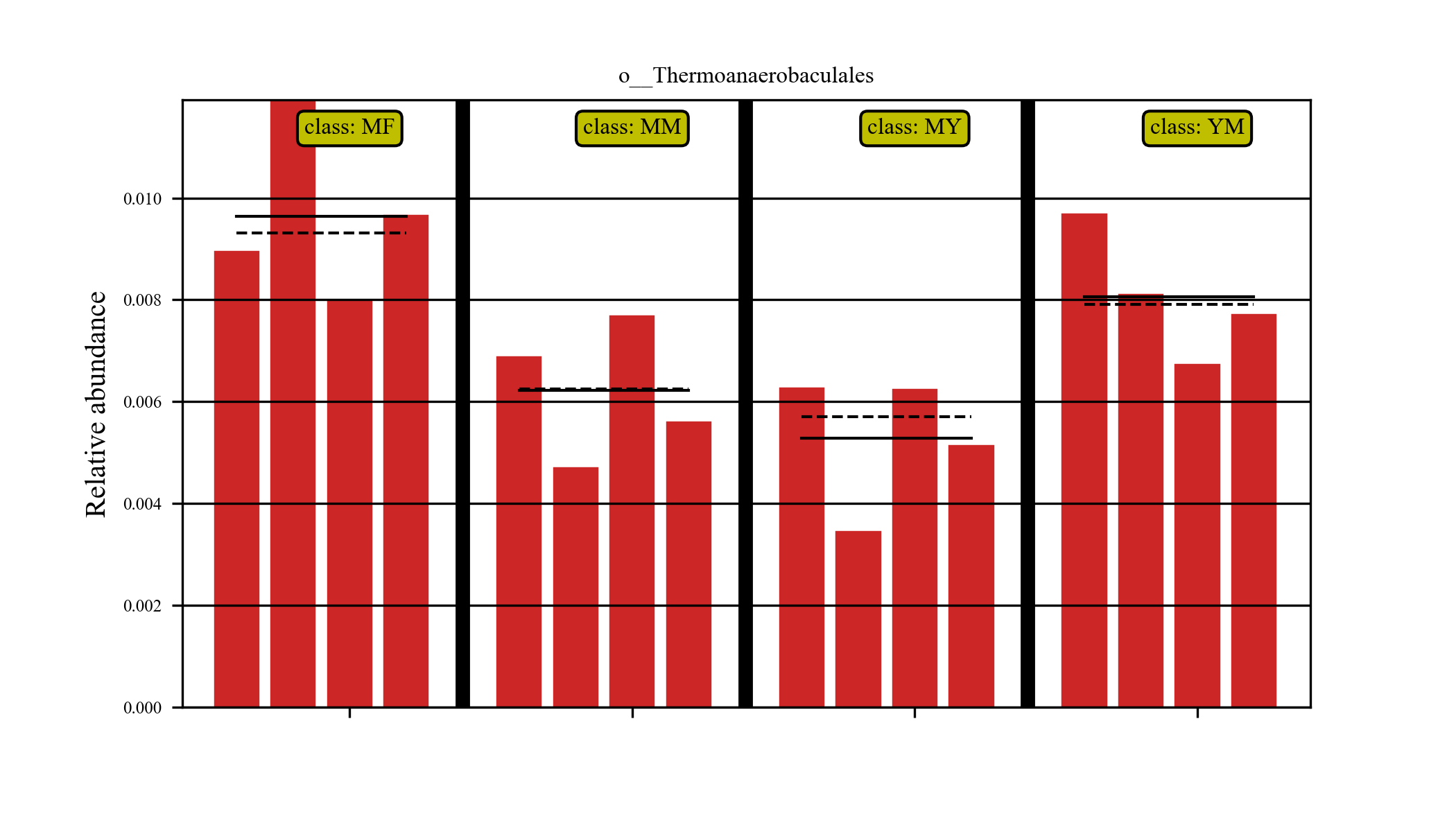

Supplement: Supplementary file 1 [file Data_Sheet_1.ZIP › Supplementary figure 1. bacterial biomarker community/1_o__Thermoanaerobaculales.png]

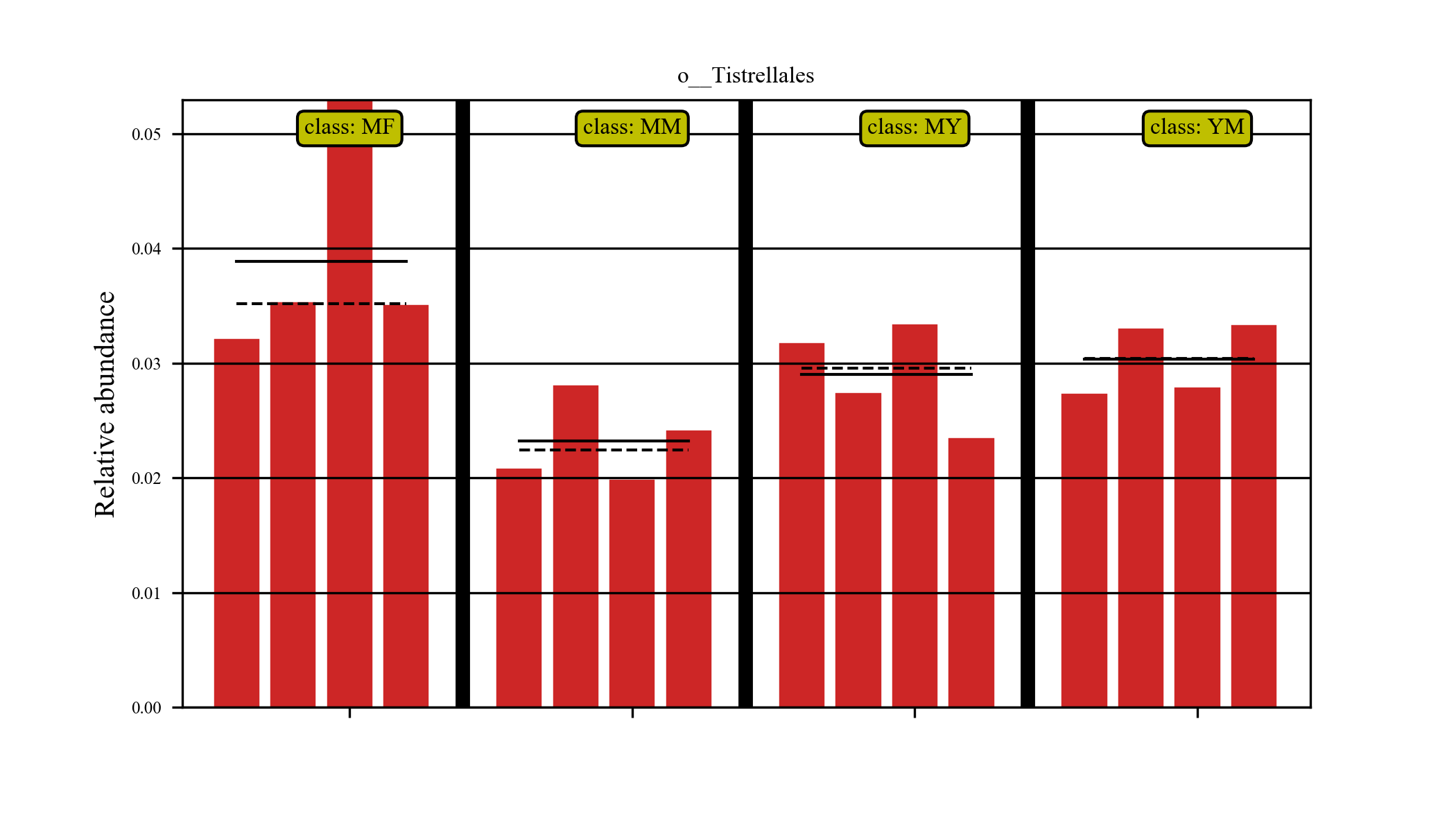

Supplement: Supplementary file 1 [file Data_Sheet_1.ZIP › Supplementary figure 1. bacterial biomarker community/1_o__Tistrellales.png]

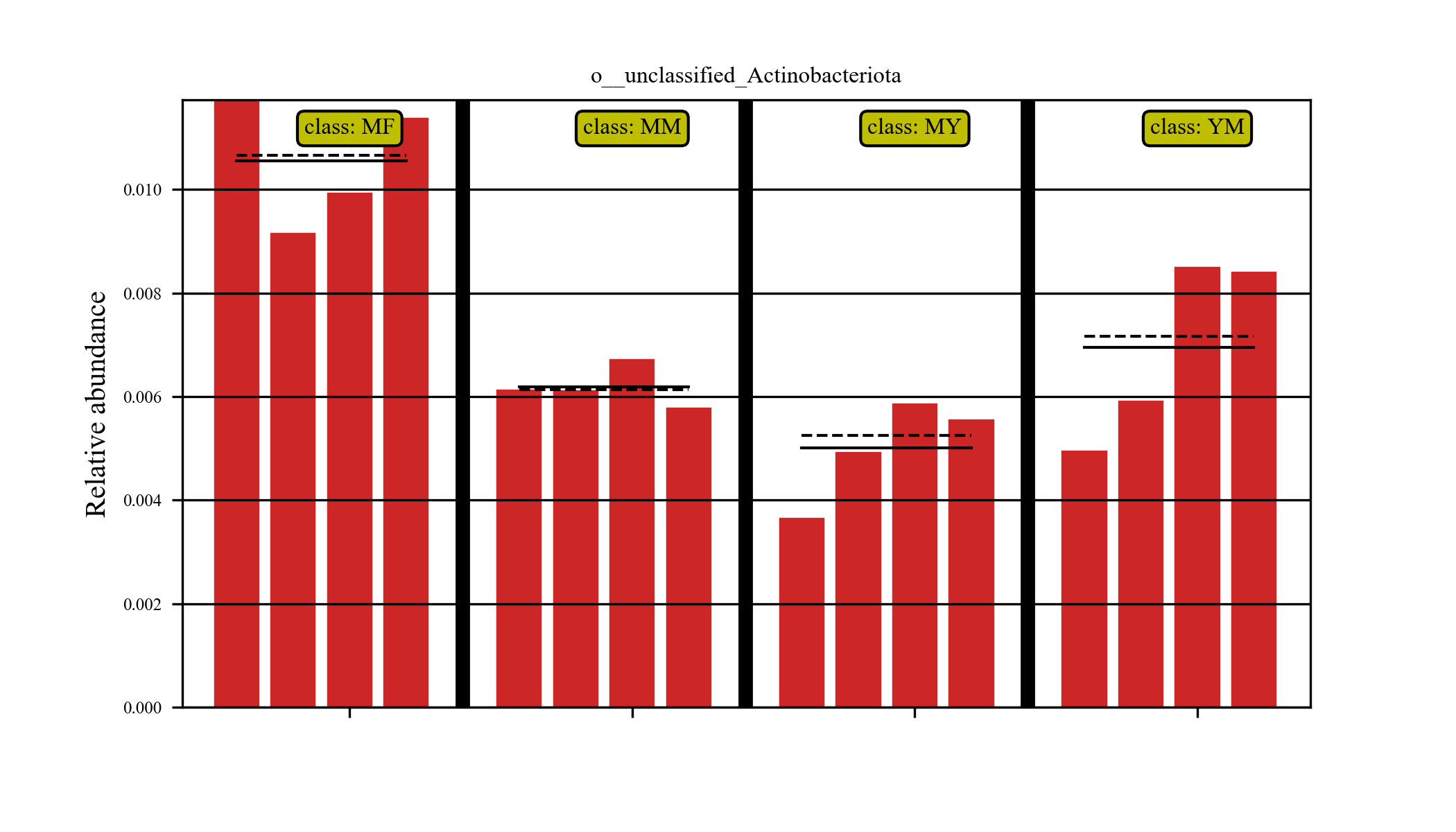

Supplement: Supplementary file 1 [file Data_Sheet_1.ZIP › Supplementary figure 1. bacterial biomarker community/1_o__unclassified_Actinobacteriota.png]

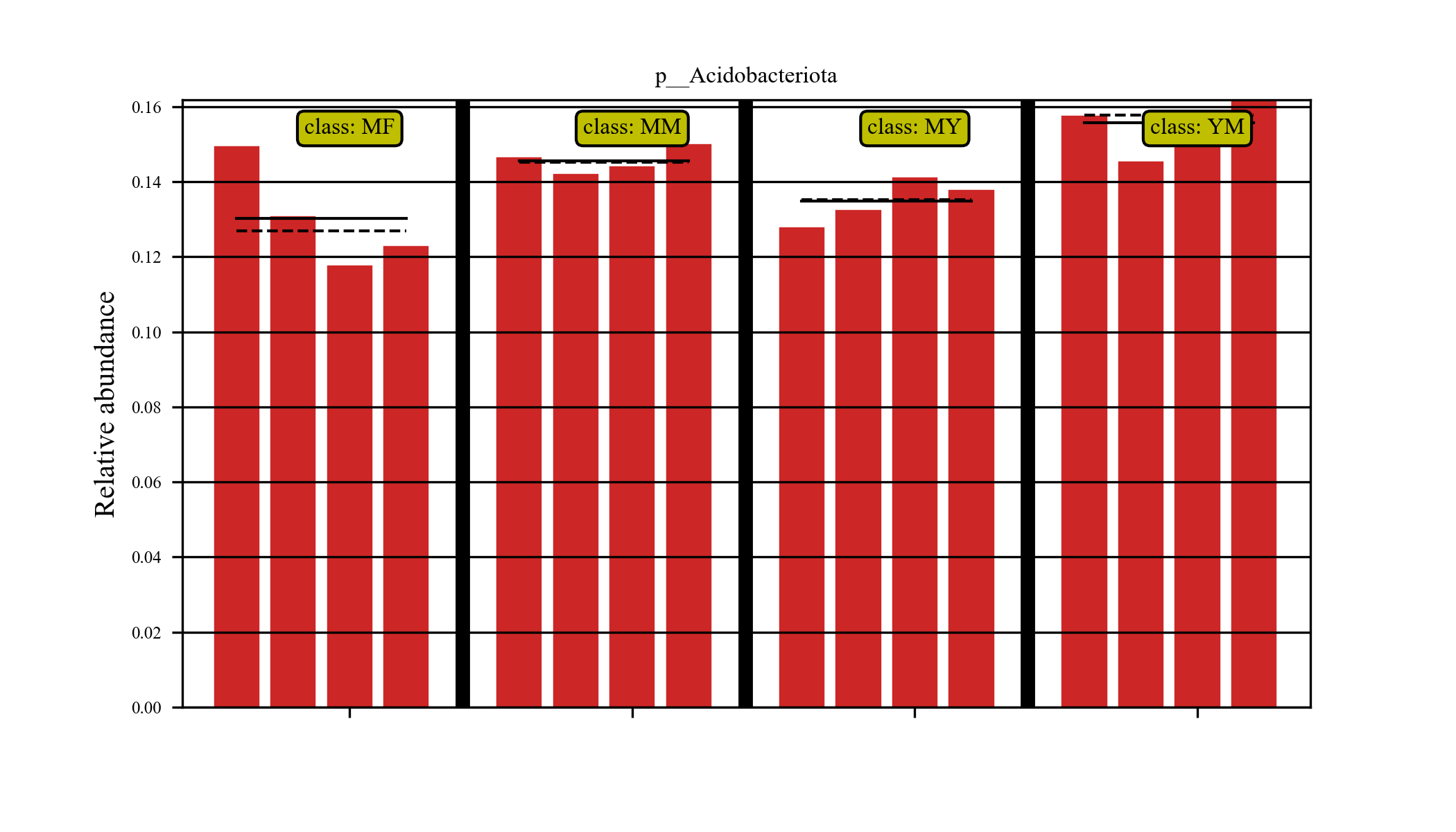

Supplement: Supplementary file 1 [file Data_Sheet_1.ZIP › Supplementary figure 1. bacterial biomarker community/1_p__Acidobacteriota.png]

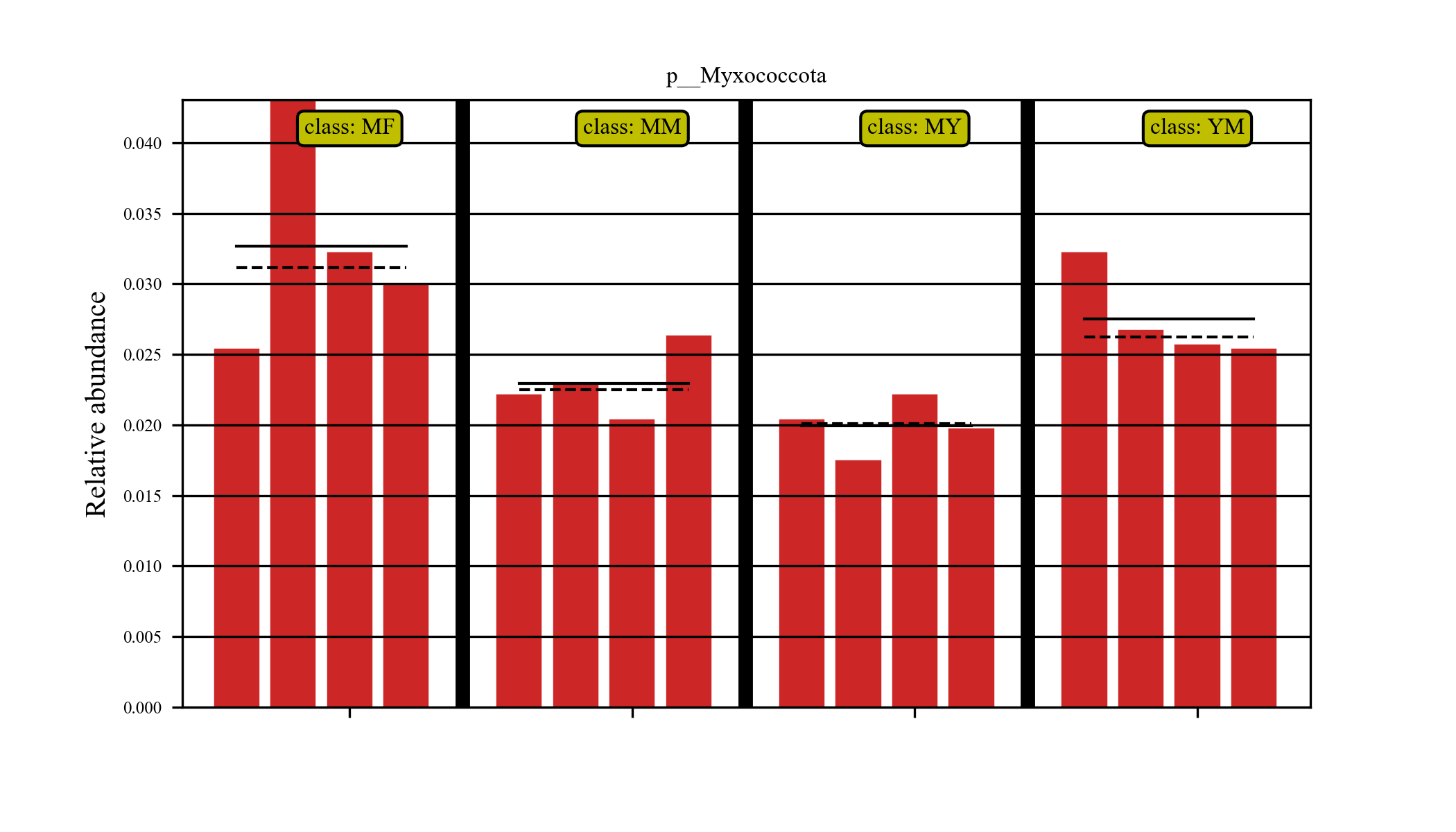

Supplement: Supplementary file 1 [file Data_Sheet_1.ZIP › Supplementary figure 1. bacterial biomarker community/1_p__Myxococcota.png]

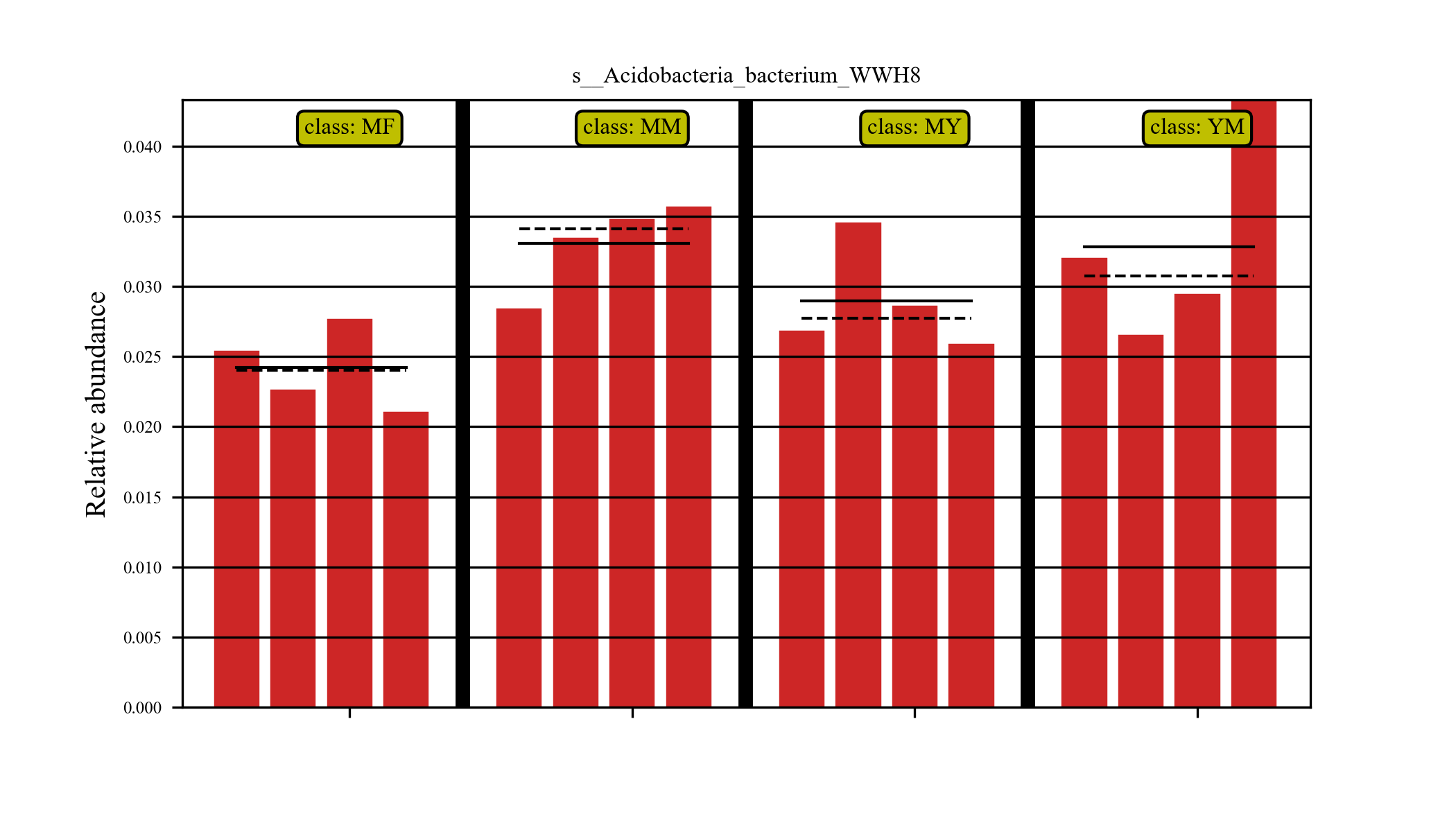

Supplement: Supplementary file 1 [file Data_Sheet_1.ZIP › Supplementary figure 1. bacterial biomarker community/1_s__Acidobacteria_bacterium_WWH8.png]

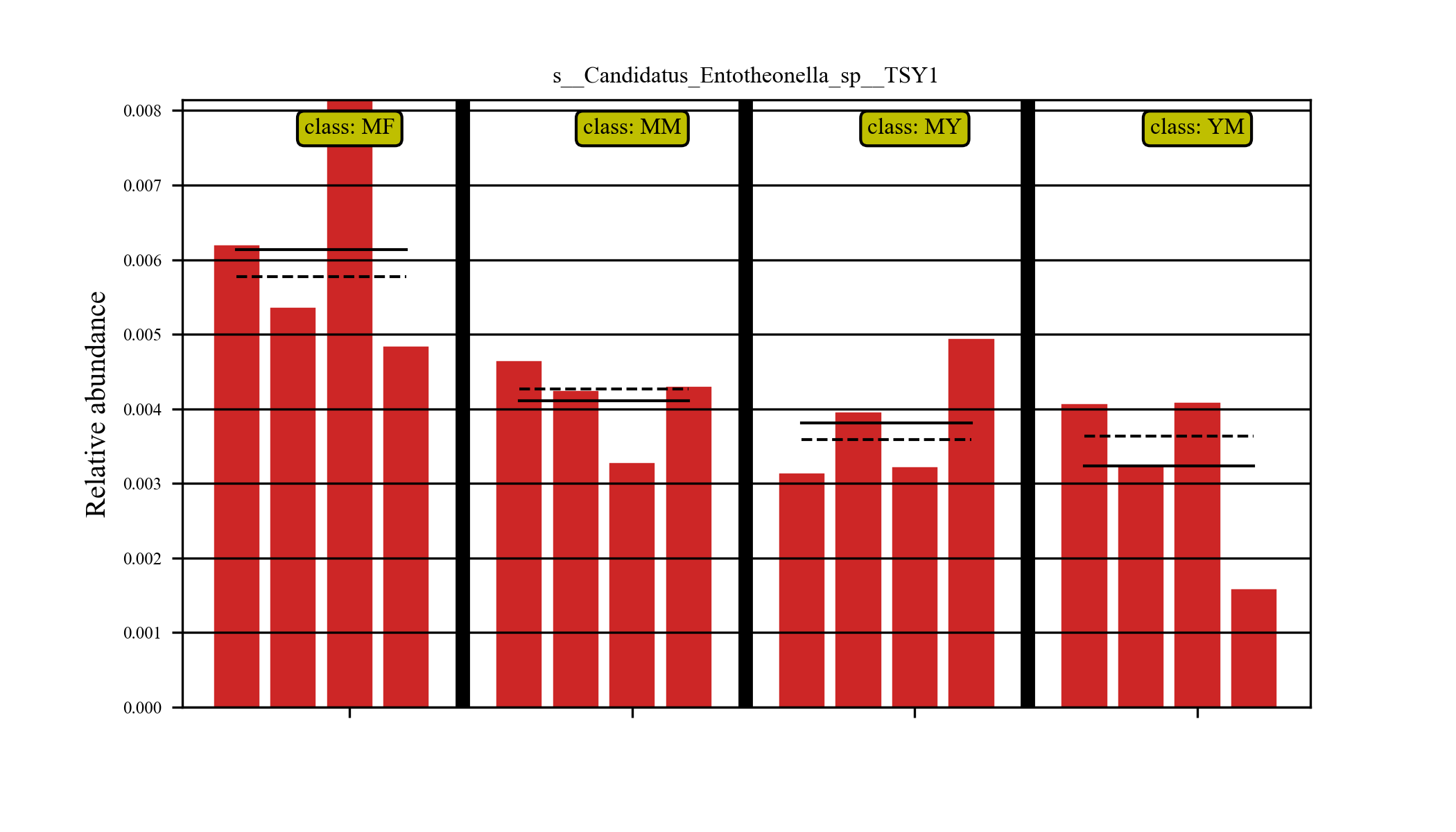

Supplement: Supplementary file 1 [file Data_Sheet_1.ZIP › Supplementary figure 1. bacterial biomarker community/1_s__Candidatus_Entotheonella_sp__TSY1.png]

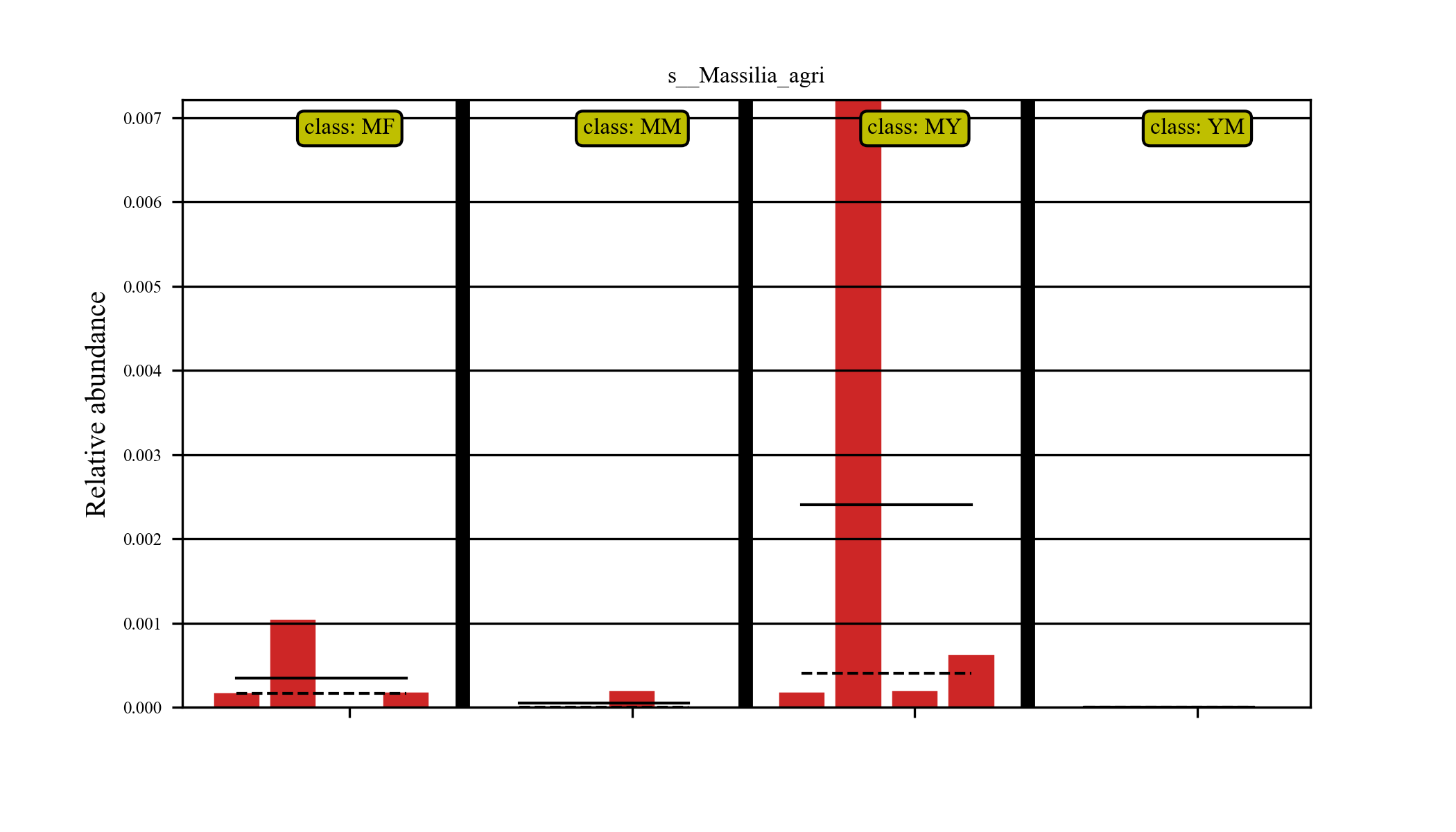

Supplement: Supplementary file 1 [file Data_Sheet_1.ZIP › Supplementary figure 1. bacterial biomarker community/1_s__Massilia_agri.png]

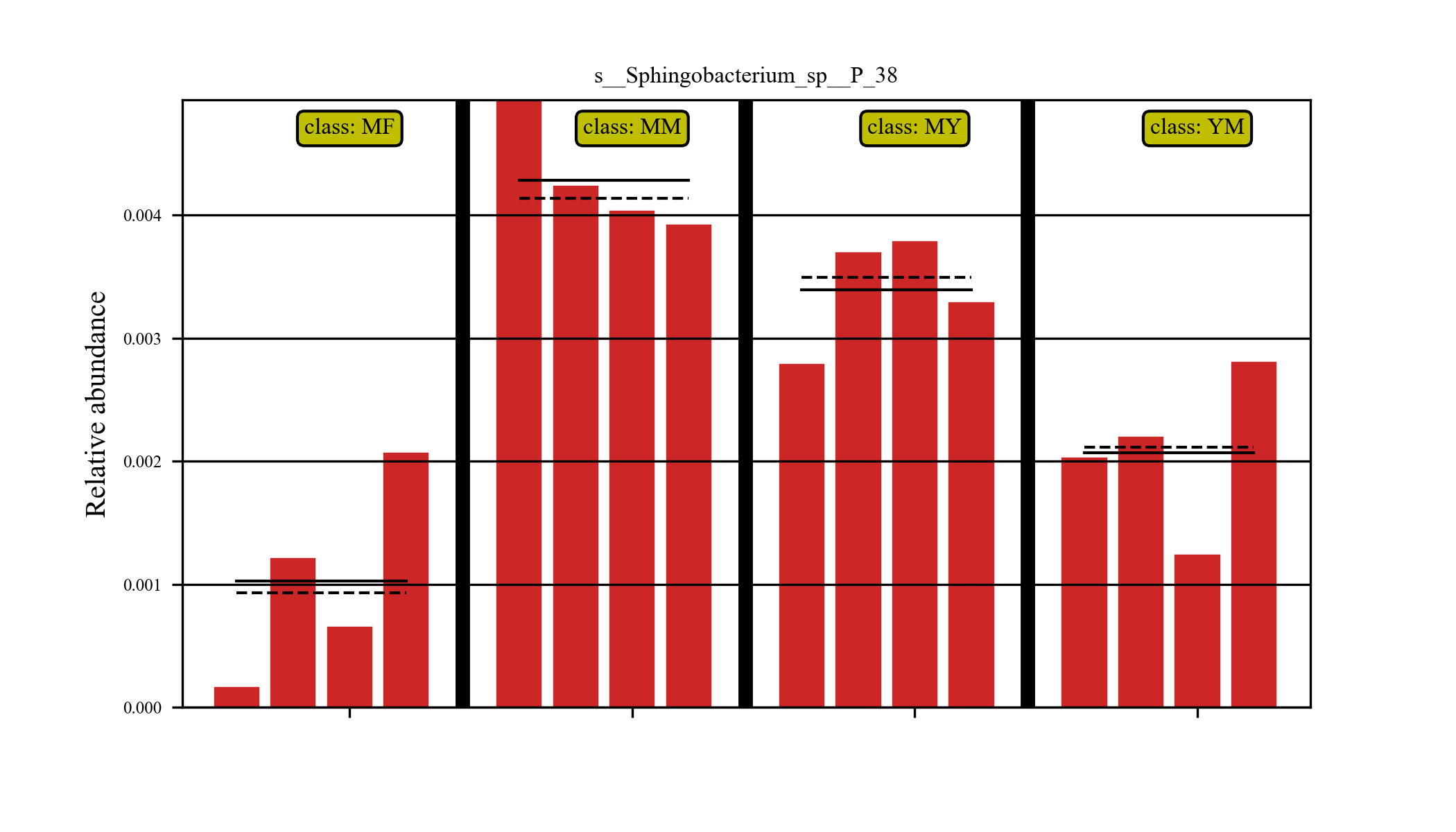

Supplement: Supplementary file 1 [file Data_Sheet_1.ZIP › Supplementary figure 1. bacterial biomarker community/1_s__Sphingobacterium_sp__P_38.png]

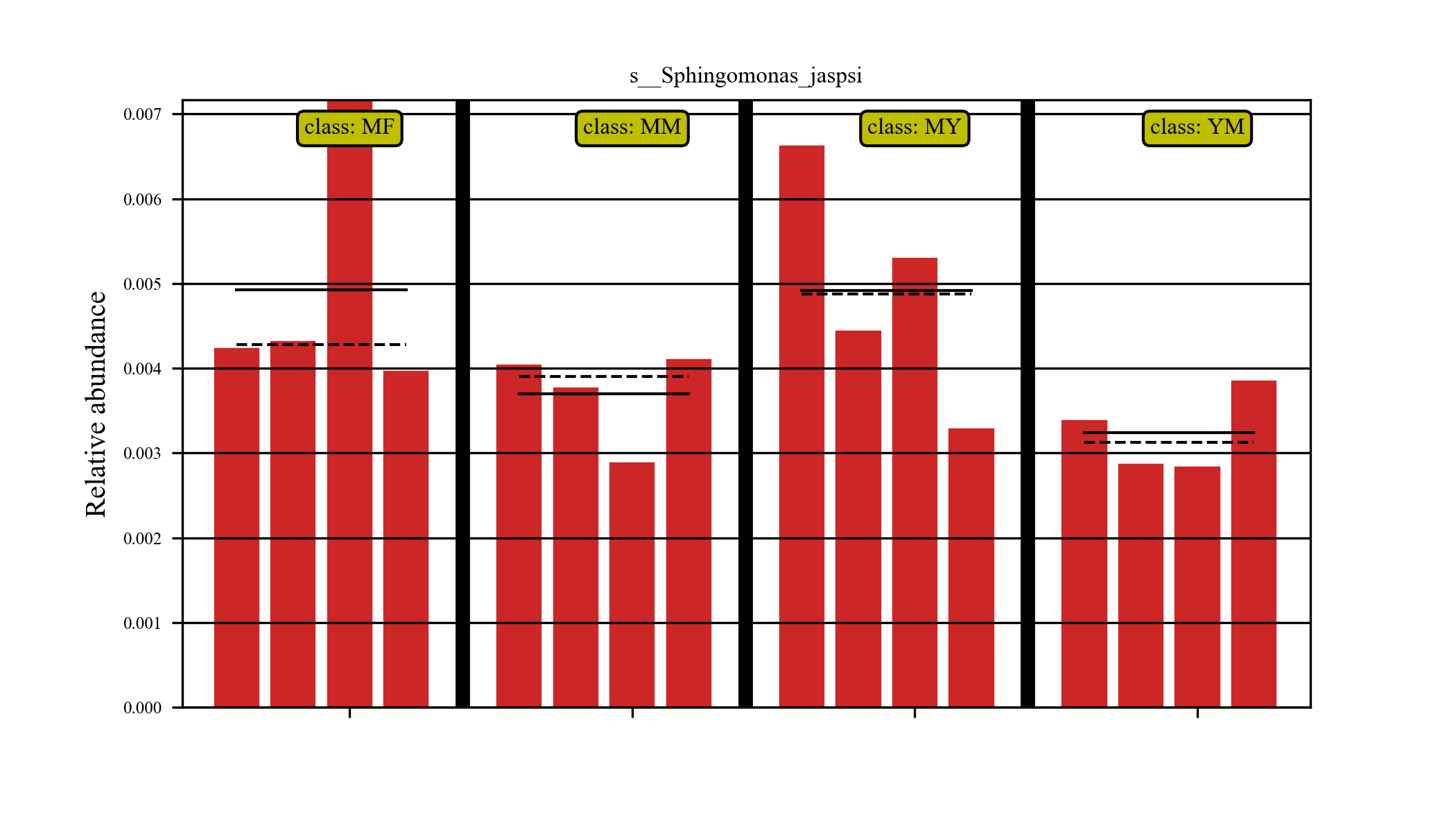

Supplement: Supplementary file 1 [file Data_Sheet_1.ZIP › Supplementary figure 1. bacterial biomarker community/1_s__Sphingomonas_jaspsi.png]

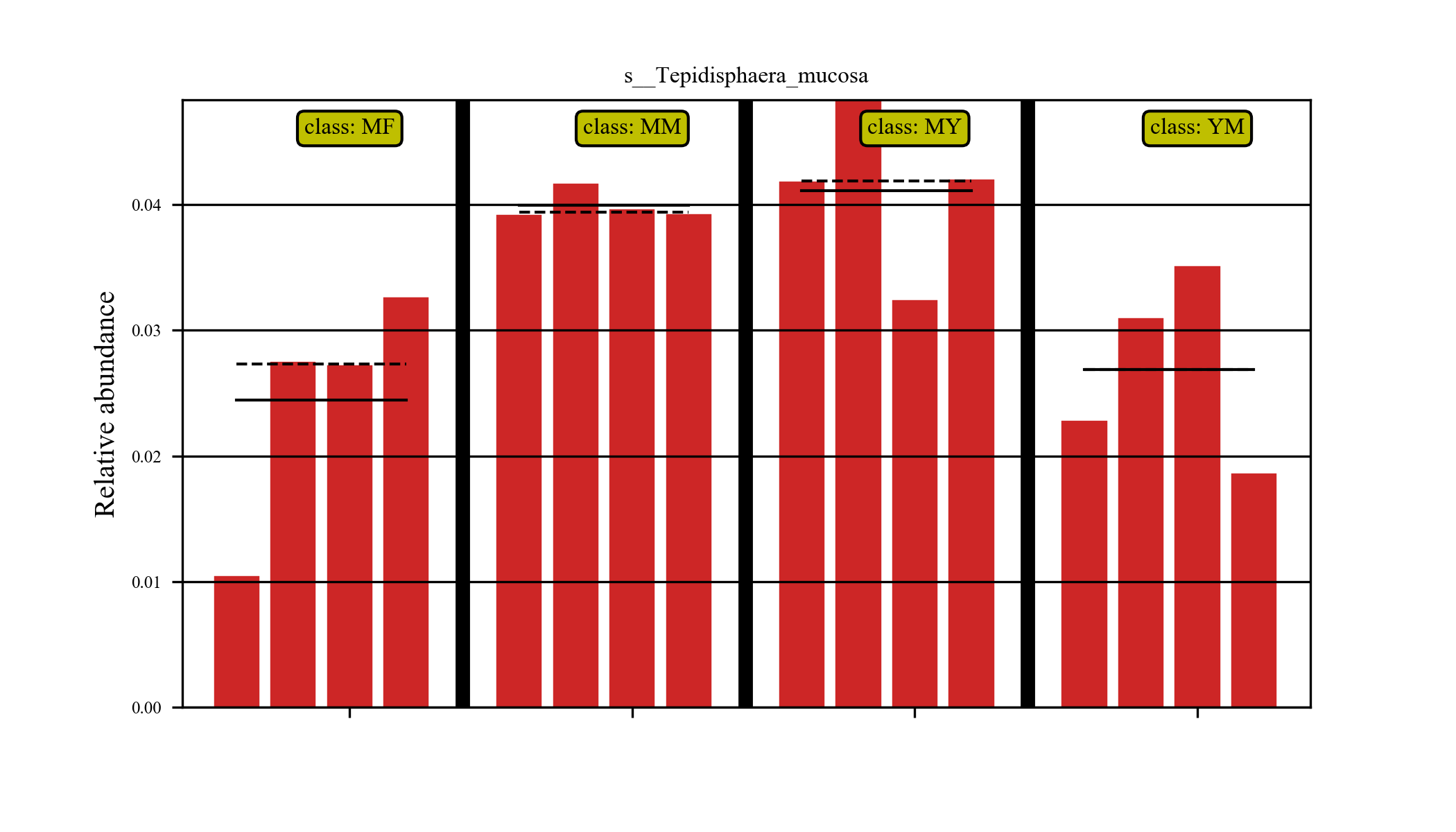

Supplement: Supplementary file 1 [file Data_Sheet_1.ZIP › Supplementary figure 1. bacterial biomarker community/1_s__Tepidisphaera_mucosa.png]

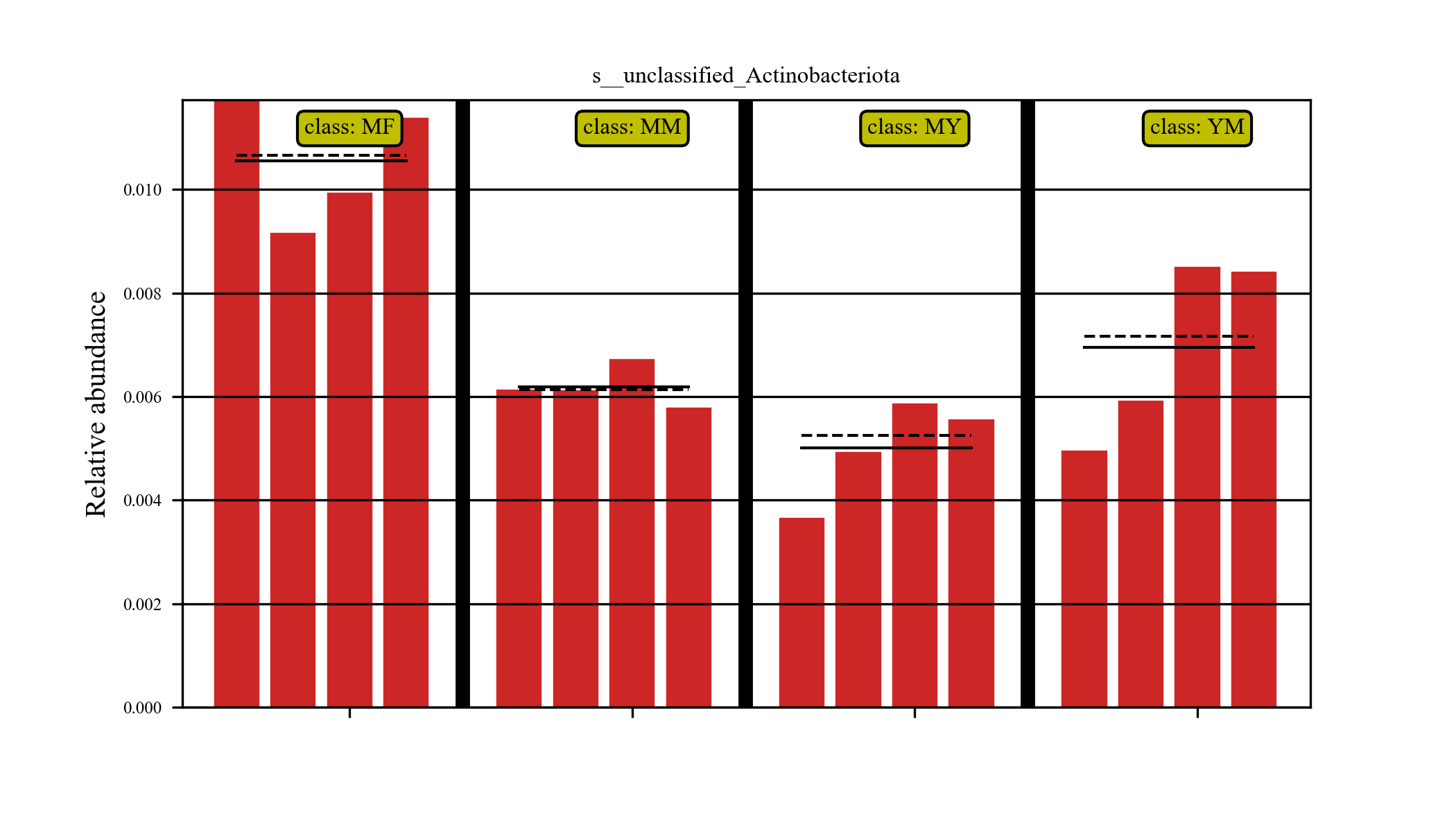

Supplement: Supplementary file 1 [file Data_Sheet_1.ZIP › Supplementary figure 1. bacterial biomarker community/1_s__unclassified_Actinobacteriota.png]

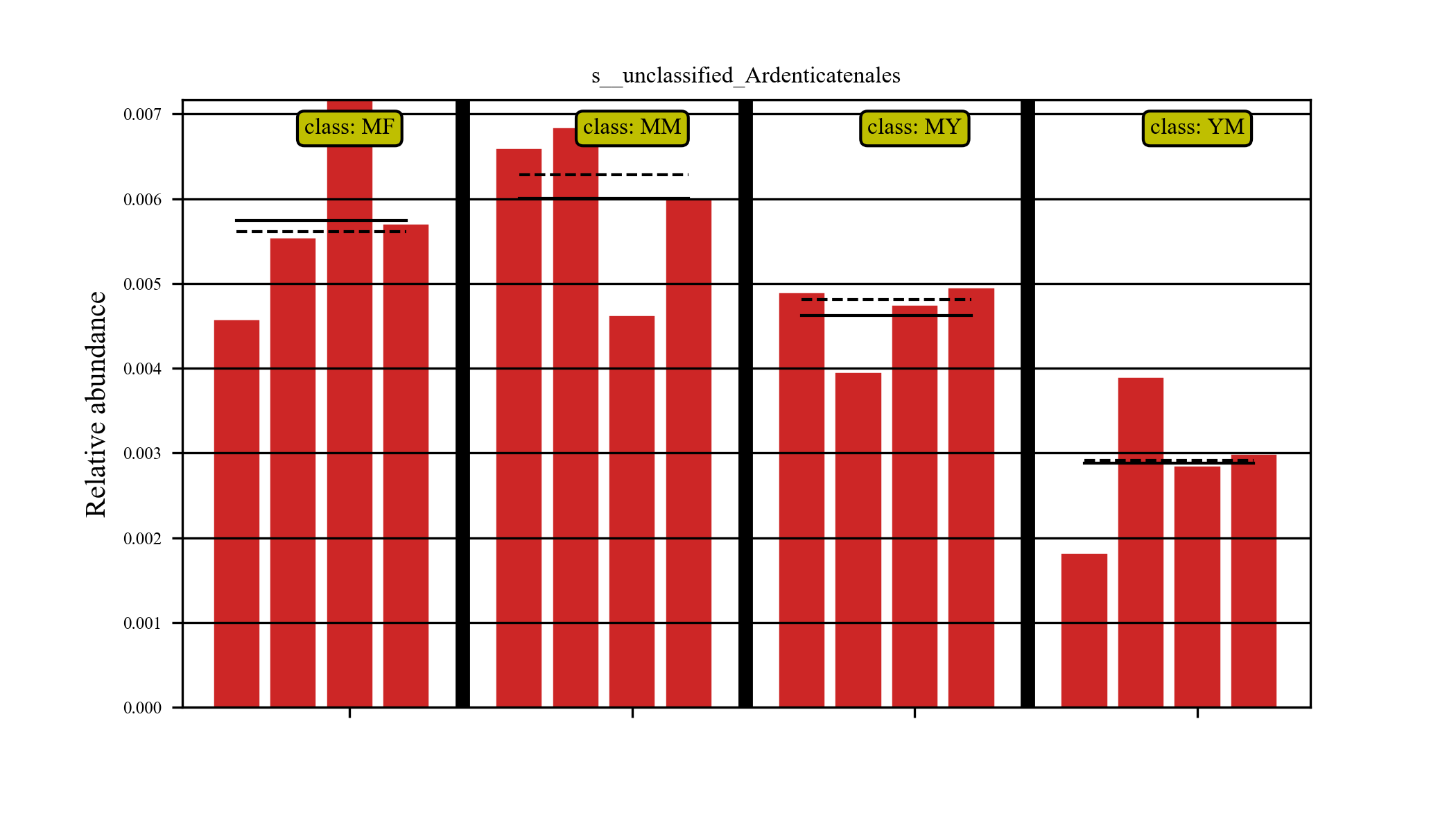

Supplement: Supplementary file 1 [file Data_Sheet_1.ZIP › Supplementary figure 1. bacterial biomarker community/1_s__unclassified_Ardenticatenales.png]

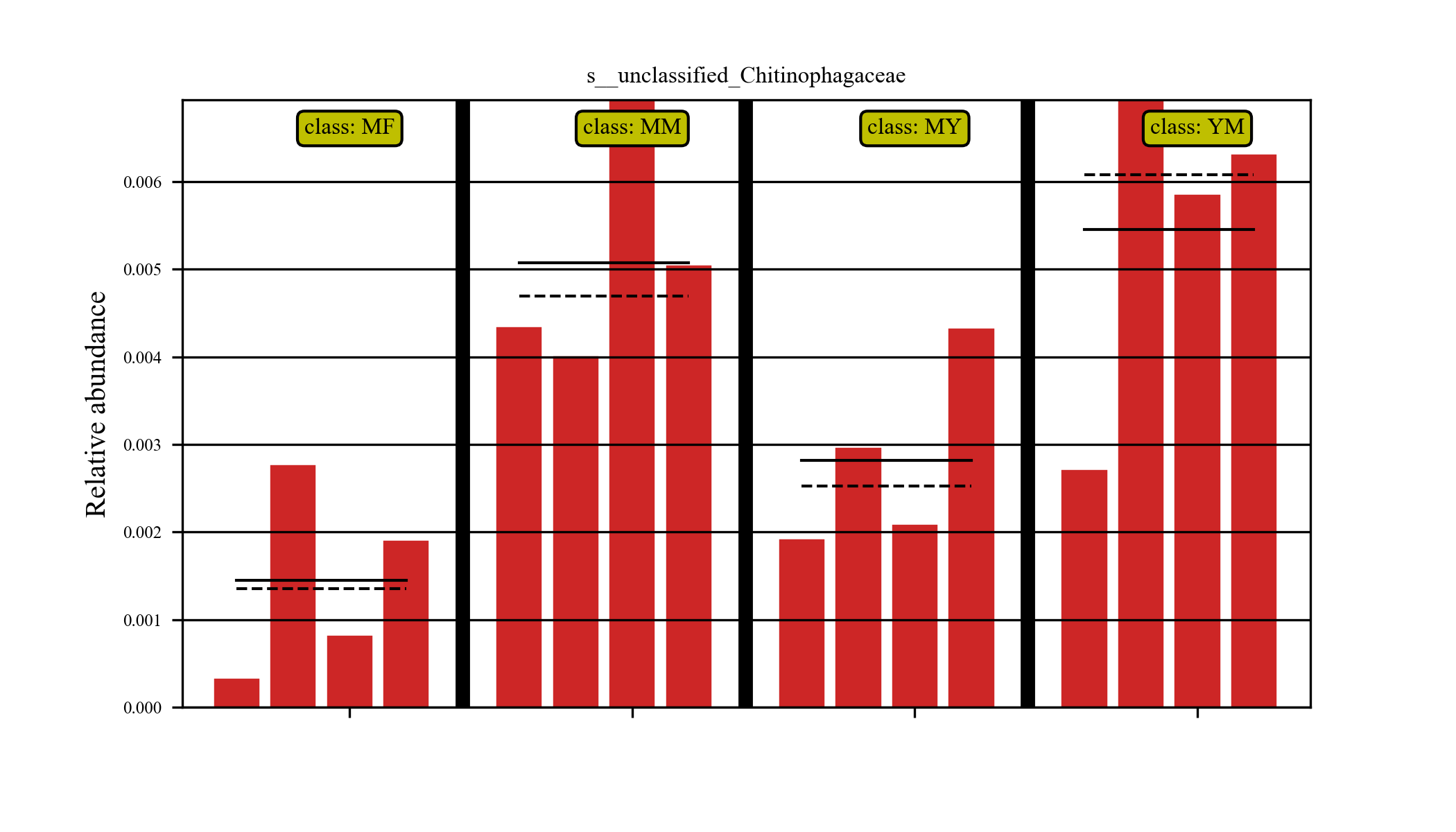

Supplement: Supplementary file 1 [file Data_Sheet_1.ZIP › Supplementary figure 1. bacterial biomarker community/1_s__unclassified_Chitinophagaceae.png]

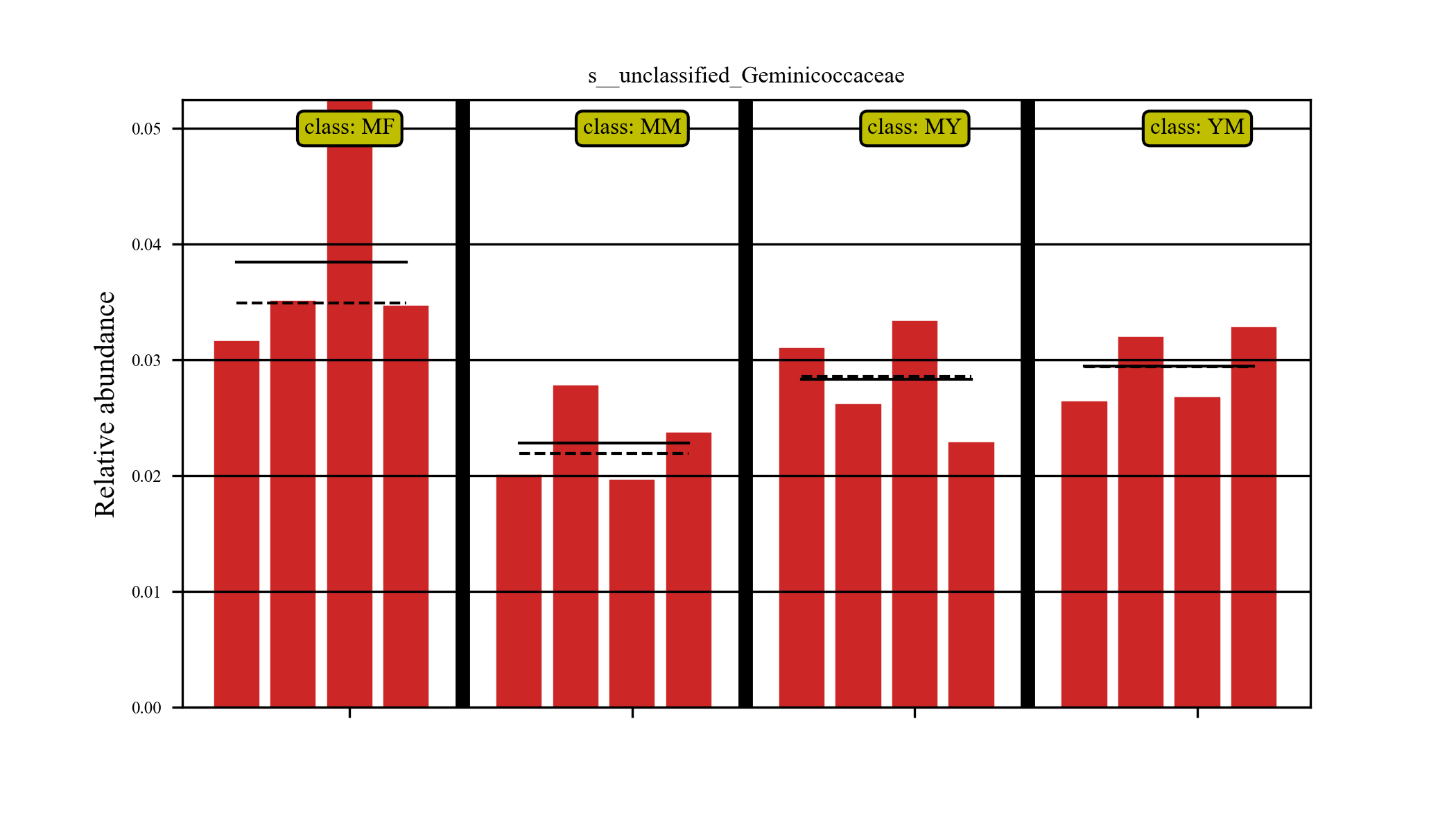

Supplement: Supplementary file 1 [file Data_Sheet_1.ZIP › Supplementary figure 1. bacterial biomarker community/1_s__unclassified_Geminicoccaceae.png]

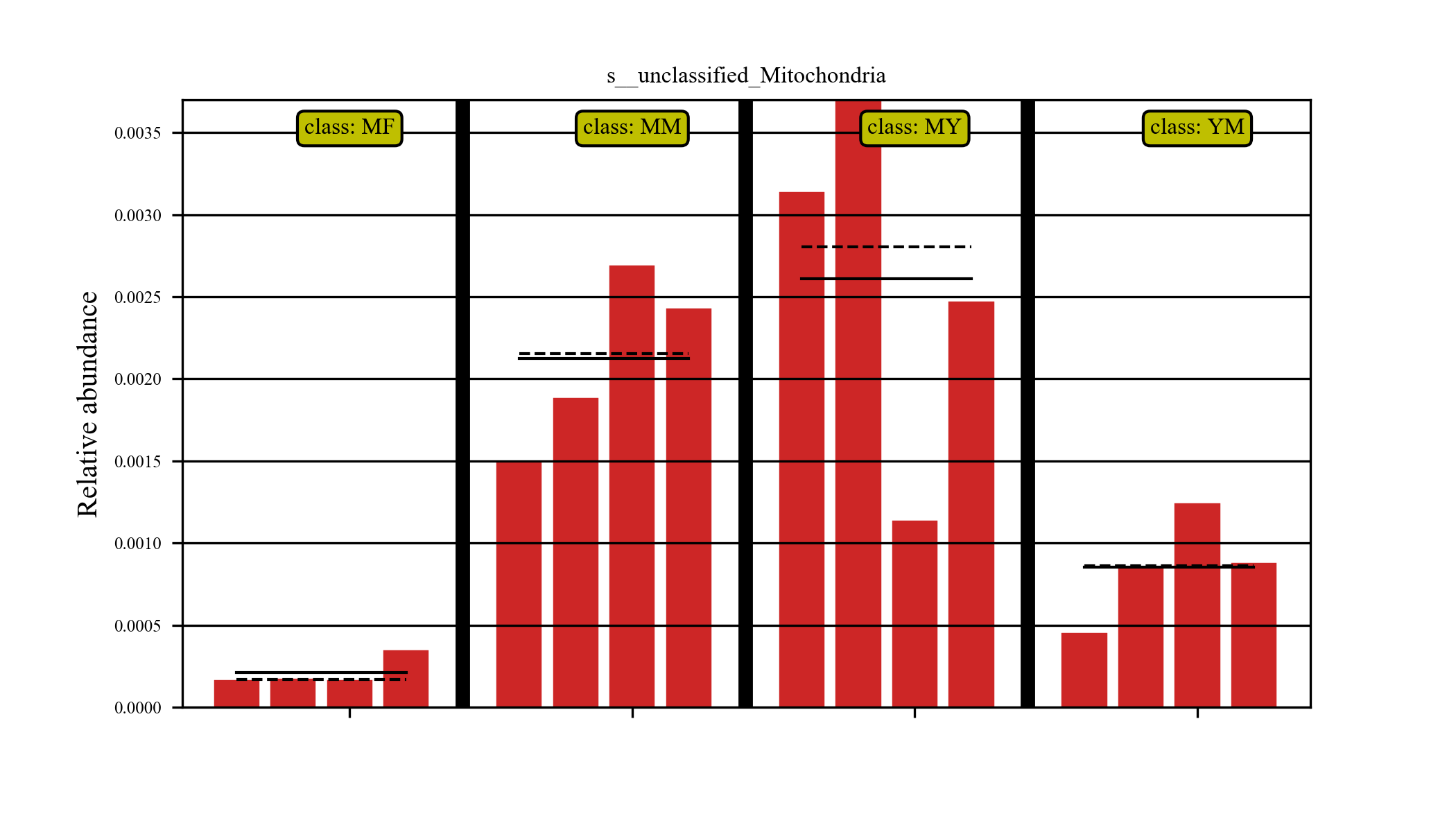

Supplement: Supplementary file 1 [file Data_Sheet_1.ZIP › Supplementary figure 1. bacterial biomarker community/1_s__unclassified_Mitochondria.png]

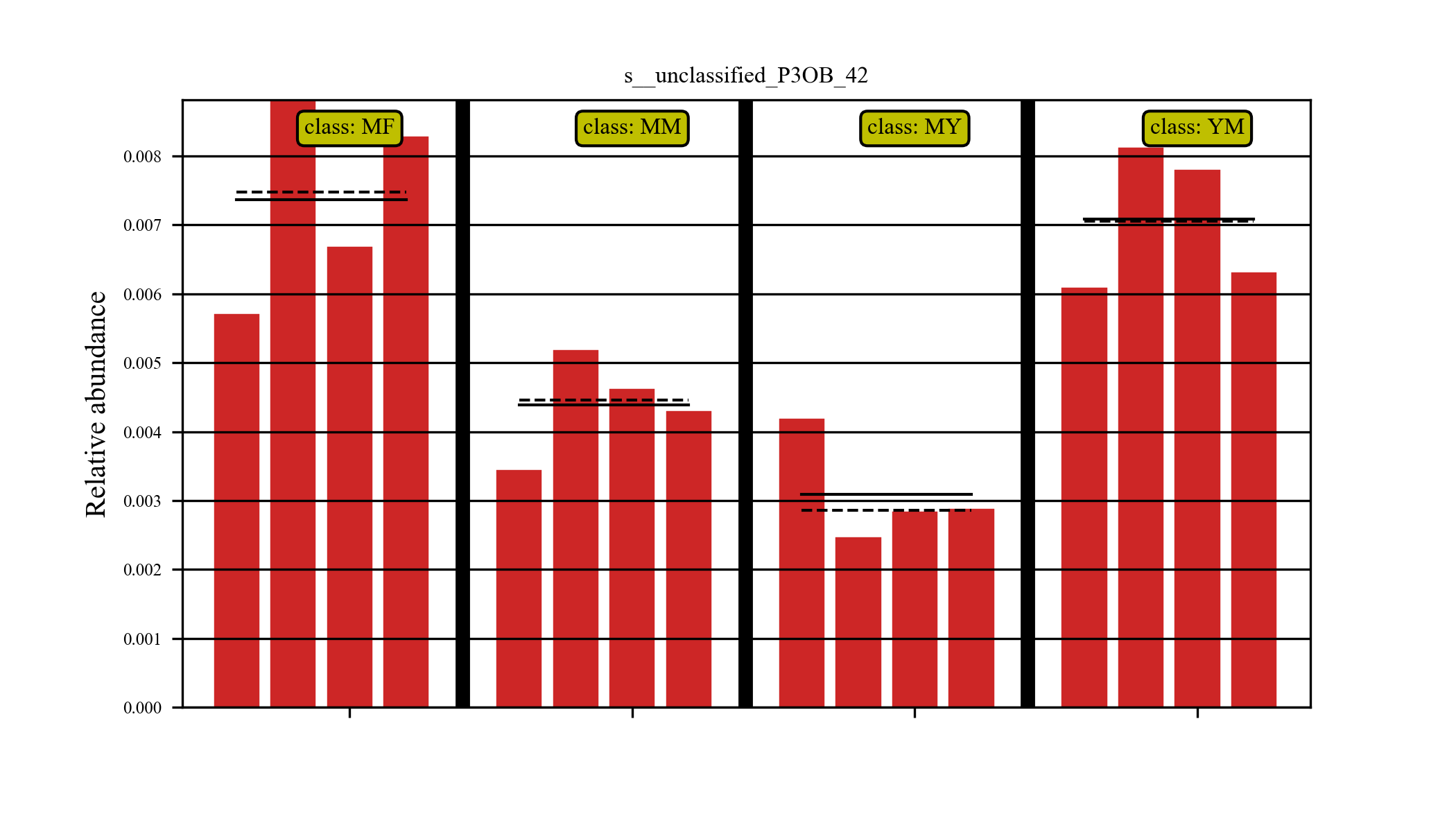

Supplement: Supplementary file 1 [file Data_Sheet_1.ZIP › Supplementary figure 1. bacterial biomarker community/1_s__unclassified_P3OB_42.png]

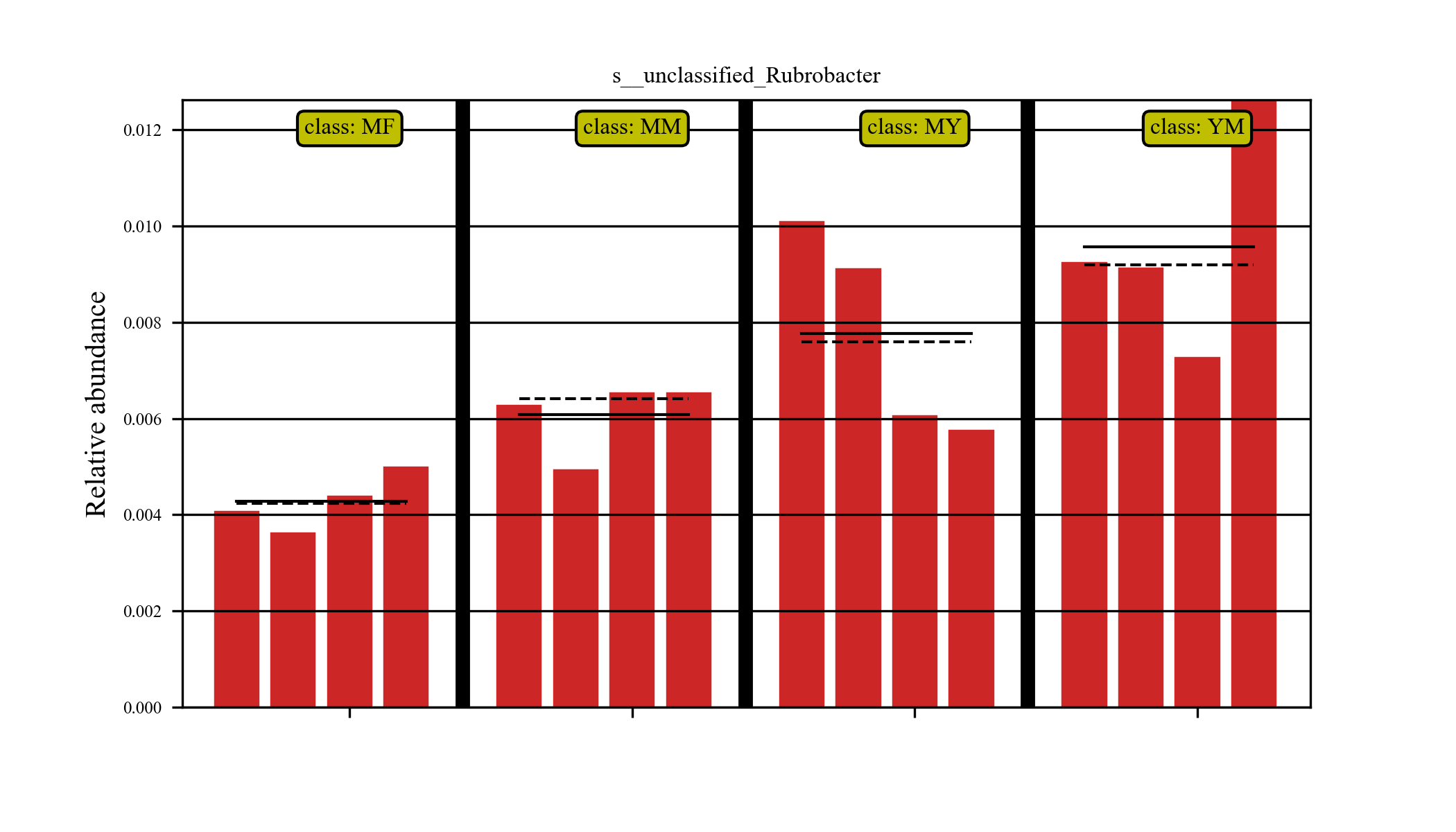

Supplement: Supplementary file 1 [file Data_Sheet_1.ZIP › Supplementary figure 1. bacterial biomarker community/1_s__unclassified_Rubrobacter.png]

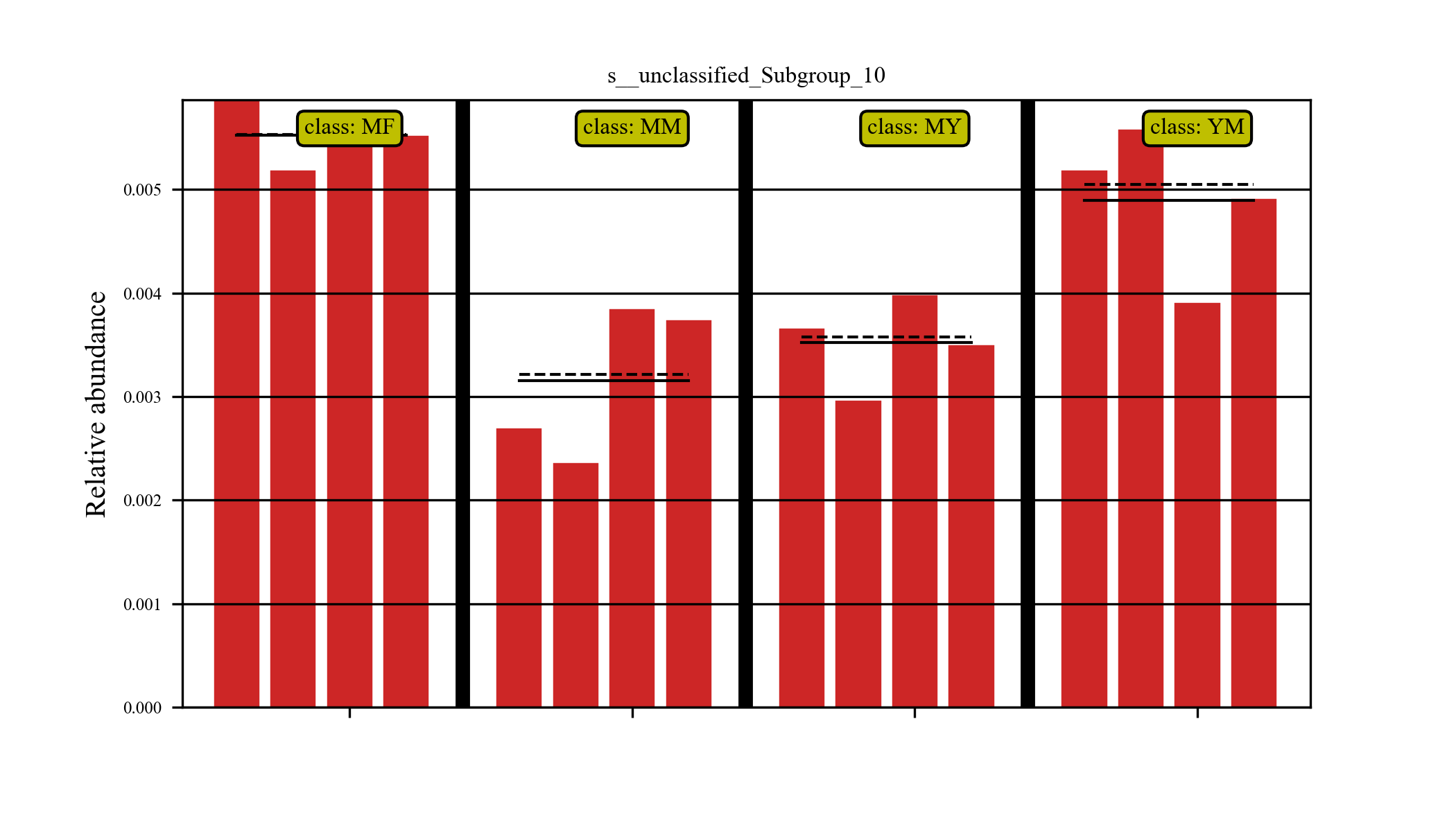

Supplement: Supplementary file 1 [file Data_Sheet_1.ZIP › Supplementary figure 1. bacterial biomarker community/1_s__unclassified_Subgroup_10.png]

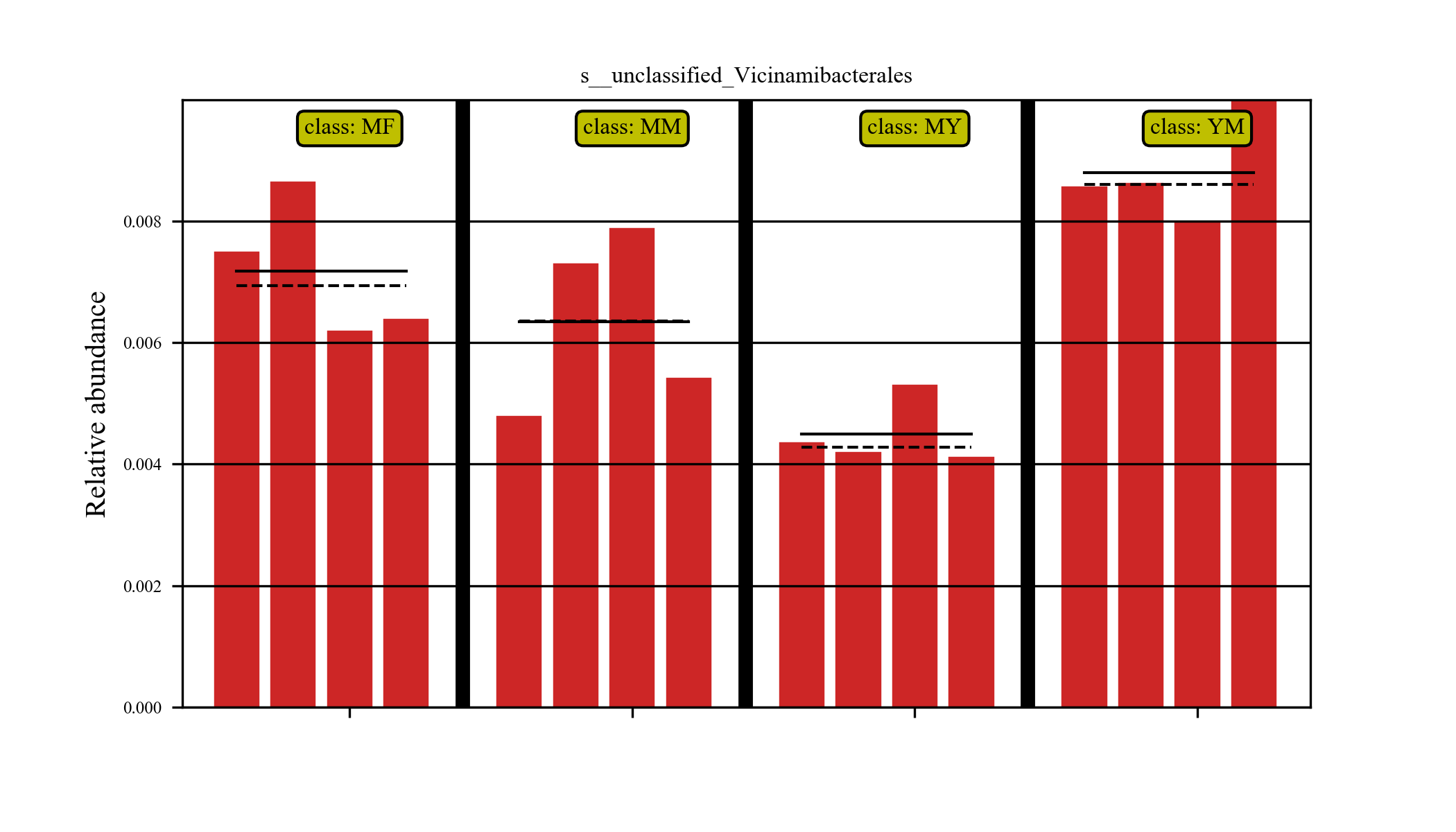

Supplement: Supplementary file 1 [file Data_Sheet_1.ZIP › Supplementary figure 1. bacterial biomarker community/1_s__unclassified_Vicinamibacterales.png]

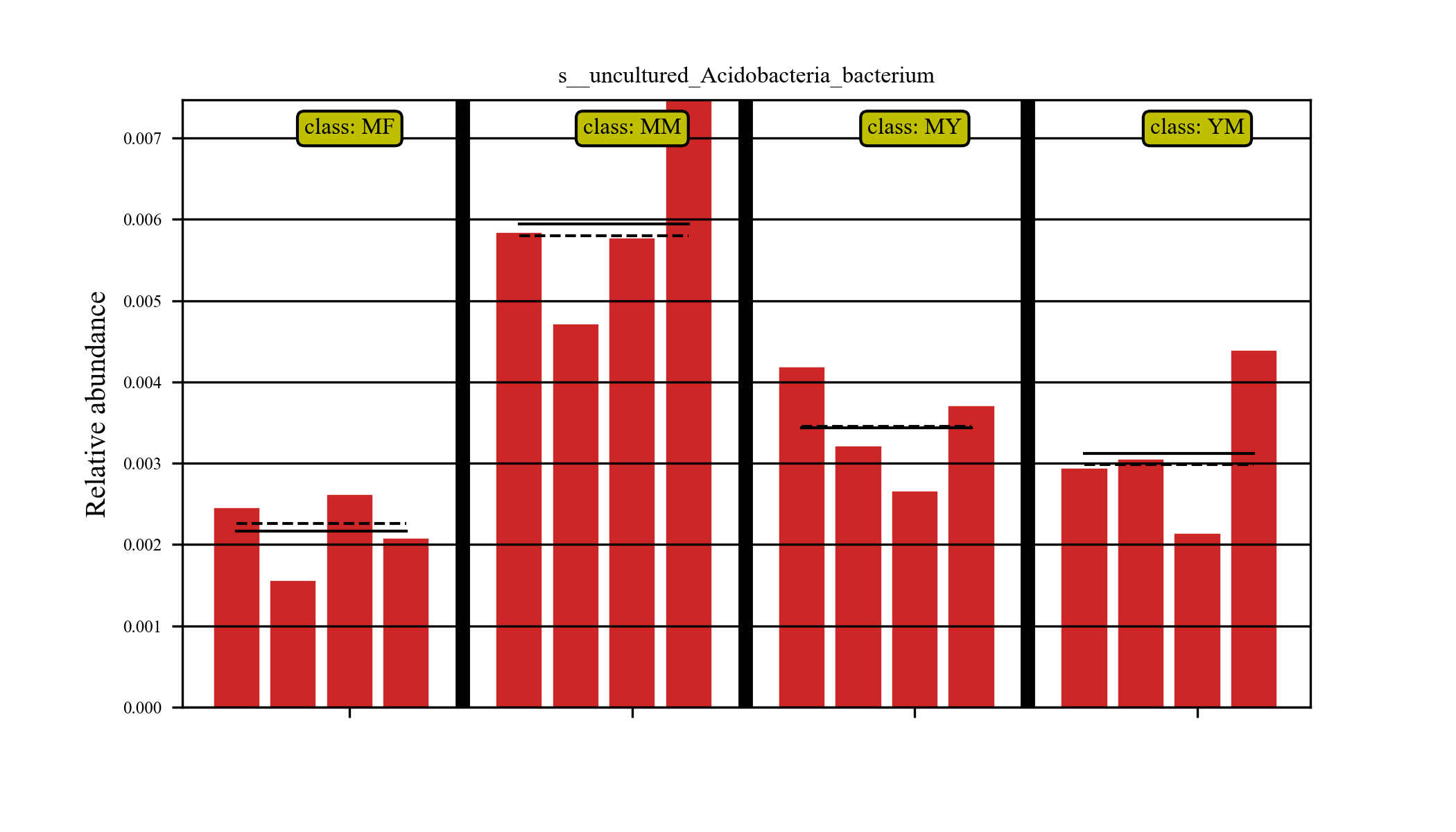

Supplement: Supplementary file 1 [file Data_Sheet_1.ZIP › Supplementary figure 1. bacterial biomarker community/1_s__uncultured_Acidobacteria_bacterium.png]

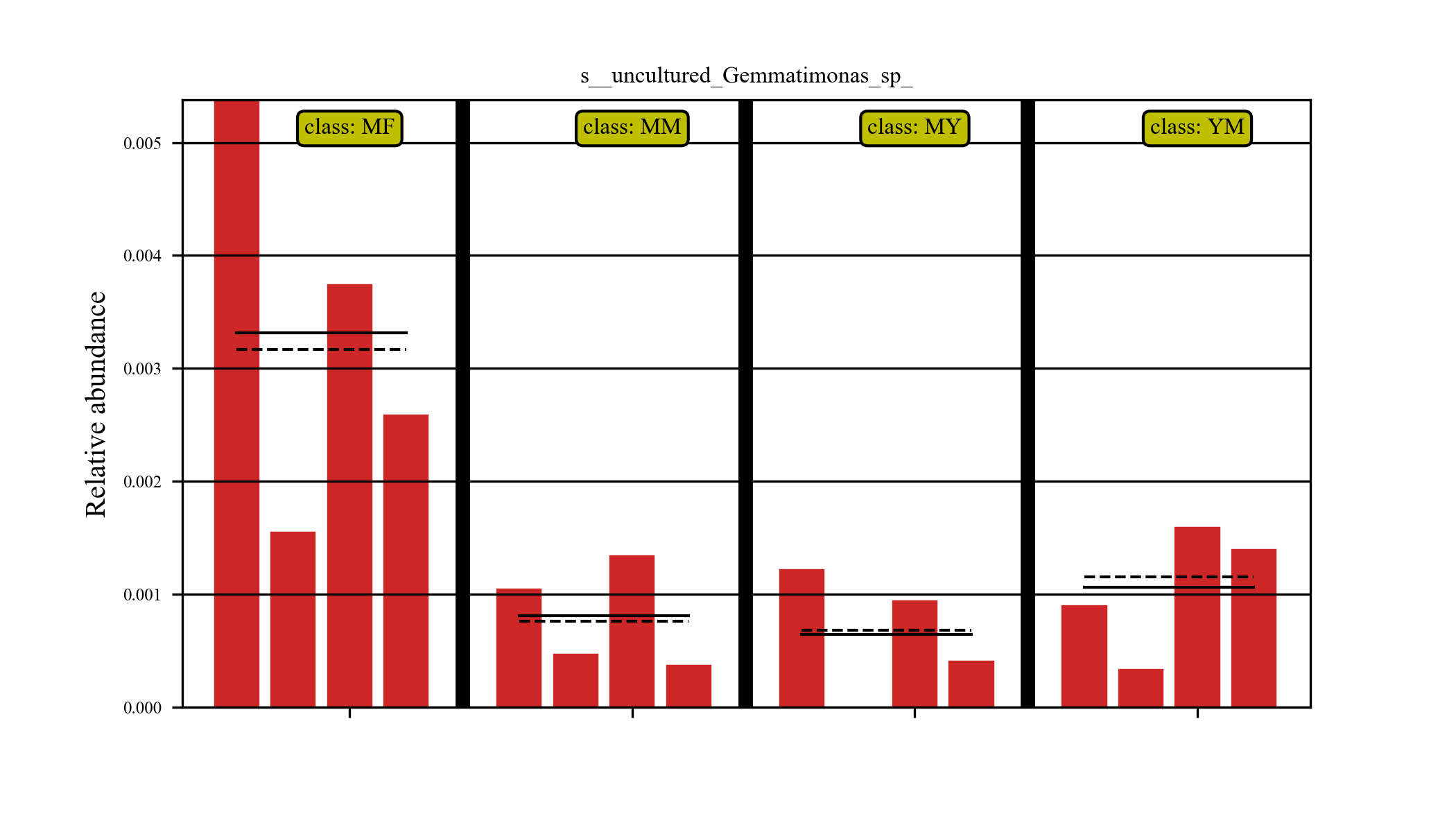

Supplement: Supplementary file 1 [file Data_Sheet_1.ZIP › Supplementary figure 1. bacterial biomarker community/1_s__uncultured_Gemmatimonas_sp_.png]

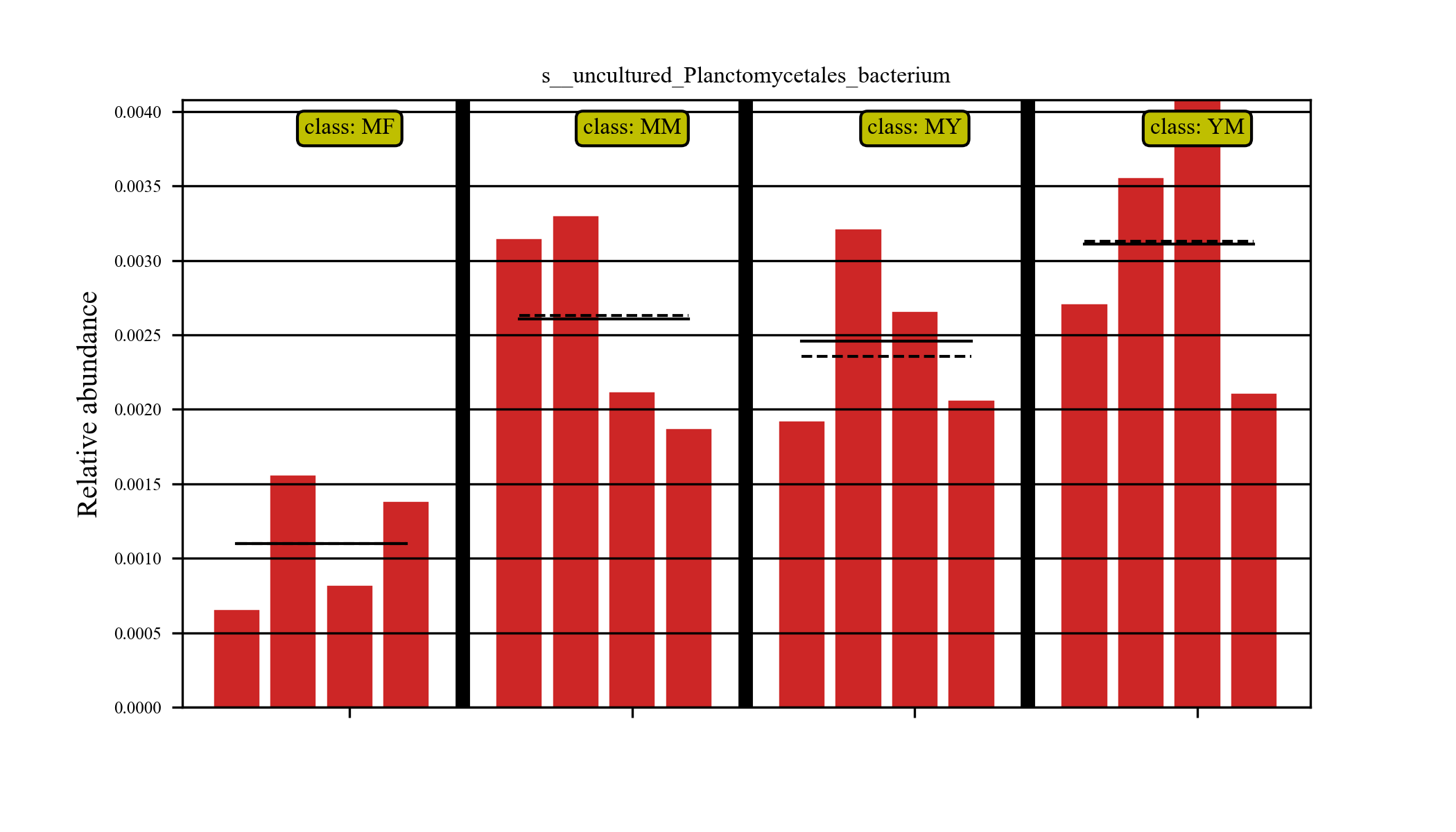

Supplement: Supplementary file 1 [file Data_Sheet_1.ZIP › Supplementary figure 1. bacterial biomarker community/1_s__uncultured_Planctomycetales_bacterium.png]

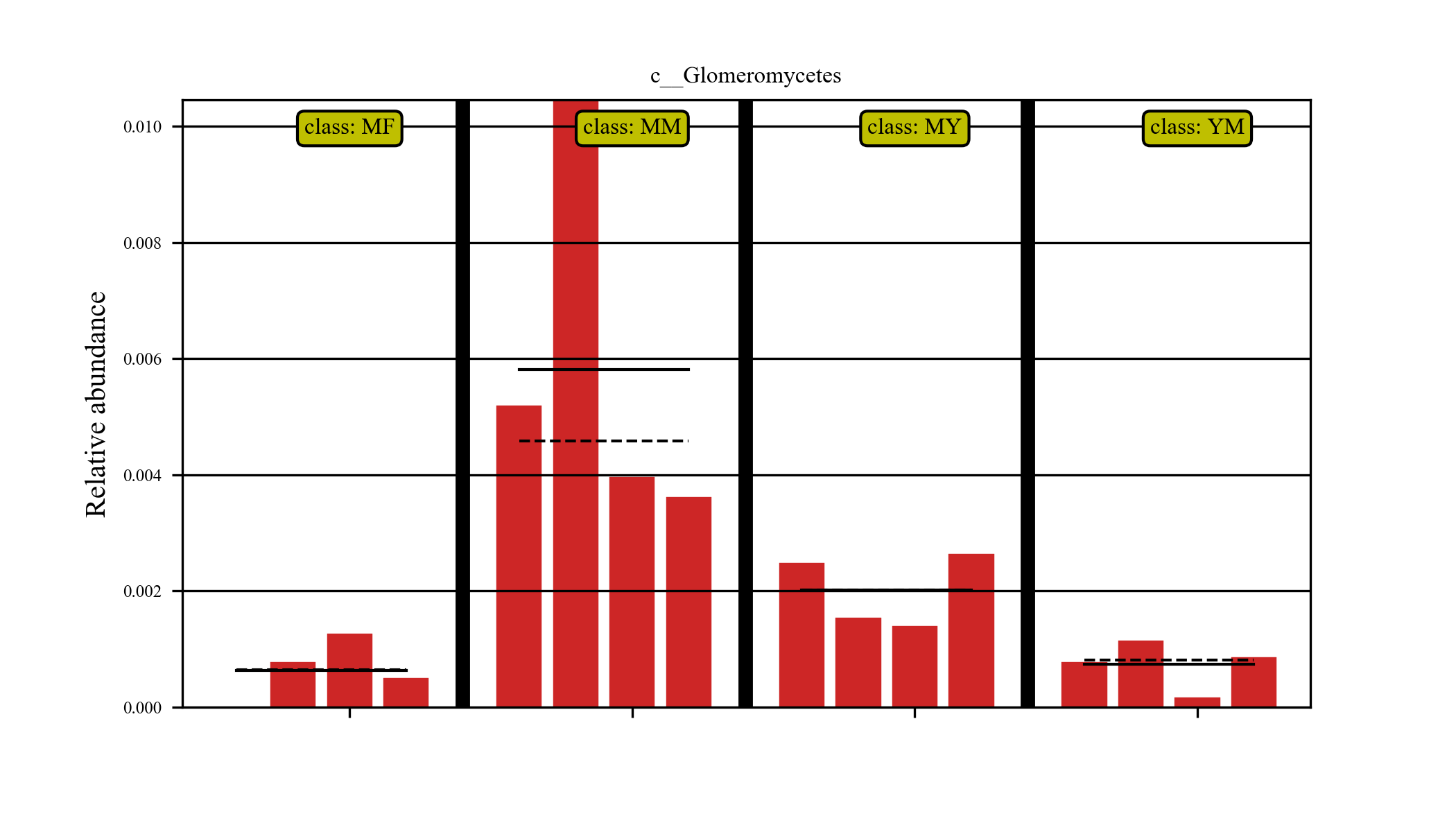

Supplement: Supplementary file 2 [file Data_Sheet_2.ZIP › Supplementary figure 2. fungal biomarker community/1_c__Glomeromycetes.png]

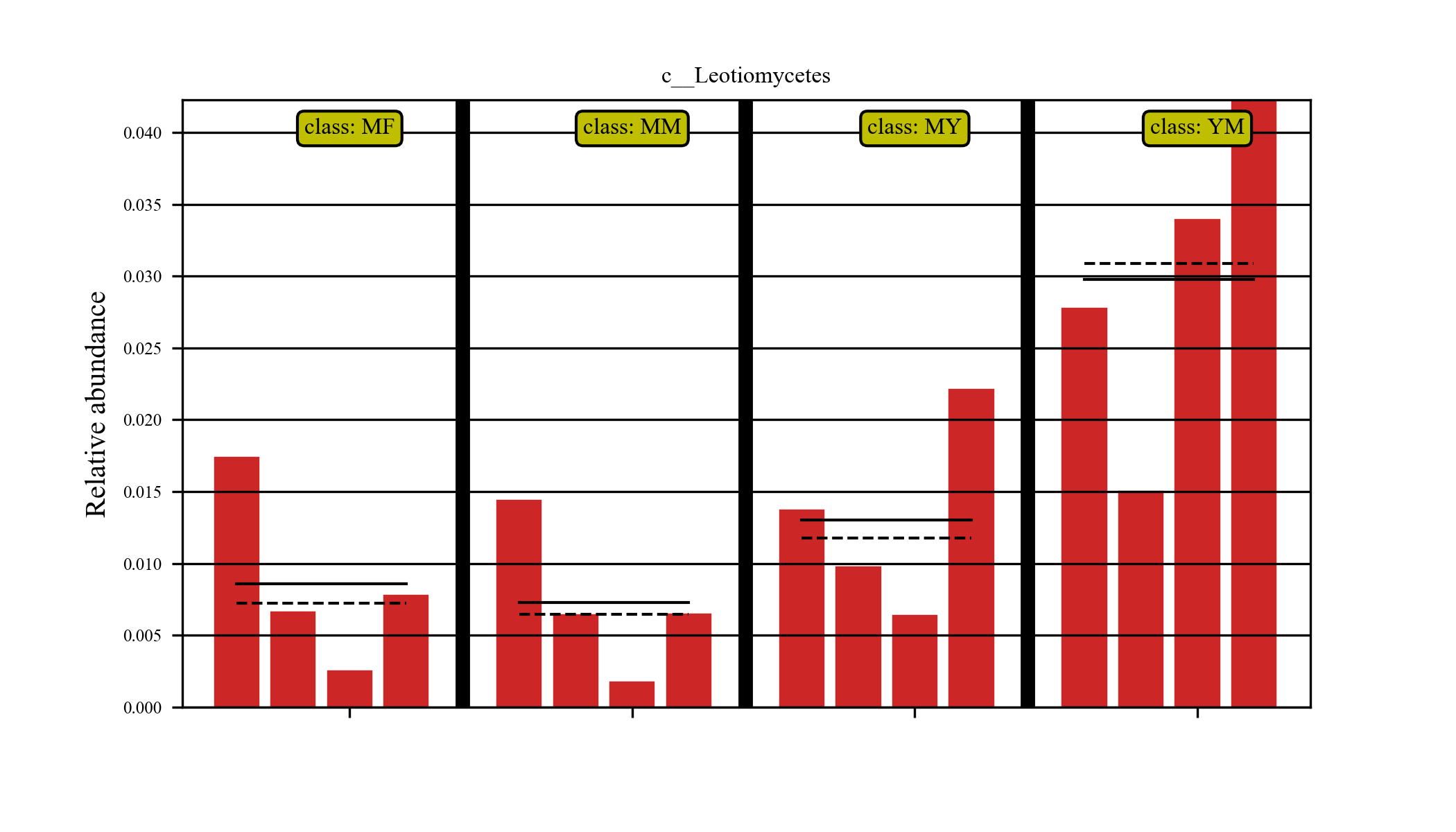

Supplement: Supplementary file 2 [file Data_Sheet_2.ZIP › Supplementary figure 2. fungal biomarker community/1_c__Leotiomycetes.png]

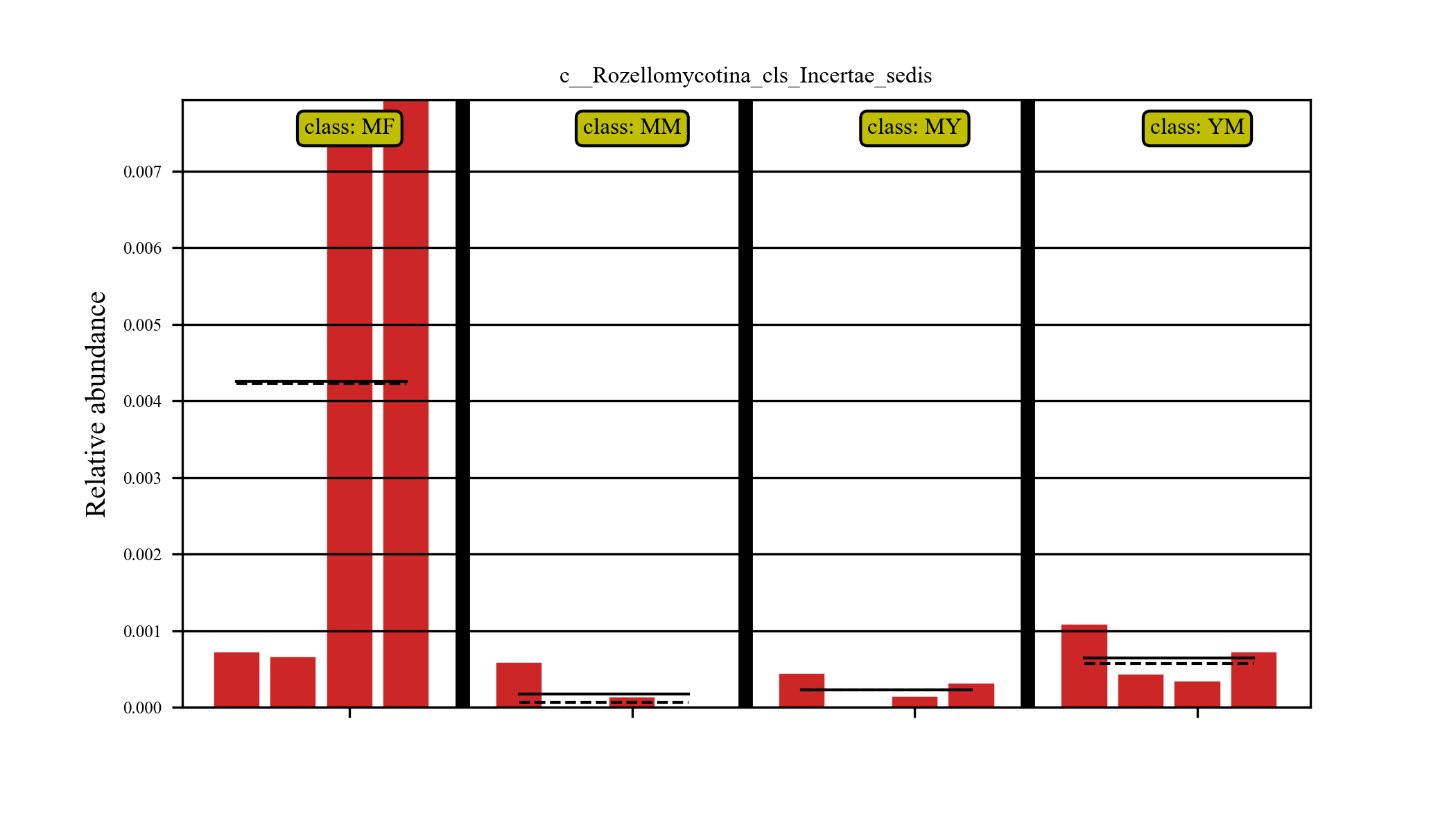

Supplement: Supplementary file 2 [file Data_Sheet_2.ZIP › Supplementary figure 2. fungal biomarker community/1_c__Rozellomycotina_cls_Incertae_sedis.png]

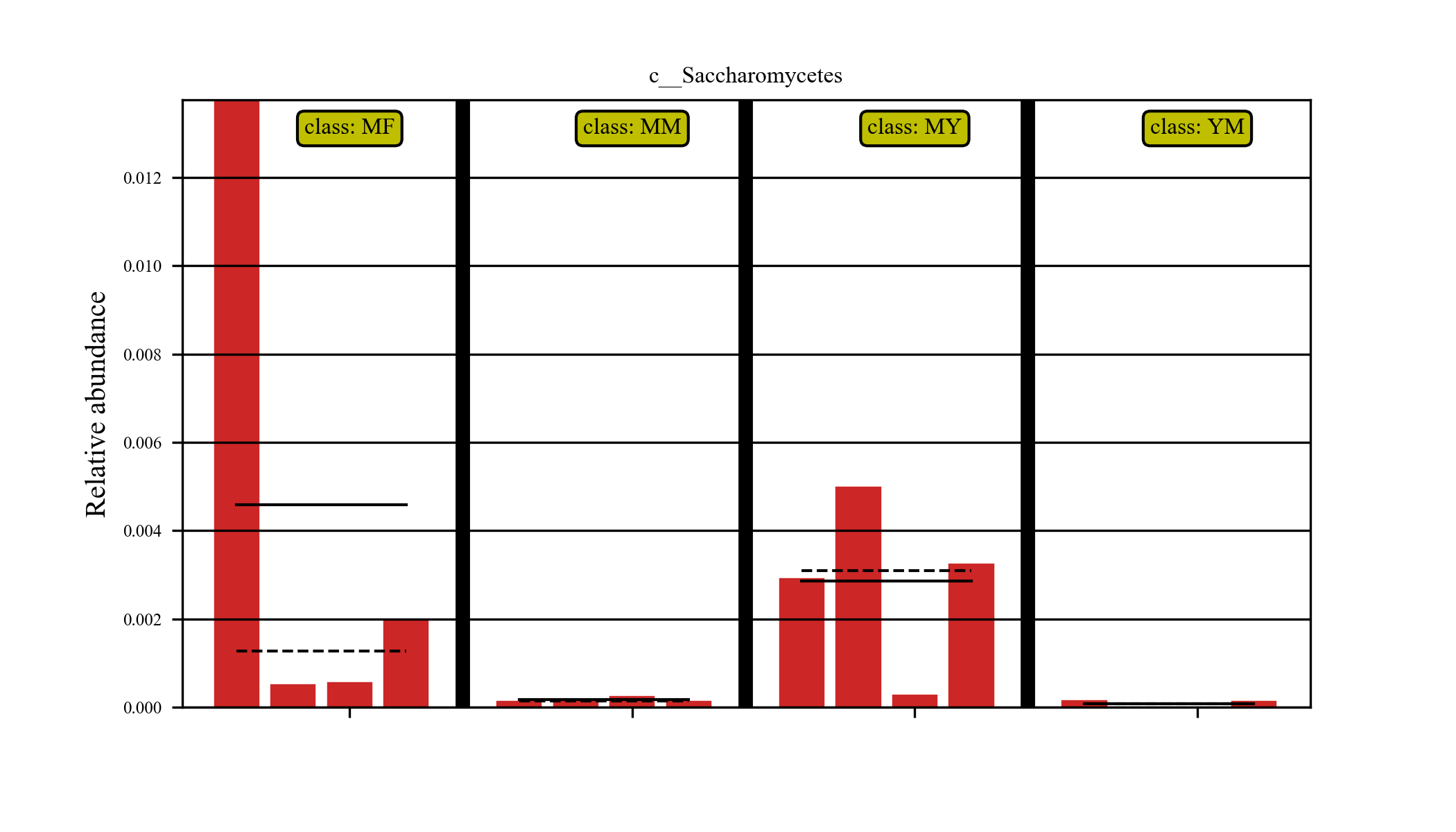

Supplement: Supplementary file 2 [file Data_Sheet_2.ZIP › Supplementary figure 2. fungal biomarker community/1_c__Saccharomycetes.png]

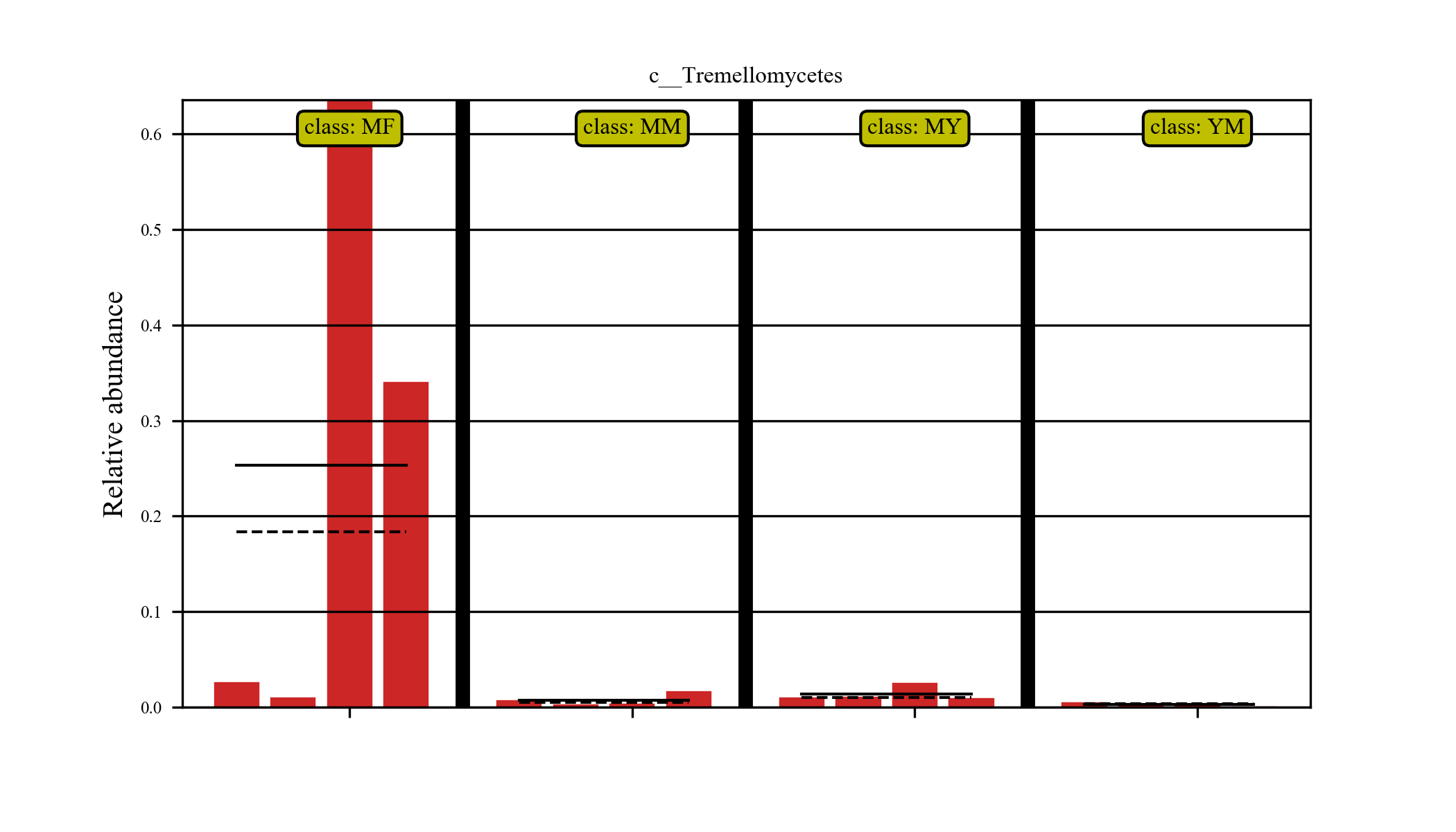

Supplement: Supplementary file 2 [file Data_Sheet_2.ZIP › Supplementary figure 2. fungal biomarker community/1_c__Tremellomycetes.png]

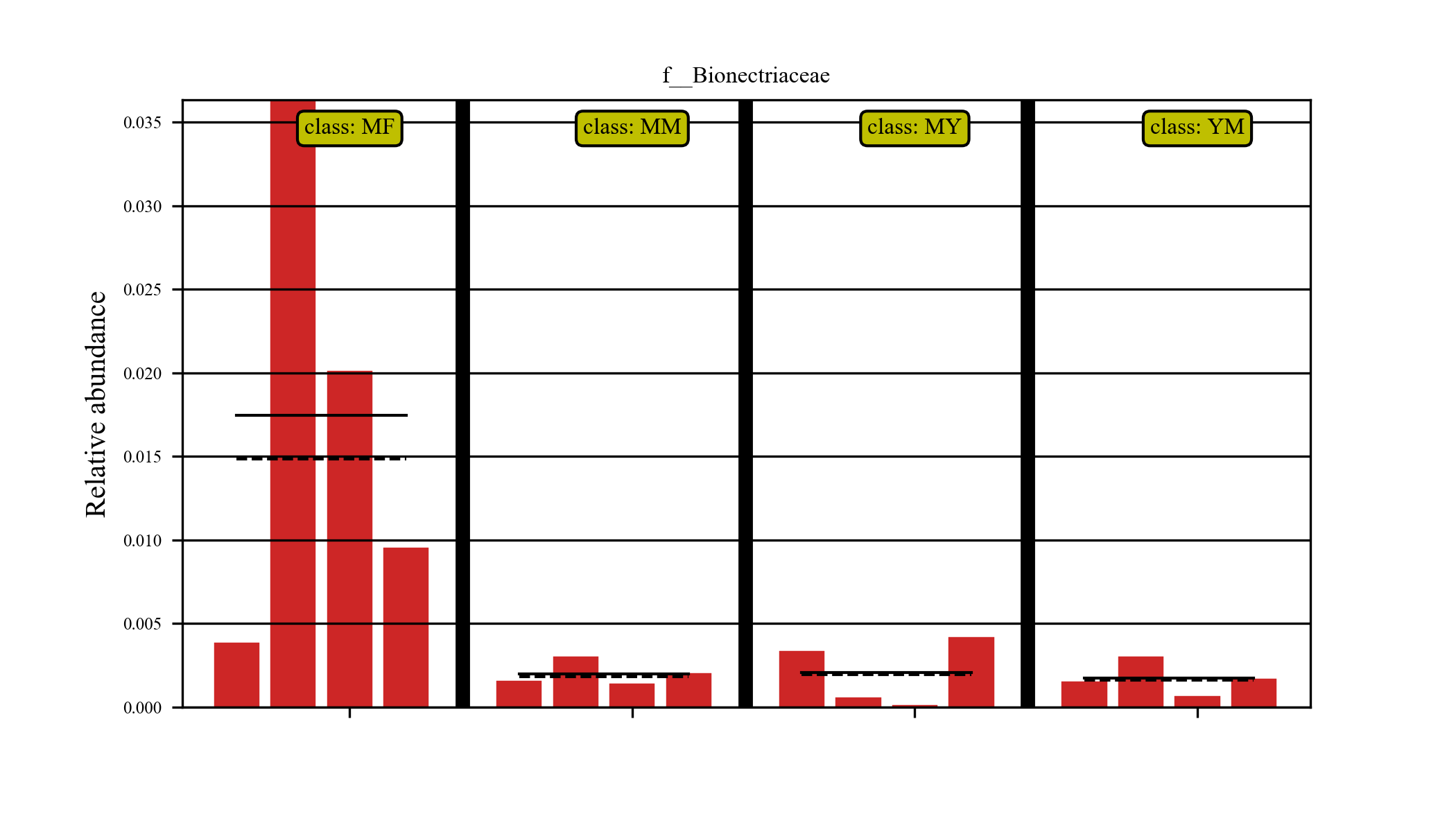

Supplement: Supplementary file 2 [file Data_Sheet_2.ZIP › Supplementary figure 2. fungal biomarker community/1_f__Bionectriaceae.png]

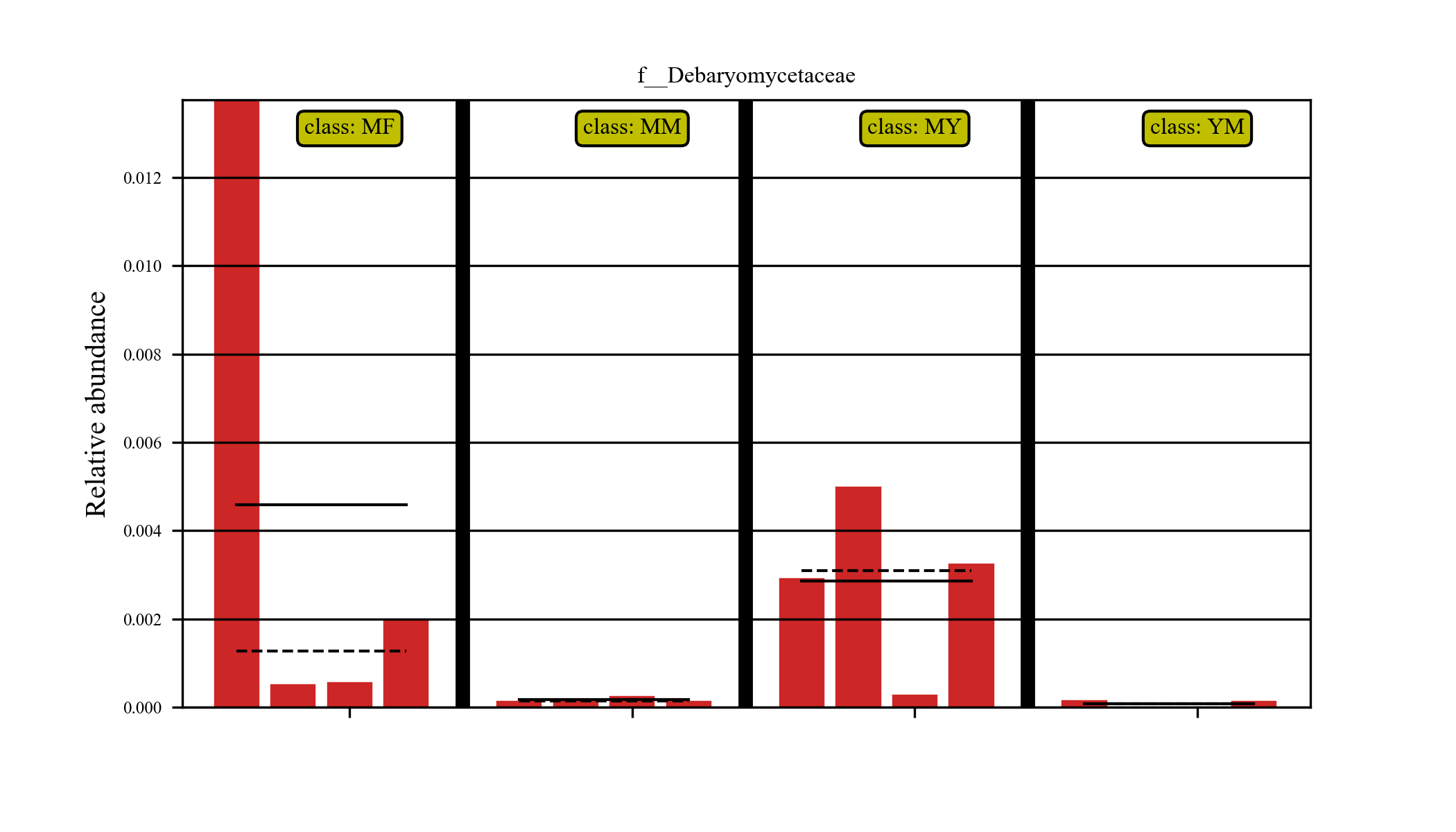

Supplement: Supplementary file 2 [file Data_Sheet_2.ZIP › Supplementary figure 2. fungal biomarker community/1_f__Debaryomycetaceae.png]

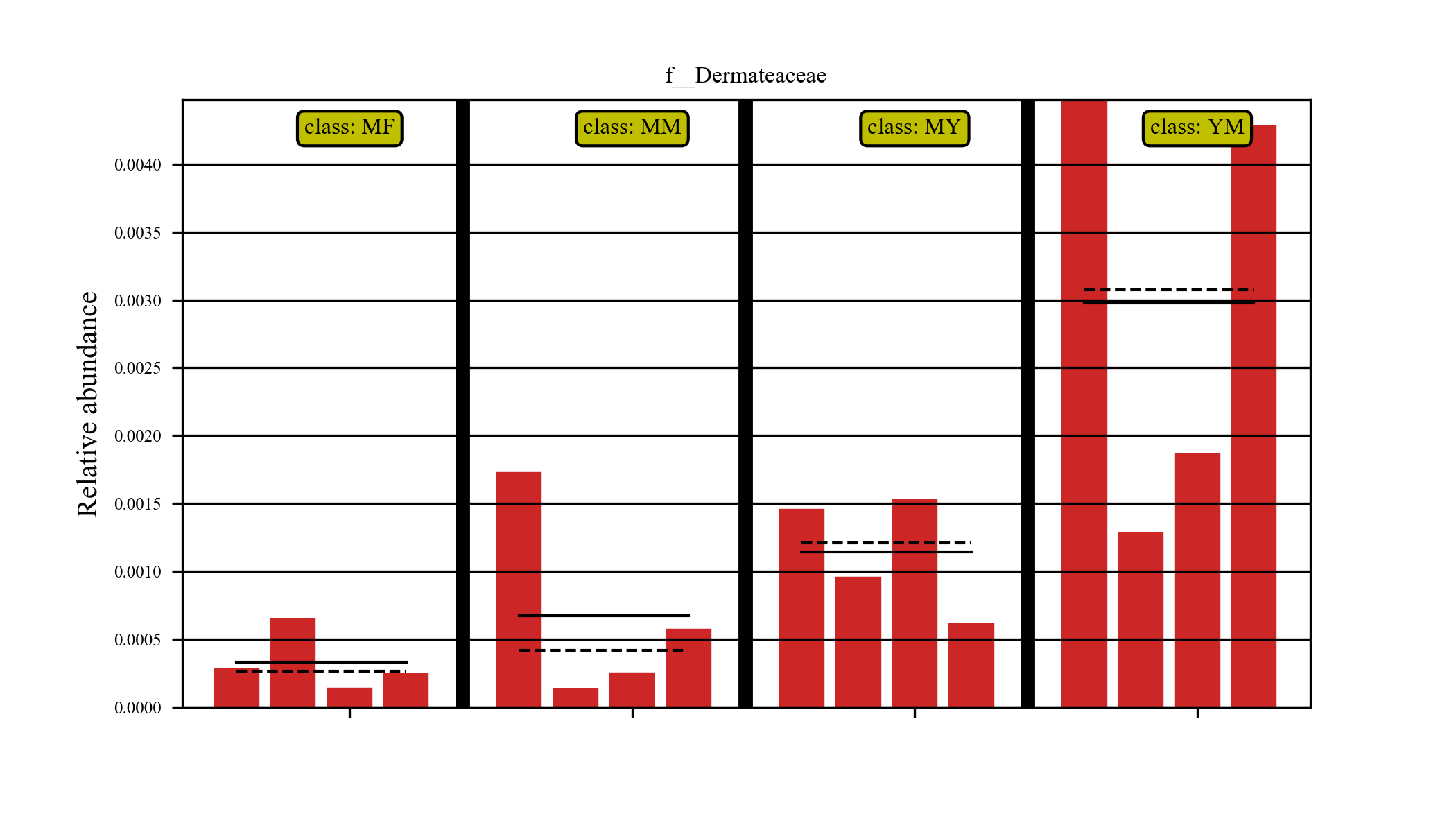

Supplement: Supplementary file 2 [file Data_Sheet_2.ZIP › Supplementary figure 2. fungal biomarker community/1_f__Dermateaceae.png]

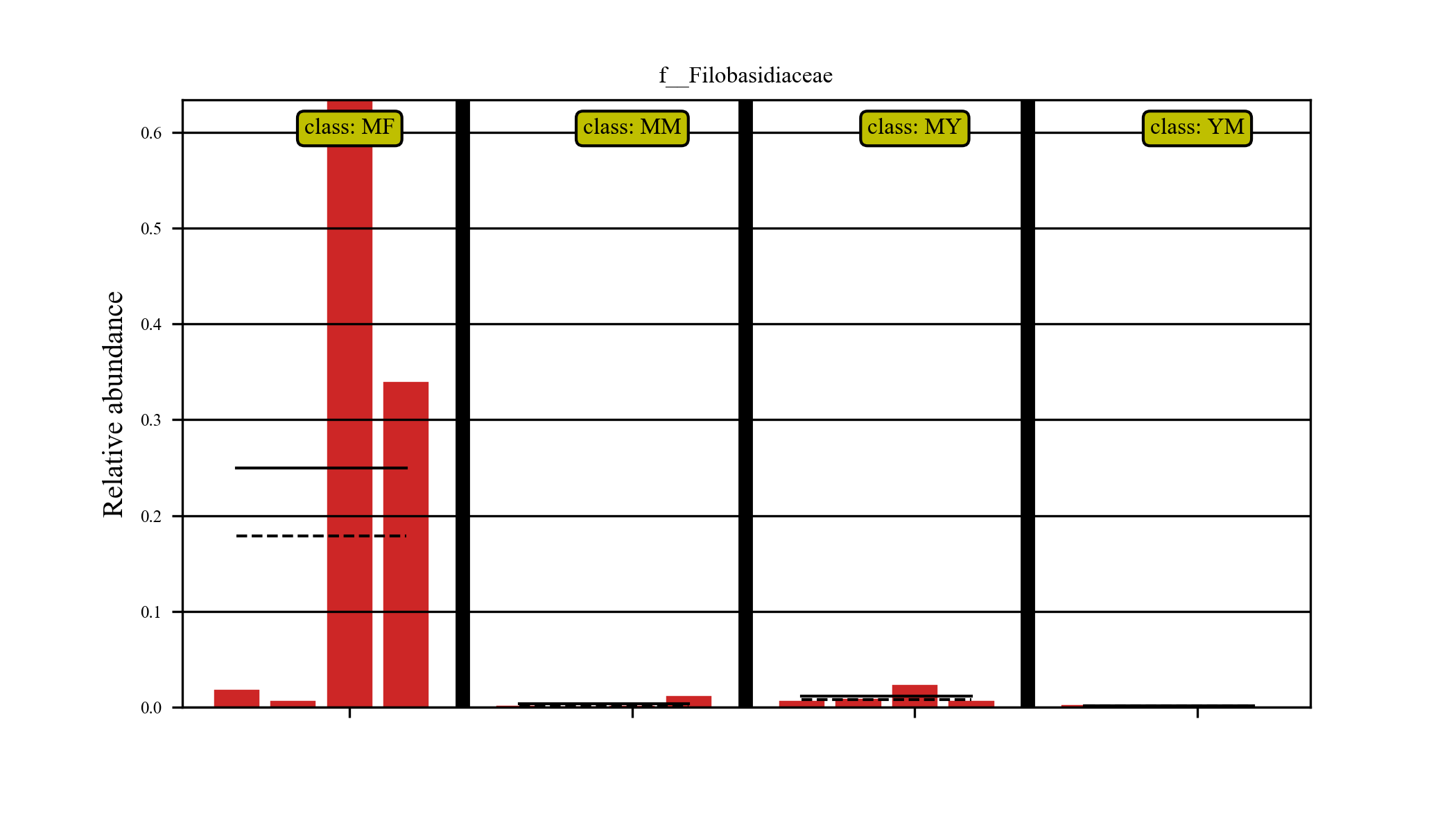

Supplement: Supplementary file 2 [file Data_Sheet_2.ZIP › Supplementary figure 2. fungal biomarker community/1_f__Filobasidiaceae.png]

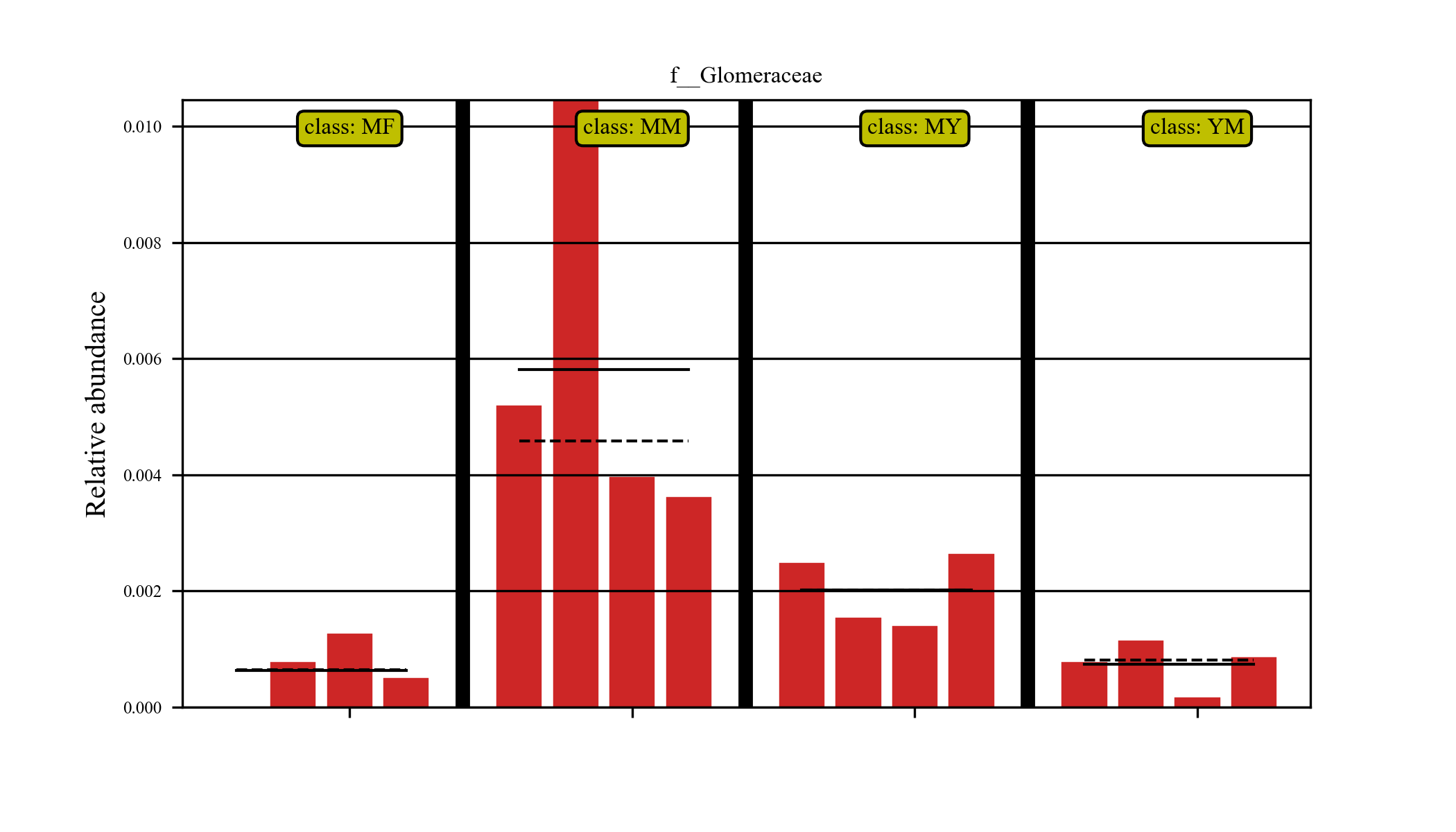

Supplement: Supplementary file 2 [file Data_Sheet_2.ZIP › Supplementary figure 2. fungal biomarker community/1_f__Glomeraceae.png]

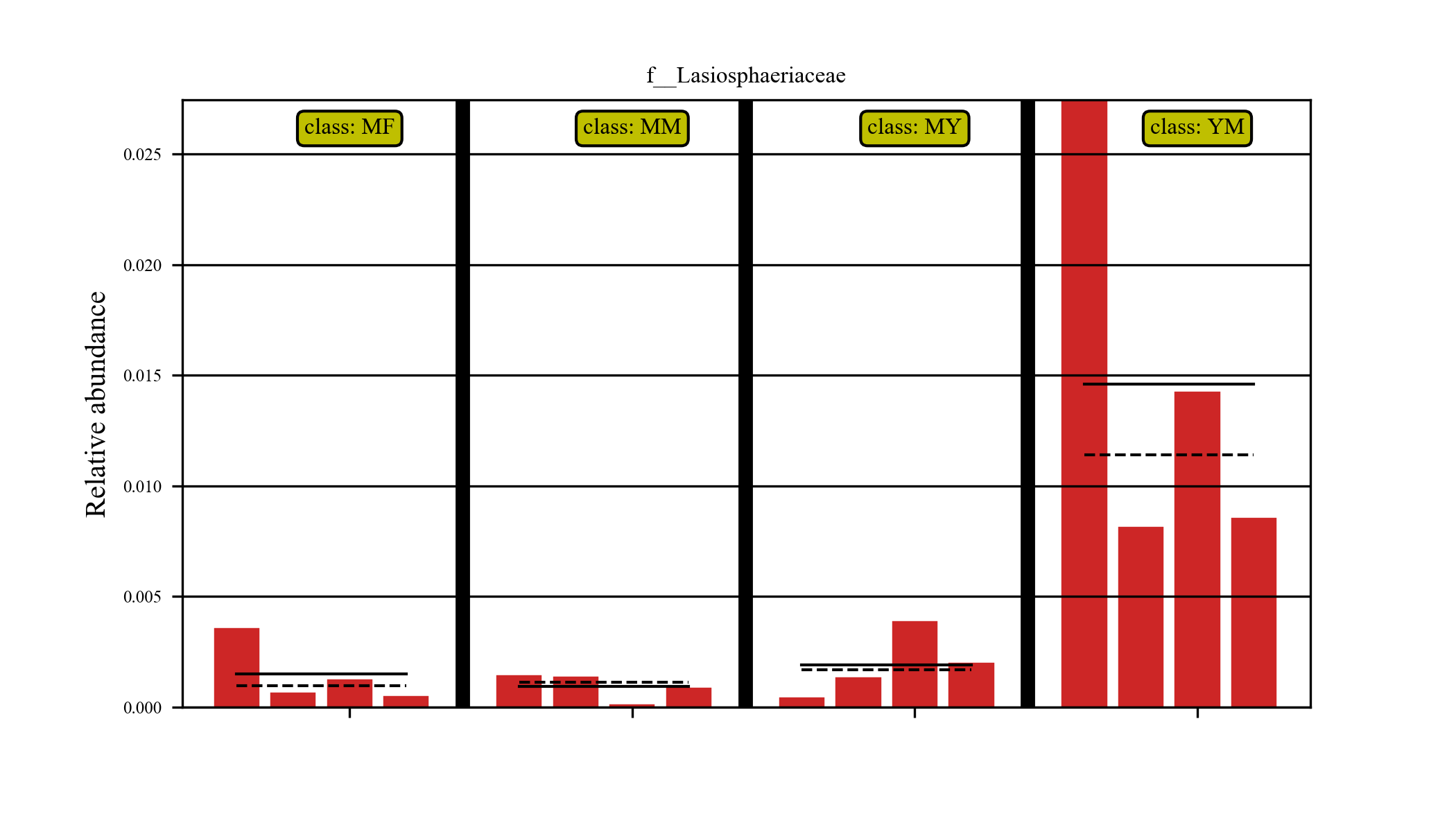

Supplement: Supplementary file 2 [file Data_Sheet_2.ZIP › Supplementary figure 2. fungal biomarker community/1_f__Lasiosphaeriaceae.png]

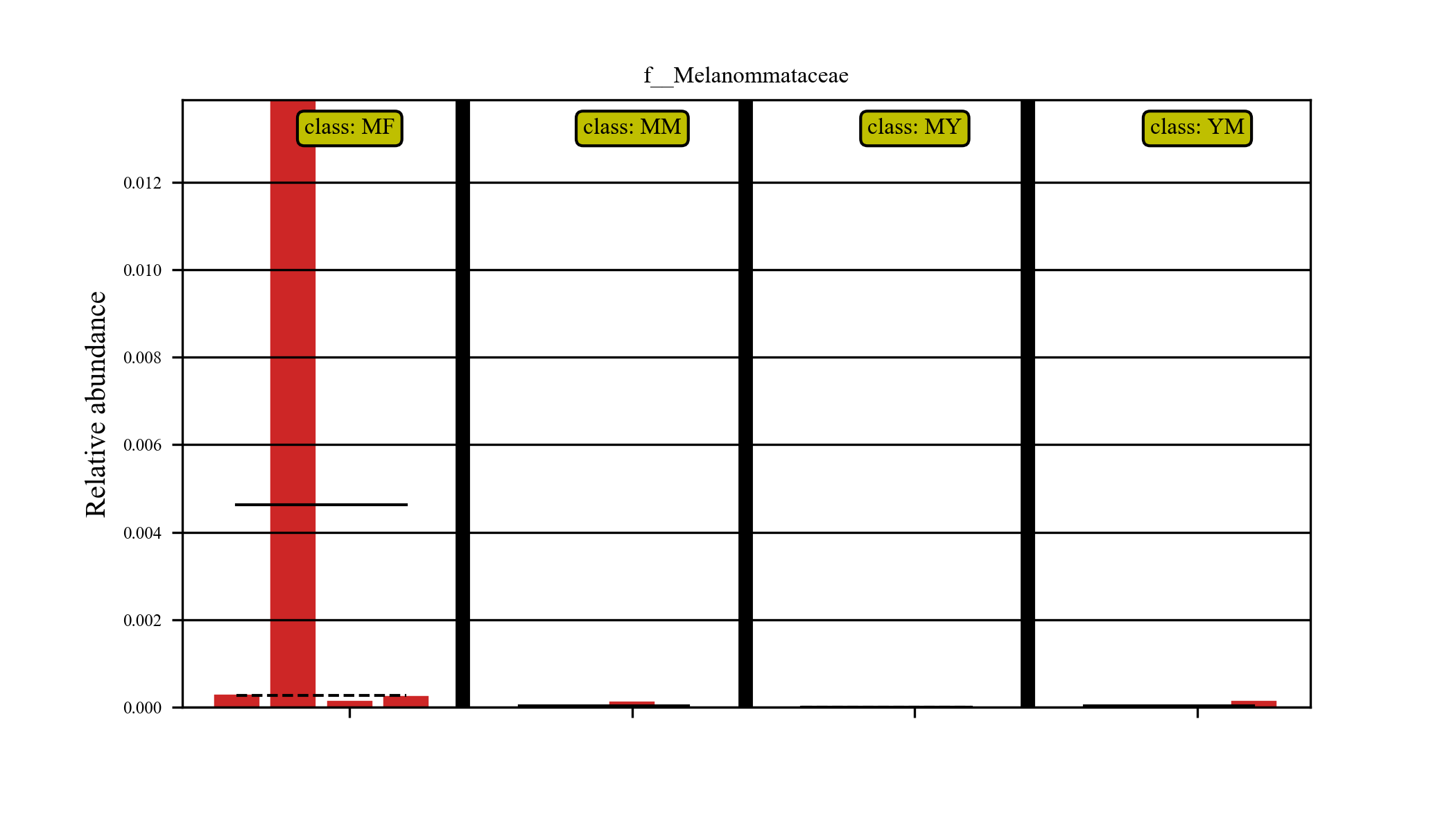

Supplement: Supplementary file 2 [file Data_Sheet_2.ZIP › Supplementary figure 2. fungal biomarker community/1_f__Melanommataceae.png]

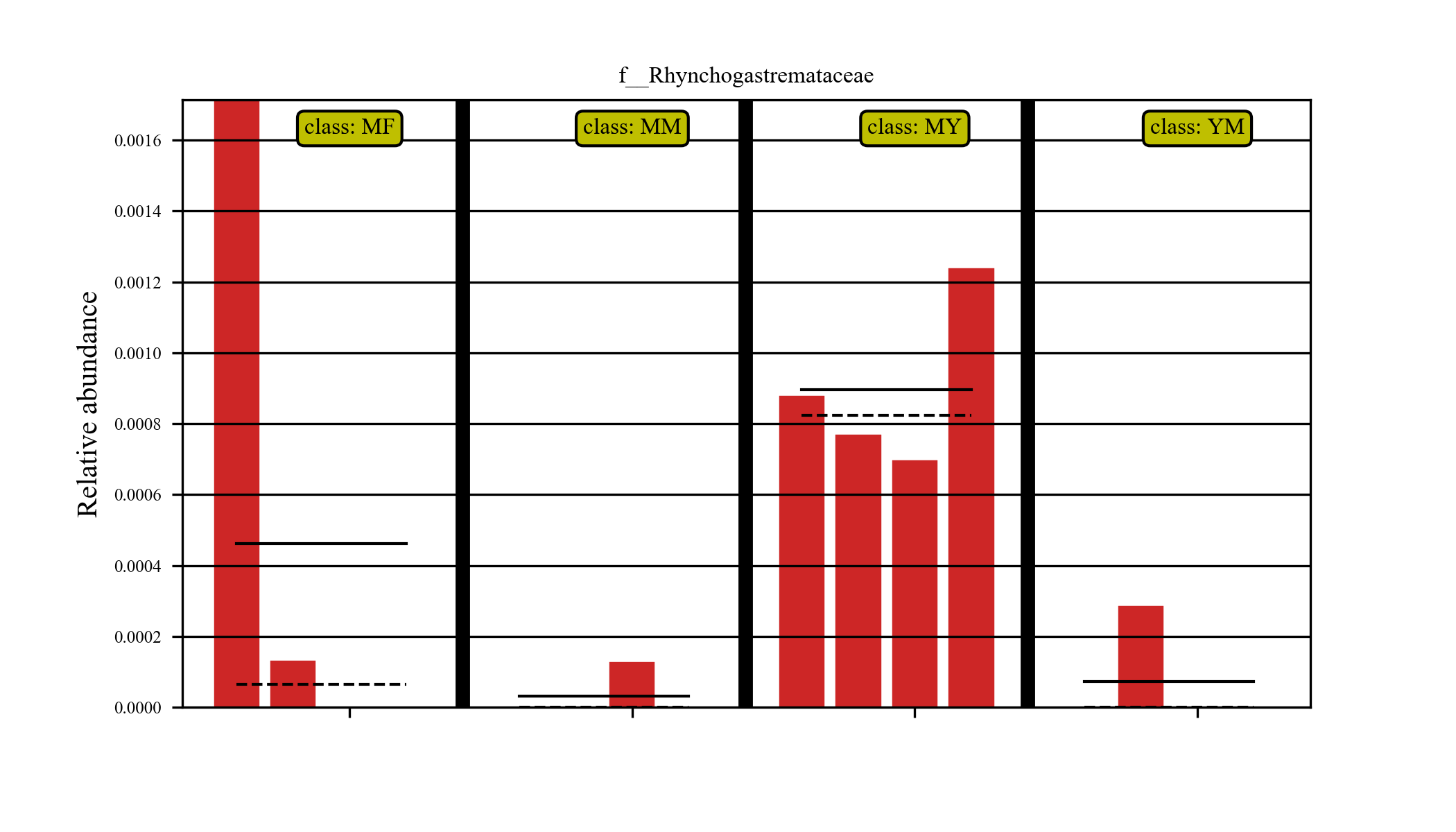

Supplement: Supplementary file 2 [file Data_Sheet_2.ZIP › Supplementary figure 2. fungal biomarker community/1_f__Rhynchogastremataceae.png]

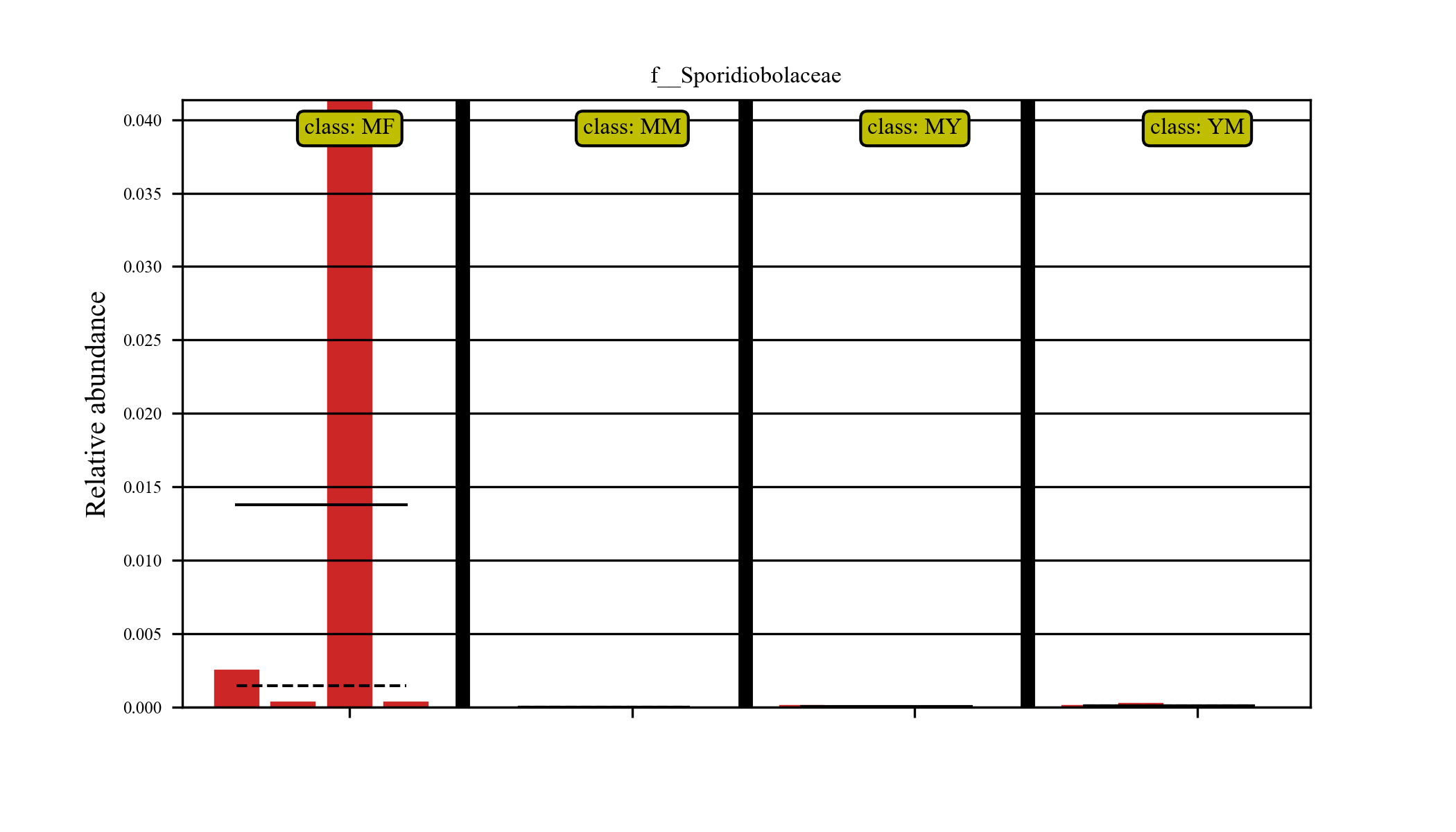

Supplement: Supplementary file 2 [file Data_Sheet_2.ZIP › Supplementary figure 2. fungal biomarker community/1_f__Sporidiobolaceae.png]

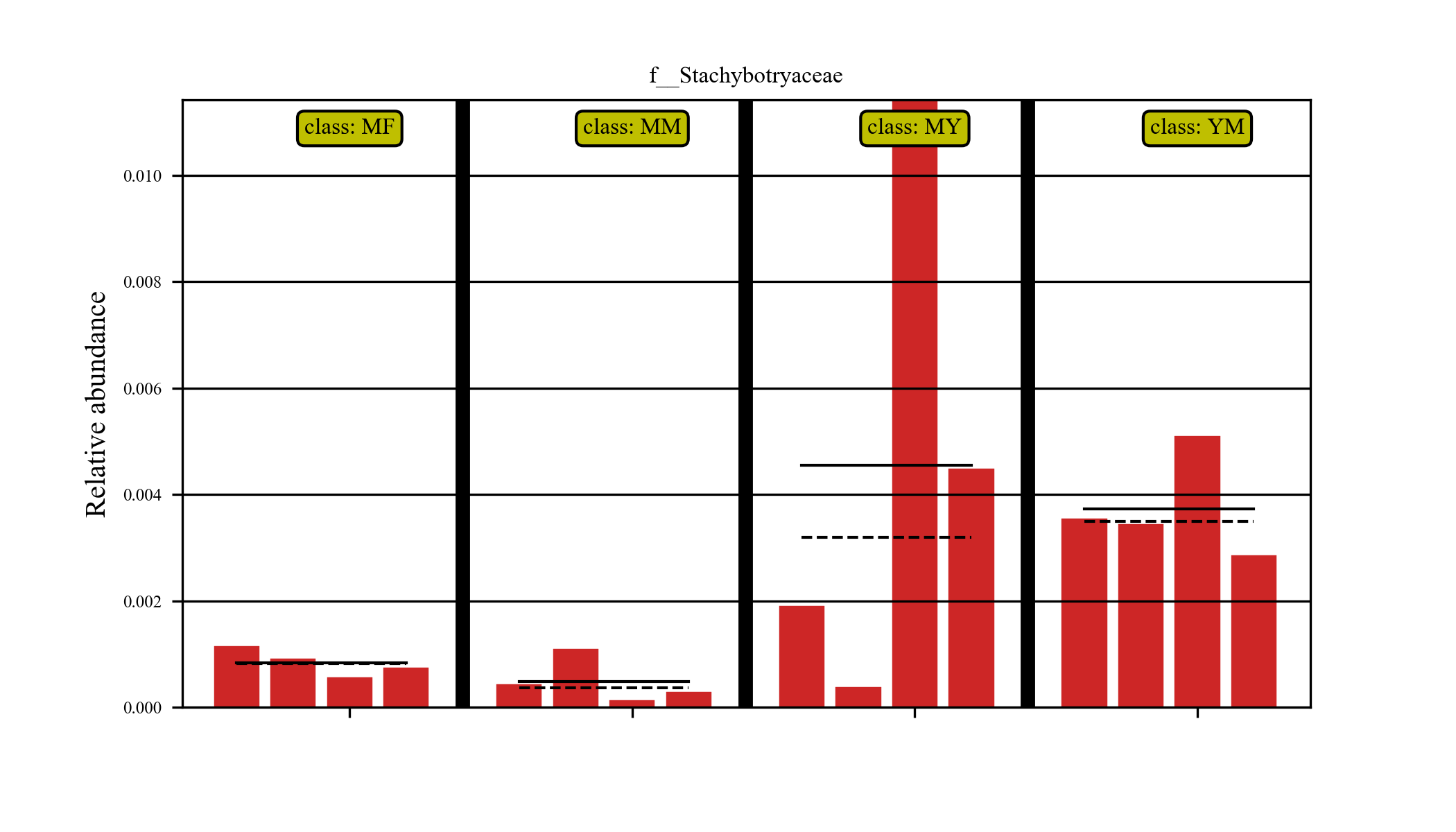

Supplement: Supplementary file 2 [file Data_Sheet_2.ZIP › Supplementary figure 2. fungal biomarker community/1_f__Stachybotryaceae.png]

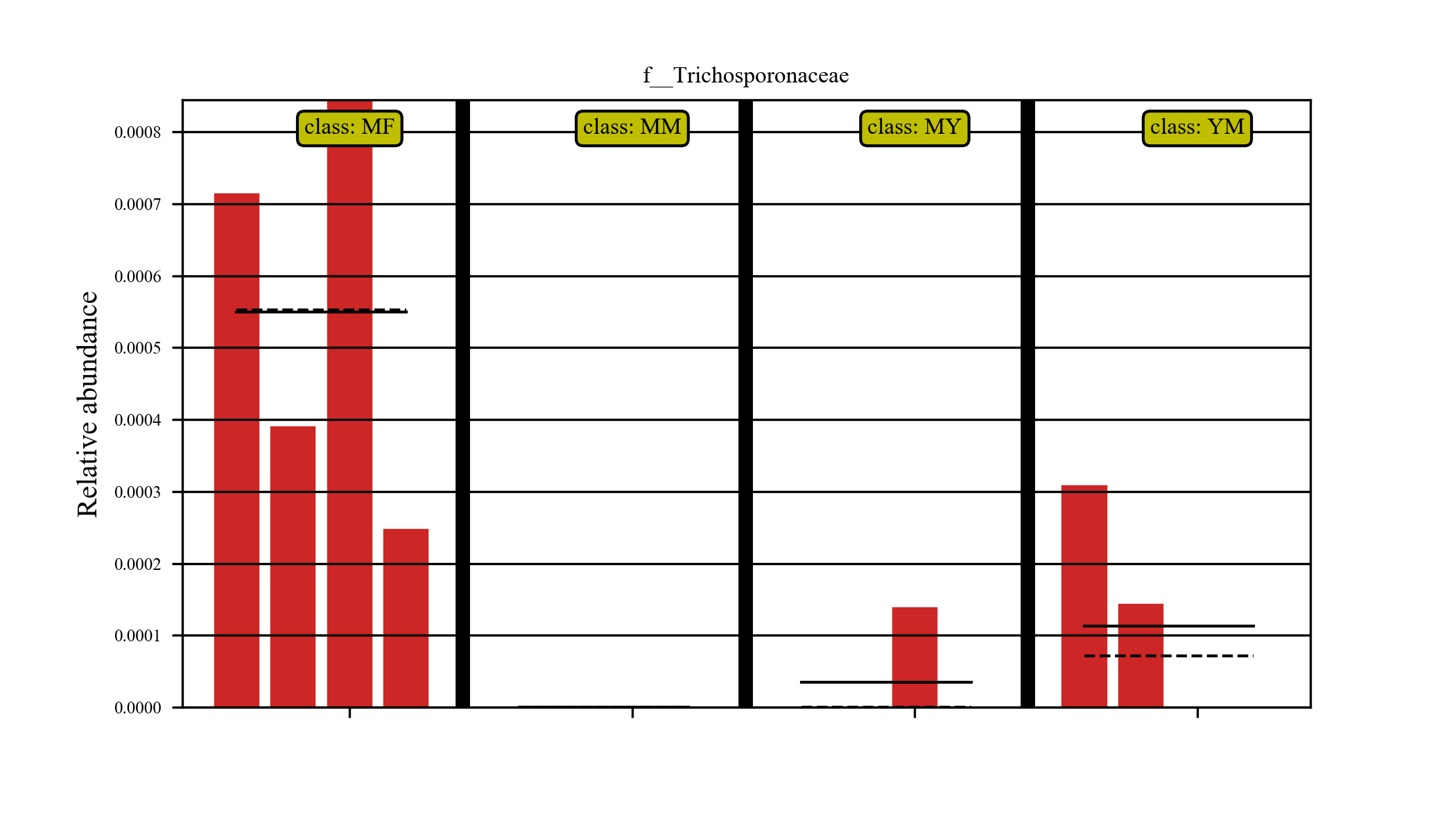

Supplement: Supplementary file 2 [file Data_Sheet_2.ZIP › Supplementary figure 2. fungal biomarker community/1_f__Trichosporonaceae.png]

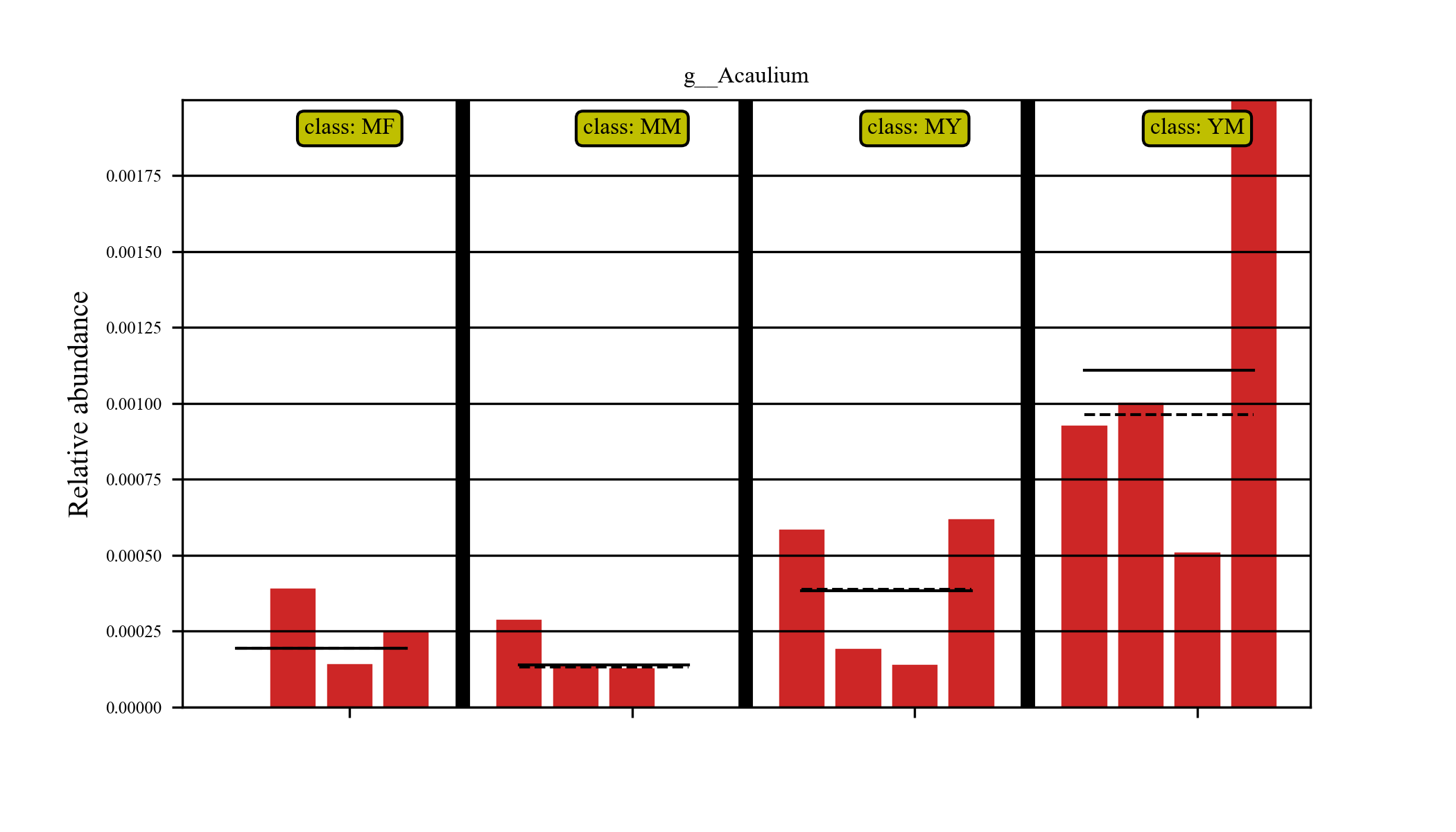

Supplement: Supplementary file 2 [file Data_Sheet_2.ZIP › Supplementary figure 2. fungal biomarker community/1_g__Acaulium.png]

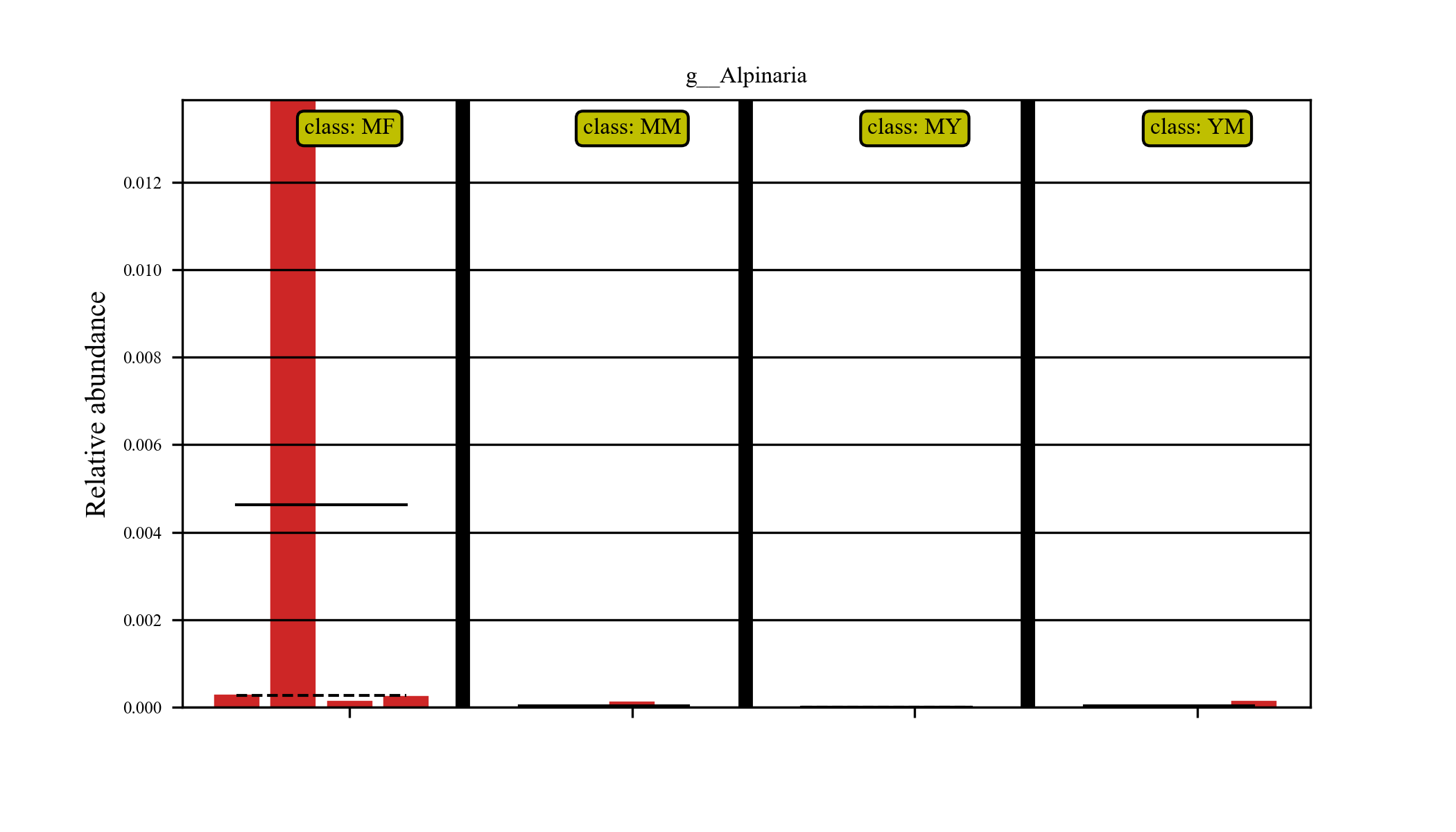

Supplement: Supplementary file 2 [file Data_Sheet_2.ZIP › Supplementary figure 2. fungal biomarker community/1_g__Alpinaria.png]

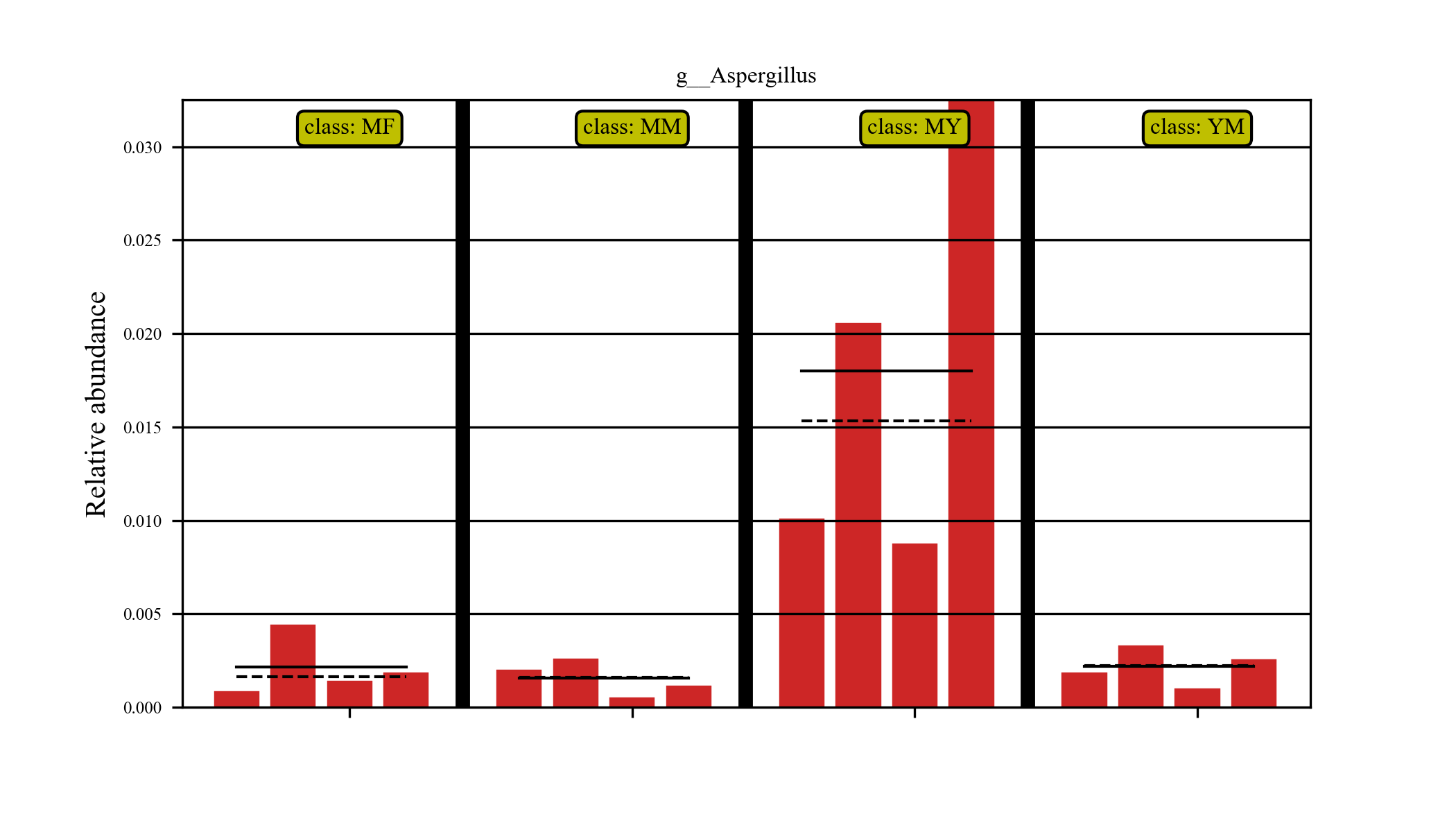

Supplement: Supplementary file 2 [file Data_Sheet_2.ZIP › Supplementary figure 2. fungal biomarker community/1_g__Aspergillus.png]

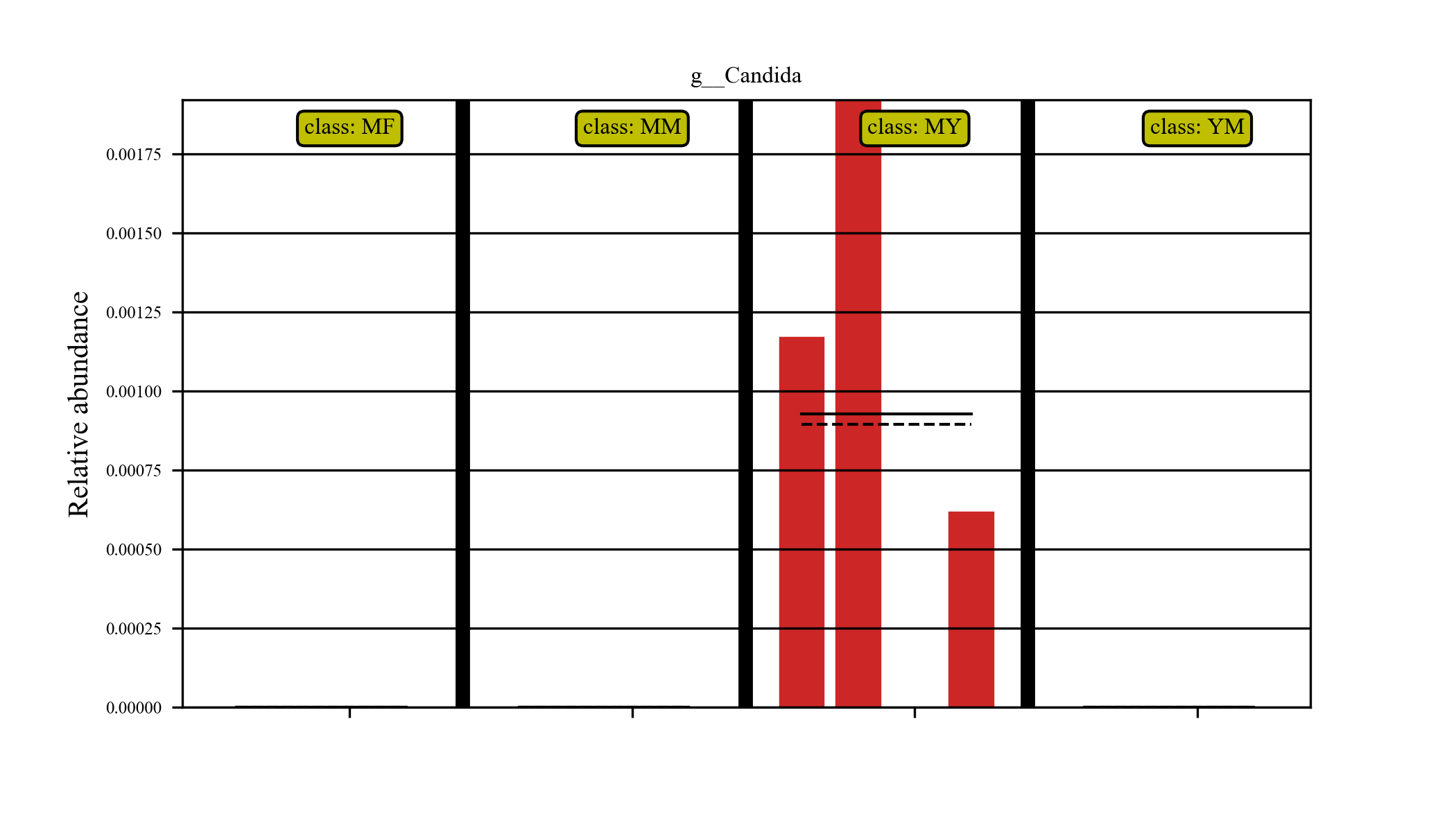

Supplement: Supplementary file 2 [file Data_Sheet_2.ZIP › Supplementary figure 2. fungal biomarker community/1_g__Candida.png]

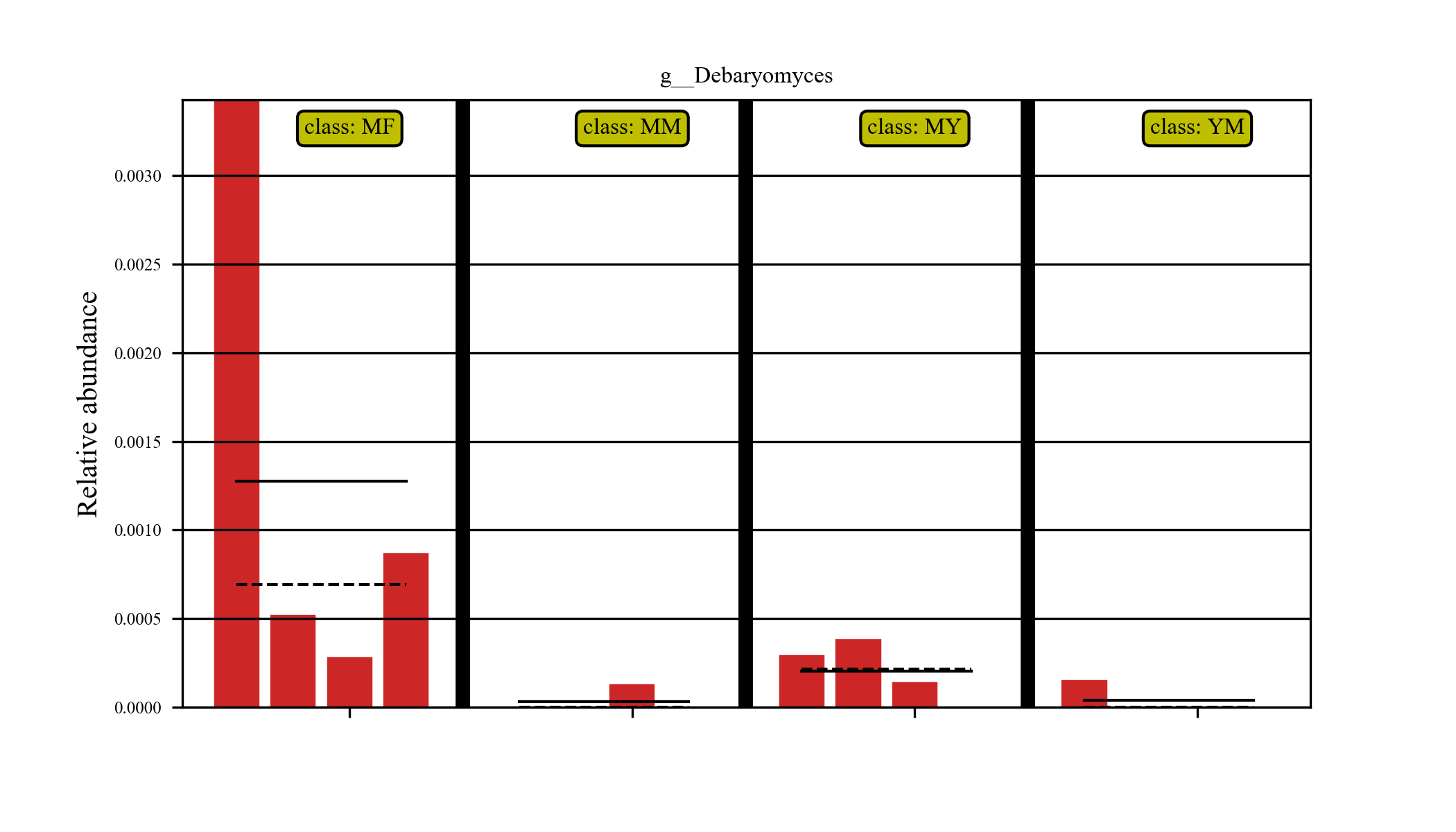

Supplement: Supplementary file 2 [file Data_Sheet_2.ZIP › Supplementary figure 2. fungal biomarker community/1_g__Debaryomyces.png]

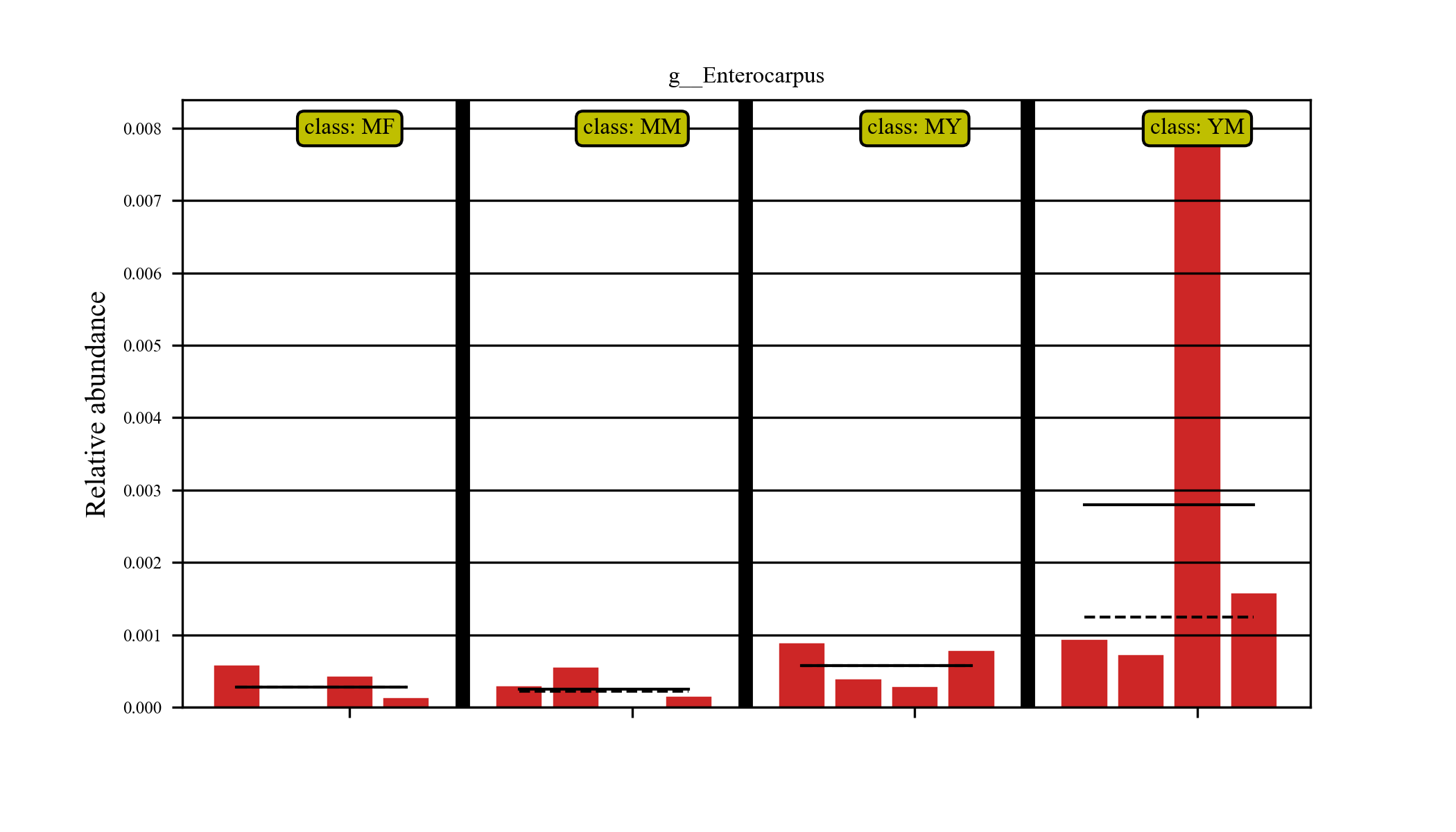

Supplement: Supplementary file 2 [file Data_Sheet_2.ZIP › Supplementary figure 2. fungal biomarker community/1_g__Enterocarpus.png]

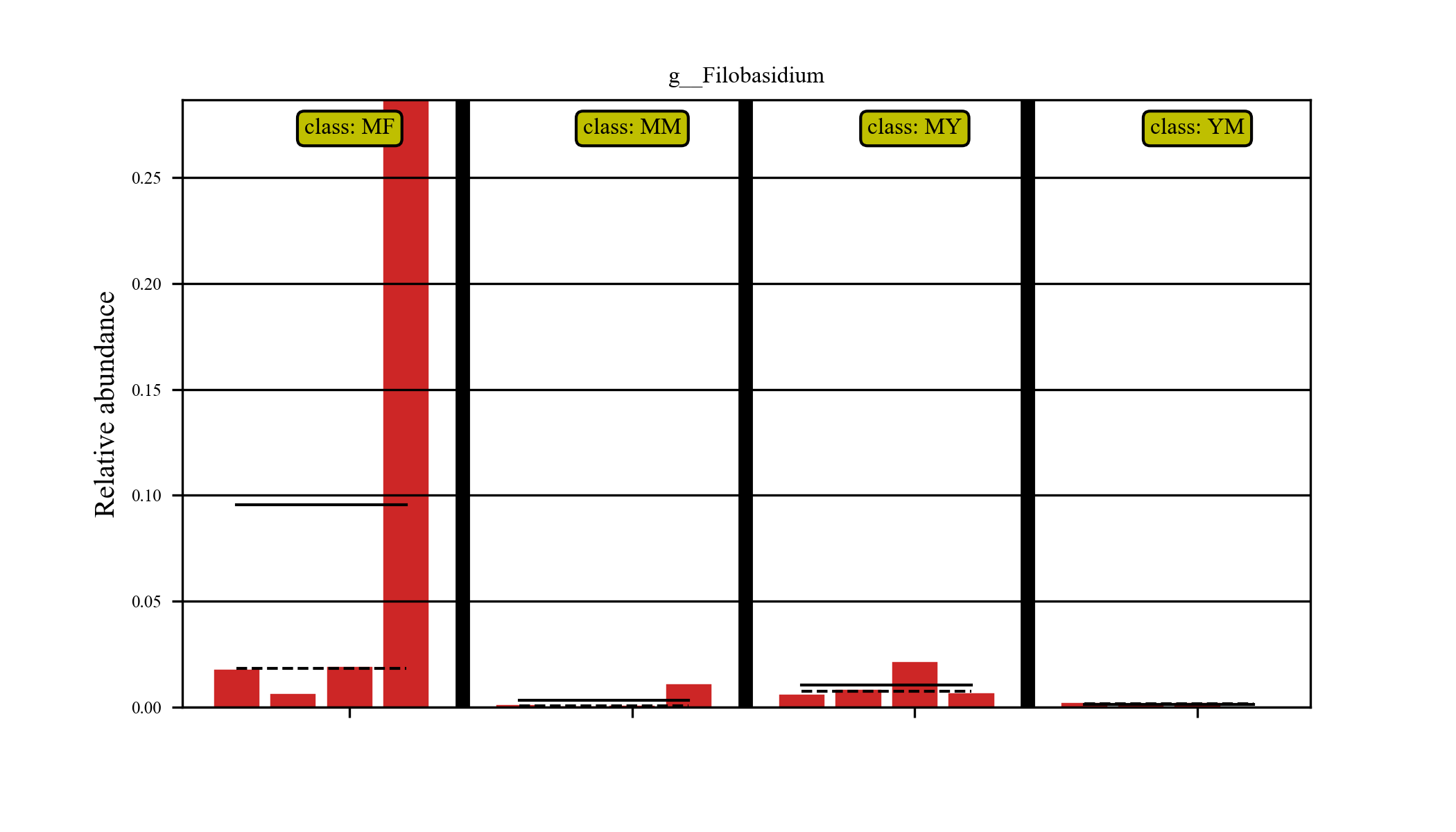

Supplement: Supplementary file 2 [file Data_Sheet_2.ZIP › Supplementary figure 2. fungal biomarker community/1_g__Filobasidium.png]

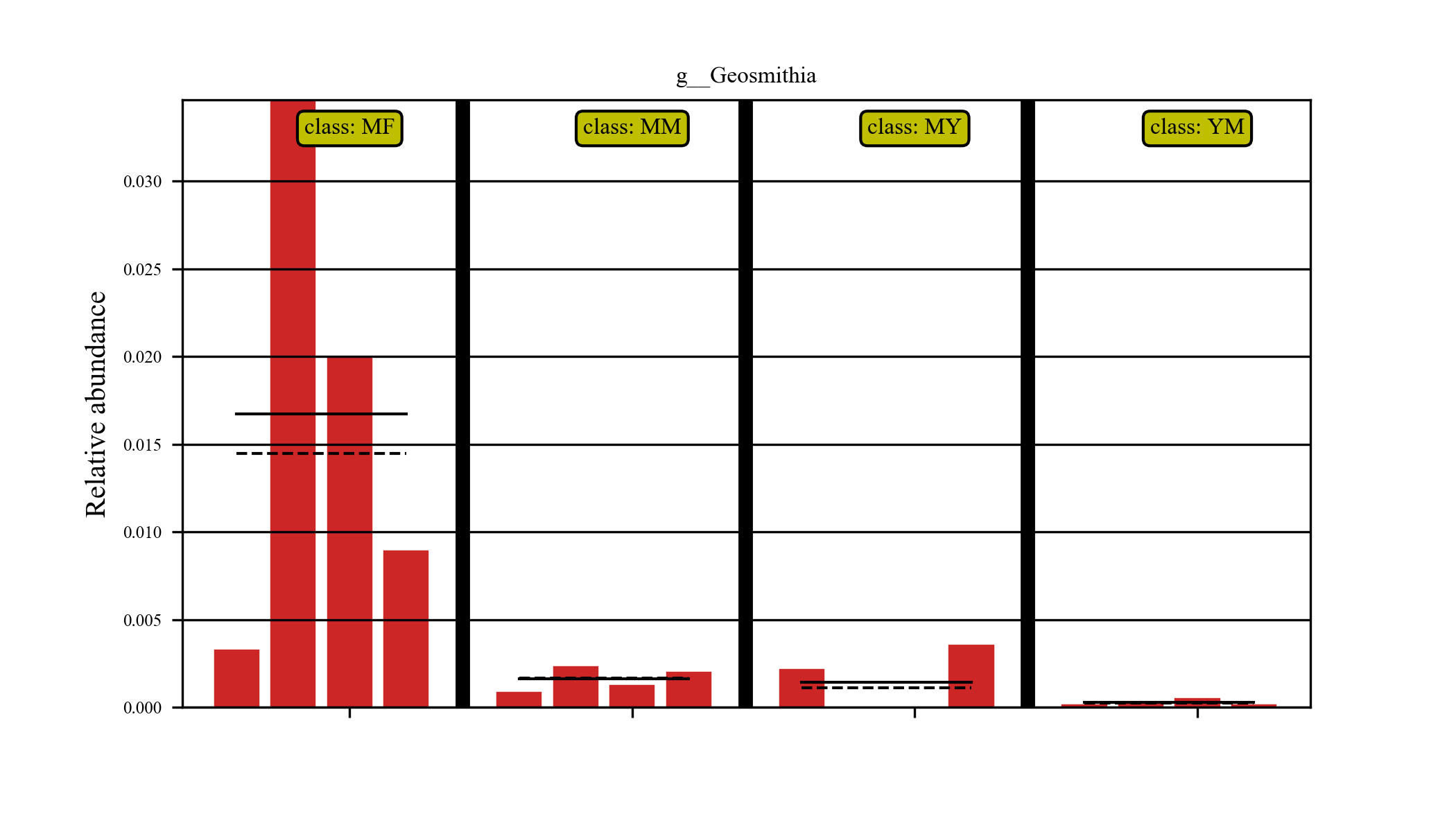

Supplement: Supplementary file 2 [file Data_Sheet_2.ZIP › Supplementary figure 2. fungal biomarker community/1_g__Geosmithia.png]
