# Supplementary figures and images for: Effects of aftercrop tomato and maize on the soil microenvironment and microbial diversity in a long-term cotton continuous cropping field (part 2 of 2)
Source: Front Microbiol. 2024 Jul 19;15:1410219. doi: 10.3389/fmicb.2024.1410219 (PMC11295657; doi:10.3389/fmicb.2024.1410219)

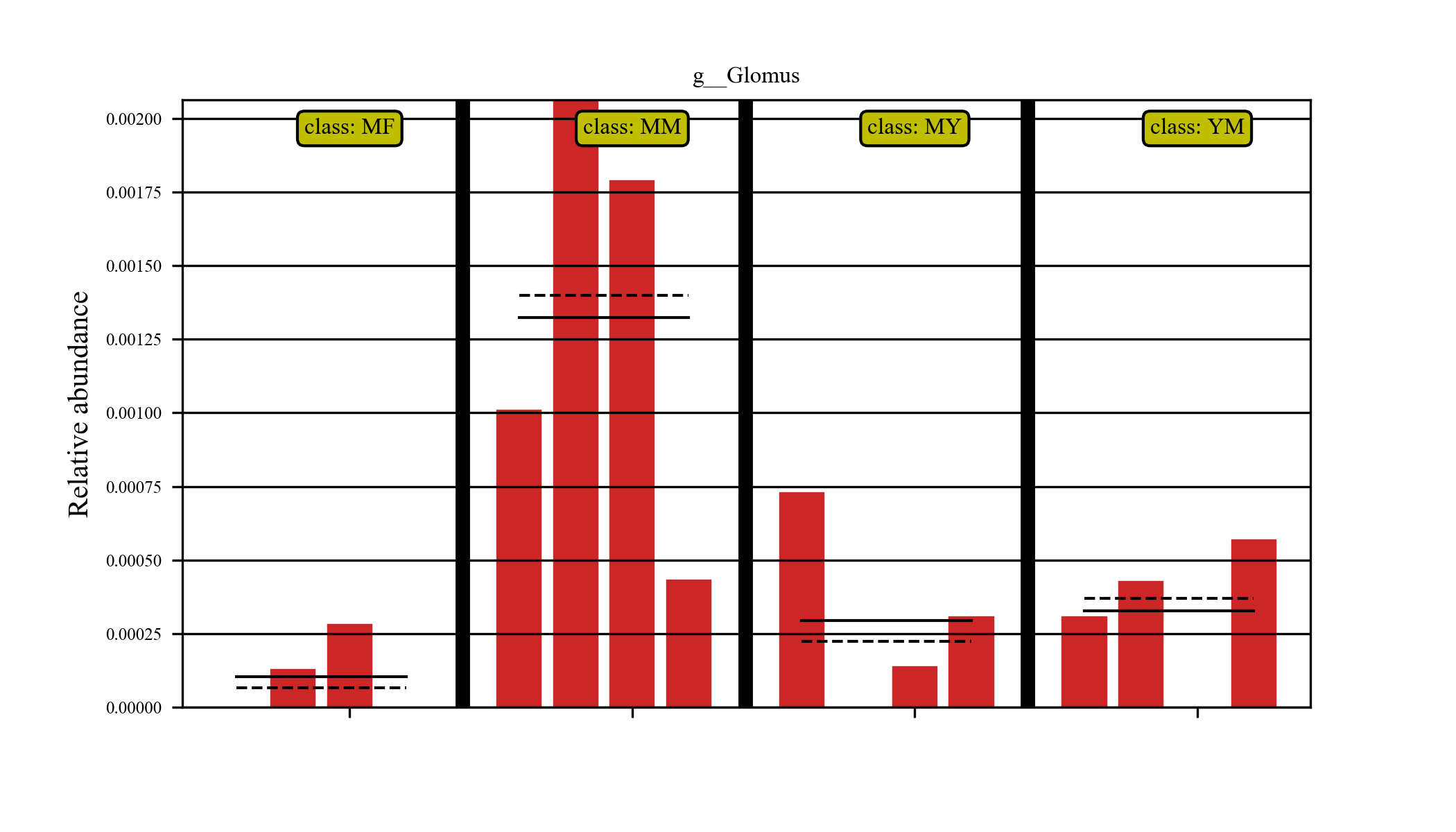

Supplement: Supplementary file 2 [file Data_Sheet_2.ZIP › Supplementary figure 2. fungal biomarker community/1_g__Glomus.png]

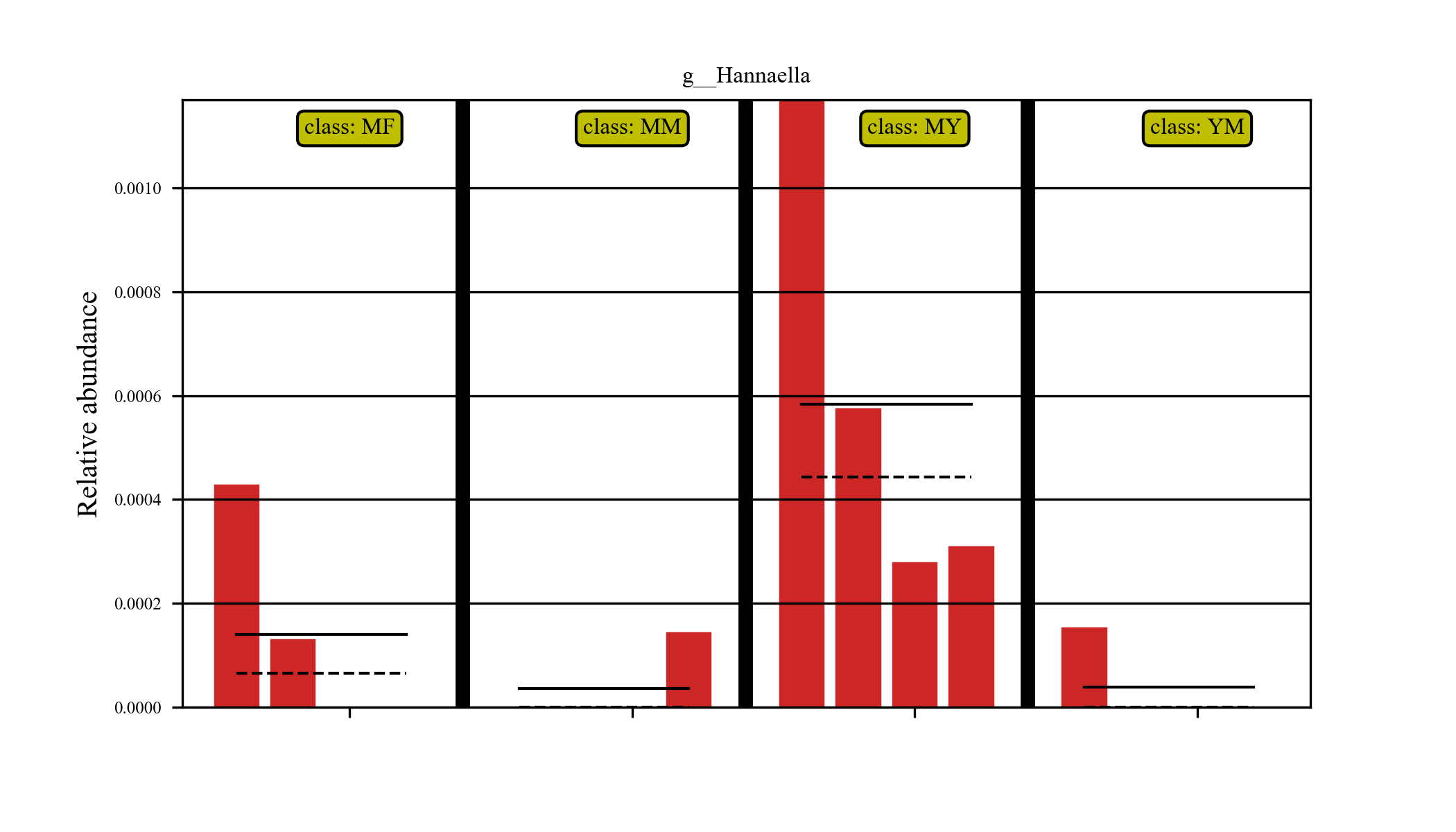

Supplement: Supplementary file 2 [file Data_Sheet_2.ZIP › Supplementary figure 2. fungal biomarker community/1_g__Hannaella.png]

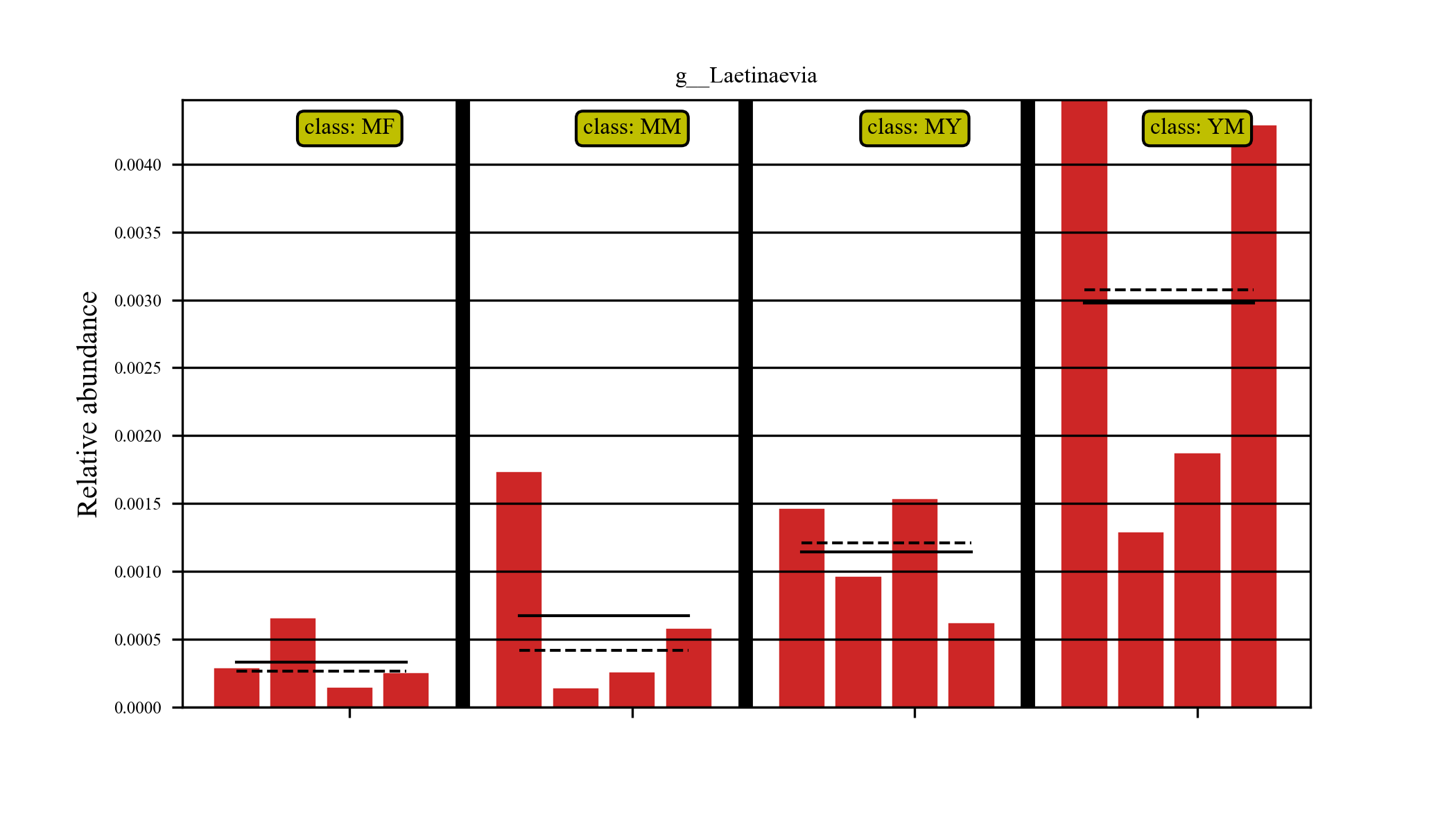

Supplement: Supplementary file 2 [file Data_Sheet_2.ZIP › Supplementary figure 2. fungal biomarker community/1_g__Laetinaevia.png]

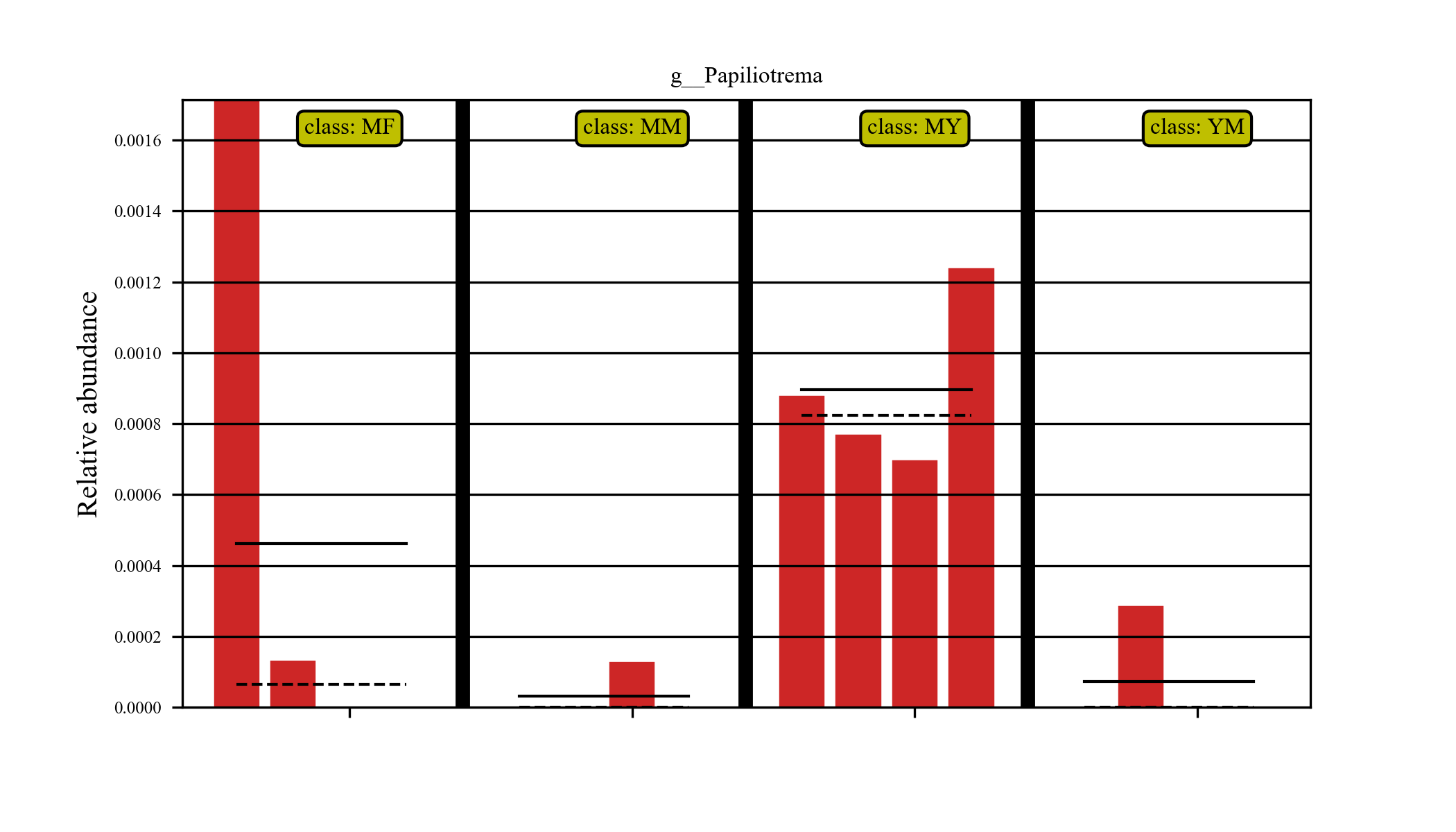

Supplement: Supplementary file 2 [file Data_Sheet_2.ZIP › Supplementary figure 2. fungal biomarker community/1_g__Papiliotrema.png]

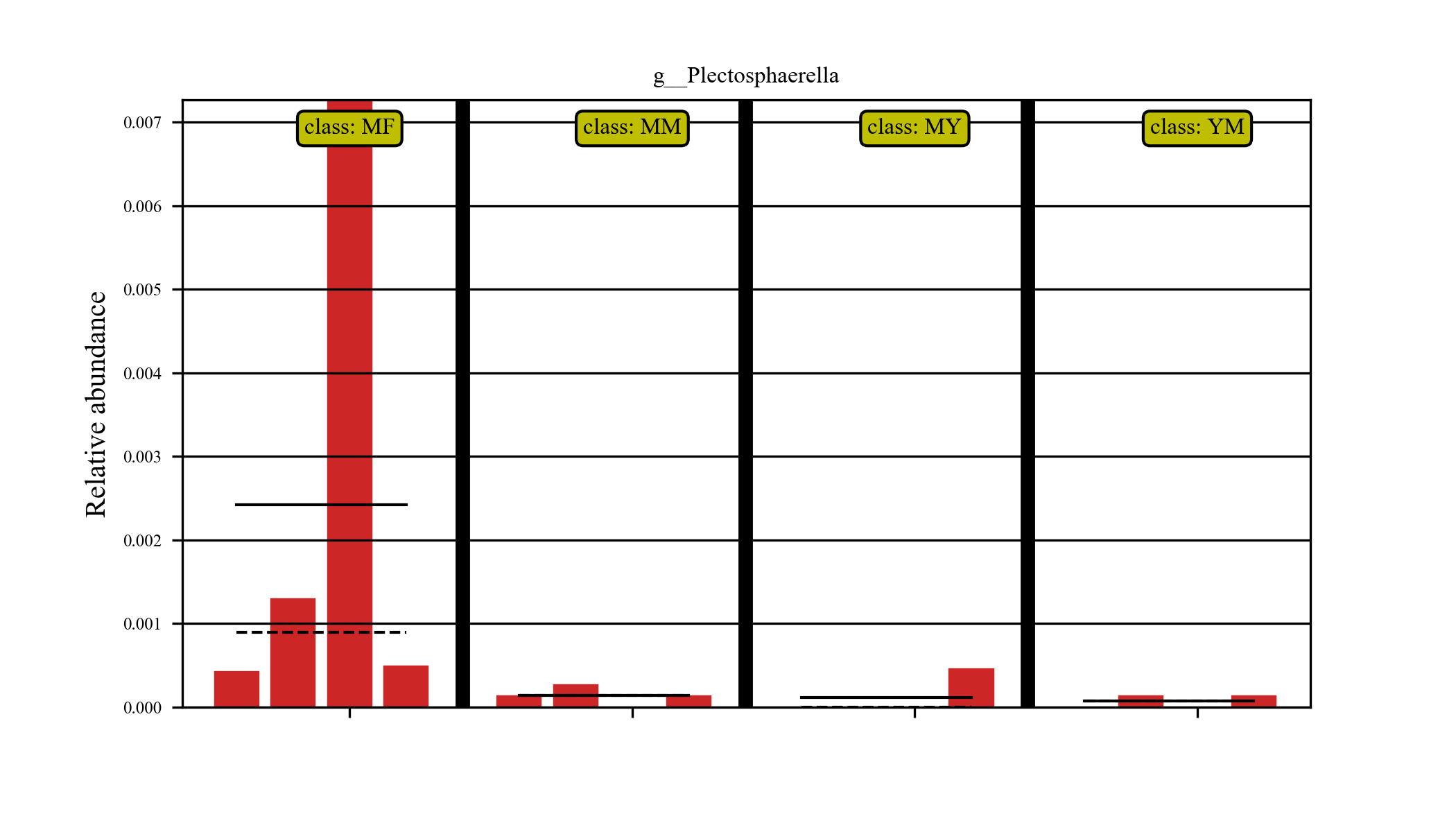

Supplement: Supplementary file 2 [file Data_Sheet_2.ZIP › Supplementary figure 2. fungal biomarker community/1_g__Plectosphaerella.png]

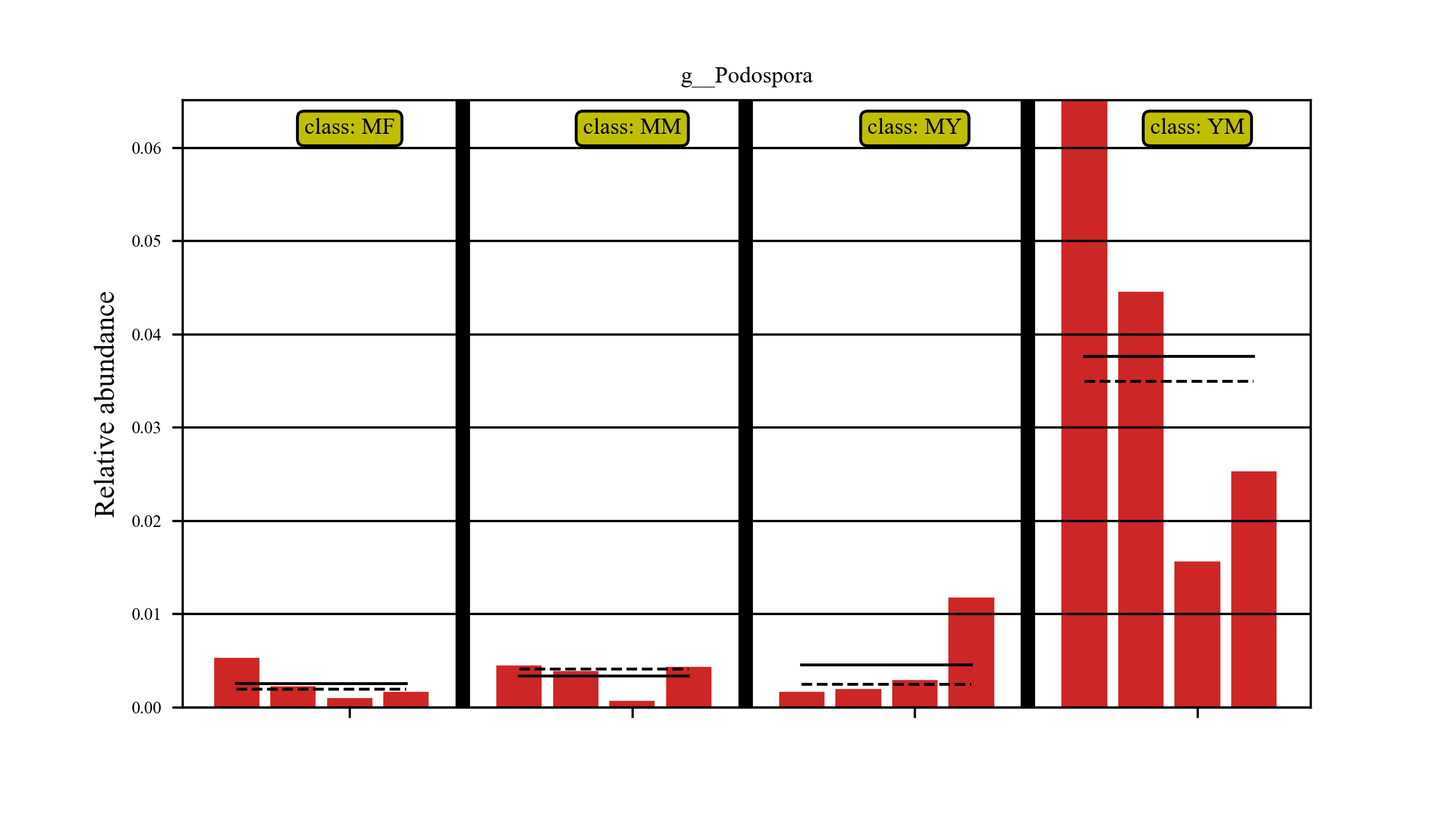

Supplement: Supplementary file 2 [file Data_Sheet_2.ZIP › Supplementary figure 2. fungal biomarker community/1_g__Podospora.png]

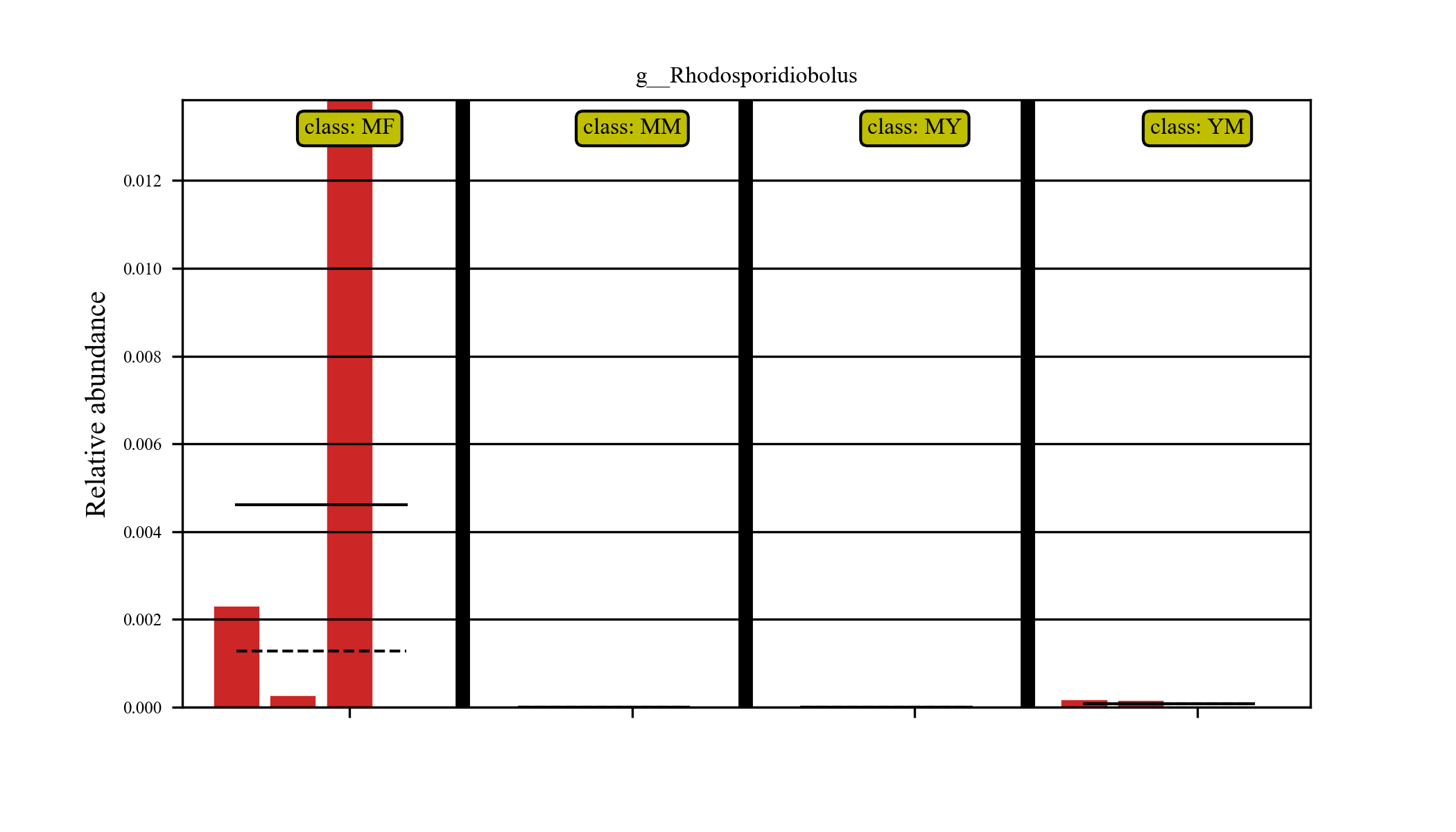

Supplement: Supplementary file 2 [file Data_Sheet_2.ZIP › Supplementary figure 2. fungal biomarker community/1_g__Rhodosporidiobolus.png]

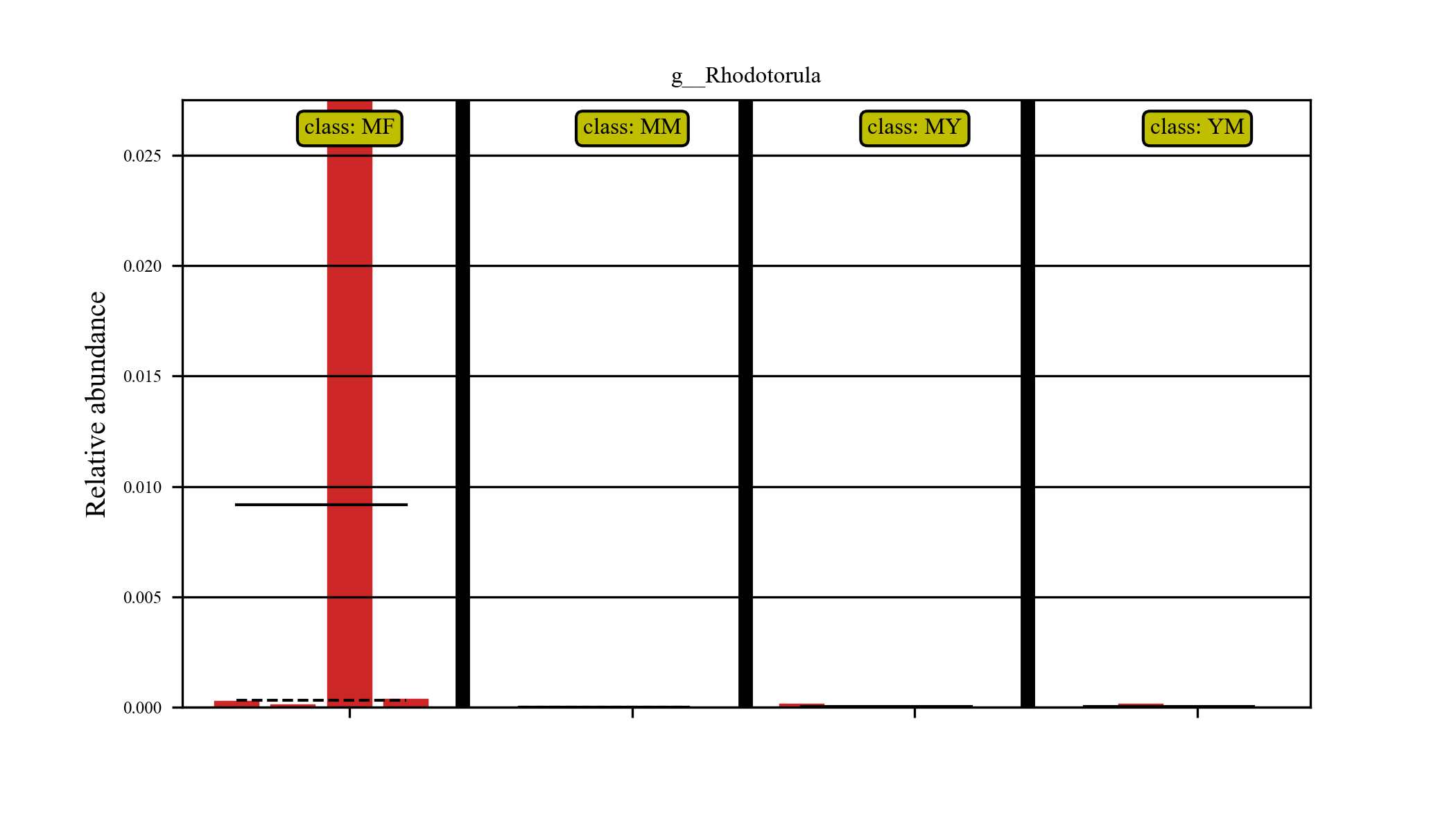

Supplement: Supplementary file 2 [file Data_Sheet_2.ZIP › Supplementary figure 2. fungal biomarker community/1_g__Rhodotorula.png]

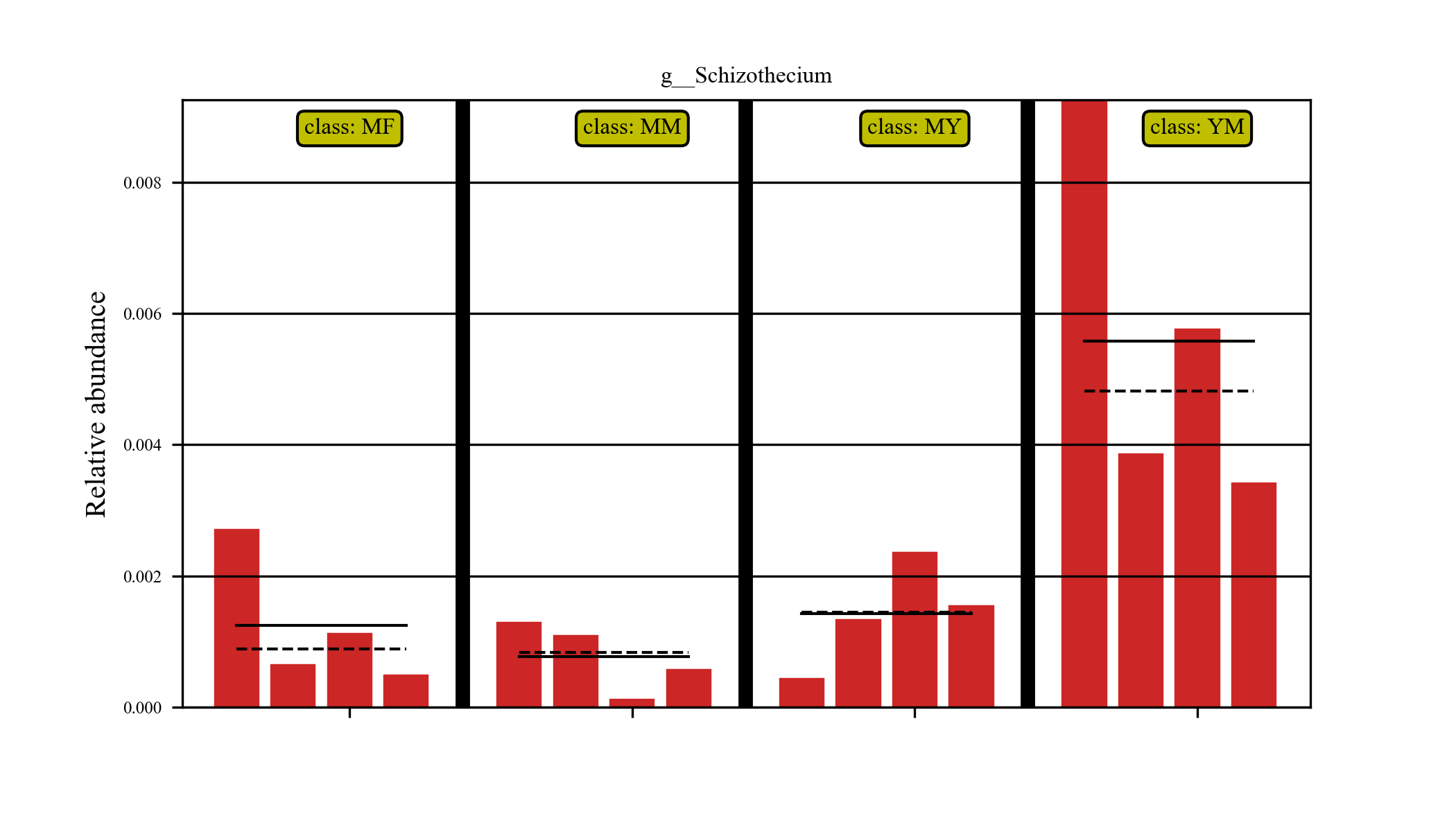

Supplement: Supplementary file 2 [file Data_Sheet_2.ZIP › Supplementary figure 2. fungal biomarker community/1_g__Schizothecium.png]

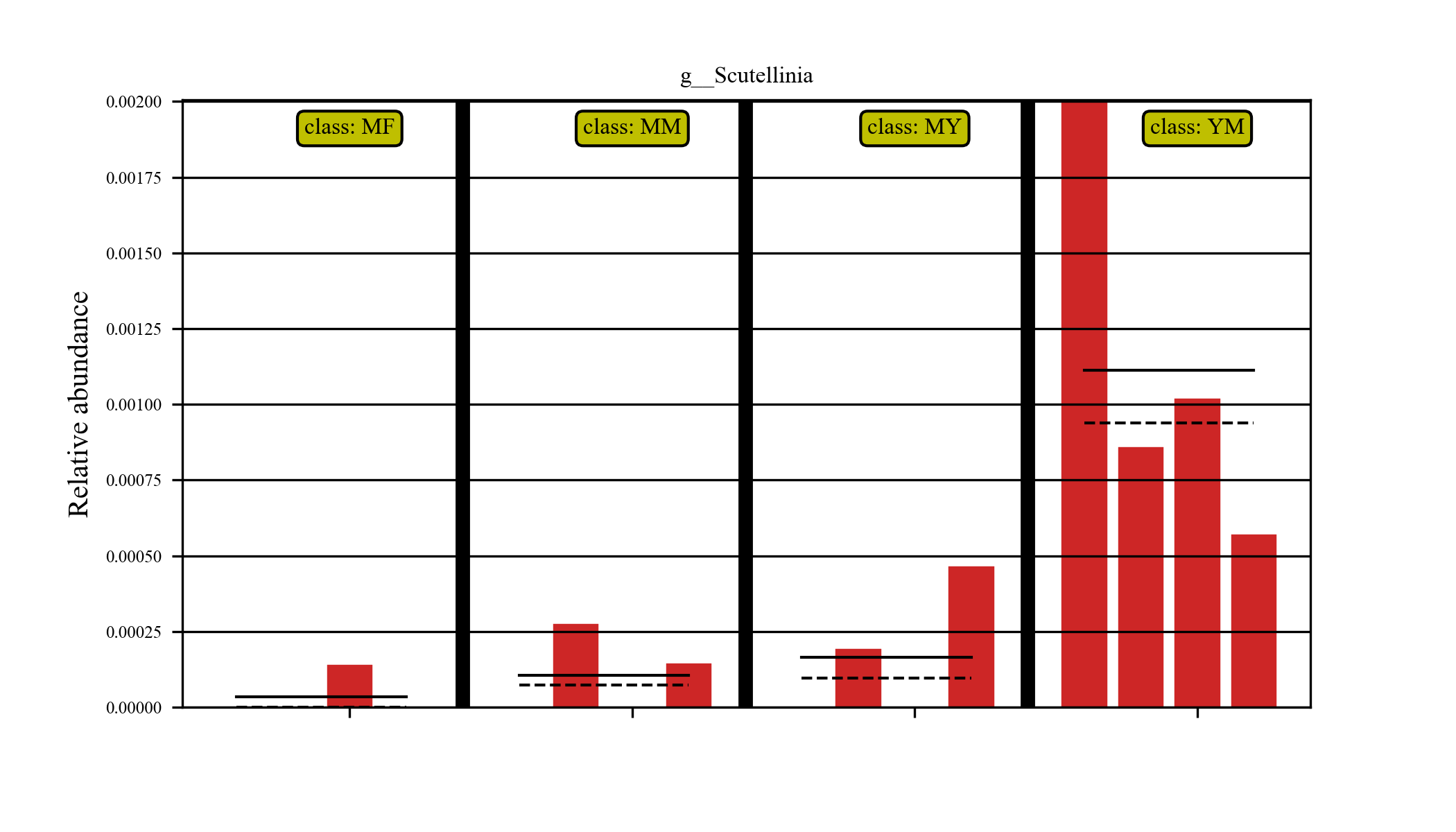

Supplement: Supplementary file 2 [file Data_Sheet_2.ZIP › Supplementary figure 2. fungal biomarker community/1_g__Scutellinia.png]

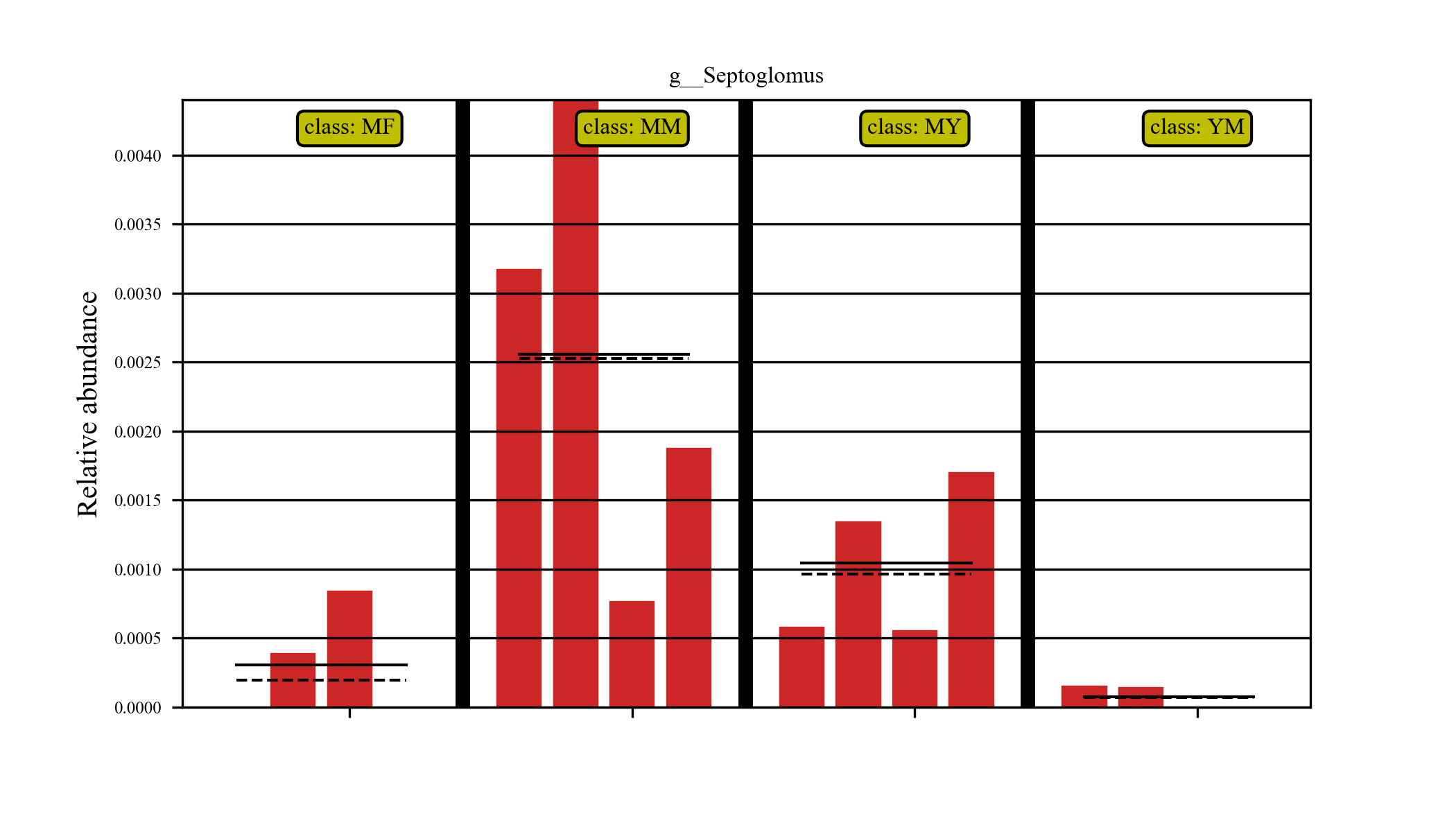

Supplement: Supplementary file 2 [file Data_Sheet_2.ZIP › Supplementary figure 2. fungal biomarker community/1_g__Septoglomus.png]

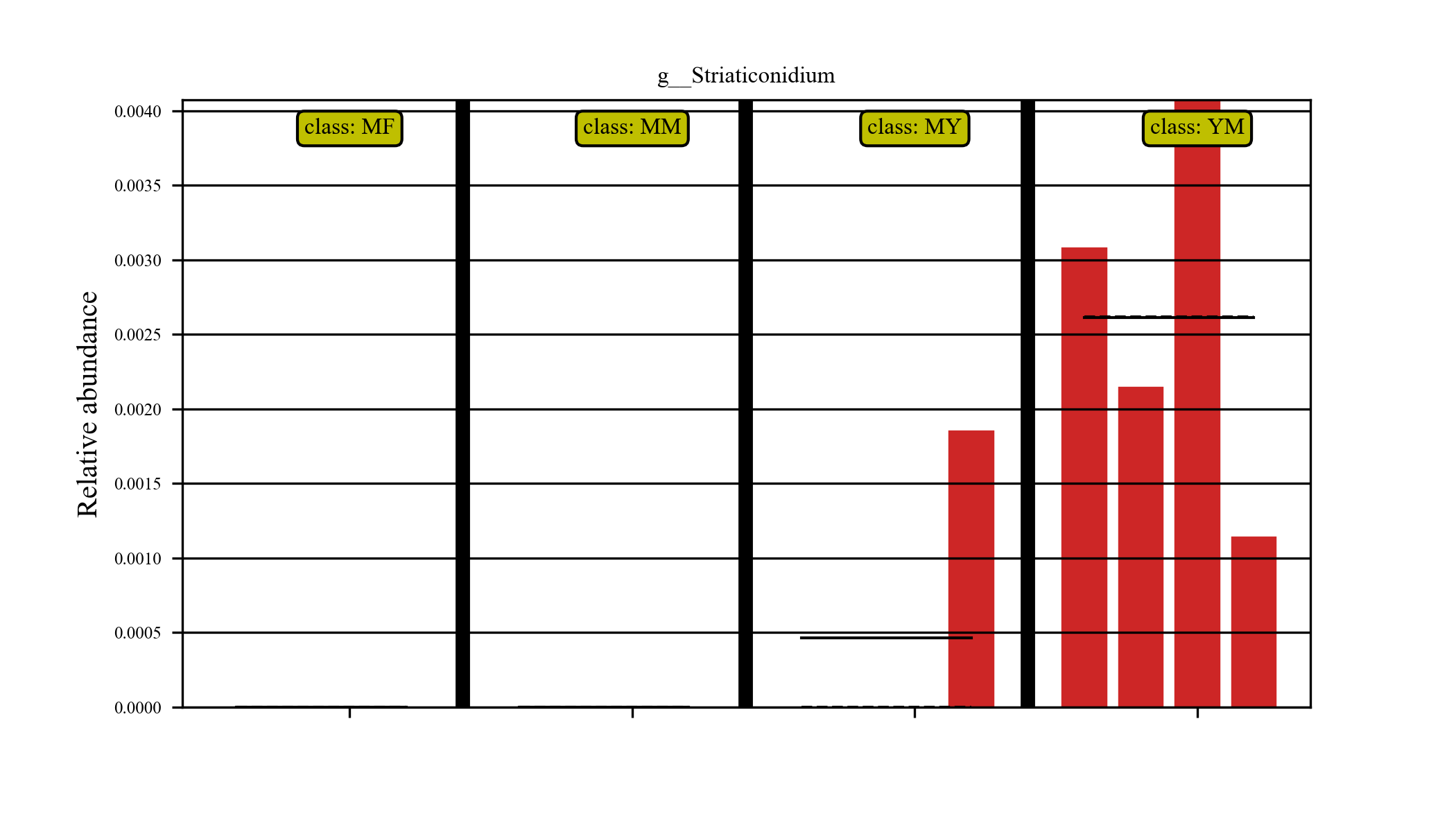

Supplement: Supplementary file 2 [file Data_Sheet_2.ZIP › Supplementary figure 2. fungal biomarker community/1_g__Striaticonidium.png]

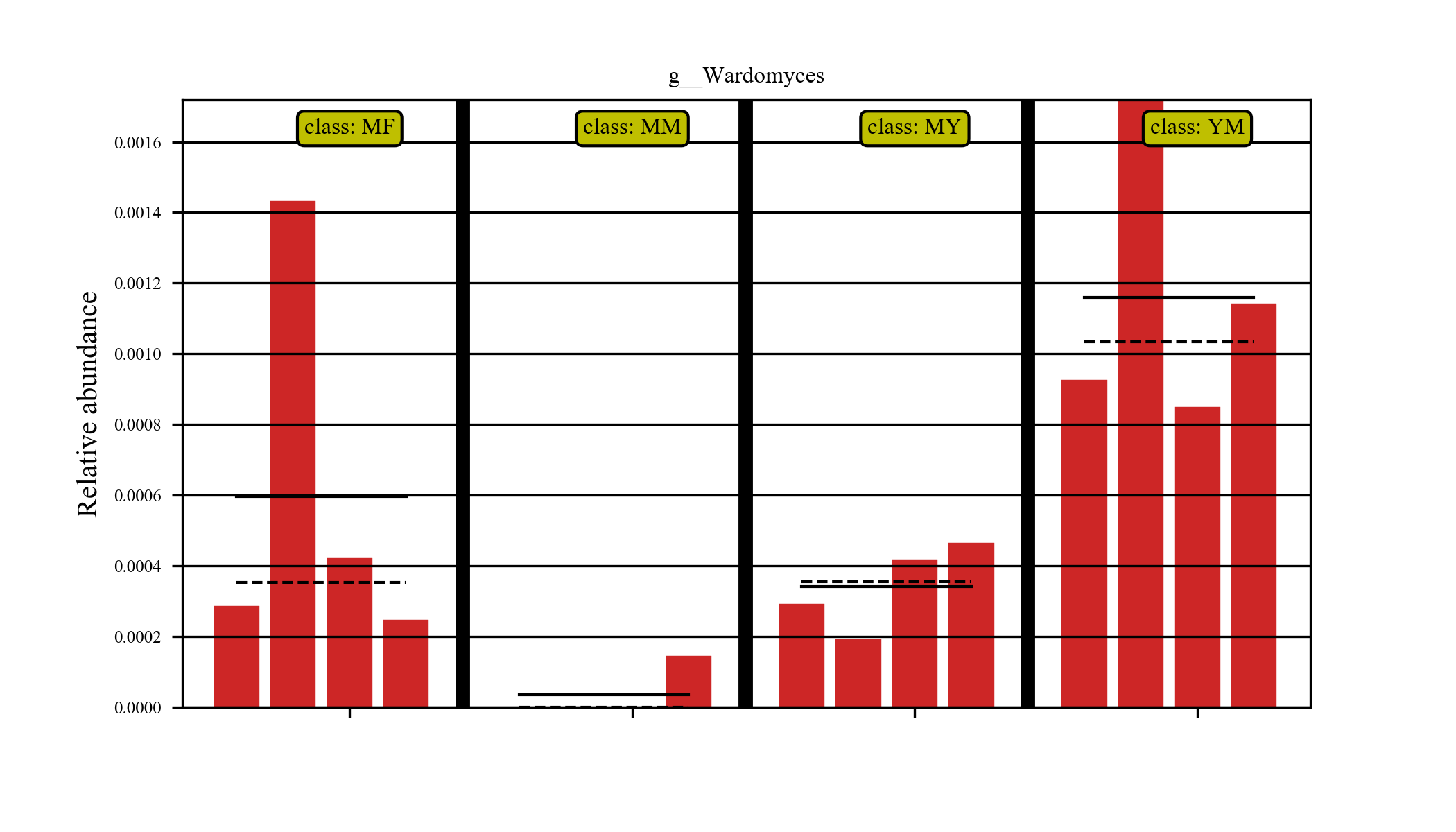

Supplement: Supplementary file 2 [file Data_Sheet_2.ZIP › Supplementary figure 2. fungal biomarker community/1_g__Wardomyces.png]

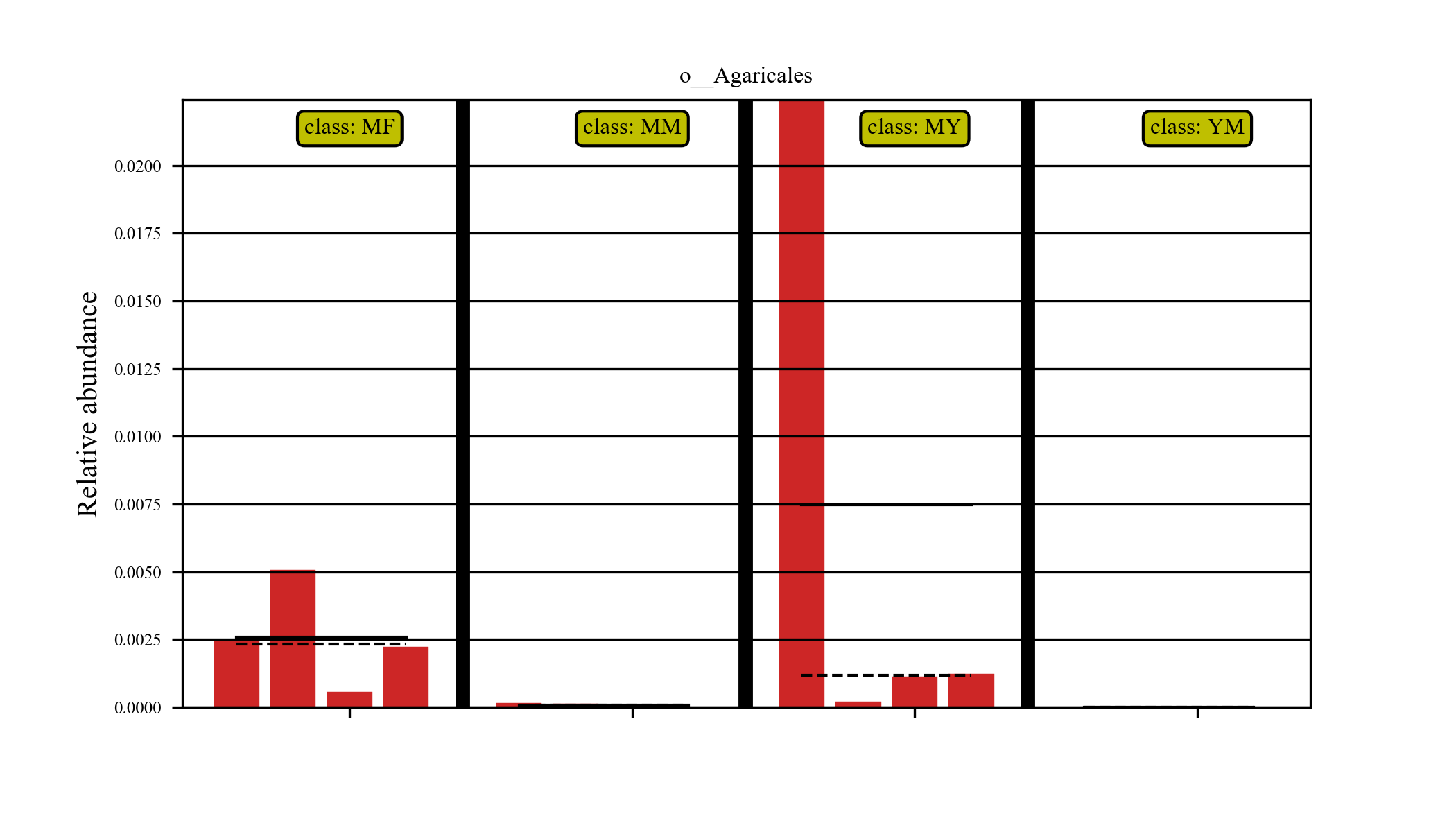

Supplement: Supplementary file 2 [file Data_Sheet_2.ZIP › Supplementary figure 2. fungal biomarker community/1_o__Agaricales.png]

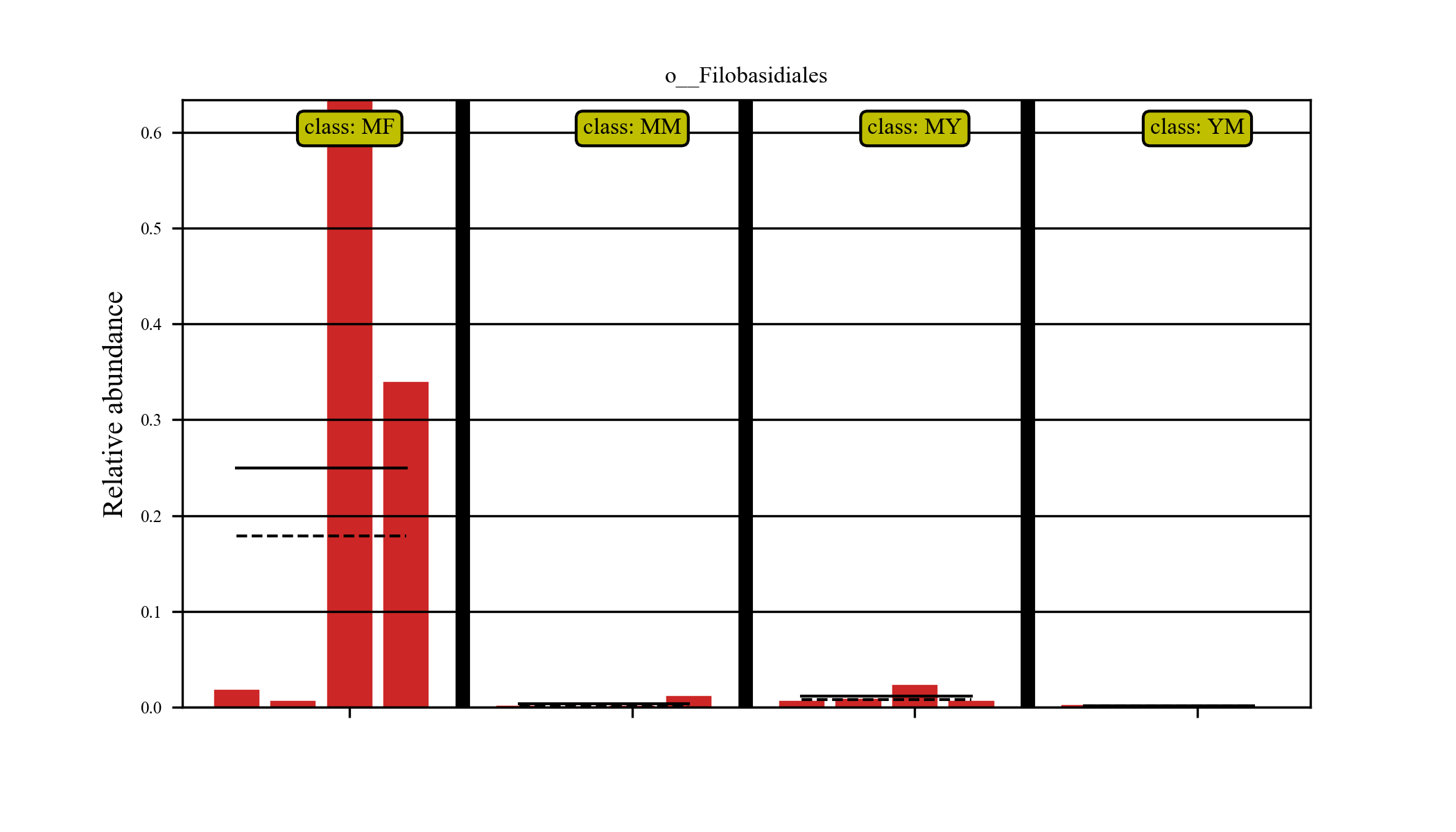

Supplement: Supplementary file 2 [file Data_Sheet_2.ZIP › Supplementary figure 2. fungal biomarker community/1_o__Filobasidiales.png]

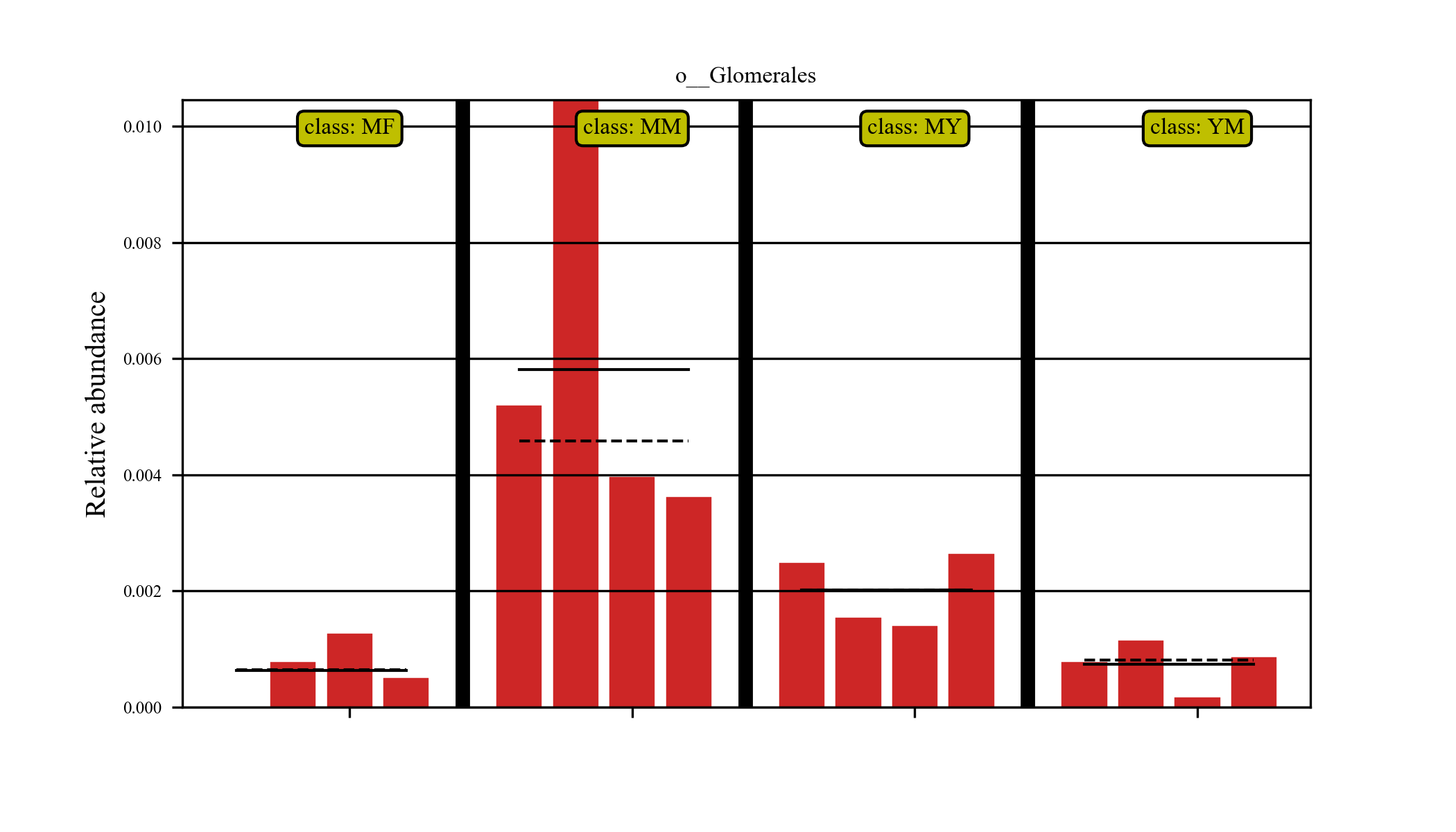

Supplement: Supplementary file 2 [file Data_Sheet_2.ZIP › Supplementary figure 2. fungal biomarker community/1_o__Glomerales.png]

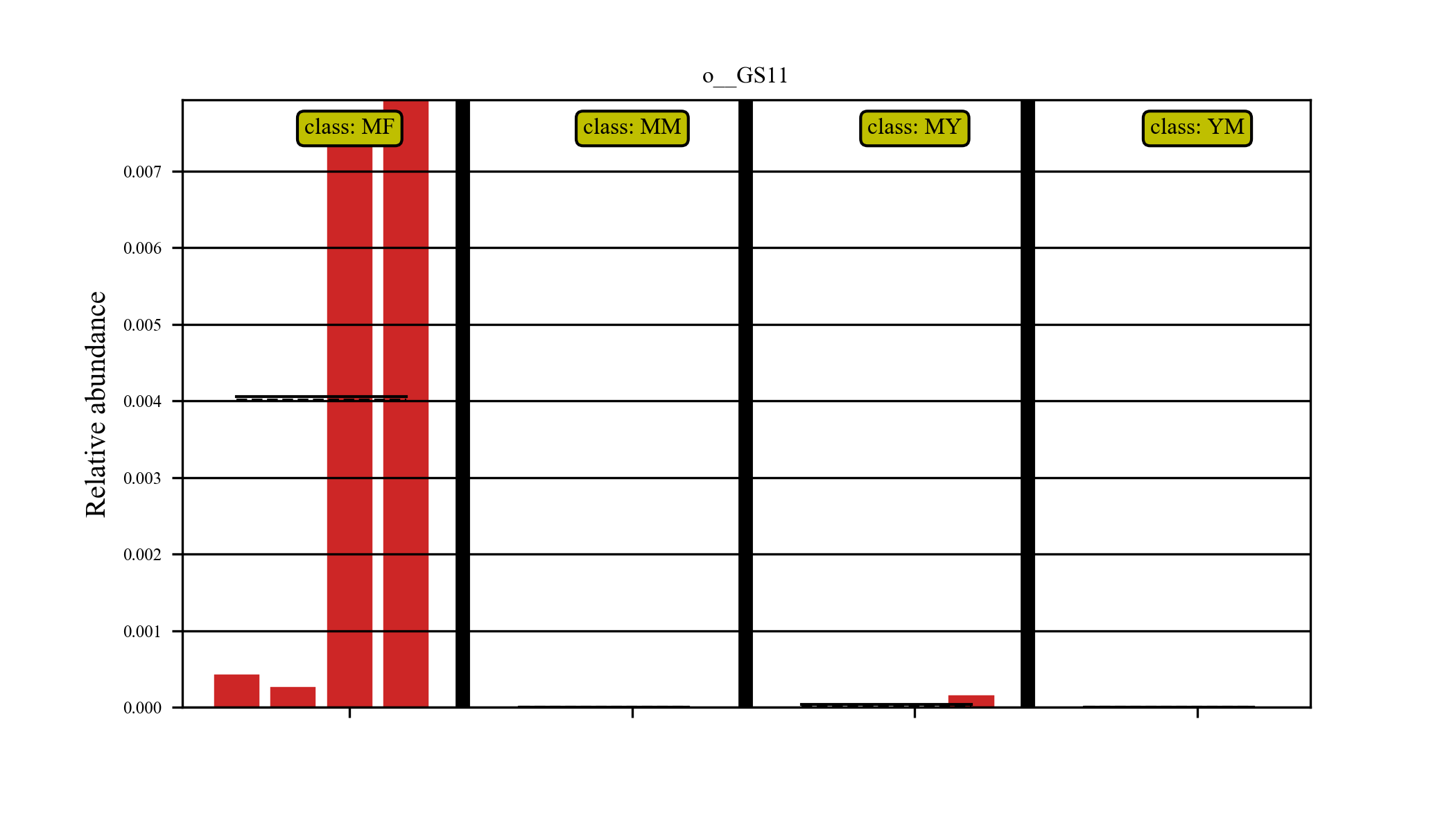

Supplement: Supplementary file 2 [file Data_Sheet_2.ZIP › Supplementary figure 2. fungal biomarker community/1_o__GS11.png]

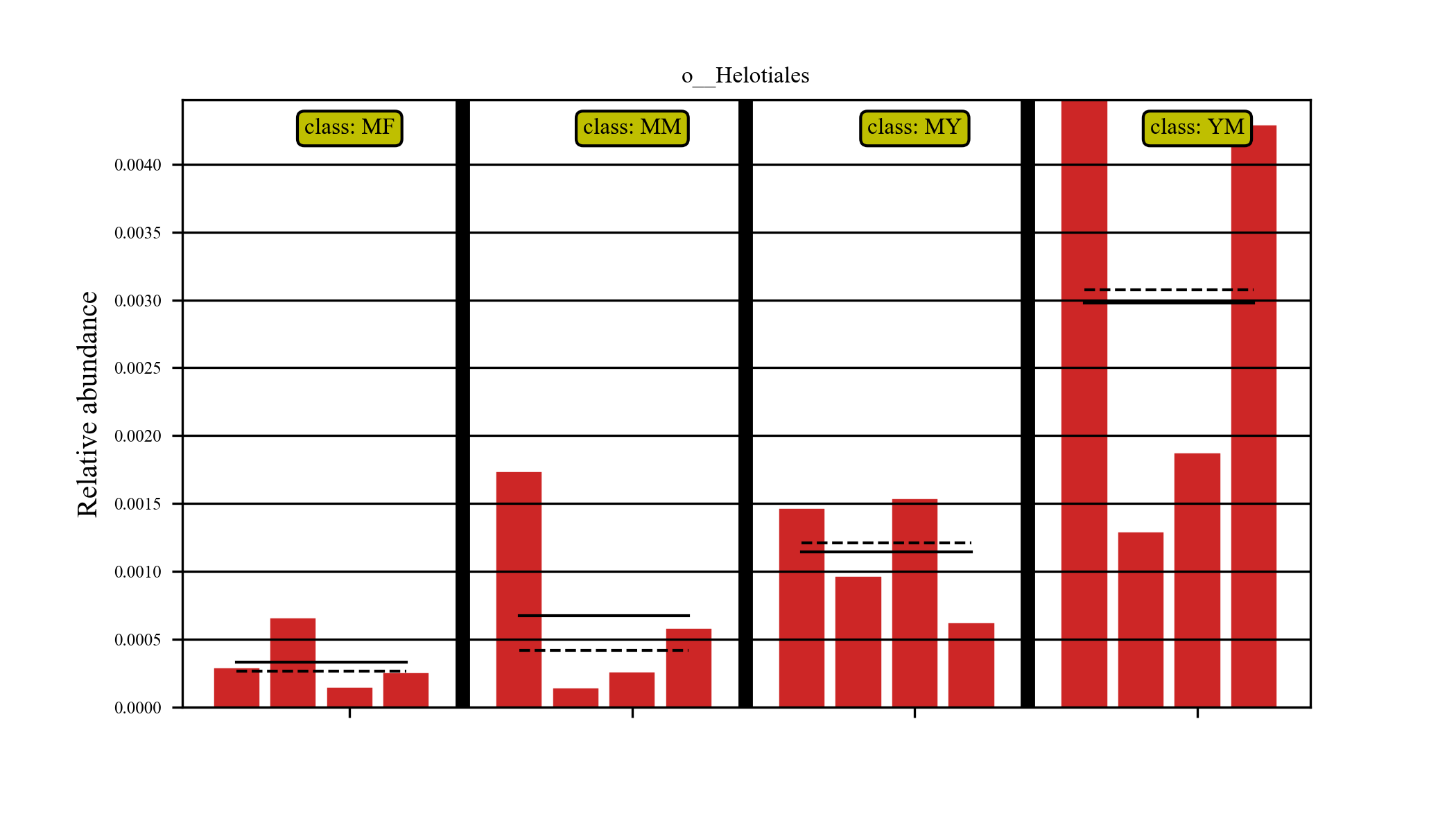

Supplement: Supplementary file 2 [file Data_Sheet_2.ZIP › Supplementary figure 2. fungal biomarker community/1_o__Helotiales.png]

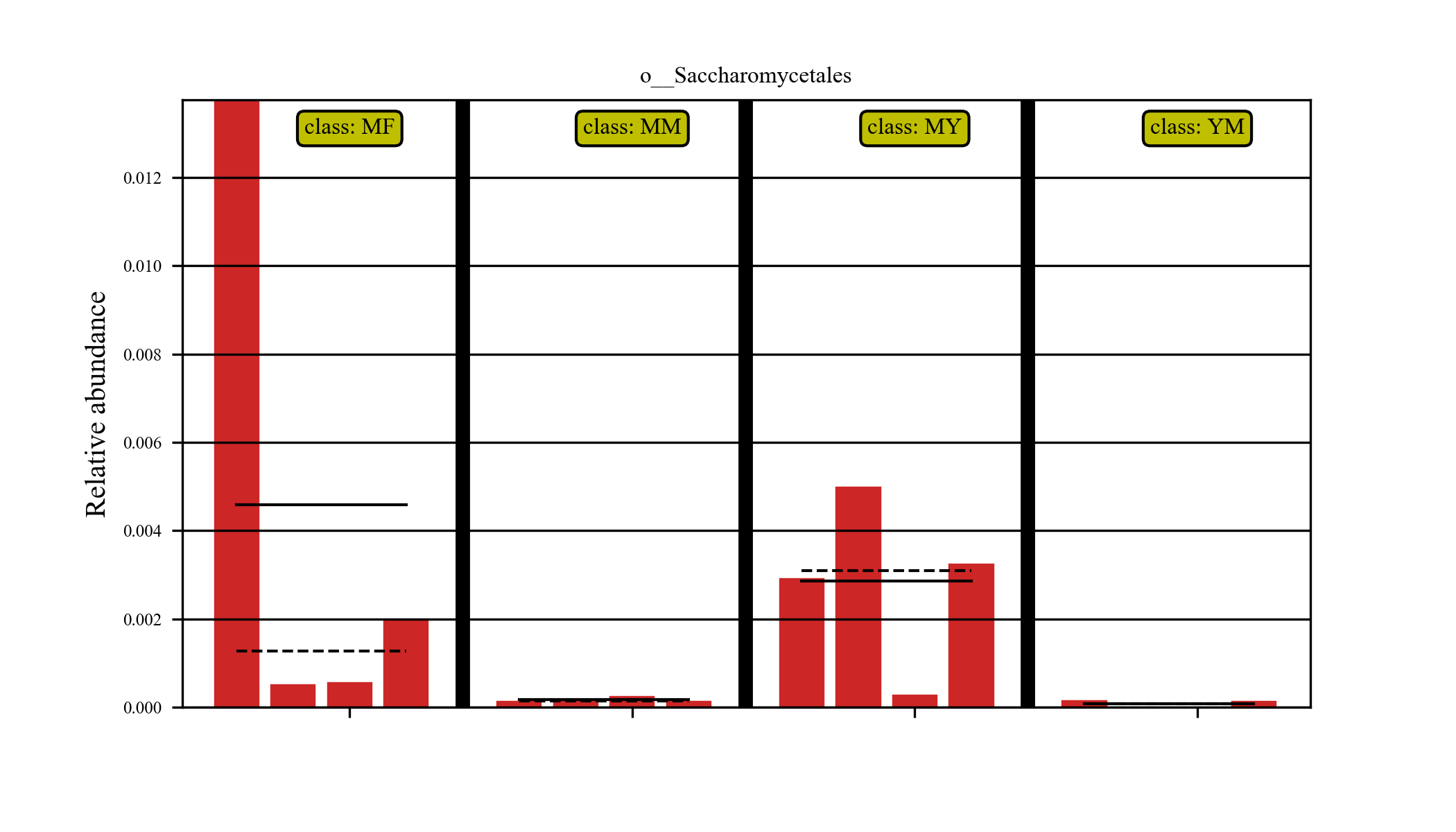

Supplement: Supplementary file 2 [file Data_Sheet_2.ZIP › Supplementary figure 2. fungal biomarker community/1_o__Saccharomycetales.png]

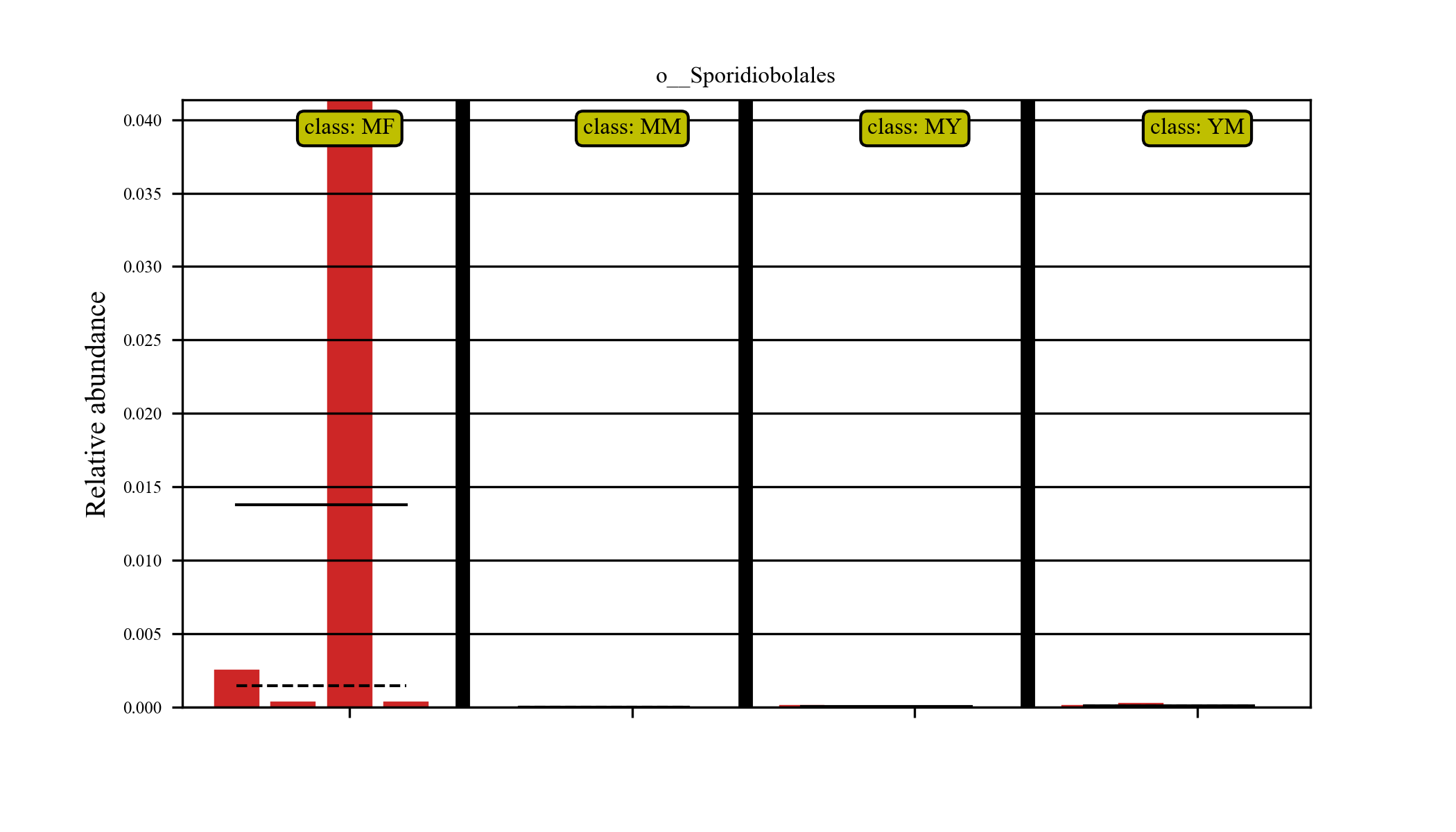

Supplement: Supplementary file 2 [file Data_Sheet_2.ZIP › Supplementary figure 2. fungal biomarker community/1_o__Sporidiobolales.png]

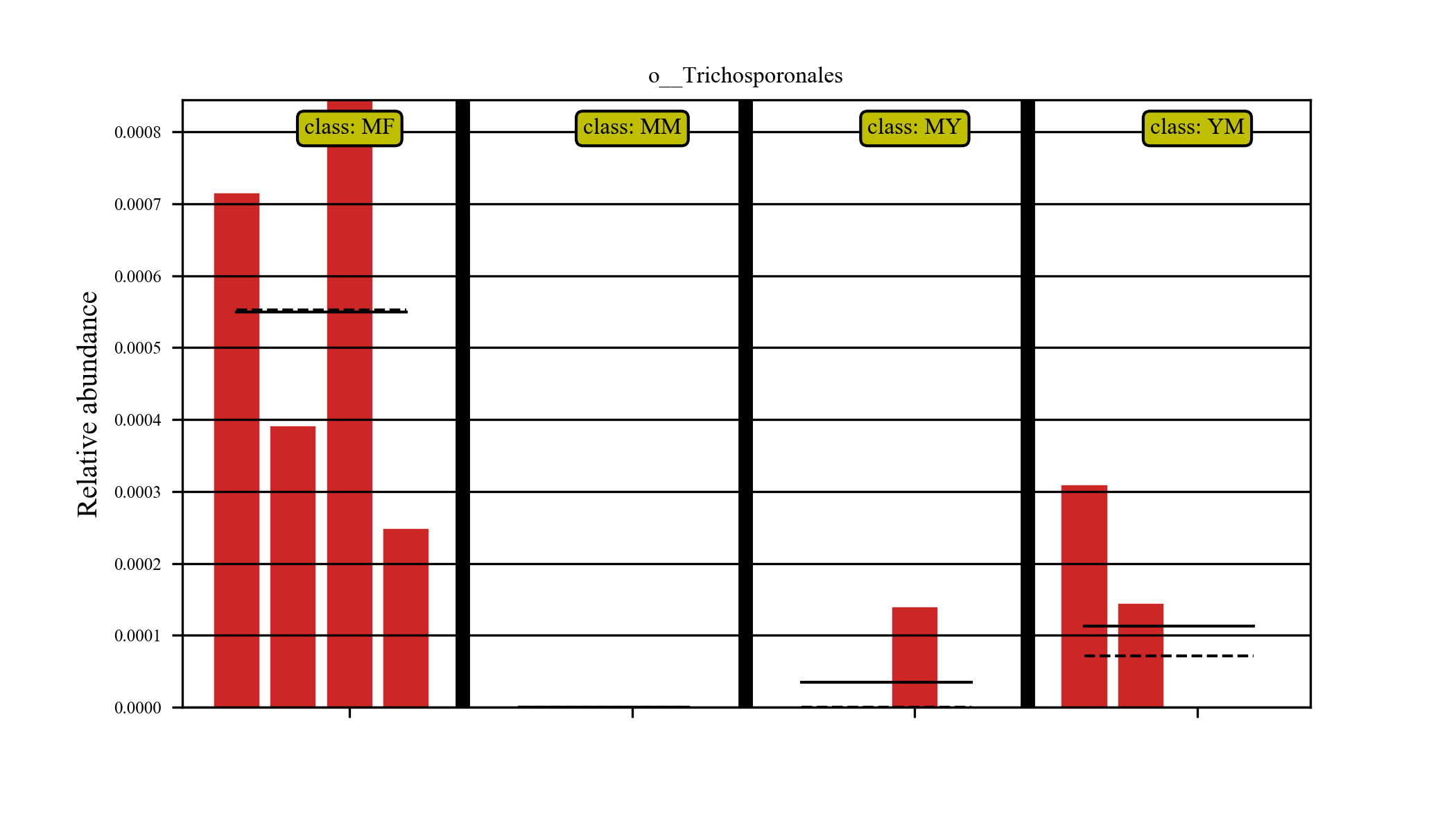

Supplement: Supplementary file 2 [file Data_Sheet_2.ZIP › Supplementary figure 2. fungal biomarker community/1_o__Trichosporonales.png]

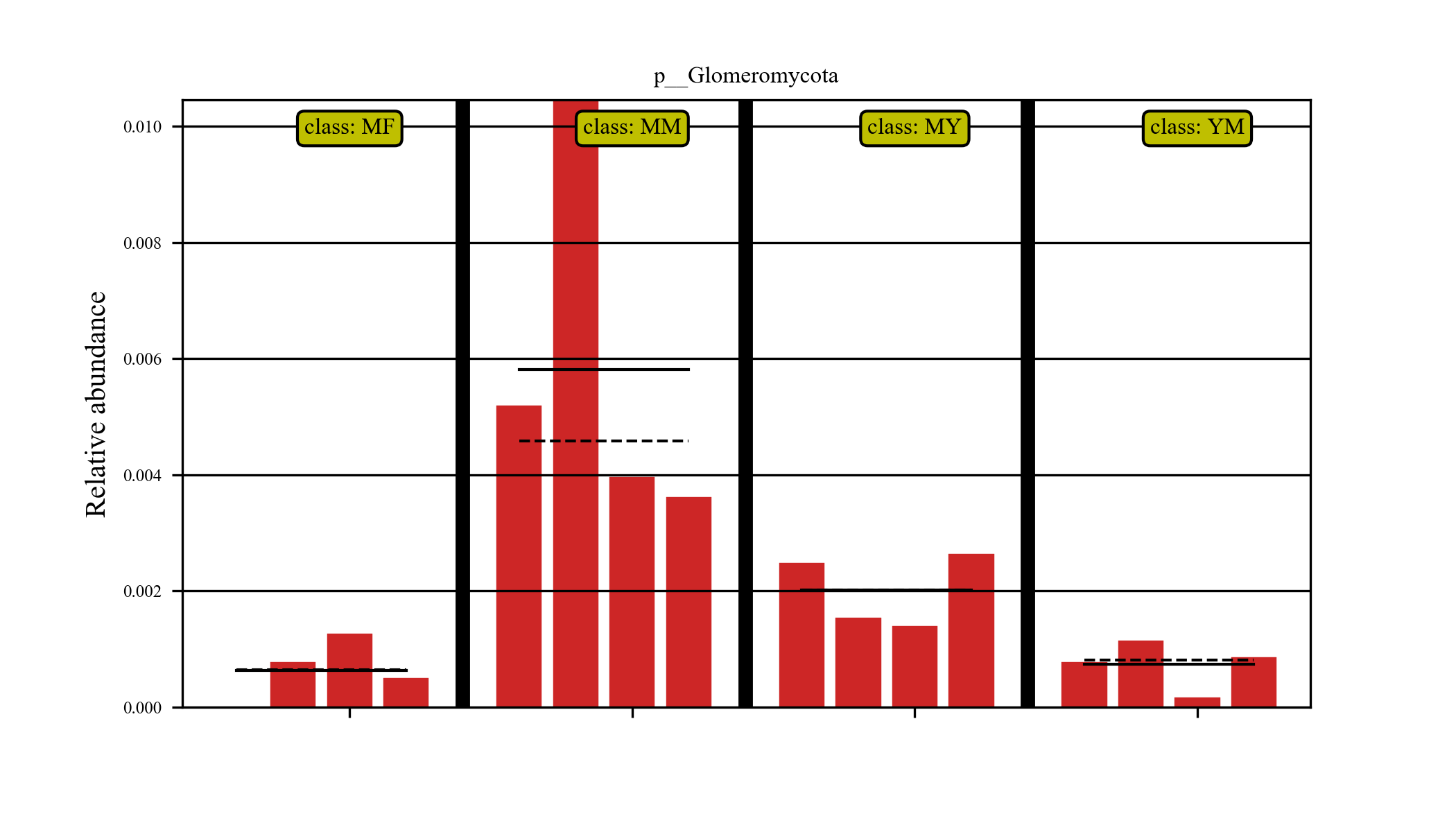

Supplement: Supplementary file 2 [file Data_Sheet_2.ZIP › Supplementary figure 2. fungal biomarker community/1_p__Glomeromycota.png]

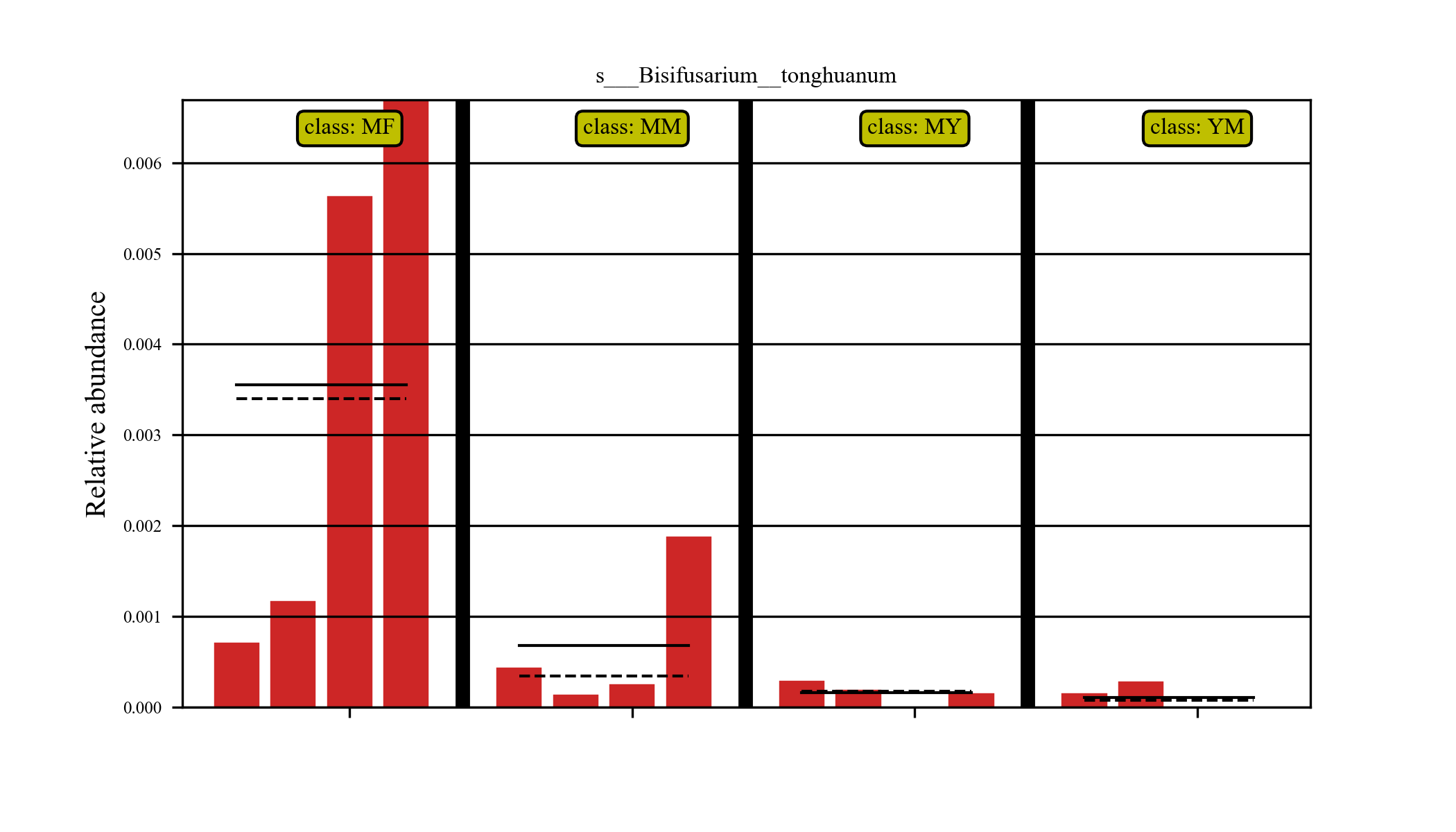

Supplement: Supplementary file 2 [file Data_Sheet_2.ZIP › Supplementary figure 2. fungal biomarker community/1_s___Bisifusarium__tonghuanum.png]

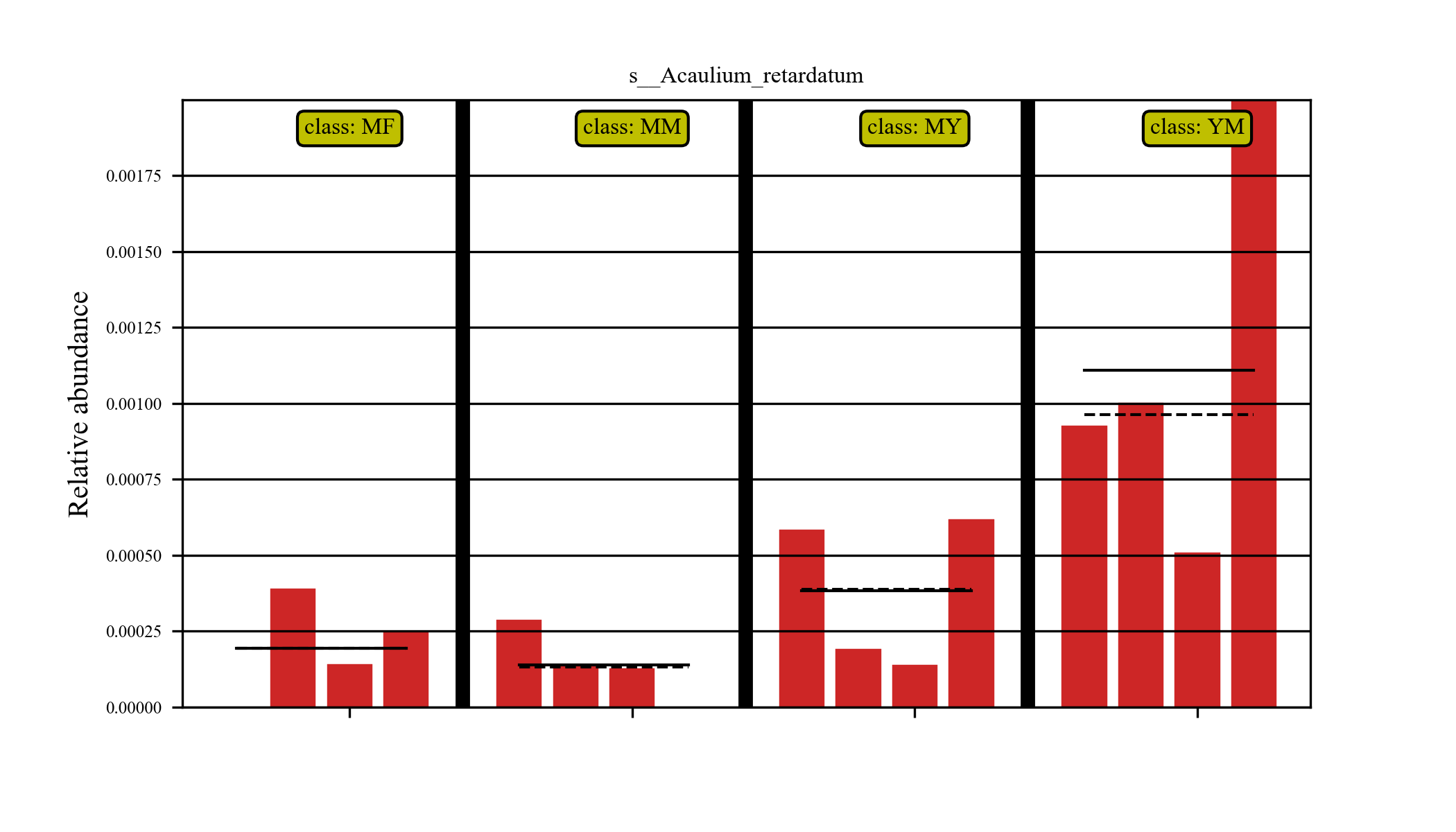

Supplement: Supplementary file 2 [file Data_Sheet_2.ZIP › Supplementary figure 2. fungal biomarker community/1_s__Acaulium_retardatum.png]

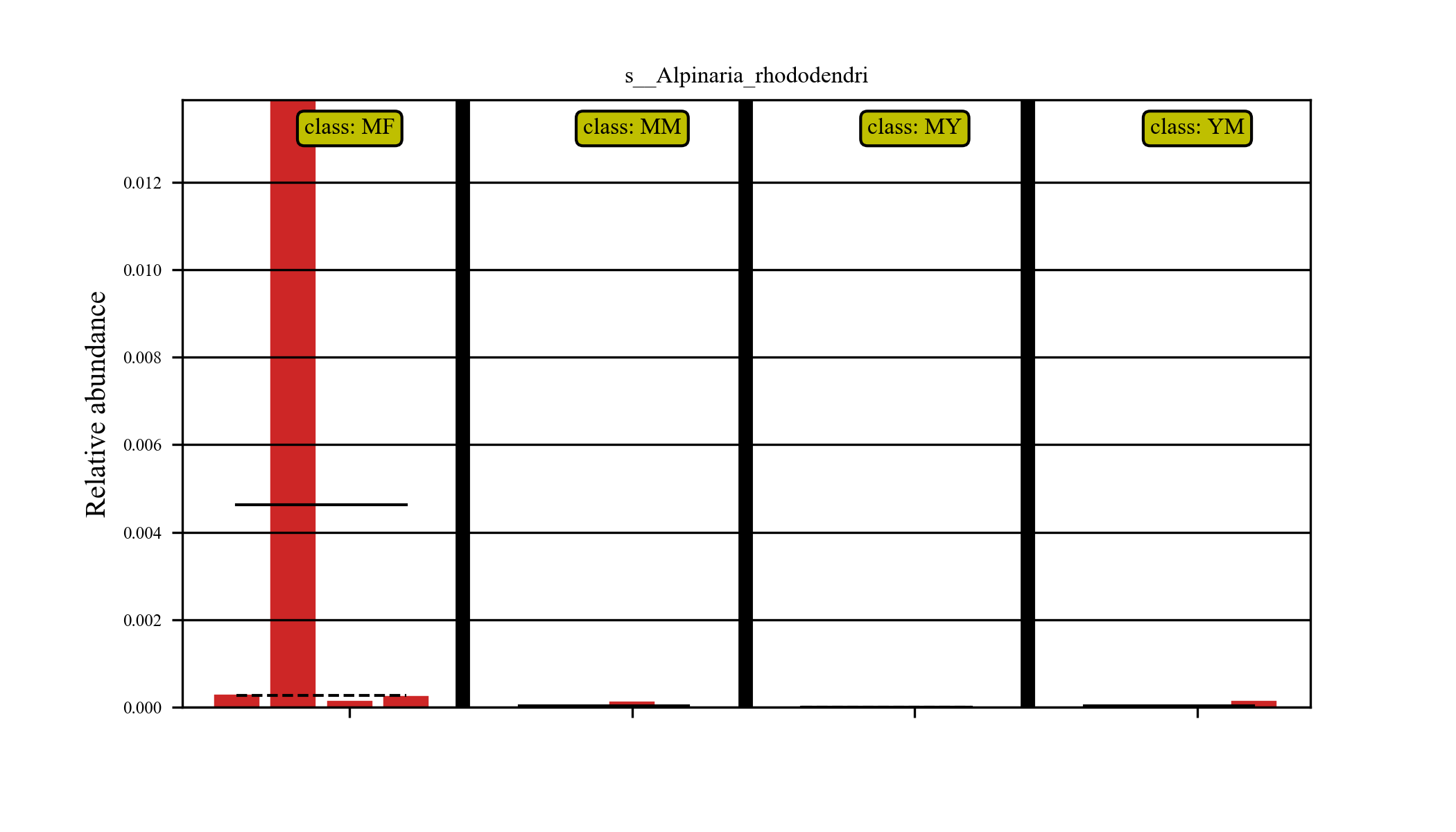

Supplement: Supplementary file 2 [file Data_Sheet_2.ZIP › Supplementary figure 2. fungal biomarker community/1_s__Alpinaria_rhododendri.png]

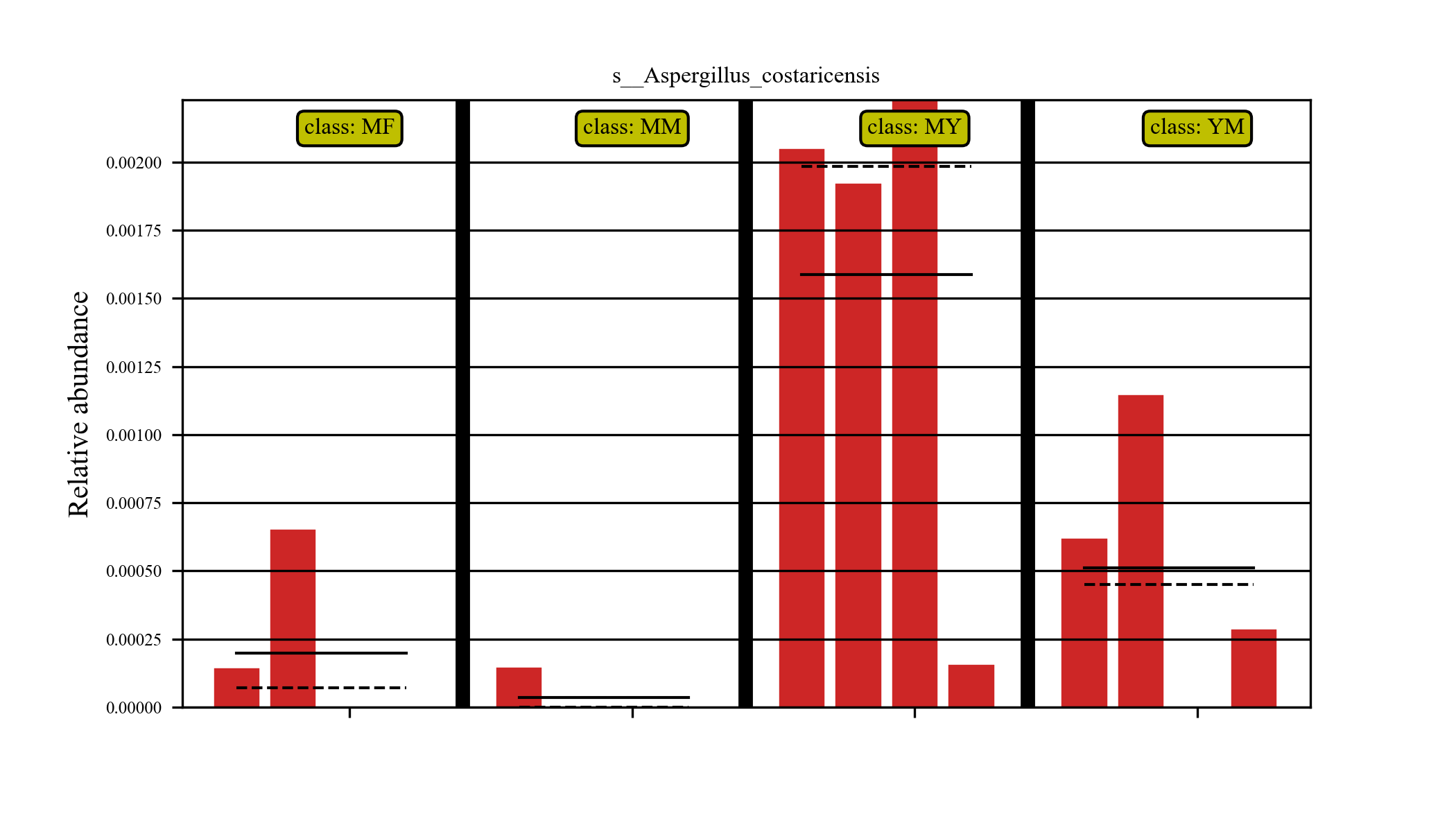

Supplement: Supplementary file 2 [file Data_Sheet_2.ZIP › Supplementary figure 2. fungal biomarker community/1_s__Aspergillus_costaricensis.png]

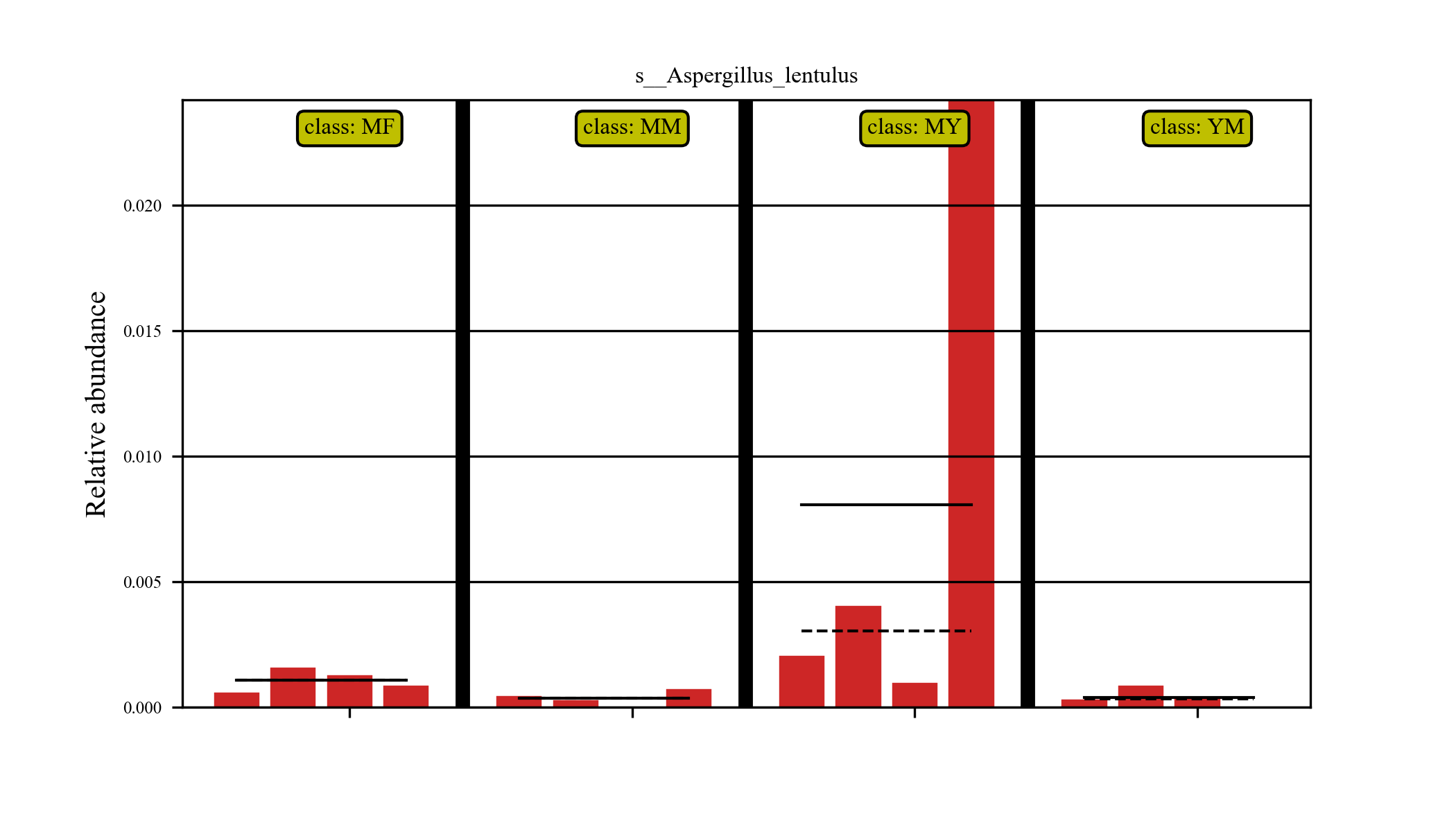

Supplement: Supplementary file 2 [file Data_Sheet_2.ZIP › Supplementary figure 2. fungal biomarker community/1_s__Aspergillus_lentulus.png]

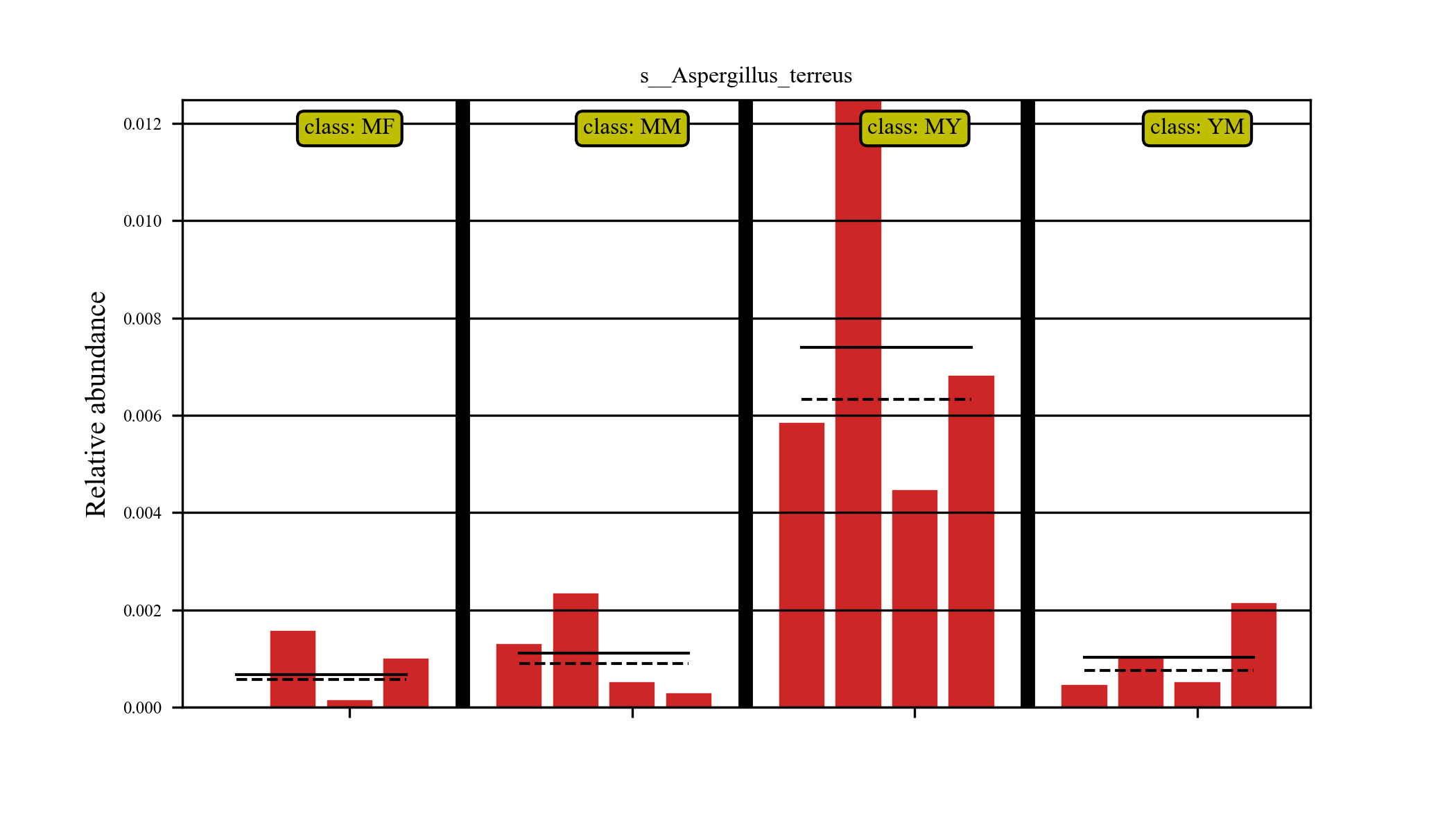

Supplement: Supplementary file 2 [file Data_Sheet_2.ZIP › Supplementary figure 2. fungal biomarker community/1_s__Aspergillus_terreus.png]

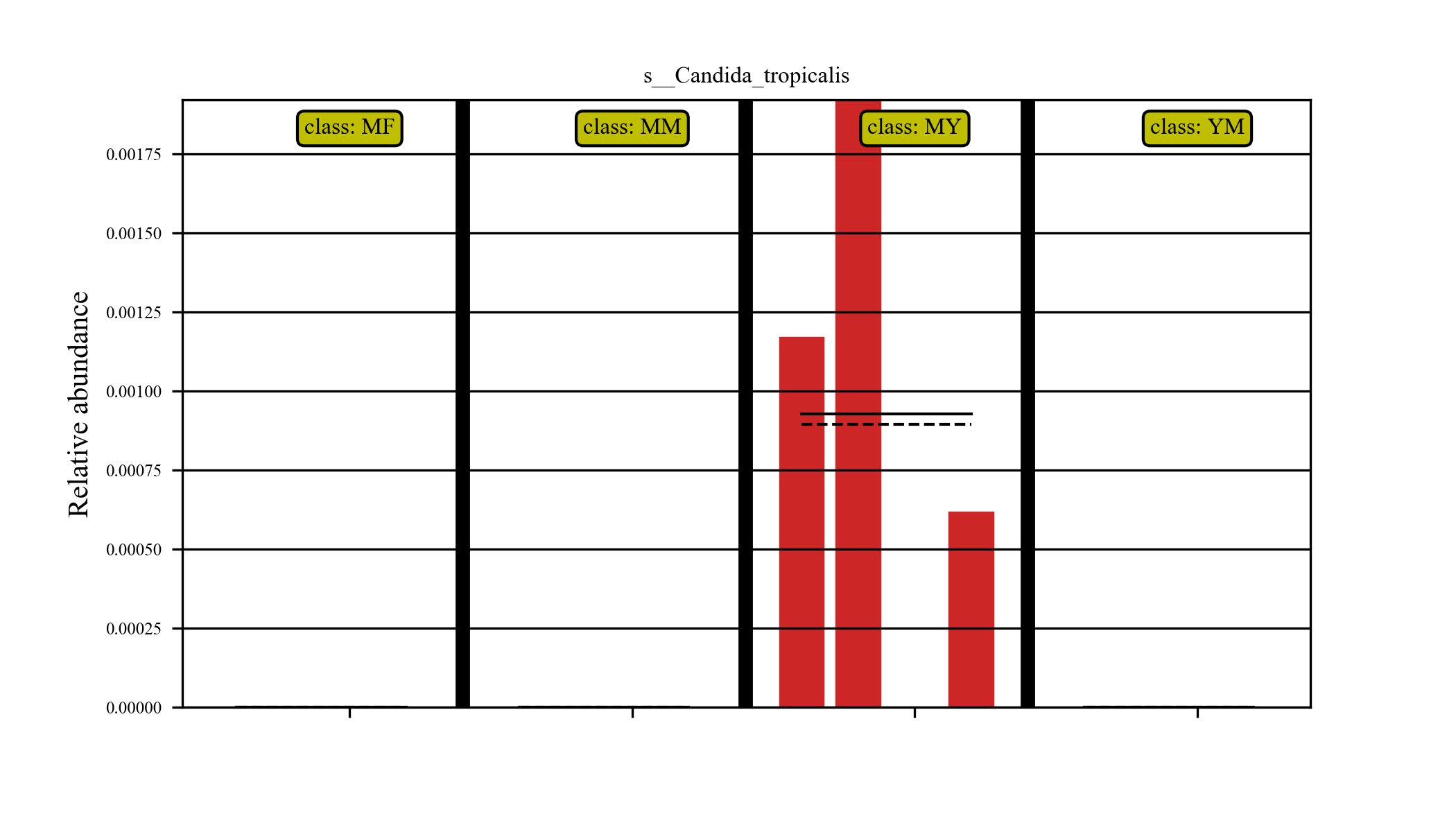

Supplement: Supplementary file 2 [file Data_Sheet_2.ZIP › Supplementary figure 2. fungal biomarker community/1_s__Candida_tropicalis.png]

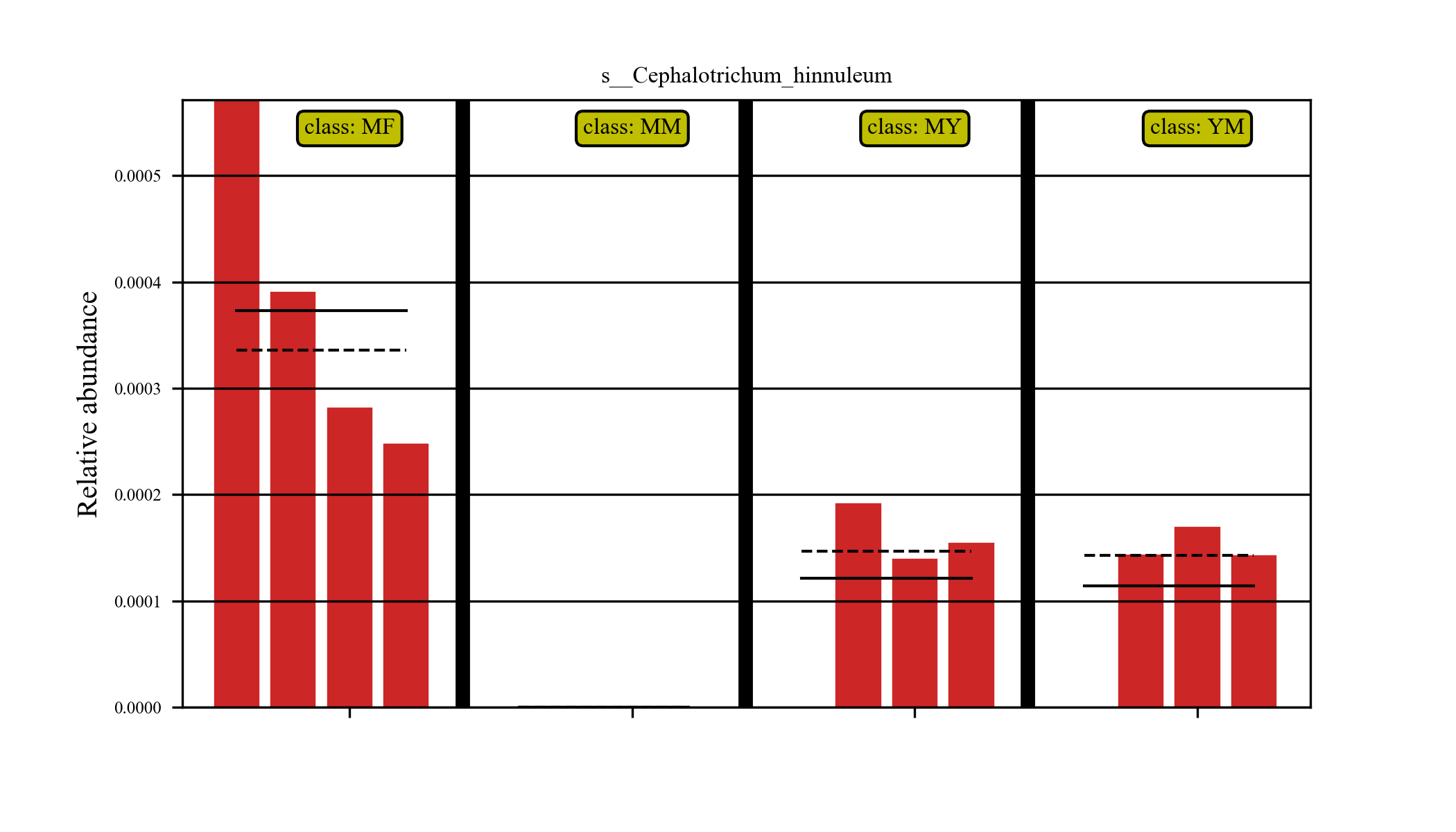

Supplement: Supplementary file 2 [file Data_Sheet_2.ZIP › Supplementary figure 2. fungal biomarker community/1_s__Cephalotrichum_hinnuleum.png]

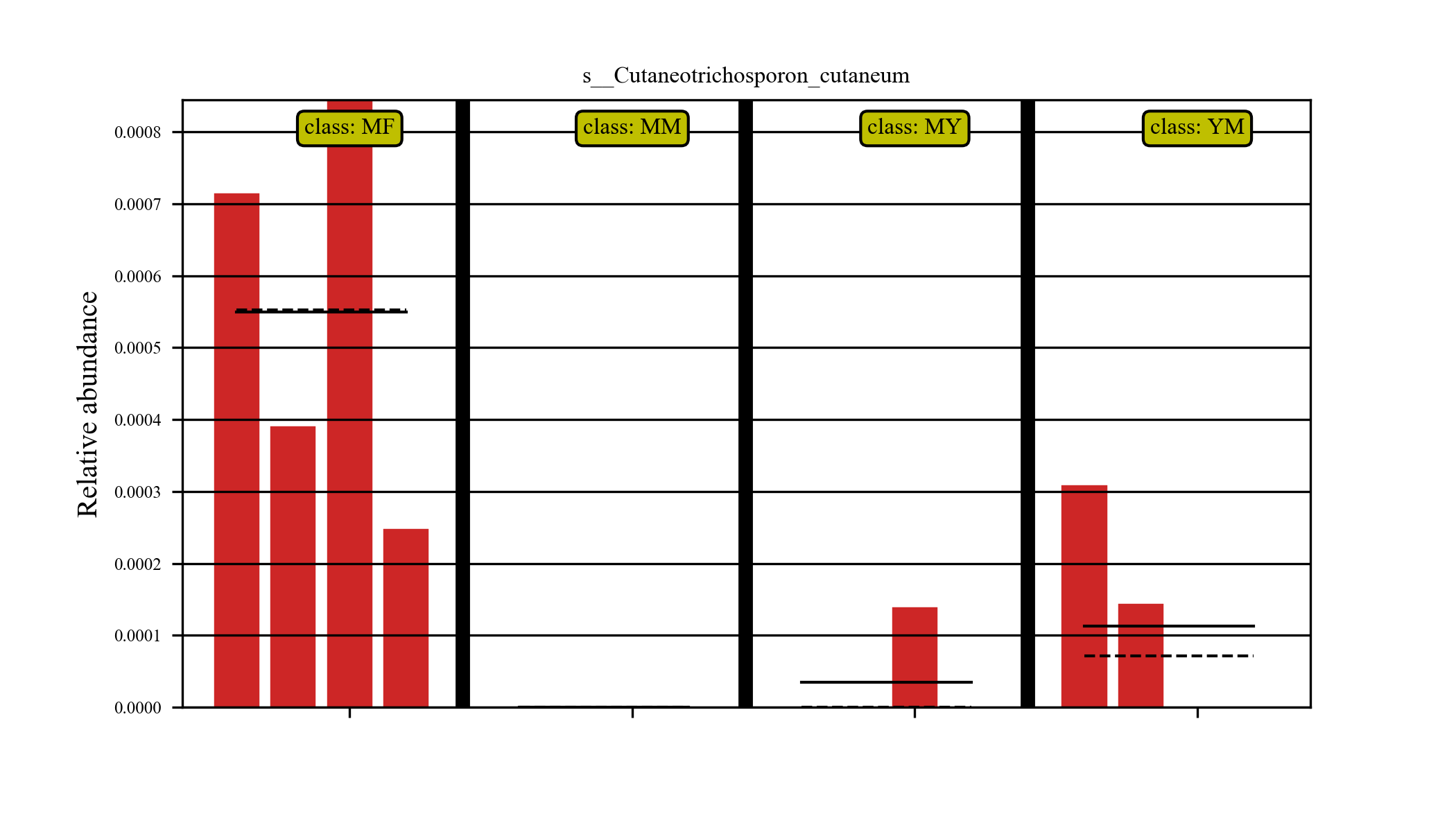

Supplement: Supplementary file 2 [file Data_Sheet_2.ZIP › Supplementary figure 2. fungal biomarker community/1_s__Cutaneotrichosporon_cutaneum.png]

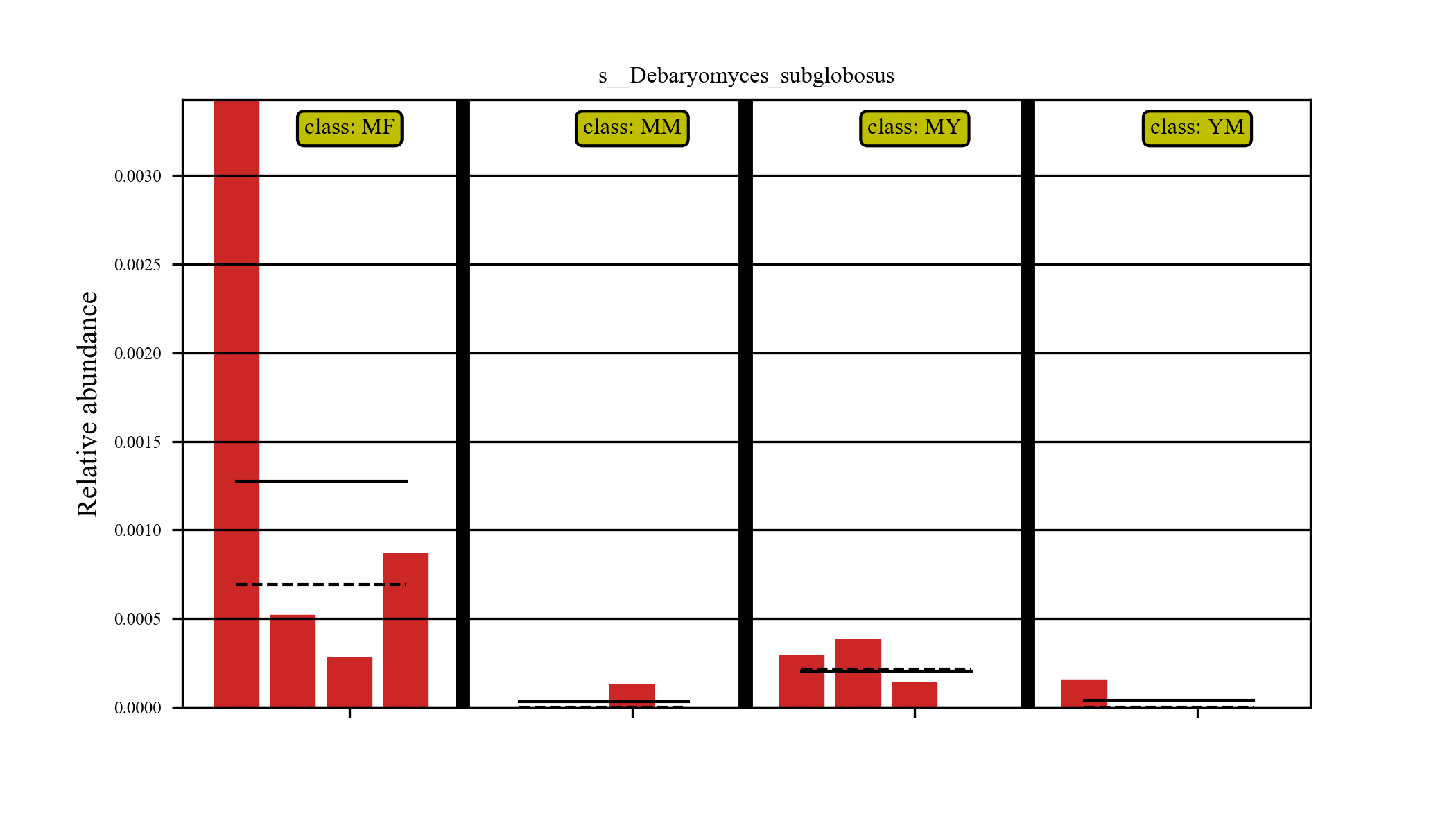

Supplement: Supplementary file 2 [file Data_Sheet_2.ZIP › Supplementary figure 2. fungal biomarker community/1_s__Debaryomyces_subglobosus.png]

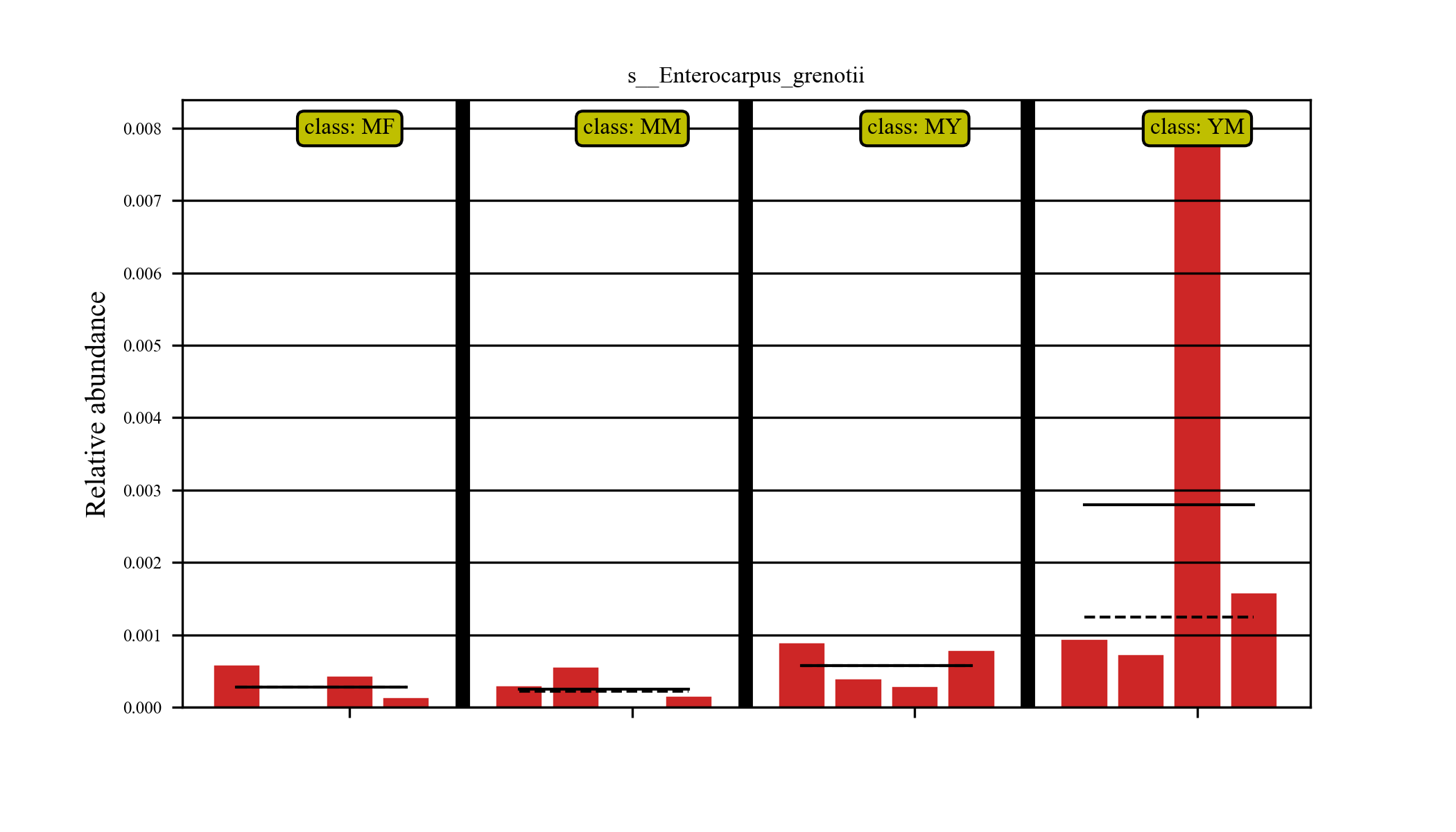

Supplement: Supplementary file 2 [file Data_Sheet_2.ZIP › Supplementary figure 2. fungal biomarker community/1_s__Enterocarpus_grenotii.png]

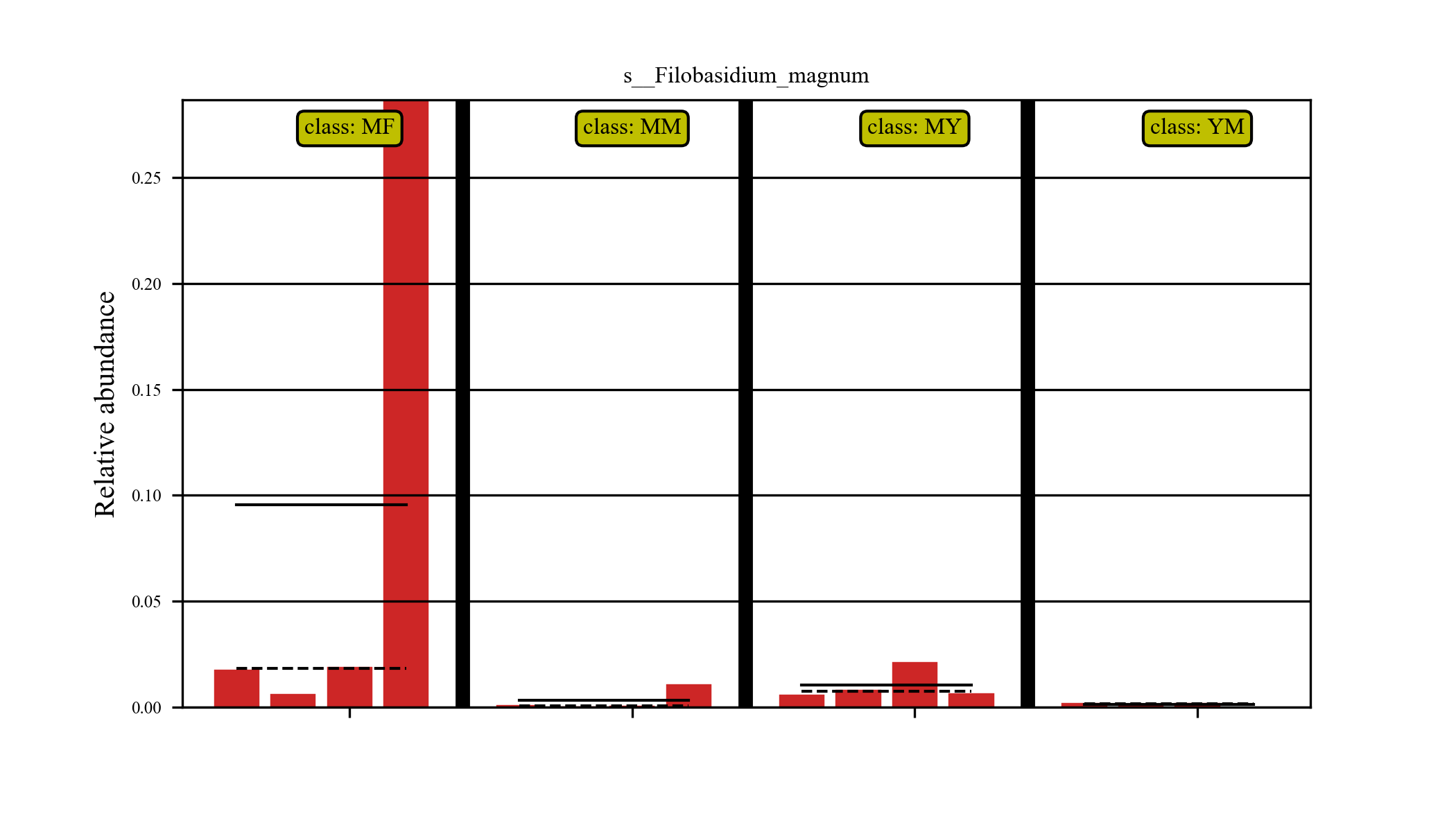

Supplement: Supplementary file 2 [file Data_Sheet_2.ZIP › Supplementary figure 2. fungal biomarker community/1_s__Filobasidium_magnum.png]

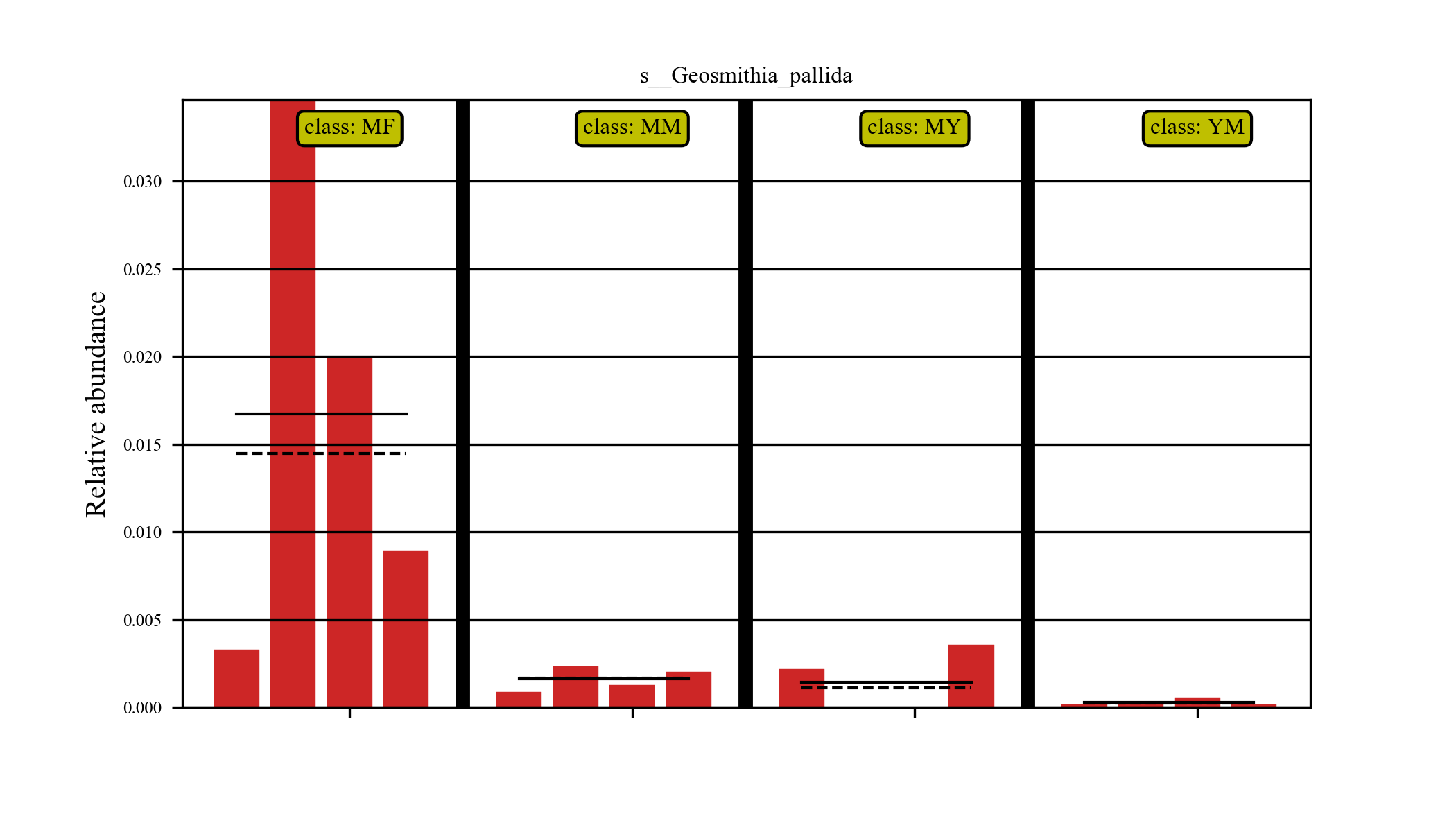

Supplement: Supplementary file 2 [file Data_Sheet_2.ZIP › Supplementary figure 2. fungal biomarker community/1_s__Geosmithia_pallida.png]

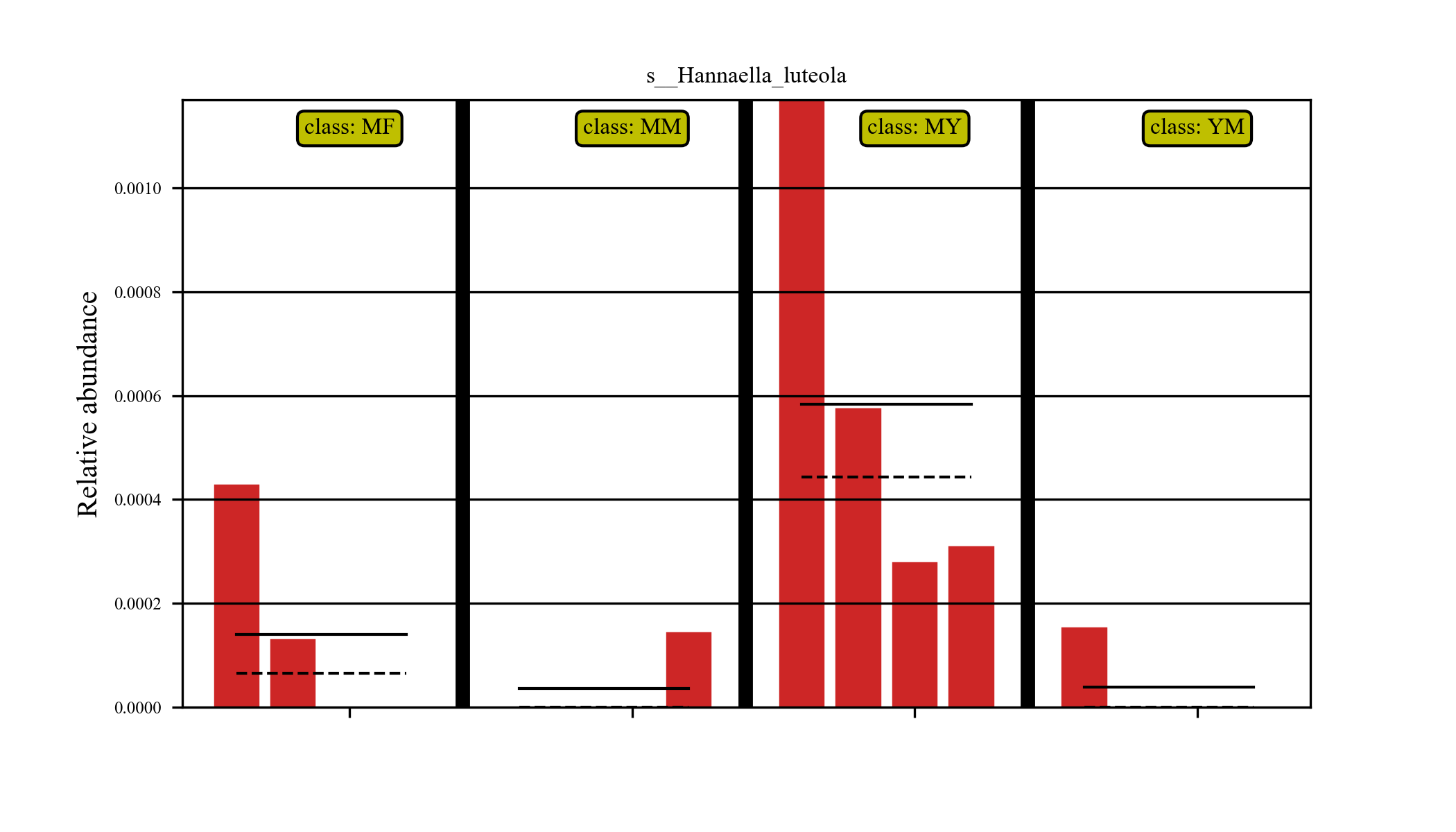

Supplement: Supplementary file 2 [file Data_Sheet_2.ZIP › Supplementary figure 2. fungal biomarker community/1_s__Hannaella_luteola.png]

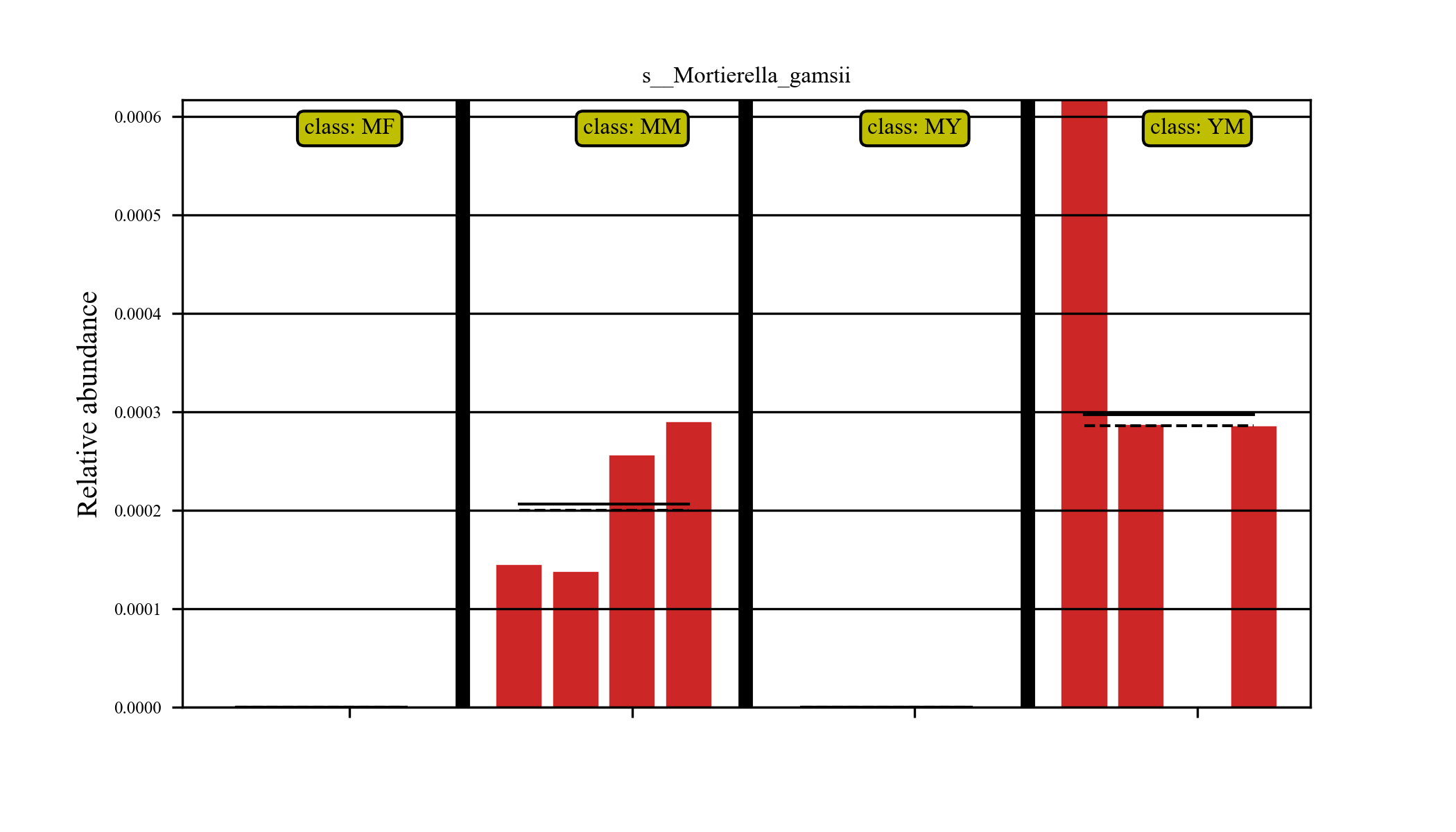

Supplement: Supplementary file 2 [file Data_Sheet_2.ZIP › Supplementary figure 2. fungal biomarker community/1_s__Mortierella_gamsii.png]

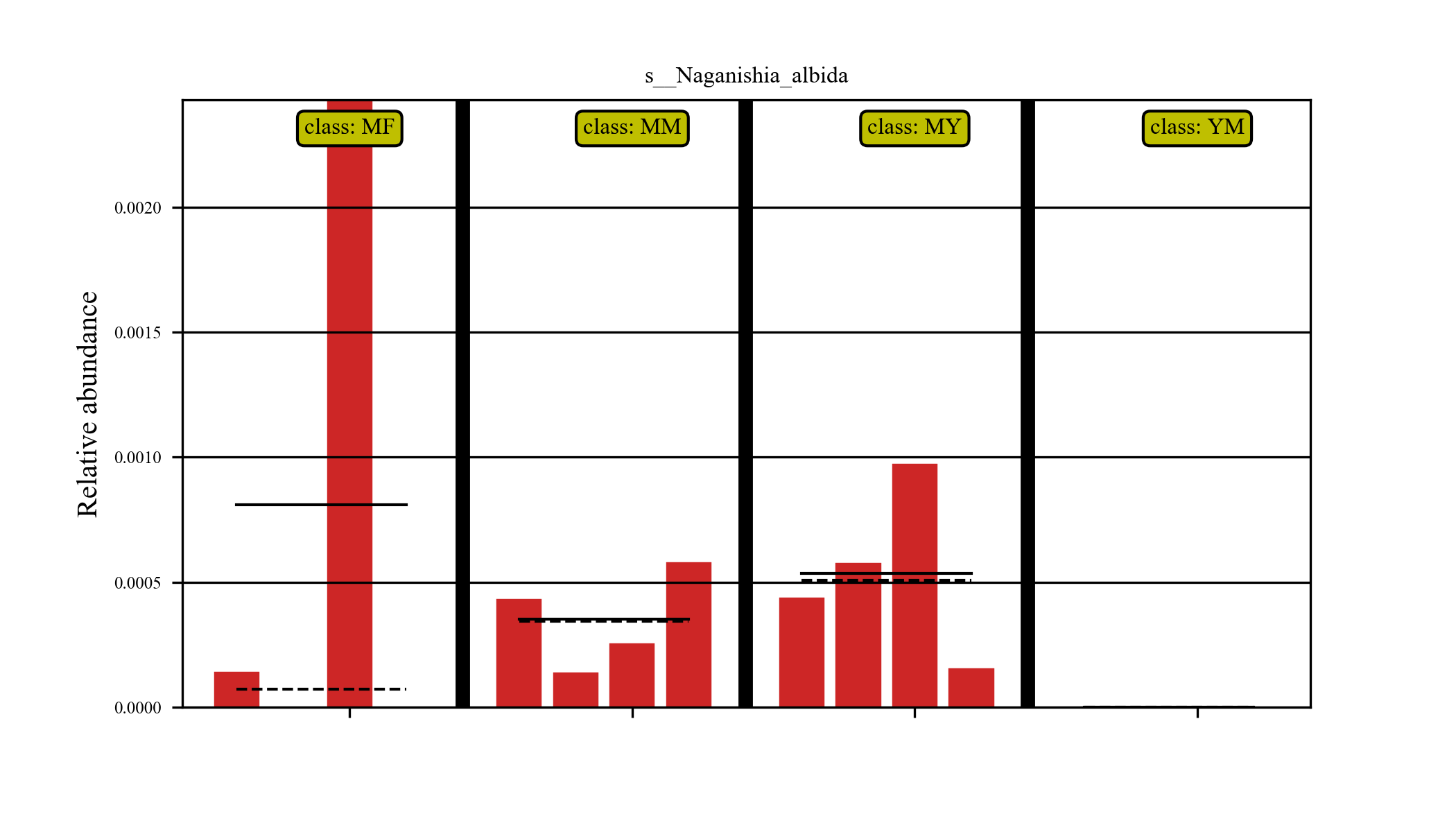

Supplement: Supplementary file 2 [file Data_Sheet_2.ZIP › Supplementary figure 2. fungal biomarker community/1_s__Naganishia_albida.png]

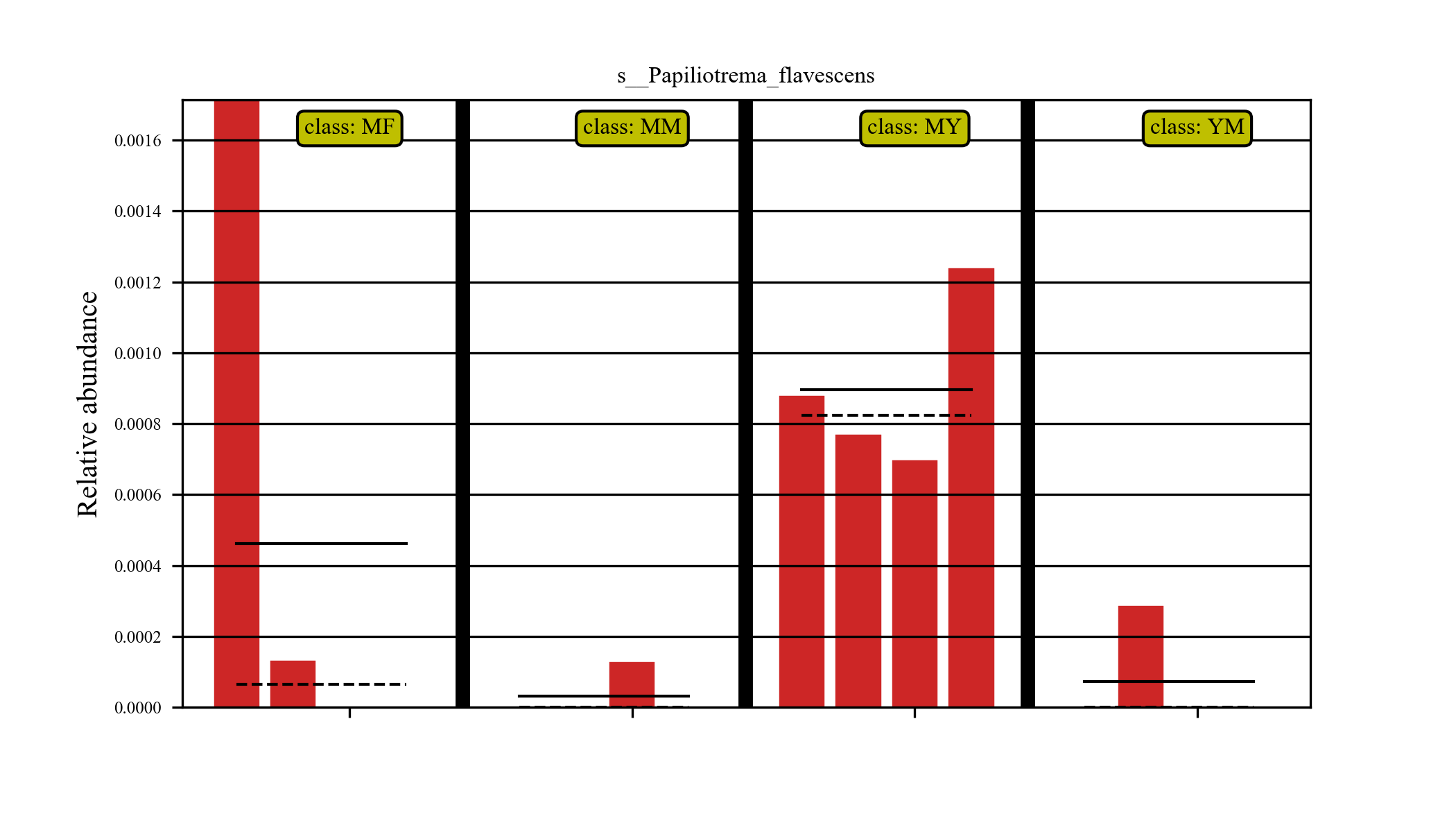

Supplement: Supplementary file 2 [file Data_Sheet_2.ZIP › Supplementary figure 2. fungal biomarker community/1_s__Papiliotrema_flavescens.png]

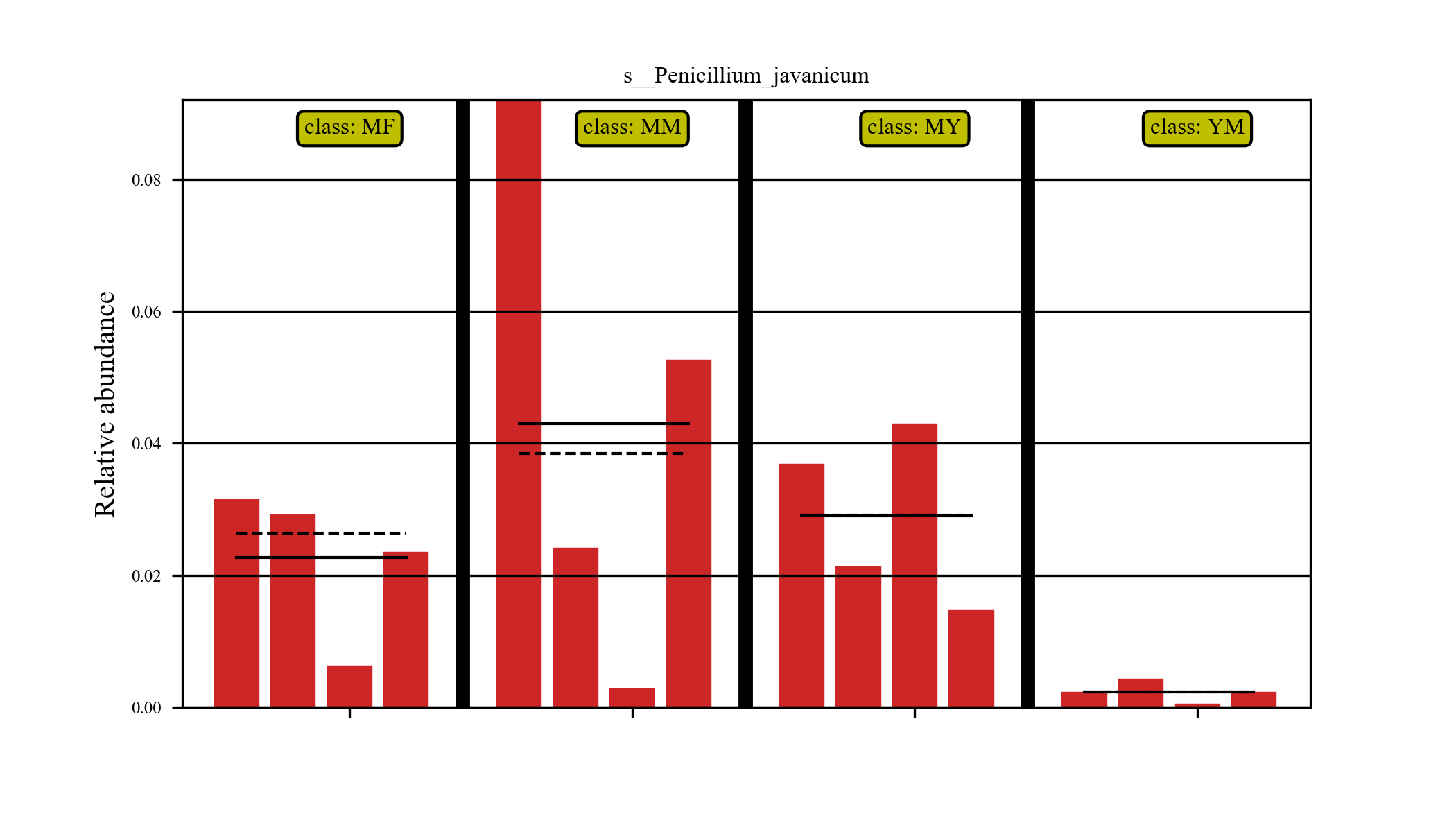

Supplement: Supplementary file 2 [file Data_Sheet_2.ZIP › Supplementary figure 2. fungal biomarker community/1_s__Penicillium_javanicum.png]

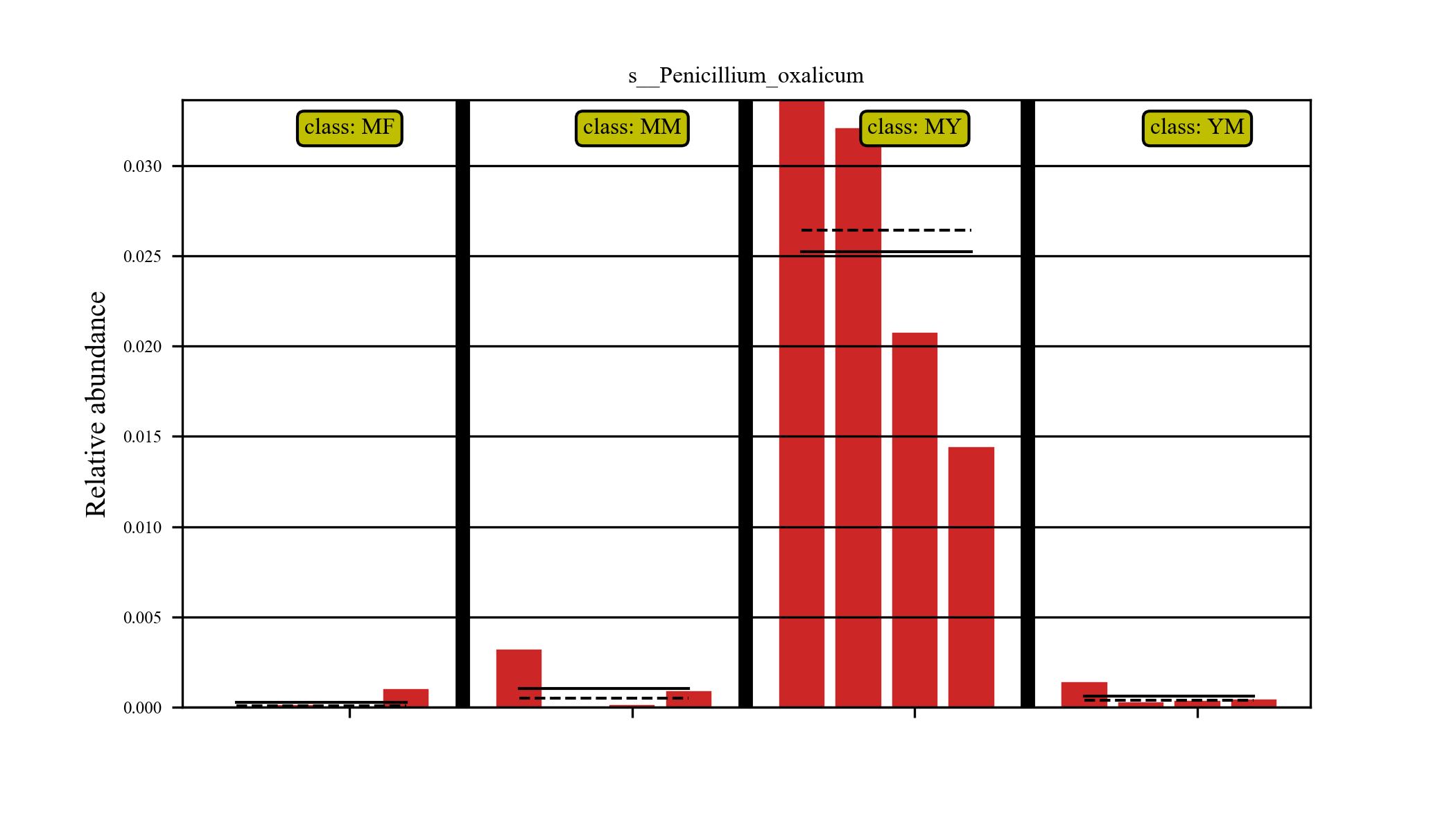

Supplement: Supplementary file 2 [file Data_Sheet_2.ZIP › Supplementary figure 2. fungal biomarker community/1_s__Penicillium_oxalicum.png]

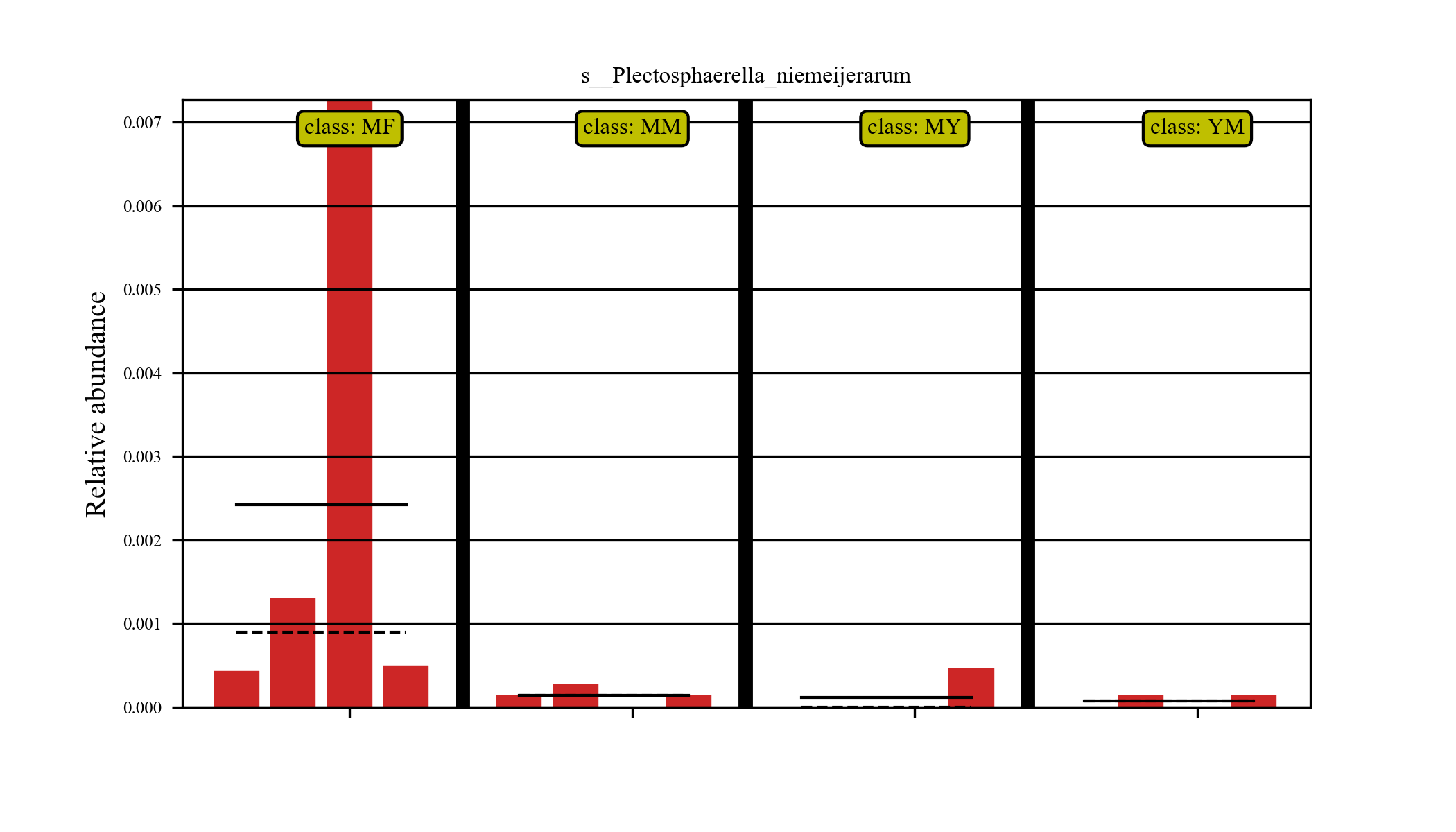

Supplement: Supplementary file 2 [file Data_Sheet_2.ZIP › Supplementary figure 2. fungal biomarker community/1_s__Plectosphaerella_niemeijerarum.png]

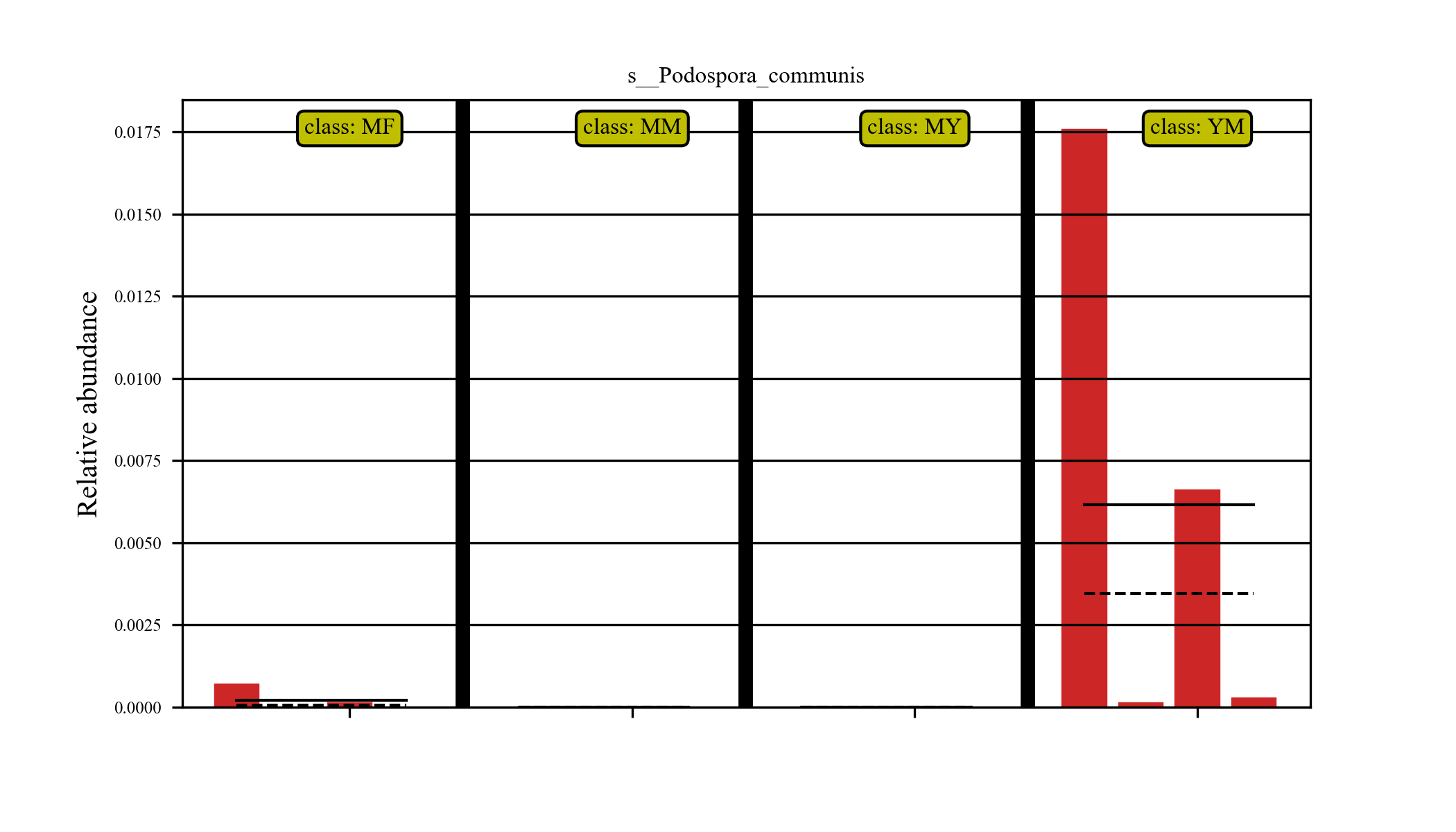

Supplement: Supplementary file 2 [file Data_Sheet_2.ZIP › Supplementary figure 2. fungal biomarker community/1_s__Podospora_communis.png]

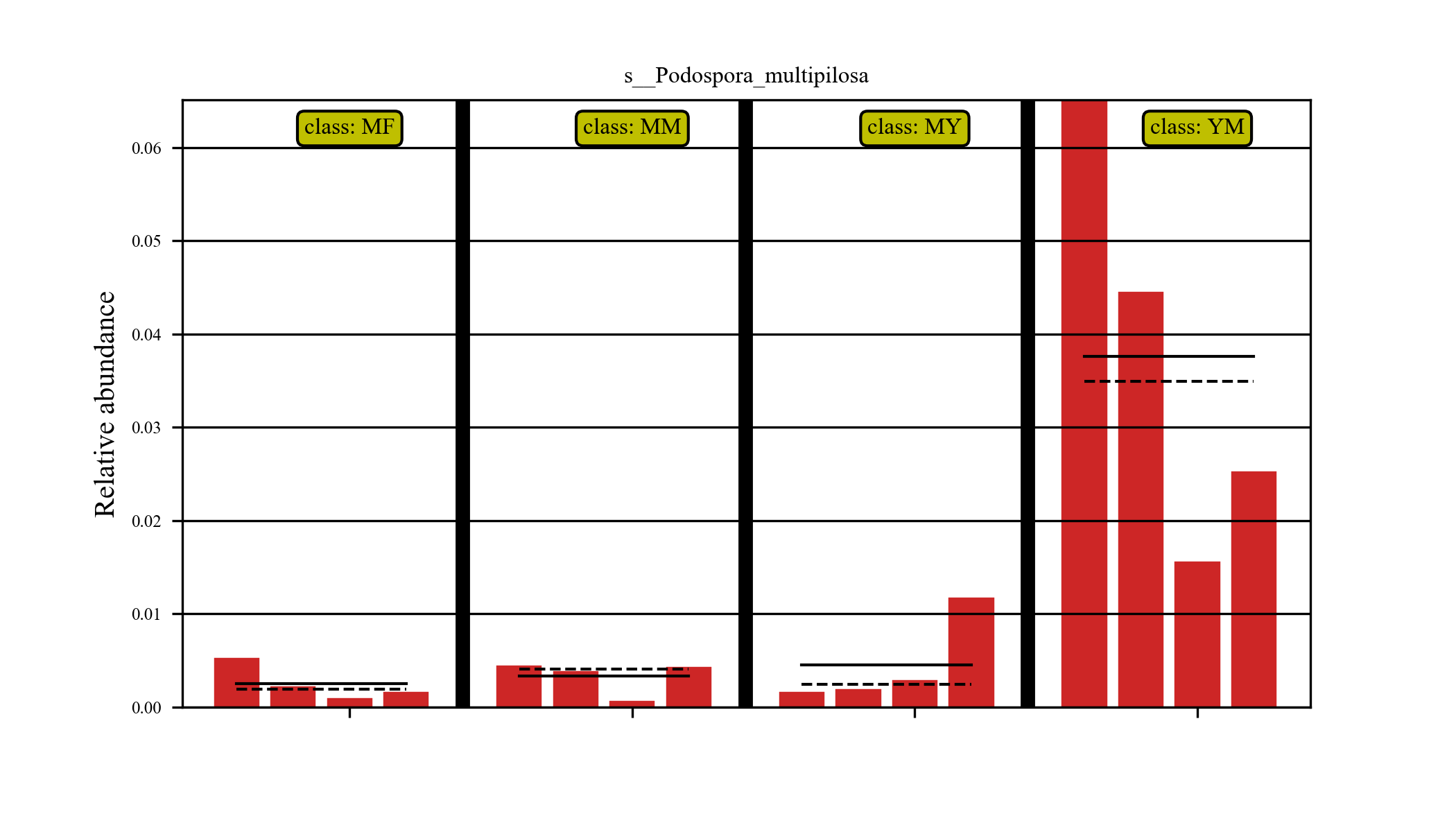

Supplement: Supplementary file 2 [file Data_Sheet_2.ZIP › Supplementary figure 2. fungal biomarker community/1_s__Podospora_multipilosa.png]

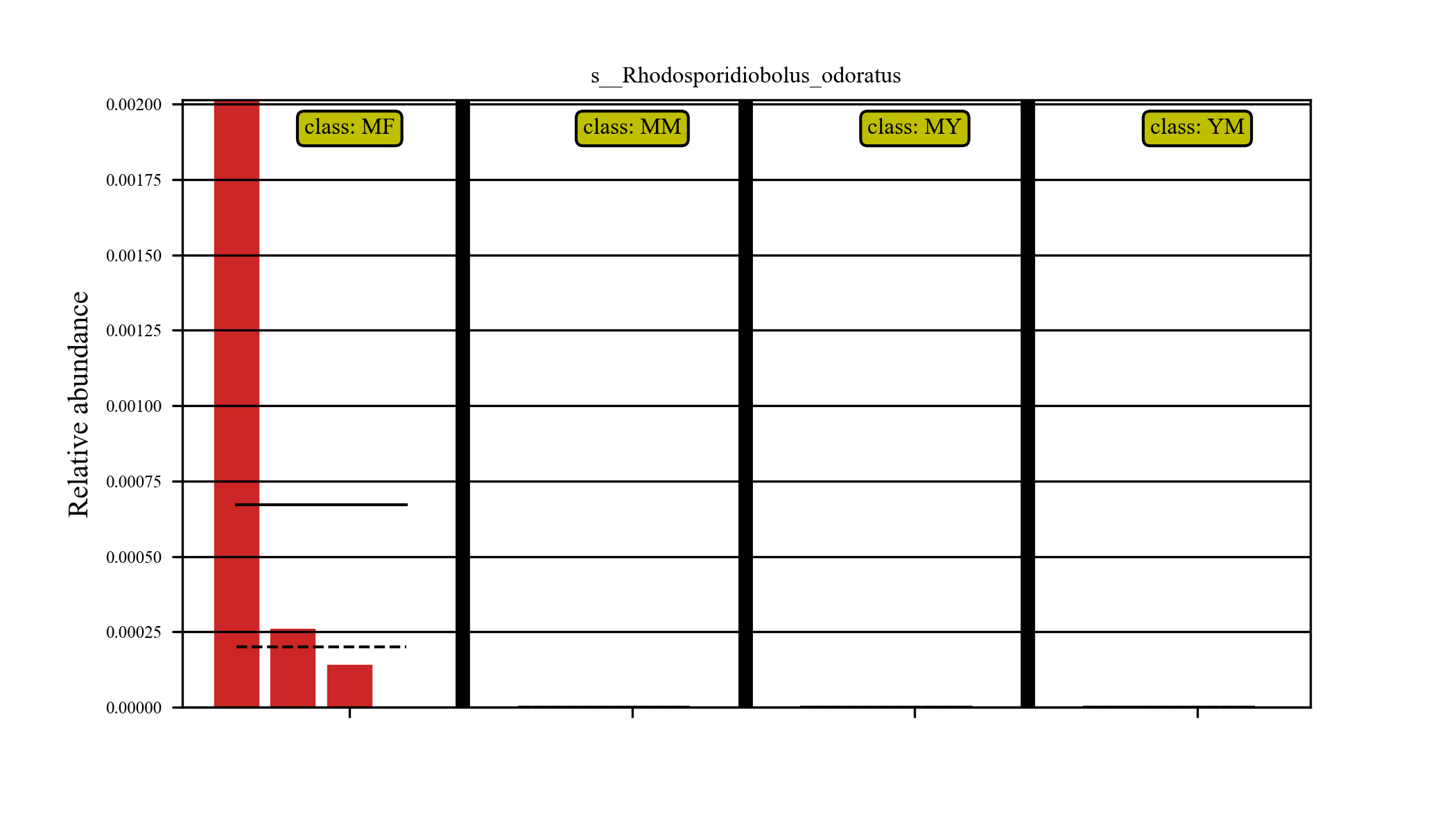

Supplement: Supplementary file 2 [file Data_Sheet_2.ZIP › Supplementary figure 2. fungal biomarker community/1_s__Rhodosporidiobolus_odoratus.png]

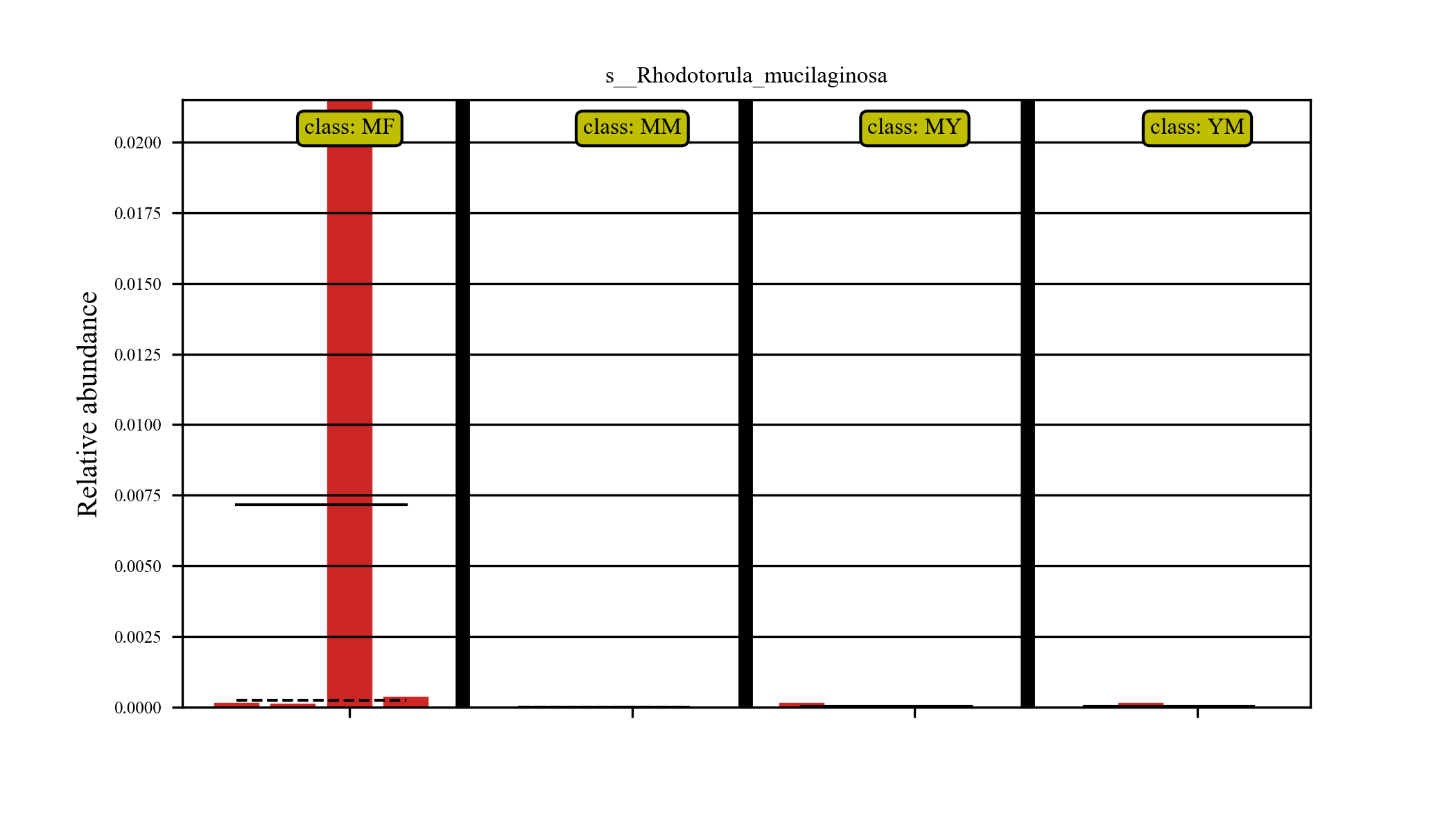

Supplement: Supplementary file 2 [file Data_Sheet_2.ZIP › Supplementary figure 2. fungal biomarker community/1_s__Rhodotorula_mucilaginosa.png]

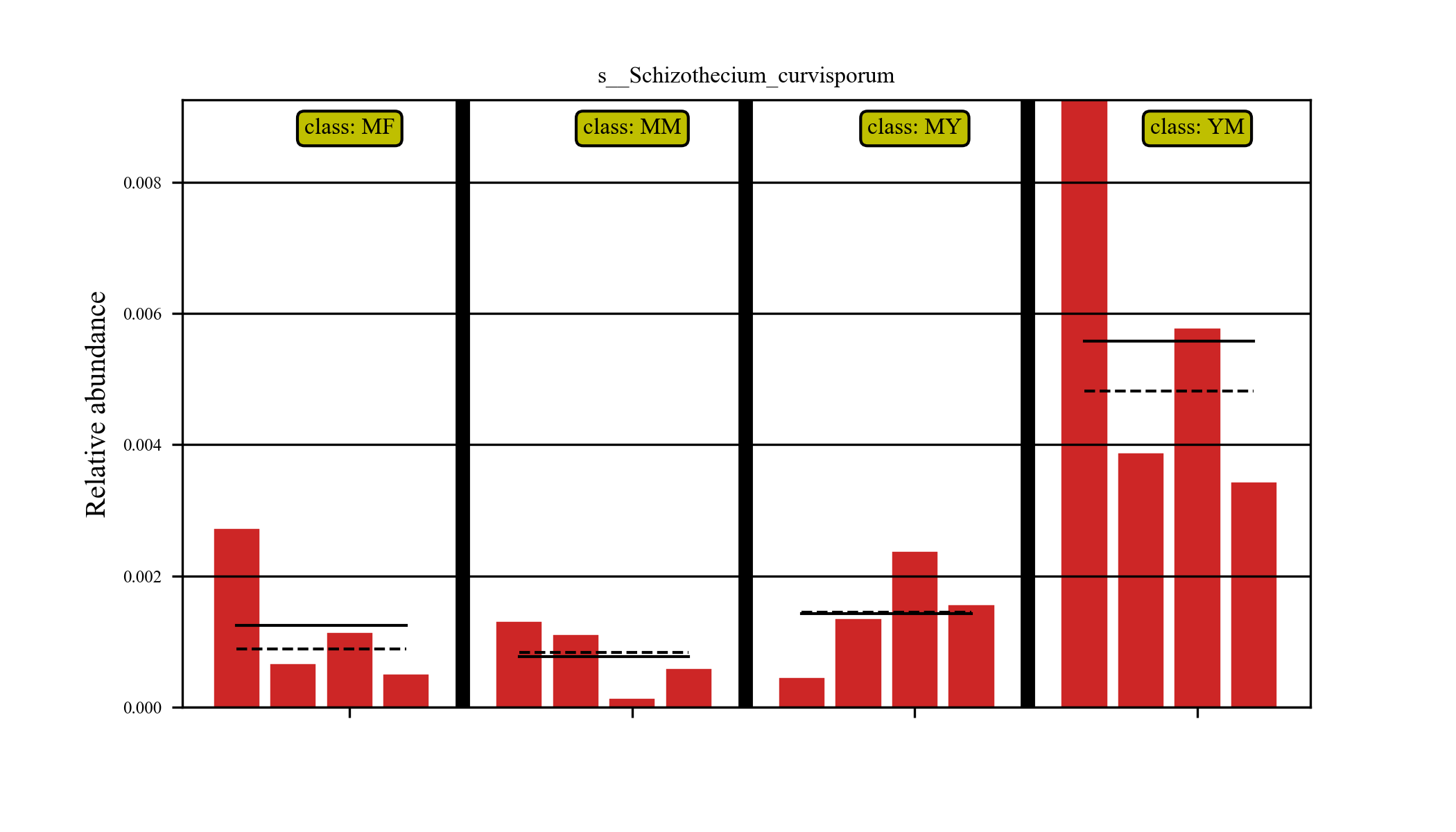

Supplement: Supplementary file 2 [file Data_Sheet_2.ZIP › Supplementary figure 2. fungal biomarker community/1_s__Schizothecium_curvisporum.png]

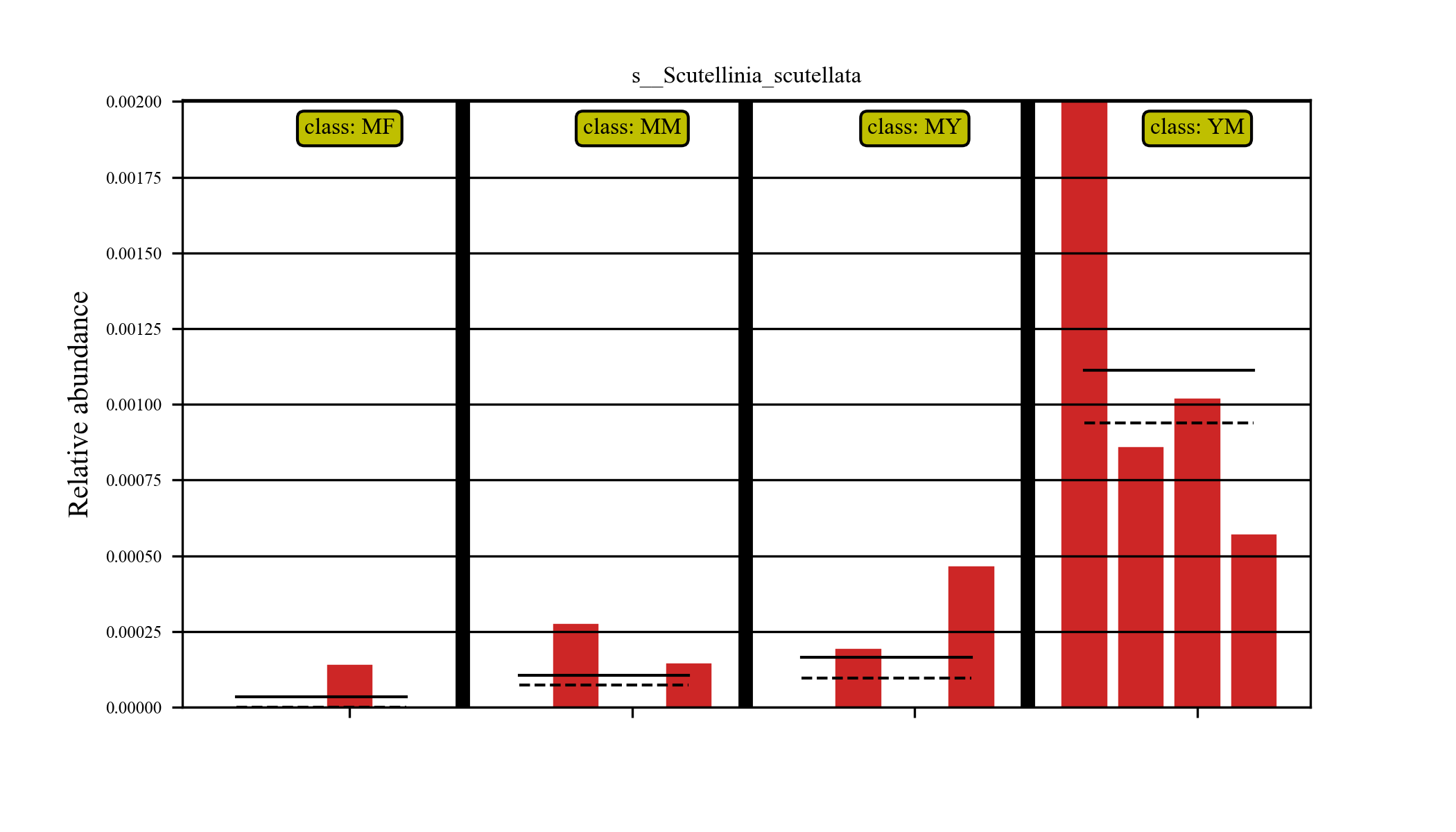

Supplement: Supplementary file 2 [file Data_Sheet_2.ZIP › Supplementary figure 2. fungal biomarker community/1_s__Scutellinia_scutellata.png]

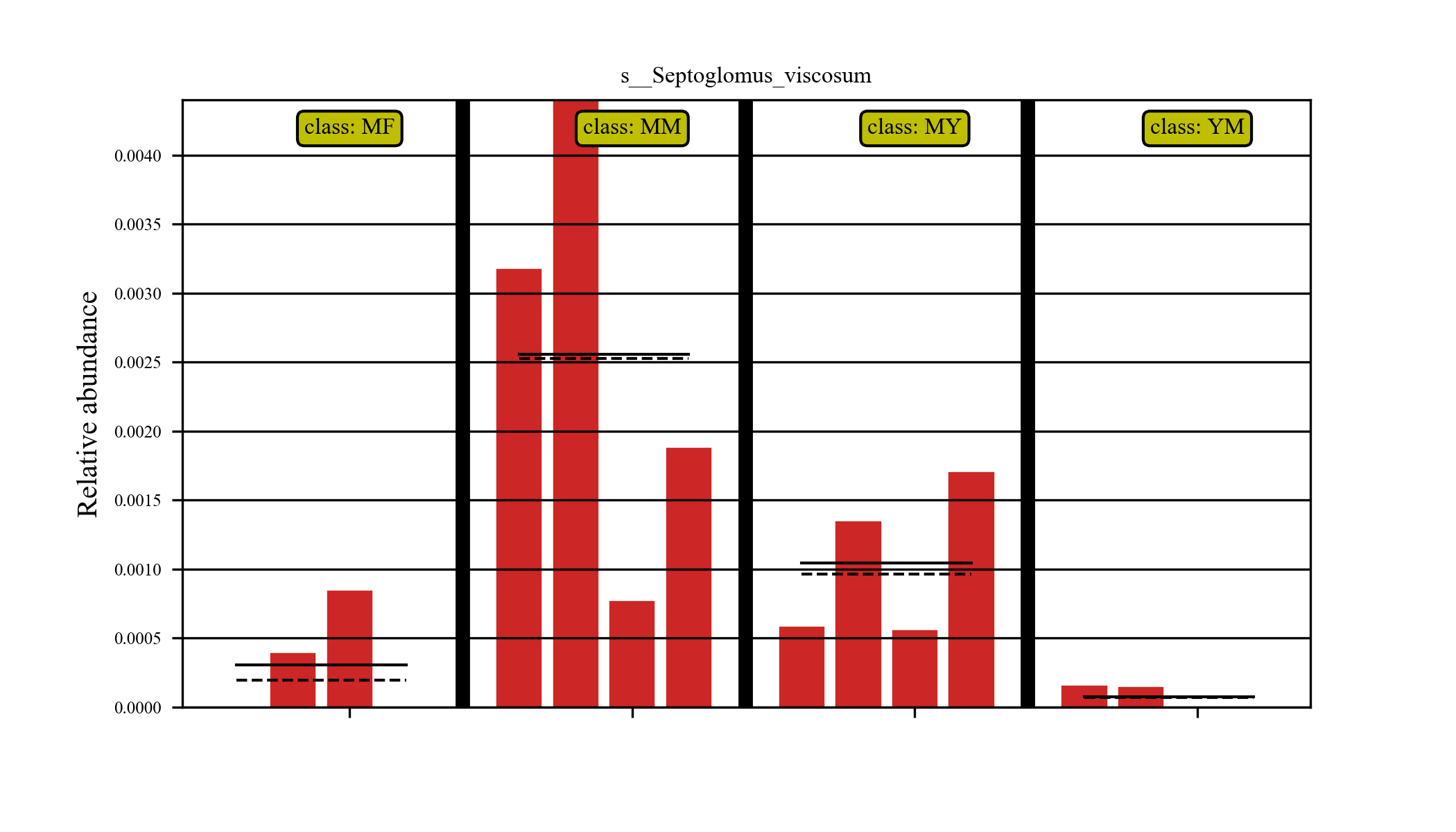

Supplement: Supplementary file 2 [file Data_Sheet_2.ZIP › Supplementary figure 2. fungal biomarker community/1_s__Septoglomus_viscosum.png]

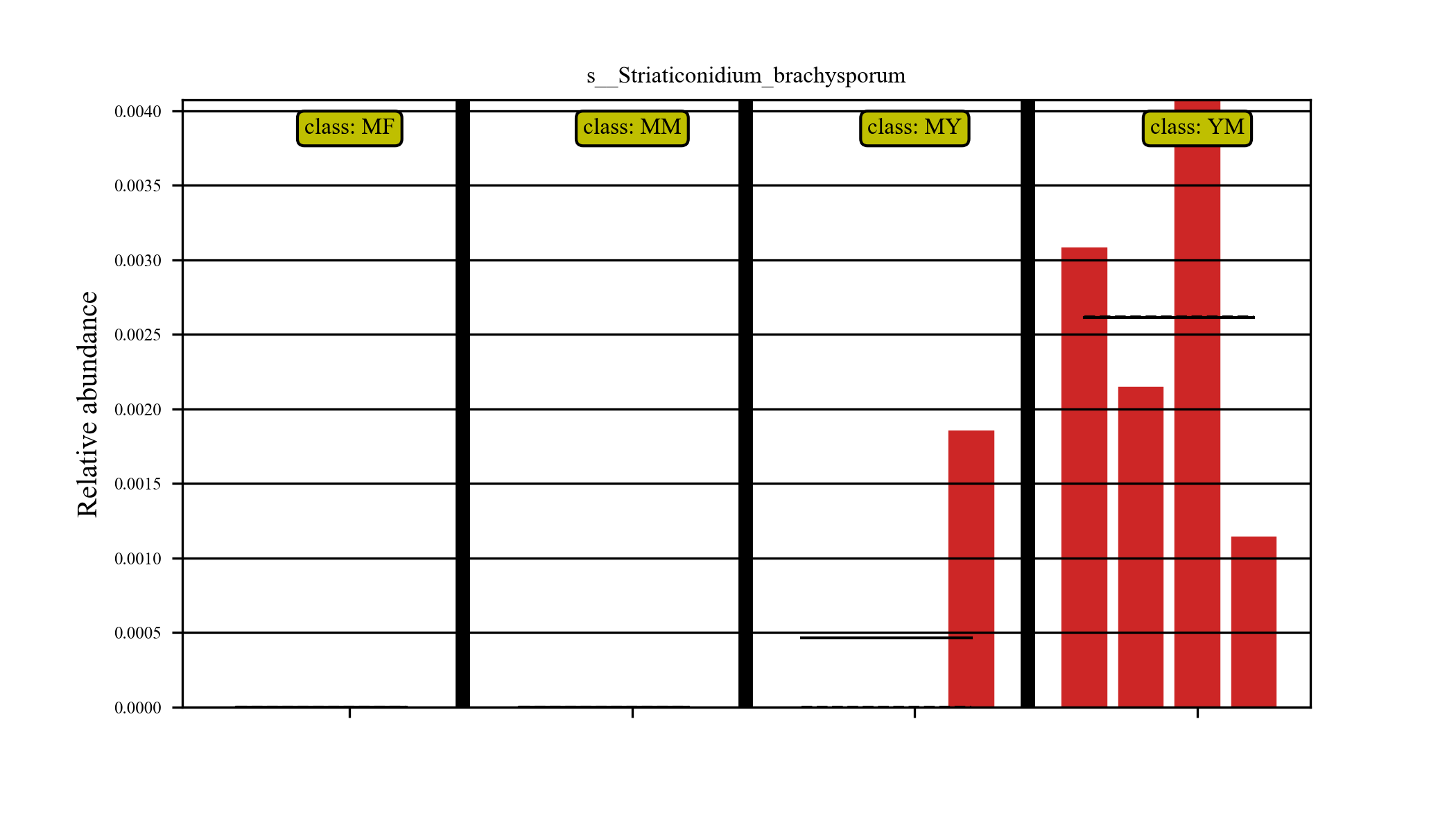

Supplement: Supplementary file 2 [file Data_Sheet_2.ZIP › Supplementary figure 2. fungal biomarker community/1_s__Striaticonidium_brachysporum.png]

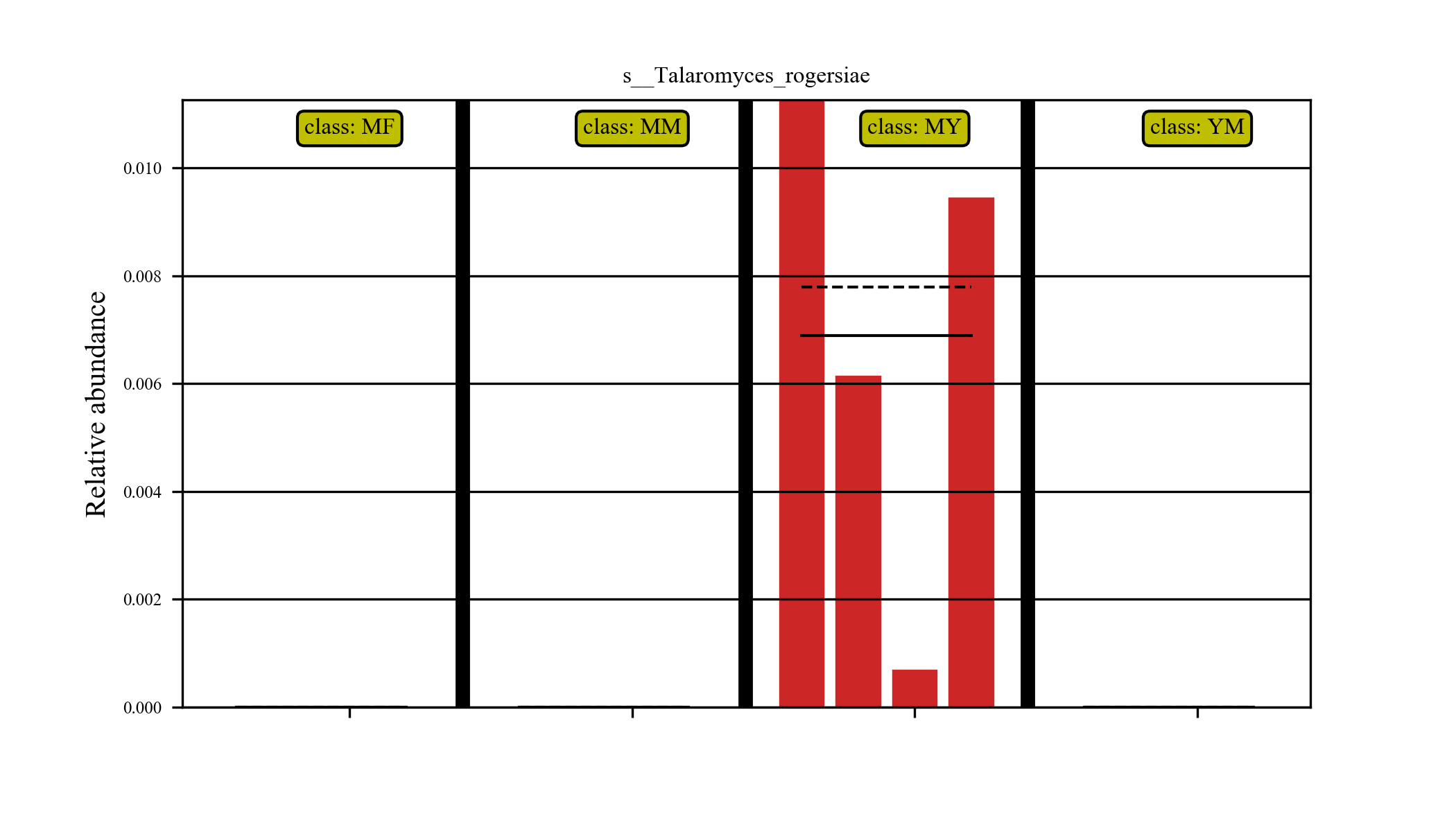

Supplement: Supplementary file 2 [file Data_Sheet_2.ZIP › Supplementary figure 2. fungal biomarker community/1_s__Talaromyces_rogersiae.png]

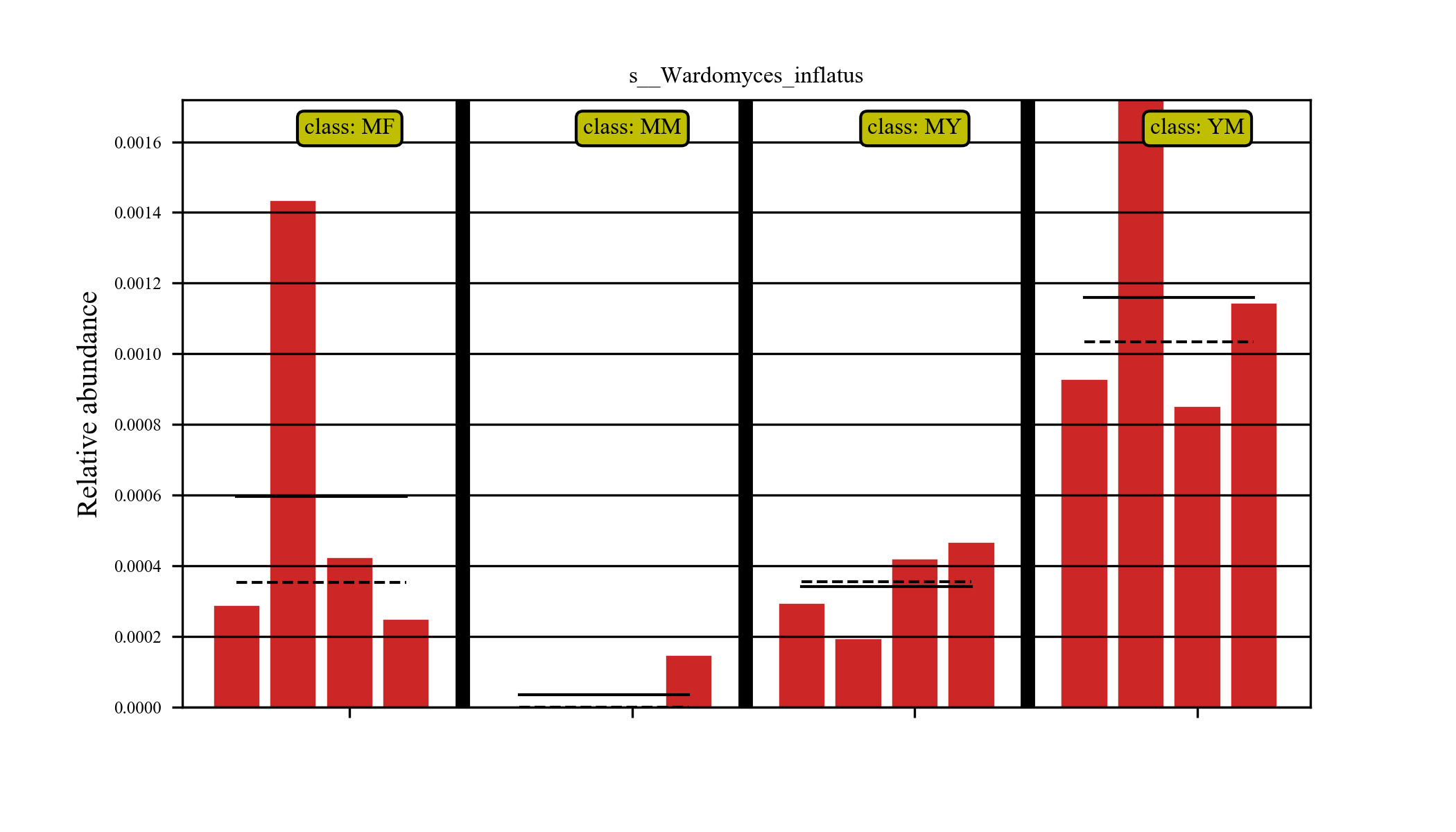

Supplement: Supplementary file 2 [file Data_Sheet_2.ZIP › Supplementary figure 2. fungal biomarker community/1_s__Wardomyces_inflatus.png]
